# Mutate and Conjugate: A Method to Enable Rapid In-Cell Target Validation

**DOI:** 10.1021/acschembio.3c00437

**Published:** 2023-10-24

**Authors:** Adam M. Thomas, Marta Serafini, Emma K. Grant, Edward A. J. Coombs, Joseph P. Bluck, Matthias Schiedel, Michael A. McDonough, Jessica K. Reynolds, Bernadette Lee, Michael Platt, Vassilena Sharlandjieva, Philip C. Biggin, Fernanda Duarte, Thomas A. Milne, Jacob T. Bush, Stuart J. Conway

**Affiliations:** † Department of Chemistry, Chemistry Research Laboratory, University of Oxford, Mansfield Road, Oxford OX1 3TA, United Kingdom; ‡ Department of Chemical Biology, GSK, Gunnels Wood Road, Stevenage, Hertfordshire SG1 2NY, United Kingdom; § Department of Biochemistry, South Parks Road, Oxford OX1 3QU, United Kingdom; ∥ MRC Molecular Haematology Unit, MRC Weatherall Institute of Molecular Medicine, Radcliffe Department of Medicine, University of Oxford, Oxford OX3 9DS, United Kingdom; ⊥ Department of Chemistry & Biochemistry, University of California Los Angeles, 607 Charles E. Young Drive East, P.O. Box 951569, Los Angeles, California 90095-1569, United States

## Abstract

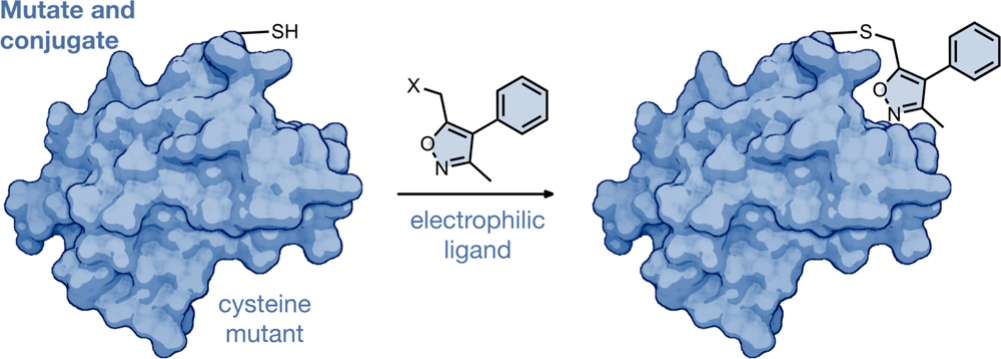

Target validation remains a challenge in drug discovery, which leads to a high attrition rate in the drug discovery process, particularly in Phase II clinical trials. Consequently, new approaches to enhance target validation are valuable tools to improve the drug discovery process. Here, we report the combination of site-directed mutagenesis and electrophilic fragments to enable the rapid identification of small molecules that selectively inhibit the mutant protein. Using the bromodomain-containing protein BRD4 as an example, we employed a structure-based approach to identify the L94C mutation in the first bromodomain of BRD4 [BRD4(1)] as having a minimal effect on BRD4(1) function. We then screened a focused, KAc mimic-containing fragment set and a diverse fragment library against the mutant and wild-type proteins and identified a series of fragments that showed high selectivity for the mutant protein. These compounds were elaborated to include an alkyne click tag to enable the attachment of a fluorescent dye. These clickable compounds were then assessed in HEK293T cells, transiently expressing BRD4(1)^WT^ or BRD4(1)^L94C^, to determine their selectivity for BRD4(1)^L94C^ over other possible cellular targets. One compound was identified that shows very high selectivity for BRD4(1)^L94C^ over all other proteins. This work provides a proof-of-concept that the combination of site-directed mutagenesis and electrophilic fragments, in a mutate and conjugate approach, can enable rapid identification of small molecule inhibitors for an appropriately mutated protein of interest. This technology can be used to assess the cellular phenotype of inhibiting the protein of interest, and the electrophilic ligand provides a starting point for noncovalent ligand development.

## Introduction

The failure rate in the latter stages of the drug discovery pipeline is high, often resulting from a lack of drug efficacy *in vivo*, newly identified safety risks, or incorrect populations enrolled in the clinical trials.^1−3^ The attrition rate in Phase II is particularly acute at approximately 70%, which results mainly from a lack of effective target validation earlier in the drug discovery process.^1,4,5^ It has been estimated that more robust target validation and proof-of-concept studies earlier in the process would increase the probability of a successful Phase II trial to approximately 50% and could reduce the cost of a new molecular entity by 30%.^6,7^

Both chemical and genetic techniques play important roles in target validation. Knock-down/knock-in, gene mutations, and siRNA/shRNA are routinely used to modulate the expression of, or to modify the function of, a specific gene in cells, linking the target with an observed phenotype.^8,9^ However, the effect of the knocking-down protein is complex, affects all functions of a multidomain protein, and can lead to the activation of compensatory pathways in cells. Chemical tools provide temporal control of protein function, allowing phenotypic studies on a native and nonengineered system.^10−12^ Small molecules can also allow the function of an individual protein domain to be removed while leaving scaffolding roles and the function of other domains unaffected. Despite these advantages, the development of small molecules with the exquisite target selectivity required to confidently link target inhibition to a phenotype is challenging and time-consuming.

**Figure 1:**
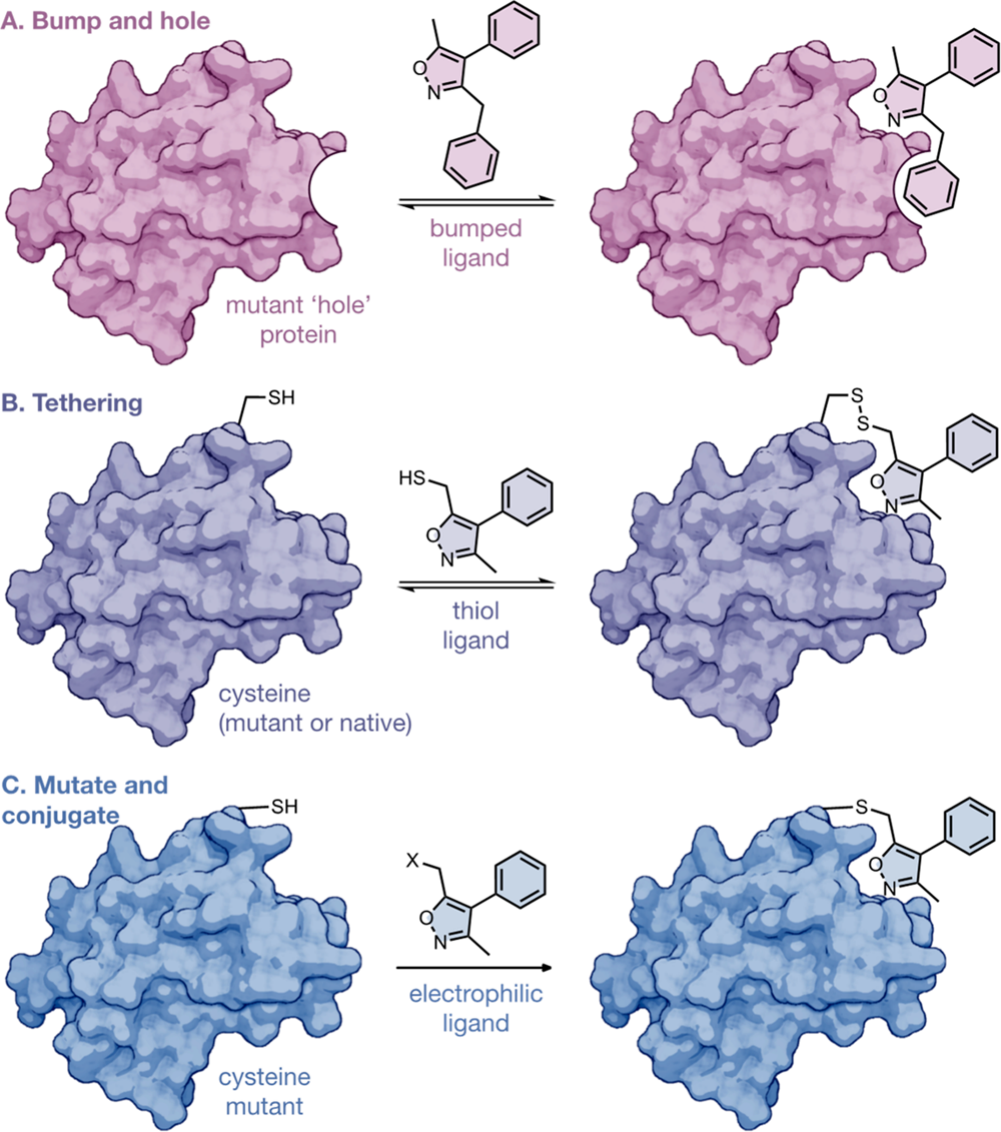
Concepts of (A) bump and hole, (B) tethering, and (C) mutate and conjugate, which all combine protein engineering with small molecules to enable the identification of selective ligands for a mutant protein. Created with BioRender.com.

A combination of genetic and pharmacological approaches allows the accuracy of single mutations to be merged with spatial and temporal control afforded by the use of small molecules. An example is the “bump and hole” methodology that involves making a mutant protein with a smaller amino acid, or “hole”, which can accommodate a “bumped” ligand that binds to the mutant but not the original wild-type protein (Figure 1A).^13^ This approach was pioneered by Schreiber using FKBP12 and cyclophilin^14,15^ and Shokat who applied this approach to generating selective kinase-ligand pairs.^16,17^ More recently, Ciulli has used a bump and hole approach to distinguish between the structurally similar first and second BETs.^18^ An alternative approach is to introduce a mutant cysteine residue to help confer selectivity of a ligand for the mutant protein within a given family.^19,20^

Cysteine residues are poorly conserved across protein classes and represent one of the least abundant natural amino acids (2% of total residues).^21,22^ Their nucleophilicity enables reaction with reactive partners including acrylamides, haloacetamides, disulfides, and α,β-unsaturated esters.^23,24^ Inclusion of these moieties into the protein–ligand results in formation of a covalent bond with the cysteine-containing protein giving a prolonged residence time, a concomitant increase in affinity, potency, and often selectivity.^25−27^ This approach can be employed for WT proteins that contain a poorly conserved cysteine and potentially for engineered proteins that include a mutant cysteine residue. One of the pioneering studies exploiting cysteine chemistry is the tethering approach, in which the presence of native or engineered cysteines is capitalized upon to discover new small molecule inhibitors via the formation of a disulfide bond (Figure 1B).^19,20^

The aim of our work was to develop a technology that combines site-directed cysteine mutation with covalent fragments, allowing rapid elucidation of ligands to inhibit the function of the mutant protein domain (Figure 1C). The advantage of this approach is that the covalent nature of the ligand avoids the need for iterative rounds of development to increase affinity for the target protein. Additionally, the combination of the mutant cysteine and the electrophilic ligand can provide selectivity over other possible off-targets. A similar approach to the development of a selective ligand for the EphB1 kinase was reported by Kung et al.,^28^ but this work employed an already established kinase ligand, rather than electrophilic fragments.

We chose to exemplify this approach using BRD4 as the target protein. While there is not an urgent need for the development of further BRD4 ligands, the availability of structural information on both BRD4 bromodomains makes it an excellent testing ground for this technology. BRD4, together with BRD2, BRD3 and BRDT, comprise the BET family of bromodomain-containing proteins, which function as readers of acetyl lysine (KAc) residues on histones and other proteins.^29^ All members of the BET family are characterized by the presence of two bromodomains, BD1 and BD2, located at the N-terminus of the protein.^30−32^ Owing to the highly conserved nature of the members of the BET family, the development of ligands that selectively target the different isoforms, and between the two tandem domains, has been an important challenge and only relatively few highly selective ligands for the discrete domains have been reported to date.^33−36^ Our strategy potentially provides a rapid method to identify inhibitors that selectively inhibit protein domains with very similar sequences, for example, BD1 over BD2 or vice versa.

In this work, we compared a structure-based design approach starting from a known KAc-mimic moiety to screening a library of diverse covalent fragments. While the former can be exploited for proteins with known binding ligands, as in the case of BRD4, the second could potentially afford a strategy to identify ligands for a target for which none currently exist. This approach allowed us to discover, without the need for extensive SAR studies, a ligand that selectively binds to BRD4(1) over BRD4(2). This “mutate and conjugate” approach has the potential to provide a general strategy for rapidly identifying covalent ligands to assist in the target validation of a protein of interest.

## Results and Discussion

### Design, Expression, and Structural Validation of a Cysteine Mutant Bromodomain

**Figure 2:**
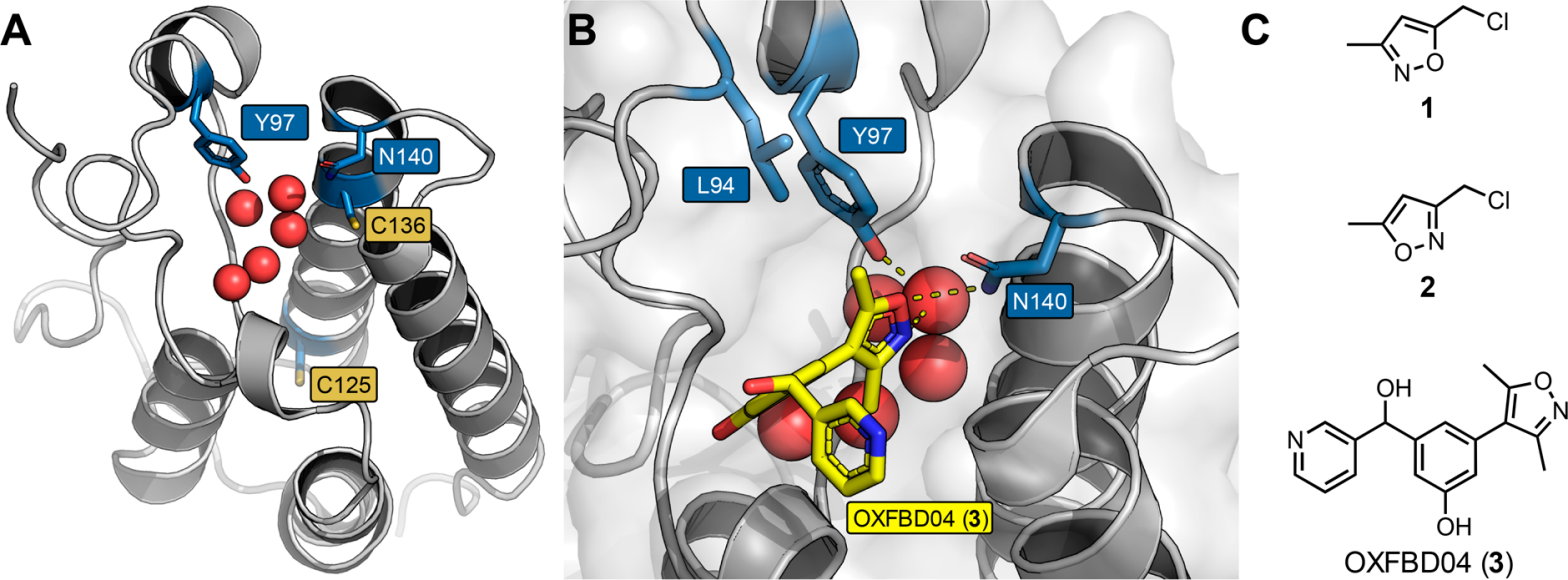
(A) X-ray crystal structure of BRD4(1)^WT^ with the key KAc binding residues, Y97 and N140, and the two native cysteines, C125 and C136, highlighted (carbon = blue; PDB ID: 6FSY). The structure of (R)-OXFBD04 (**3**) has been removed from the original crystal structure to better highlight the cysteine residues in BRD4(1)^WT^.^37^ (B) Analysis of the X-ray crystal structure of (R)-OXFBD04 (**3**) (carbon = yellow) bound to BRD4(1)^WT^ (carbon = blue; PDB ID: 6FSY) suggested that the ZA loop region might be a suitable location to introduce a mutation. L94 was identified as a candidate for cysteine mutation because it is located proximal to the ligand binding site but does not play a significant role in the WT function of the protein. (C) The structures of the two reactive isoxazole fragments **1** and **2** and the structure of OXFBD04 (**3**).

Before introducing a Cys mutant into the first bromodomain of BRD4 [BRD4(1)], we wanted to rule out the possibility that the native Cys residues C125 and C136 (Figures 2A and S1), would react with electrophilic fragments. To test this, we incubated two KAc-mimicking methylisoxazole-based reactive fragments (**1** and **2**, Figure 2C) with purified BRD4(1)^WT^. After 20 h, no covalent labeling of the protein by either fragment was detected using LCMS, indicating that neither of the native cysteines are highly reactive nucleophiles (Figure S2).

Having demonstrated that the WT protein is not susceptible to reaction with electrophilic fragments, we next determined suitable sites to incorporate a Cys mutation. Analysis of the X-ray crystal structure of BRD4(1)^WT^ bound to OXFBD04 (**3**), a 3,5-dimethylisoxazole-based BRD ligand that we previously developed (PDB ID: 6FSY) (Figure 2B),^37^ indicated that the ZA loop region might be a suitable area for mutagenesis as it is located above the KAc binding pocket and is therefore unlikely to have a negative impact on native KAc binding. The L94 residue, which is conserved across the BET family (Figure S1), is part of the ZA loop and is located above the 5-position methyl group of the OXFBD04 isoxazole. This placement suggests that attachment of a 5-position electrophile could locate the L94C cysteine and the electrophile proximal, allowing them to react. Interestingly, Baud et al.^39^ and Runcie et al.^38^ employed two mutations at this position in their bump and hole approaches, suggesting that modifications at this position are tolerated by the protein. An L94 V mutation led to a 2-fold decrease in tetra-acetylated H4-mimicking peptide (H4_1–20_(KAc)_4_) binding affinity,^38^ while the L94A mutation decreased the affinity by 10-fold.^39^

To further investigate the impact of L94C on protein stability, molecular dynamics (MD) simulations using GROMACS 2016.4 were employed, which compared the backbone root-mean-square deviation (RMSD) and backbone root-mean-square fluctuation (RMSF) of BRD4(1)^WT^ and BRD4(1)^L94C^. Simulations, performed over a 50 ns time scale (Figure S3) showed that the calculated RMSD values for both BRD4(1)^WT^ and BRD4(1)^L94C^ were comparable with deviations stable and remaining below 2.5 Å for BRD4(1)^L94C^ after equilibrium had been reached. This result suggested that the L94C mutation would not significantly disrupt the stability or structure of the protein.

**Figure 3:**
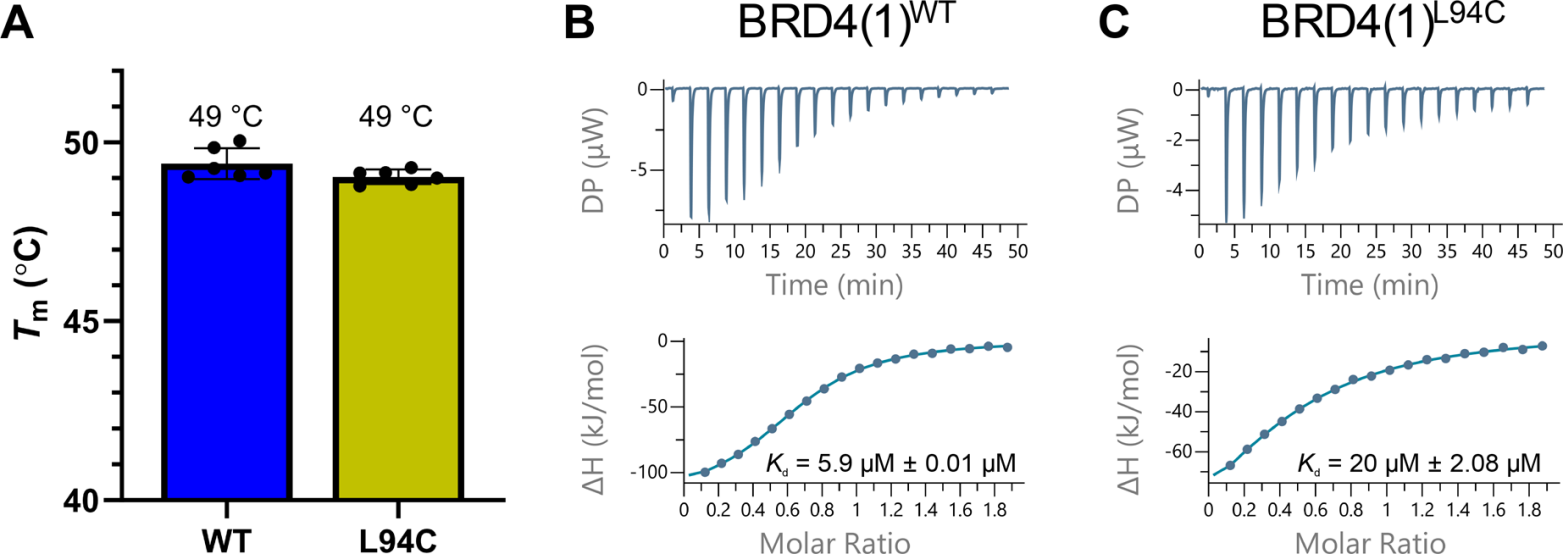
Biophysical data comparing the stability and functionality of BRD4(1)^WT^ and BRD4(1)^L94C^. (A) DSF derived melting temperatures for BRD4(1)^WT^ (blue bar) and BRD4(1)^L94C^ (yellow bar). The mean values are shown above the bars (*n* = 6). Error bars represent the s.e.m. Representative ITC traces for H4_1–20_(KAc)_4_ peptide affinity for BRD4(1)^WT^ (B) and BRD4(1)^L94C^ (C) with the mean *K*_d_ ± s.e.m. values quoted (*n* = 3).

We therefore produced BRD4(1)^L94C^, which was obtained in high yields and purified to homogeneity using immobilized metal affinity chromatography (IMAC) and size exclusion chromatography (SEC) (Figures S4–S6). Both BRD4(1)^L94C^ and BRD4(1)^WT^ had the same melting temperature of 49 °C, determined using differential scanning fluorimetry (DSF), suggesting that the protein stability had not been compromised by the mutation (Figure 3A). Furthermore, BRD4(1)^L94C^ retained the ability to bind the H4_1–20_(KAc)_4_ peptide with only a 3.5-fold decrease in affinity compared to BRD4(1)^WT^ (*K*_d_ values of 20 and 5.9 μM, respectively, determined using isothermal titration calorimetry (ITC), Figures 3B,C and S7 and Table S1). Having confirmed that the H4_1–20_(KAc)_4_ peptide bound to BRD4(1)^L94C^, we could employ it in an AlphaScreen assay. OXFBD04 (**3**) prevented binding of the H4_1–20_(KAc)_4_ peptide to BRD4(1)^L94C^ with an IC_50_ value of 80 ± 9 nM, which is comparable to the value measured for BRD4(1)^WT^ of 183 ± 25 nM (Figures S12–S13).^37^

**Figure 4:**
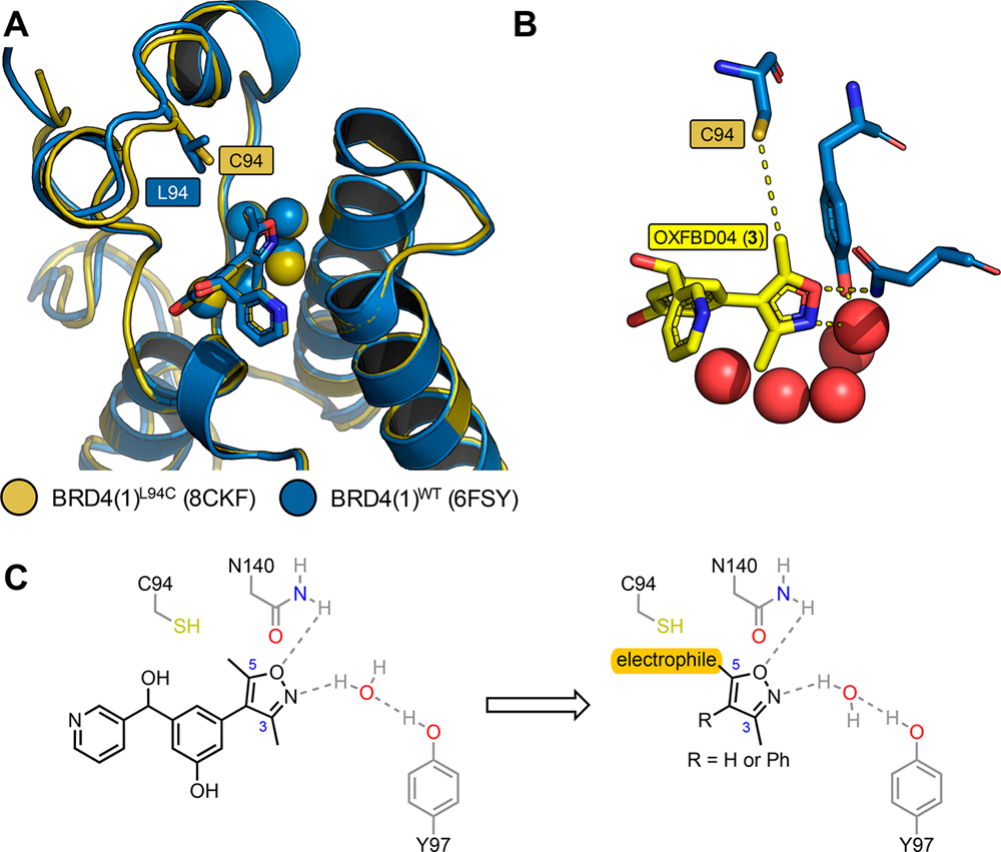
(A) Overlaid structures of (R)-OXFBD04 (**3**) bound to BRD4(1)^WT^ (carbon = blue; PDB ID: 6FSY) and BRD4(1)^L94C^ (carbon = yellow; PDB ID: 8CKF). There is overall high structural alignment with a backbone alignment RMSD of 0.41 Å. The structure of (S)-OXFBD04 bound to BRD4(1)^L94C^ has been removed for clarity. Structures were aligned using the “align” command in PyMOL. (B) The X-ray crystal structure of (R)-OXFBD04 bound to BRD4(1)^L94C^ (carbon = blue; PDB ID: 8CKF), showing that (R)-OXFBD04 (**3**) retains the key interactions with N140 and Y97. The structure of (S)-OXFBD04 bound to BRD4(1)^L94C^ has been removed for clarity. (C) A schematic showing the interactions of OXFBD04 with BRD4(1)^L94C^ guiding the design of electrophilic fragment ligands for this protein.

To confirm the structural similarity of BRD4(1)^WT^ and BRD4(1)^L94C^, we obtained an X-ray crystal structure of *rac*-OXFBD04 (**3**) bound to BRD4(1)^L94C^ (1.88 Å resolution; PDB ID: 8CKF). Alignment and overlay of this crystal structure with the X-ray structure of (*R*)-OXFBD04 (**3**) bound to BRD4(1)^WT^ (PDB ID: 6FSY) revealed a high overall structural similarity (Figure 4A). While there is some small variation in the ZA loop where the mutation is located (backbone alignment variation of 0.41 Å RMSD for the full protein), OXFBD04 (**3**) retains the key interactions with N140 and Y97 of BRD4(1)^L94C^ (Figure 4B,C). In the previously reported X-ray crystal structure of (*R*)-OXFBD04 (**3**) bound to BRD4(1)^WT^ (PDB ID: 6FSY), electron density for only one enantiomer was visible,^37^ despite the compound being added as a racemate. However, in the case of BRD4(1)^L94C^, both enantiomers were observed in the electron density map (Figure S8).

### Identification of Reactive Fragments That Covalently Bind to BRD4(1)^L94C^

We next sought to identify electrophilic fragments that would bind covalently and selectively to BRD4(1)^L94C^. To achieve this, we took two different approaches: (i) a structure-guided approach starting from the KAc-mimicking dimethylisoxazole moiety^32,37,40−43^ and (ii) screening of a diverse library of published reactive fragments (GSK diverse fragment screen).^44^

The position of OXFBD04 (**3**) when bound to BRD4(1)^L94C^ (Figure 4B) suggested that attachment of the electrophile at the 5-position of the dimethylisoxazole ring would locate the electrophile and C94 close in space (Figure 4C). To retain the key noncovalent interactions between the isoxazole KAc mimic and the protein, we focused on the regioisomer in which the 5-position of the isoxazole ring was functionalized with a range of electrophiles (compounds **4**, **7**, **8**, **10**, **13**, **16**, **18**, **19**–**21**, and **23**, Table 1).^45,46^ Five fragments bearing a phenyl at the 4-position group were also synthesized to evaluate the influence of increased lipophilic interactions with the protein on covalent protein labeling (compounds **6**, **12**, **14**, **15**, and **22**, Table 1). To confirm that the 3-substituted regioisomers would engage less favorably with the KAc binding pocket, we synthesized four fragments (compounds **5**, **9**, **11**, and **17**, Table 1) based on this scaffold.

**Table 1:**
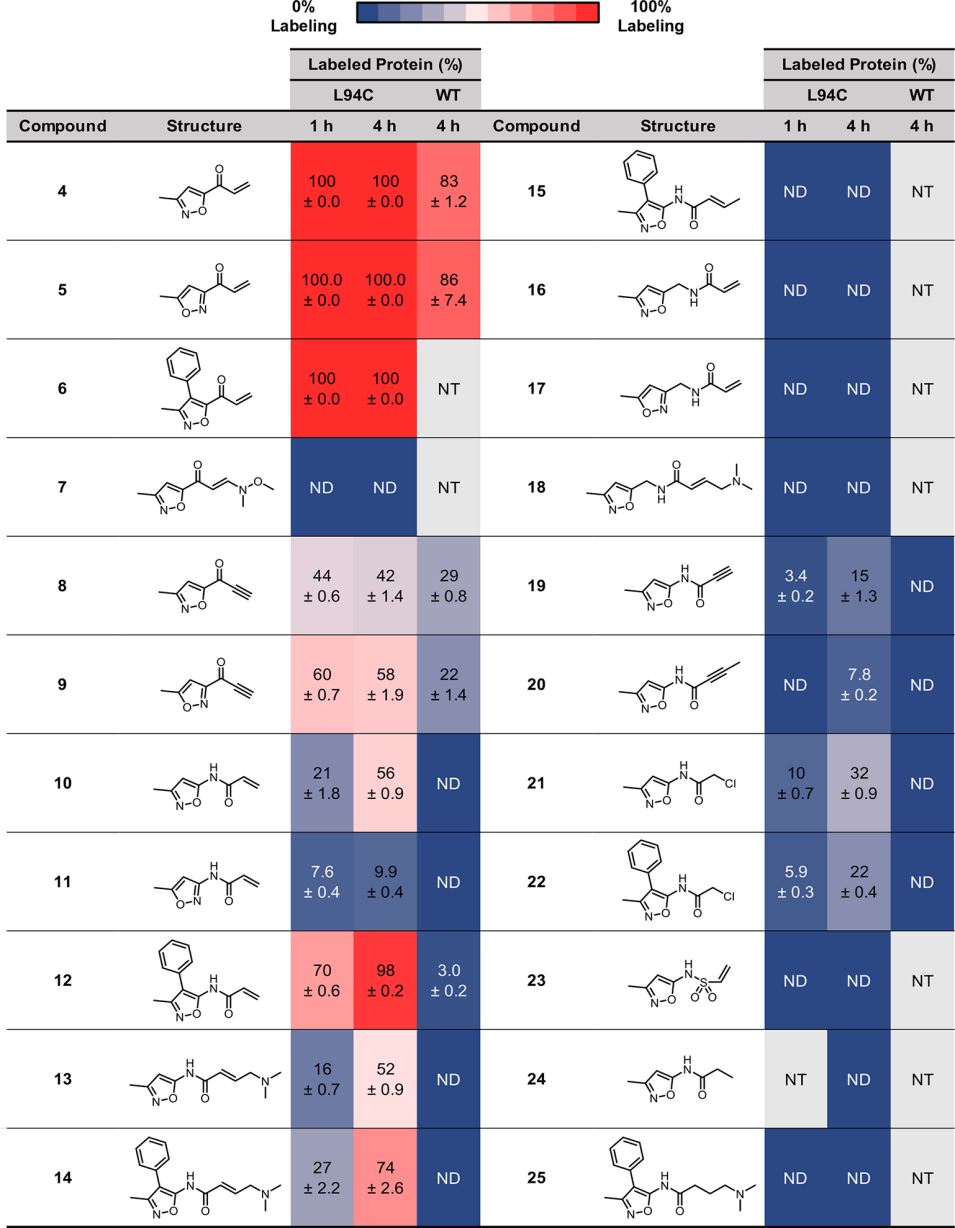
Percentage Labeling for the Isoxazole-Based Structure-Guided Covalent Fragments Tested at a Concentration of 50 μM against BRD4(1)^L94C^ and BRD4(1)^WT^^a^

a The color scale is shown, where low levels of protein labeling are shown in blue and high levels are shown in red. Values quoted for hits are the mean of three independent experiments ± s.e.m. ND = not detectable. NT = not tested.

**Figure 5:**
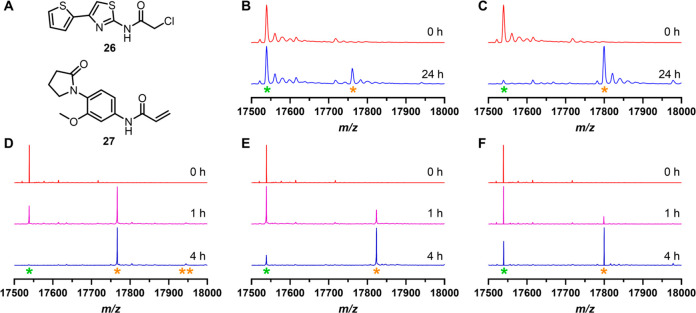
(A) Structures of the two “hit” fragments (**26** and **27**) identified through the diverse reactive fragment screen. Deconvoluted mass spectra showing labeling of BRD4(1)^L94C^ by fragments **26** (B) and **27** (C) under “labeling assay 2” conditions. Deconvoluted mass spectra showing time-dependent labeling of BRD4(1)^L94C^ by fragments **12** (D), **14** (E), and **27** (F) under “labeling assay 1” conditions. The *Y*-axis shows the relative signal intensity (%). The green asterisk shows peaks corresponding to unlabeled protein, and the orange asterisks show peaks corresponding to labeled protein, with the number of asterisks representing the number of labeling events.

The resulting library of 22 fragments was screened for BRD4(1)^L94C^ binding at a concentration of 50 μM using a protein LCMS assay (referred to as “labeling assay 1”). Fragments which showed a mass corresponding to the protein plus bound fragment with a peak intensity of >2% were classified as “hits”. To assess the selectivity of the fragments for the labeling of C94, hits were also screened against BRD4(1)^WT^ under the same conditions. In this screen, two 3-methylisoxazole-based fragments, **12** and **14**, were observed to label BRD4(1)^L94C^ (Table 1 and Figure S9). The acrylamide-based fragment, **12**, labeled 98% of BRD4(1)^L94C^ after 4 h. While 95% of the protein was labeled once, 3% of the protein was labeled twice (Figure 5D). This fragment showed good selectivity over BRD4(1)^WT^ with a single labeling event of 3% intensity observed after 4 h (Figure S9). Fragment **14**, designed by appending a β-dimethylaminomethyl acrylamide to the structure of **12**, led to 74% of singly labeled BRD4(1)^L94C^ after 4 h with no labeling of BRD4(1)^WT^ observed (Table 1 and Figure 5E). Interestingly, the corresponding fragments lacking the phenyl ring at the 4-position showed a lower percentage of BRD4(1)^L94C^ labeling (56% and 52% for **10** and **13**, respectively), suggesting that the addition of the phenyl group increases the fragments affinity for BRD4(1)^L94C^ or induces a conformation that allows for more favorable reactivity between the electrophile and C94. Finally, as initially suggested by the X-ray structure (*vide supra*), only one of the 5-methyl analogues, **11**, displayed selective labeling of BRD4(1)^L94C^. However, only 9.9% of labeled BRD4(1)^L94C^ was observed, substantially lower than the 56% labeling observed for the corresponding 3-methyl regioisomer **10** (Table 1). Other 5-methylisoxazole derivatives possessing reactive enone and ynone electrophiles were observed to label both BRD4(1)^L94C^ and BRD4(1)^WT^ unselectively (Table 1 and Figure S9).

To investigate whether screening of an unbiased set of covalent fragments could identify ligands for BRD4(1)^L94C^, we evaluated 250 previously published cysteine-reactive compounds, comprising 138 acrylamide- and 112 chloroacetamide-functionalized reactive fragments with molecular weights ranging from 152 to 300 Da.^44^ Due to the higher throughput of this screening assay, the conditions varied compared to “labeling assay 1”. In this case, 1 μM BRD4(1)^L94C^ was incubated with either a chloroacetamide fragment (50 μM) or an acrylamide fragment (200 μM) at 4 °C for 24 h (referred to as “labeling assay 2”). Samples were then analyzed using protein LCMS, where a positive hit was classified as a single protein labeling event with a mass shift corresponding to the mass of the fragment of ±2 Da and a labeled peak of >20% intensity. Of the 250 diverse reactive compounds screened against BRD4(1)^L94C^, only one chloroacetamide and one acrylamide fragment were classified as hits (Figures 5A and S10). The chloroacetamide hit (**26**, Figure 5A) displayed 27% labeling of BRD4(1)^L94C^ (Figure 5B), and the acrylamide hit (**27**, Figure 5A) showed 89% labeling of BRD4(1)^L94C^ (Figure 5C). Given the difference in the two screening conditions, a selection of isoxazole fragments identified by “labeling assay 1” were included as positive (**10**, **13**, and **21**) and negative (**17**) controls. All of these control compounds showed protein labeling trends comparable to those obtained from labeling assay 1, confirming the overlap between the two assay conditions (Figure S11).

As fragment **27** displayed a single covalent labeling of BRD4(1)^L94C^ at a level of 89%, this compound represented a promising candidate for further investigation (Figure S10). When the labeling was assessed under “labeling assay 1” conditions, used for the structure-guided fragment screening, compound **27** labeled 61% of BRD4(1)^L94C^ with no covalent binding to BRD4(1)^WT^ detected (Figures 5F and S12). The shorter time point used in “labeling assay 1” (4 h instead of 24 h used in “labeling assay 2”) likely accounts for the lower level of labeling displayed by the fragment (61% of labeling versus 89%). Overall, two compounds from the structure-guided approach (**12** and **14**, Table 1) and one compound from the diverse fragment-based screening (**27**, Table 2) were selected for further evaluation.

**Table 2:**
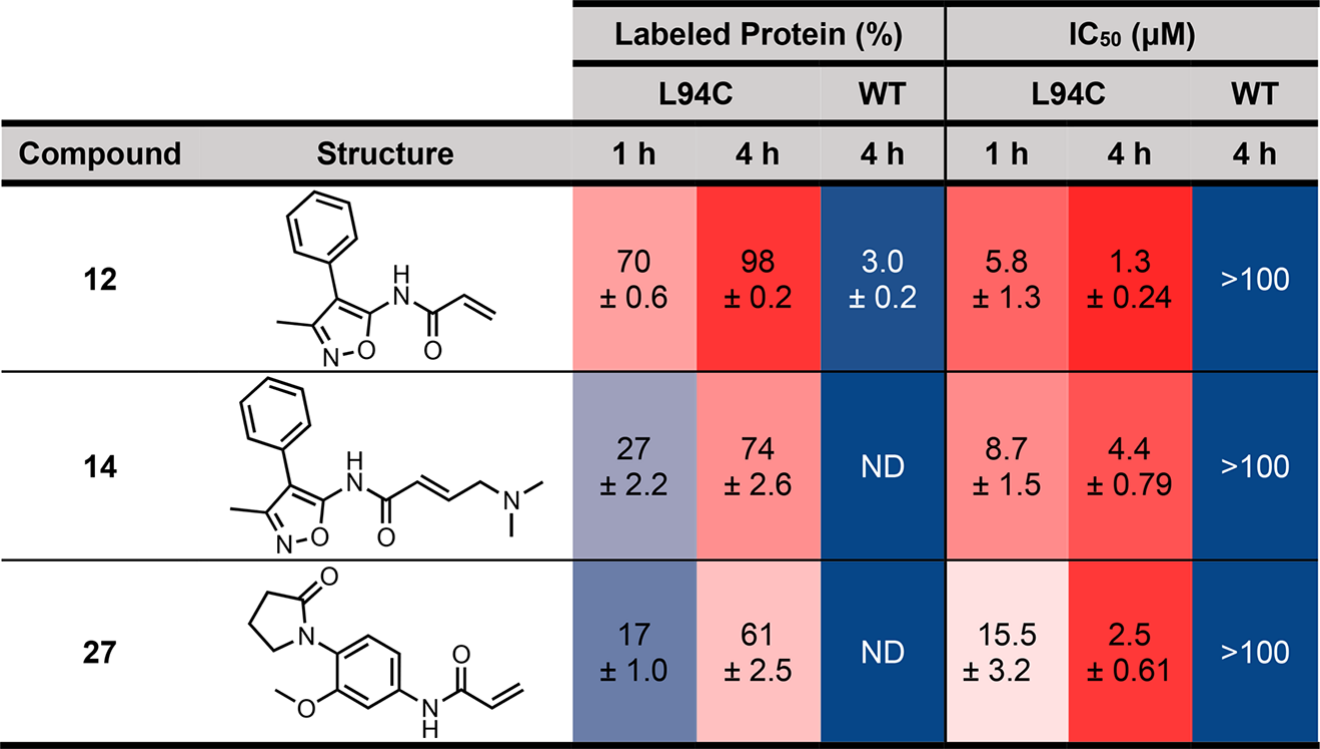
Percentage Labeling and AlphaScreen IC_50_ Values for Fragments **12**, **14**, and **27** against BRD4(1)^L94C^ and BRD4(1)^WT^^a^

a Values quoted are the mean of three in dependent experiments ± s.e.m. ND = not detectable.

Using an AlphaScreen assay,^47,48^ the three identified fragments were evaluated for their ability to displace the H4_1–20_(KAc)_4_ peptide from BRD4(1)^L94C^ and BRD4(1)^WT^. The compounds were incubated at RT for 1 or 4 h in the presence of the biotinylated H4_1–20_(KAc)_4_ peptide, and either BRD4(1)^L94C^ or BRD4(1)^WT^. Under these conditions, all three fragments displaced the peptide from BRD4(1)^L94C^, which is consistent with the fragments binding to BRD4(1)^L94C^ in the expected location (Table 2). In all cases, the IC_50_ values at 4 h were lower than those seen at 1 h, which is characteristic of covalent inhibition (Figure S13).^26,49^ The longer the mutant protein and electrophile are incubated together, the more time there is for a covalent interaction to occur. None of the fragments showed significant inhibition of BRD4(1)^WT^ after 4 h (Figure S14), confirming their selective labeling of BRD4(1)^L94C^. This result also shows that a covalent reaction is required for the fragment to bind the bromodomain with sufficient affinity to displace the H4_1–20_(KAc)_4_ peptide.

### Clickable Probes to Evaluate In-Cell Selectivity of Reactive Fragments Targeting BRD4(1)^L94C^

With three promising fragments in hand that showed high selectivity for BRD4(1)^L94C^ in a cell-free environment, we next developed “clickable” analogs by functionalization with an alkyne handle. The aim of developing these probes was to evaluate their target engagement with BRD4(1)^L94C^ in live cells and assess their proteome-wide target selectivity.

**Scheme 1:**
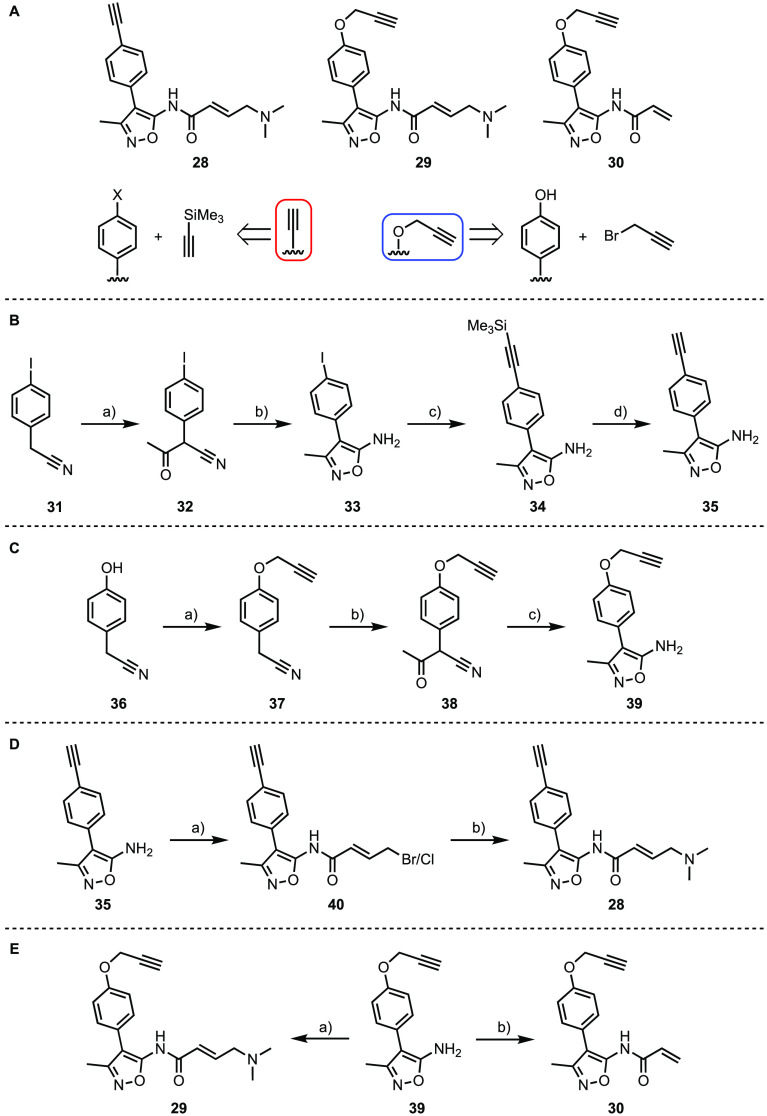
Syntheses of Clickable Probes (A) Synthesis of the clickable probes **28**, **29**, and **30**, which are based on the fragments **12** and **14**. Two alkyne variations were explored, utilizing either a Sonogashira coupling or an alkylation approach. (B) Synthesis of amine scaffold **35**. Reagents and conditions: (a) EtOAc, NaH, THF, 10% v/v DMF, 0 °C to rt, 18 h, 50–86%, *n* = 2; (b) hydroxylamine hydrochloride, 10% w/v aq. Na_2_CO_3_, EtOH, reflux, 3 h, 51–99%, *n* = 5; (c) TMS acetylene, Pd(PPh_3_)_2_Cl_2_, CuI, DMF, Et_3_N, rt, 1.5 h, 67–74%, *n* = 5; (d) K_2_CO_3_, MeOH, rt, 1 h, 87–92%, *n* = 4. (C) Synthesis of amine scaffold **39**. Reagents and conditions: (a) propargyl bromide, K_2_CO_3_, CH_2_Cl_2_, 0 °C to rt, 3 h, 99%; (b) EtOAc, NaH, THF, 10% v/v DMF, 0 °C to rt, 4 h, 91%; (c) hydroxylamine hydrochloride, 10% w/v aq. Na_2_CO_3_, EtOH, reflux, 1 h, 83%. (D) (a) (i) (*E*)-4-Bromocrotonic acid, oxalyl chloride, DMF, CH_2_Cl_2_, 0 °C to rt, 15 h; (ii) **35**, pyridine, CH_2_Cl_2_, 0 °C, 5 h, 37% (based on a 20% Cl:80% Br ratio determined using ^1^H NMR); (b) (i) NaI, acetone, 50 °C, 1 h; (ii) dimethylamine (2.0 M in THF), K_2_CO_3_, DMF, 0 °C, 1.5 h, 28% over two steps. (E) (a) (i) (*E*)-4-(Dimethylamino)but-2-enoic acid hydrochloride, oxalyl chloride, DMF, THF, 0 °C to rt, 1.5 h; (ii) **39**, NMP, 0–5 °C, 22 h, 37%; (b) acryloyl chloride, pyridine, CH_2_Cl_2_, 0 °C to rt, 2.5 h, 18%.

One analogue of compound **12** was designed, which contains an *O*-propargyl handle to give fragment **30** (Scheme 1). Two analogs of compound **14** were developed, one with a terminal alkyne directly attached to the phenyl ring (**28**) and another with an *O*-propargyl handle (**29**) (Scheme 1A). Compound **35** with the alkyne directly attached to the phenyl ring was synthesized from 2-(4-iodophenyl)acetonitrile (**31**, Scheme 1B). A Claisen-type condensation between **31** and ethyl acetate,^50^ followed by condensation with hydroxylamine and subsequent intramolecular cyclization,^51^ gave the 5-amino-3-methylisoxazole **33**. A Sonogashira coupling was used to append the alkyne motif to iodide **33**, giving TMS-protected alkyne **34** (Scheme 1B). Deprotection of the TMS group under basic conditions yielded free alkyne **35**. Compound **39** with a propargyl group attached to the phenol was synthesized from 2-(4-hydroxyphenyl)acetonitrile **36** (Scheme 1C). In this case, the propargyl group was first attached to the phenol by using an alkylation reaction. Subsequent condensation and cyclization, as described above, gave the 5-amino-3-methylisoxazole **39**. Compound **35** was reacted with (*E*)-4-bromocrotonic acid and oxalyl chloride followed by halogen exchange with NaI and substitution by dimethylamine to give the desired product **28** (Scheme 1D). Compound **29** was obtained by treatment of **39** with (*E*)-4-(dimethylamino)but-2-enoic acid hydrochloride in the presence of oxalyl chloride and DMF.^52^ Compound **30** was obtained by acylation with acryloyl chloride (Scheme 1E).

**Scheme 2:**
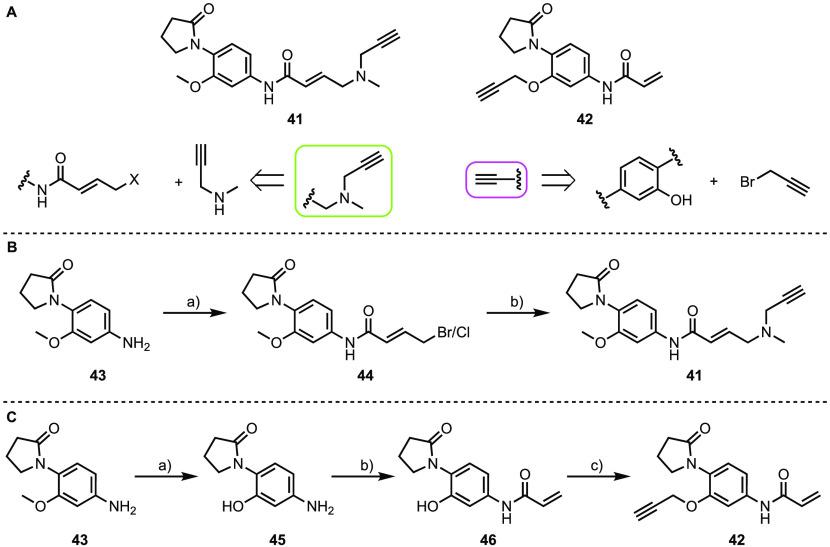
Syntheses of **41** and **42** (A) Clickable probes based on fragment **27**, discovered using the GSK diverse fragment screen. Two alkyne variations were explored, using either an amination or an alkylation approach. (B) Synthesis of **41**. Reagents and conditions: (a) (i) (*E*)-4-Bromocrotonic acid, oxalyl chloride, DMF, THF, 0 °C to rt, 45 min; (ii) **43**, NMP, 0 °C, 75 min, 94% (based on a 40% Cl:60% Br ratio determined using ^1^H NMR); (b) *N*-methylpropargylamine, KI, K_2_CO_3_, DMF, 0 °C to rt, 2 h, 28%. (C) Synthesis of **42**. Reagents and conditions: (a) BBr_3_ (1.0 M in CH_2_Cl_2_), CH_2_Cl_2_, 0 °C to rt, 23 h, 78%; (b) acryloyl chloride, pyridine, CH_2_Cl_2_, 0 °C to rt, 7 h, 43%; (c) propargyl bromide, K_2_CO_3_, DMF, rt, 16 h, 35%.

Compound **27** possesses a γ-lactam structure, which is a known KAc binding motif, derived from *N*-methyl-2-pyrrolidone (NMP).^29,40,47,53,54^ We have previously shown that the γ-lactam-based solvent NMP is a weak bromodomain ligand.^40,47^ Hilton-Proctor et al.^53,54^ subsequently used the NMP motif as the basis for developing higher affinity bromodomain ligands. Given these observations, we designed the clickable probes based on compound **27** so as to leave the γ-lactam unaltered. In compound **41**, a *N*-methylpropargylamine is used to add both the electrophile and the alkyne to the aniline nitrogen (Scheme 2A).^55−58^ In compound **42**, the aniline nitrogen is functionalized as an acrylamide, while a propargyl group is attached to the phenolic oxygen to provide the click handle (Scheme 2A).^51−54^ Compound **43** was purchased and reacted with (*E*)-4-bromocrotonic acid in the presence of oxalyl chloride and DMF to give **44**. Treatment of **44** with KI resulted in halogen exchange followed by substitution with *N*-methylpropargylamine to give **41** (Scheme 2B). To synthesize **42**, compound **43** was demethylated by using BBr_3_, giving **45**, which was selectively *N*-acylated by treatment with acryloyl chloride to yield **46**. Reaction of **46** with propargyl bromide afforded the desired product **42** (Scheme 2C).

To ensure that the addition of the alkyne handle did not hinder covalent labeling of BRD4(1)^L94C^, all of the clickable probes were evaluated using “labeling assay 1” (Table 3). Compounds **28**–**30** showed a similar or increased percentage of labeling compared to the parent fragments **12** and **14**. Like its parent fragment **12**, compound **30** displayed low levels of nonspecific labeling, leading to a second labeling event accounting for 3% of total labeled protein (Figure S15). Compound **41** showed only 2% of BRD4(1)^L94C^ labeling after 4 h, demonstrating that modification of the acrylamide moiety was not tolerated. Compound **42**, however, showed 37% of BRD4(1)^L94C^ labeling after 1 h and 82% after 4 h. This result shows that attachment of the alkyne moiety to the phenolic oxygen is tolerated, although the slower labeling might reflect a lower intrinsic BRD4(1) affinity for this ligand class, in addition to lower electrophilicity, compared to compounds **28**–**30**.

**Table 3:**
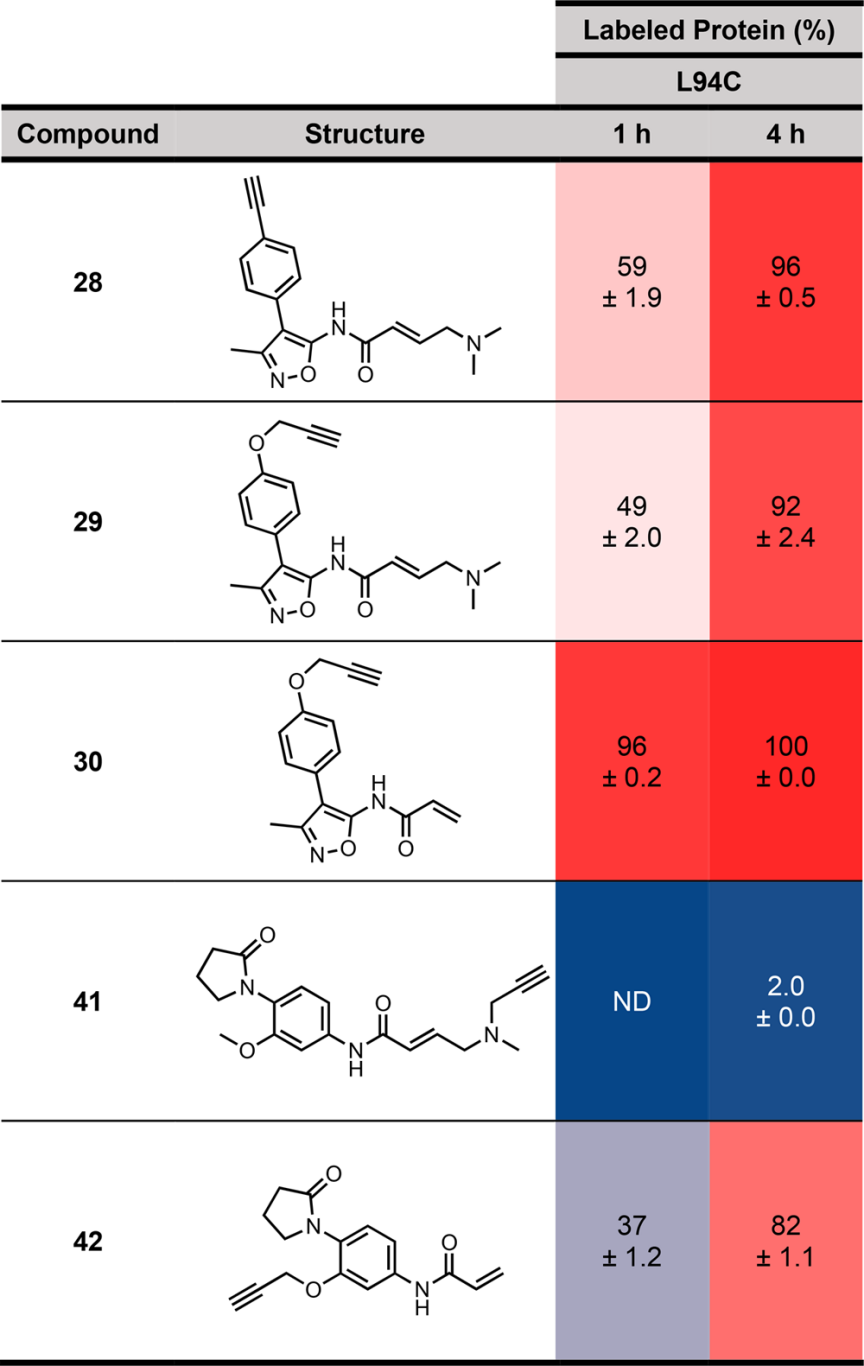
Percentage of Labeling of BRD4(1)^L94C^ by Clickable Probes after 1 and 4 h by LCMS Analysis^a^

a Values quoted are the result of three Independent experiments ± s.e.m. ND, not detectable.

**Figure 6:**
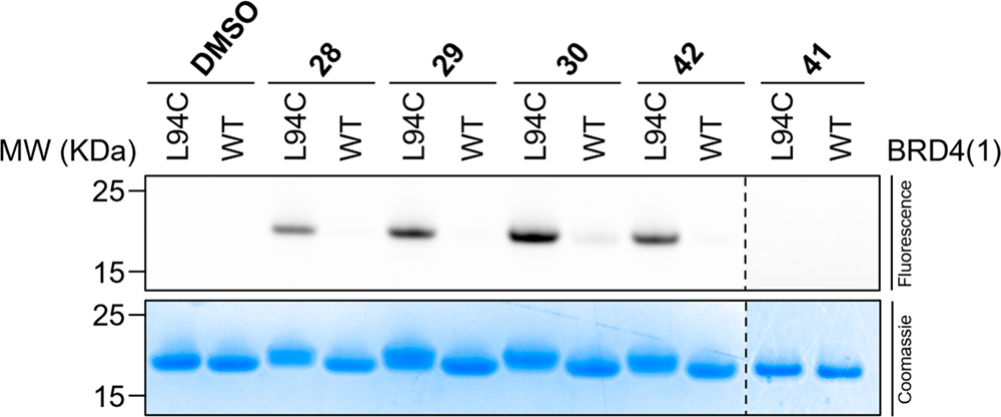
Clickable probes **28**, **29**, **30**, and **42** covalently label BRD4(1)^L94C^ and undergo a subsequent CuAAC reaction with TAMRA-PEG_3_ azide to fluorescently label the protein, as shown by in-gel fluorescence imaging. Compounds **28**, **29**, **30**, **41**, and **42** (50 μM) were incubated with purified BRD4(1)^L94C^ or BRD4(1)^WT^ (10 μM) for 4 h at 37 °C, followed by CuAAC reaction with TAMRA-PEG_3_ azide for 1 h and separation of components by SDS-PAGE. For all compounds other than **30**, only labeling of BRD4(1)^L94C^ was observed. Compound **30** showed a low level of BRD4(1)^WT^ labeling.

When **28**, **29**, **30**, and **42** were incubated with either purified BRD4(1)^L94C^ or BRD4(1)^WT^ and subjected to a copper(I)-catalyzed alkyne–azide click reaction (CuAAC) reaction with the fluorescent TAMRA-PEG_3_-azide dye, all clickable probes labeled the mutant protein and could be visualized using fluorescence imaging of a gel (Figure 6). While probes **28**, **29**, **30**, and **42** showed high levels of BRD4(1)^L94C^ labeling, compound **30** also displayed low levels of BRD4(1)^WT^ labeling, and compound **41** showed no labeling of either BRD4(1)^L94C^ or BRD4(1)^WT^, consistent with the protein LCMS data. Compound **28** produced a lower fluorescence intensity than **29**, **30**, or **42**, perhaps indicating that direct attachment of the alkyne to the phenyl ring might hinder the CuAAC reaction.

**Figure 7:**
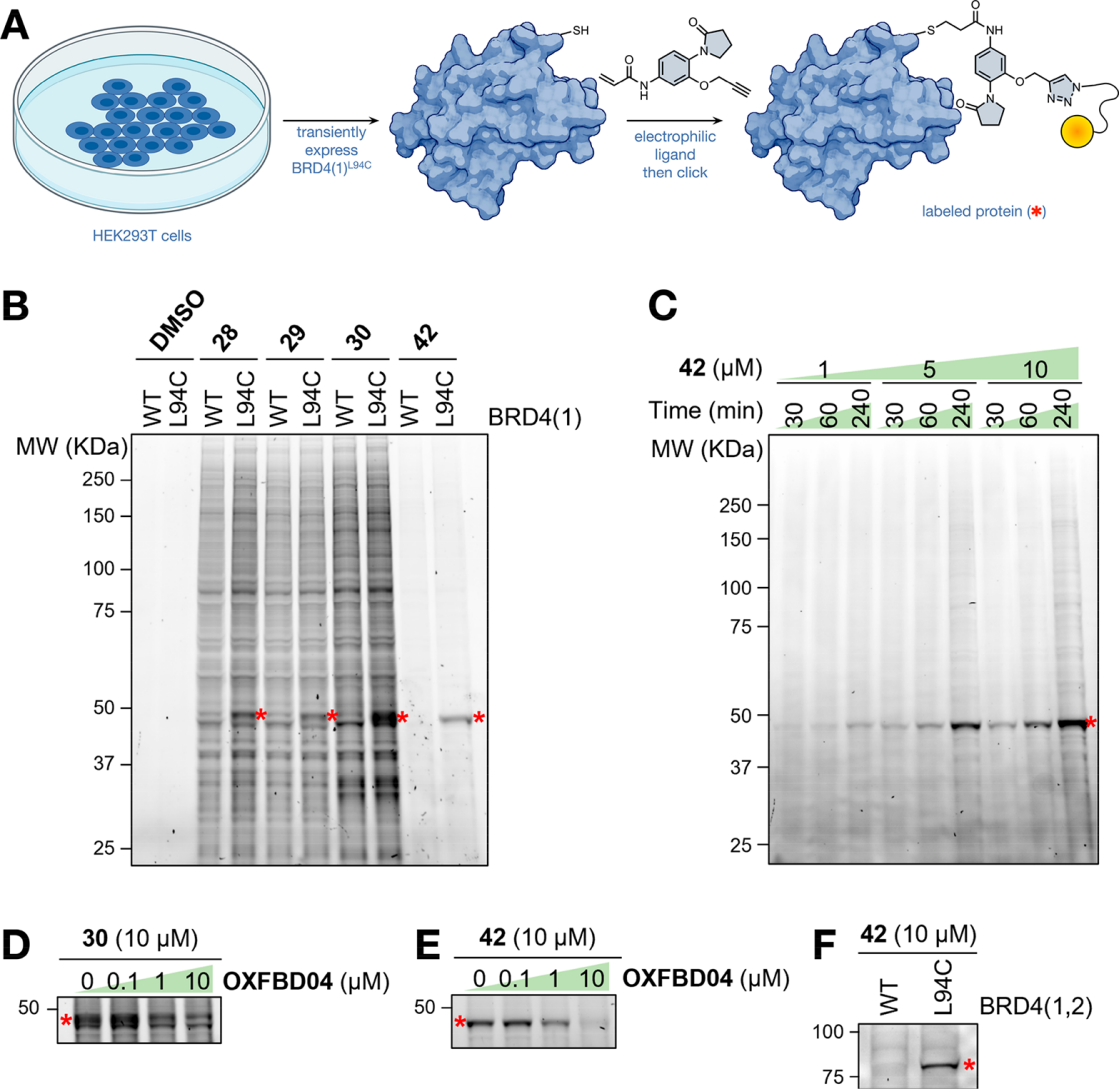
(A) Graphical representation of the in-cell labeling assay protocol. Created with BioRender.com. (B) In-gel fluorescence, using TAMRA-PEG_3_ azide, showing proteome-wide labeling by clickable probes **28**–**30** and **42** in live HEK293T cells transiently expressing BRD4(1)^WT^ or BRD4(1)^L94C^. The red asterisks indicate the labeling of BRD4(1)^L94C^. (C) In-cell labeling of BRD4(1)^L94C^ by **42** is time and dose dependent. (D) A competition assay using OXFBD04 (**3**) and HEK293T cells transiently expressing BRD4(1)^L94C^ confirmed live cell target engagement of BRD4(1)^L94C^ by **30**. (E) A competition assay using OXFBD04 (**3**) and HEK293T cells transiently expressing BRD4(1)^L94C^ confirmed live cell target engagement of BRD4(1)^L94C^ by **42**. (F) Clickable probe **42** binds selectively to BRD4(1,2)^L94C^ but not BRD4(1,2)^WT^, confirming that the labeling results from a selective covalent interaction with the L94C-containing first bromodomain of BRD4(1,2)^L94C^.

To evaluate proteome-wide selectivity of the clickable probes, HEK293T cells transiently expressing BRD4(1)^L94C^ or BRD4(1)^WT^ were incubated with 10 μM of probes **28**–**30** and **42** for 1 h before the cells were lysed and the CuAAC reaction with TAMRA-PEG_3_-azide was conducted (Figure 7A). SDS-PAGE separation and analysis using in-gel fluorescence and Coomassie blue staining revealed high levels of off-target binding to endogenous proteins for all three of the isoxazole-derived probes **28**–**30** (Figures 7B and S18). Of these three probes, **30** exhibited the highest level of BRD4(1)^L94C^ engagement, with a stronger fluorescent band at the expected molecular weight (∼49 kDa) in cells expressing BRD4(1)^L94C^, which was not visible in the BRD4(1)^WT^ expressing cells (Figure 7B). In contrast, the γ-lactam-based probe **42**, displayed exquisite proteome-wide selectivity toward BRD4(1)^L94C^ with little evidence of it binding to any other proteins. Treatment of HEK293T cells transiently expressing BRD4(1)^L94C^ with **42** at concentrations ranging from 1 to 10 μM showed that covalent labeling of this protein is dose dependent (Figure 7C). A time course of 0.5–4 h revealed that labeling occurs in a time-dependent manner, with maximum labeling observed at the longer time point (Figure 7C).

BRD4(1)^L94C^ target engagement of compound **30** was investigated using a competition experiment with the BRD4(1)^L94C^ ligand OXFBD04 (**3**). Increasing the concentration of OXFBD04 (**3**) resulted in a concomitant reduction in the level of BRD4(1)^L94C^ labeling by compound **30** (Figures 7D and S21). Increasing concentrations of OXFBD04 (**3**) also led to decreased levels of BRD4(1)^L94C^ labeling by **42** (Figures 7E and S22). These results are consistent with both compounds residing in the KAc binding pocket and covalently labeling BRD4(1)^L94C^. Compound engagement with BRD4(1)^L94C^ was further validated by using an immunoprecipitation (IP) assay (Figure S22). Here, transfected HEK293T cells expressing StrepII-tagged BRD4(1)^L94C^ or StrepII-tagged BRD4(1)^WT^ were incubated with compound **42** before being lysed. The StrepII-tagged bromodomains were then pulled down using anti-StrepII antibodies, and the samples were separated by SDS-PAGE for analysis. Observation of a fluorescent band only for the cells expressing BRD4(1)^L94C^, but not those expressing BRD4(1)^WT^, confirmed selective engagement of BRD4(1)^L94C^.

To investigate whether the covalent ligands showed selective labeling of BRD4(1)^L94C^ over BRD4(2), the in-cell labeling assay was repeated using HEK293T cells transiently expressing either the wild-type tandem bromodomain [BRD4(1,2)^WT^] or the tandem bromodomain bearing the L94C mutation within the first domain [BRD4(1,2)^L94C^]. Compound **42** was observed to selectively label BRD4(1,2)^L94C^, which was observed as a fluorescent band corresponding to ∼80 kDa, while no covalent engagement with BRD4(1,2)^WT^ was observed (Figures 7F and S23). This result confirms that the labeling results from a selective covalent interaction with the L94C-containing first bromodomain of BRD4(1,2)^L94C^ (Figure 7F).

While compound **30** shows robust labeling of BRD4(1)^L94C^ and not BRD4(1)^WT^, it has low cellular selectivity and interacts with a large number of other targets. It is likely that this promiscuity results from low intrinsic affinity of the fragment for BRD4(1): 4-phenyl-3,5-dimethylisoxazole has an IC_50_ = 50 μM for BRD4(1),^59^ coupled with the relatively high electrophilicity of the acrylamide moiety. Remarkably, however, compound **42** shows almost complete selectivity for BRD4(1)^L94C^ and does not substantially label BRD4(1)^WT^ or any other proteins. Fluorescent labeling studies with BRD4(1,2)^L94C^ confirmed this selectivity. The parent compounds **12** and **27** have similar IC_50_ values for BRD4(1)^L94C^ (Table 2), suggesting that subtle variations in the reactivity or vector of the electrophile are responsible for the difference in selectivity observed. The electron-withdrawing nature of the isoxazole ring likely increases the electrophilicity of the acrylamide in **30**, while the electron-donating nature of the phenyl ring substituents in **42** has the opposite effect. This idea is supported by the less complete labeling of purified BRD4(1)^L94C^ by **42**, compared to that by **30** (Table 3). The lower reactivity of the electrophile means that compound **42** does not react covalently with proteins with which it interacts with transiently. The intrinsic noncovalent affinity of **42** for BRD4(1)^L94C^ is required to provide sufficient protein residency time to allow a covalent reaction to occur.

## Conclusion

In this work, we describe a new methodology, “mutate and conjugate”, that combines site-directed mutagenesis and the use of covalent inhibitors to rapidly identify selective small molecule ligands for a protein of interest. Our technology was exemplified by inserting an L to C mutation into BRD4(1) and screening a library of small reactive fragments to discover a highly selective acrylamide-based probe (**27**), which possesses high selectivity for BRD4(1)^L94C^ over BRD4(1)^WT^. The identified probe was functionalized with an alkyne handle (**42**) allowing for fluorescent labeling of proteins that were covalently engaged by the probe in living cells. In this cellular environment, the probe demonstrated high selectivity for BRD4^L94C^ over other endogenous proteins while showing no binding to the second BRD4 bromodomain. Such a compound represents the ideal starting point to selectively probe the cellular function of target protein and was arrived at with minimal SAR optimization.

The “mutate and conjugate” methodology allows for the rapid development of selective probes to investigate the biological function of target proteins or protein domains, circumventing the limitations normally encountered while developing chemical probes. Although we selected BRD4 as the ideal target to test our methodology, this approach can be potentially applied to newly emerging or poorly understood targets for which no selective ligands are yet available. In this work, we compared two different approaches: a structure-based method and screening of a diverse set of fragments; in this case, the latter proved more effective. With the advancement of CRISPR-Cas9 technologies and site-directed mutagenesis of endogenous proteins, the application of “mutate and conjugate” will improve the reliability of target validation and phenotypic studies while expanding the ligandable proteome.

## Supporting Information Available

Biological methods, chemical experimental section, computational methods sections, supplementary figures and tables, and NMR spectra and HPLC traces for new compounds (PDF)

## Abbreviations

BD1: first bromodomain
BD2: second bromodomain
BET: bromodomain and extra-terminal domain
BRD: bromodomain
CuAAC: copper(I)-catalyzed alkyne–azide cycloaddition
IMAC: immobilized metal affinity chromatography
IP: immunoprecipitation, ITC, isothermal titration calorimetry
KAc: acetyl lysine
MD: molecular dynamics
ND: not detectable
NMP: *N*-methyl-2-pyrrolidone
RMSF: root-mean-square fluctuation
RMSD: root-mean-square deviation
SDS-PAGE: sodium dodecyl-sulfate polyacrylamide gel electrophoresis
SAR: structure activity relationship, SEC, size exclusion chromatography
SEM: standard error of the mean
shRNA: short hairpin RNA
siRNA: small interfering RNA
