# Supplementary material for: Mutate and Conjugate: A Method to Enable Rapid In-Cell Target Validation
Source: ACS Chem Biol. 2023 Oct 24;18(11):2405–17. doi: 10.1021/acschembio.3c00437 (PMC10660337; doi:10.1021/acschembio.3c00437)
Supplement: Supplementary file 1 — cb3c00437_si_001.pdf [file cb3c00437_si_001.pdf]

Mutate and Conjugate: A Method to Enable Rapid In-Cell Target Validation

Adam M. Thomas,[1] Marta Serafini,[1] Emma K. Grant,[2] Edward A. J. Coombs,[1] Joseph P. Bluck,[1,3] Matthias Schiedel,[1] Michael A. McDonough,[1] Jessica K. Reynolds,[1] Bernadette Lee,[1] Michael Platt,[1] Vassilena Sharlandjieva,[4] Philip C. Biggin,[3] Fernanda Duarte,[1] Thomas A. Milne,[4] Jacob T. Bush,[2] Stuart J. Conway[1,5,*]

Supporting Information

[*] Corresponding author: stuart.conway@chem.ox.ac.uk

[1]Department of Chemistry, Chemistry Research Laboratory, University of Oxford, Mansfield Road, Oxford OX1 3TA, United Kingdom

[2]Department of Chemical Biology, GSK, Gunnels Wood Road, Stevenage, Hertfordshire, SG1 2NY, UK

[3]Department of Biochemistry, South Parks Road, Oxford OX1 3QU, U.K.

[4]MRC Molecular Haematology Unit, MRC Weatherall Institute of Molecular Medicine, Radcliffe Department of Medicine, University of Oxford, Oxford, OX3 9DS, United Kingdom.

[5]Department of Chemistry & Biochemistry, University of California Los Angeles, 607 Charles E. Young Drive East, P. O. Box 951569, Los Angeles, California, 90095-1569, USA

Table of contents

Supplementary Figures and Tables 3

General Chemistry Experimental 26

Synthetic Procedures and Compound Data 28

General Biology Methods 63

Protein X-ray Crystallography 82

Computational Studies 84

NMR Spectra for Novel Compounds 85

HPLC Traces of Biologically Tested Compounds 167

References 194

Supplementary Figures and Tables

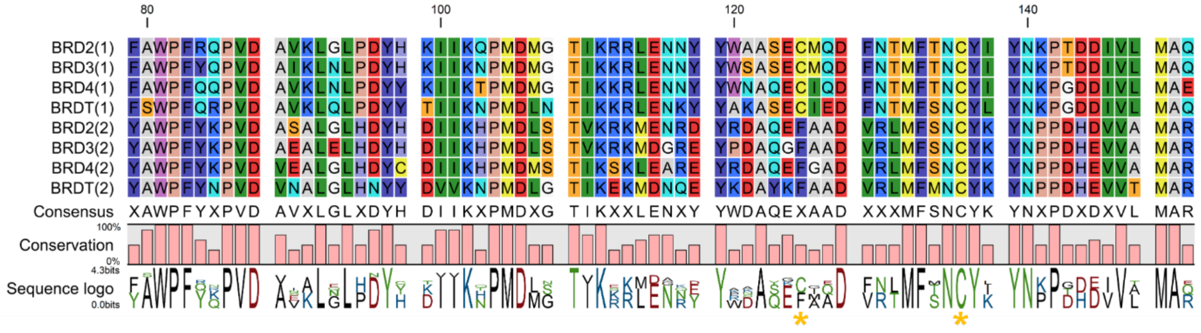

Figure S1. Protein sequence alignment of the BET bromodomains (BRD4(1) numbering). Sequences were aligned using CLC Genomics Workbench 22. The orange asterisks highlight C125 and C136.

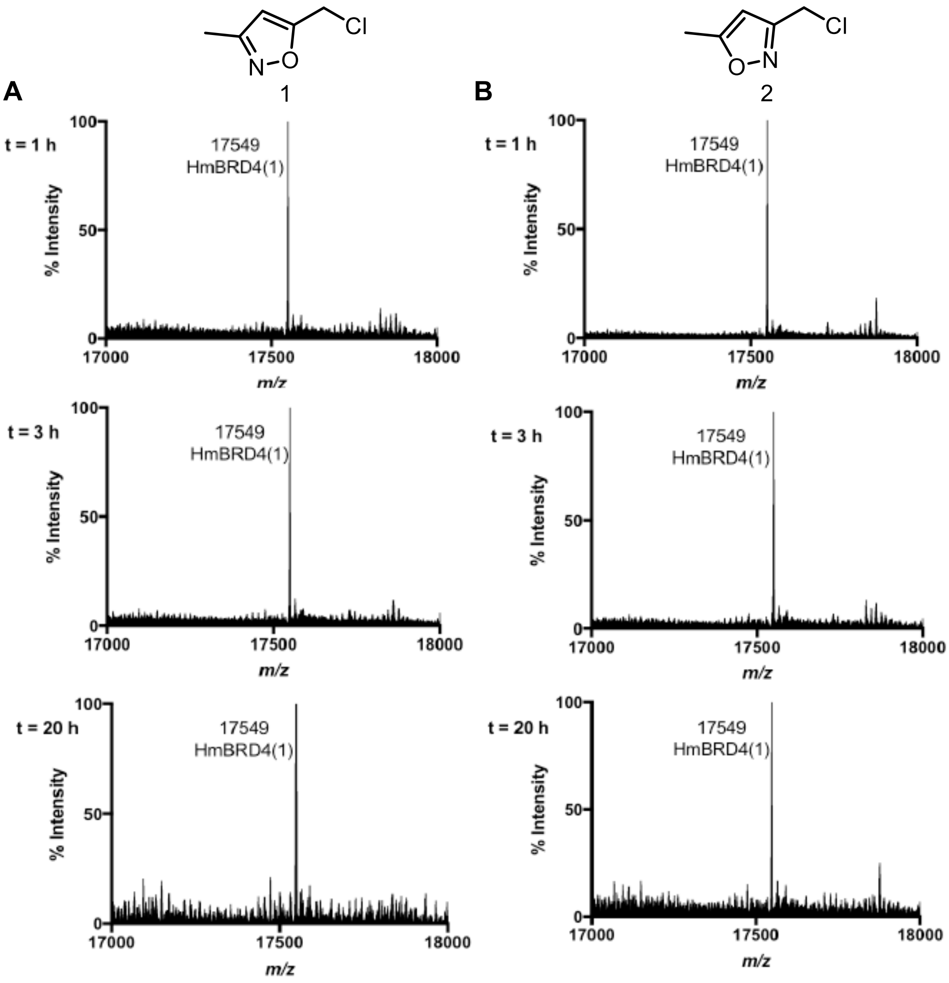

Figure S2. Small reactive isoxazole fragments **1** and **2** displayed no covalent labeling of BRD4(1)^{WT} in a protein LCMS assay, indicating that C125 and C136 were not highly reactive to electrophiles.

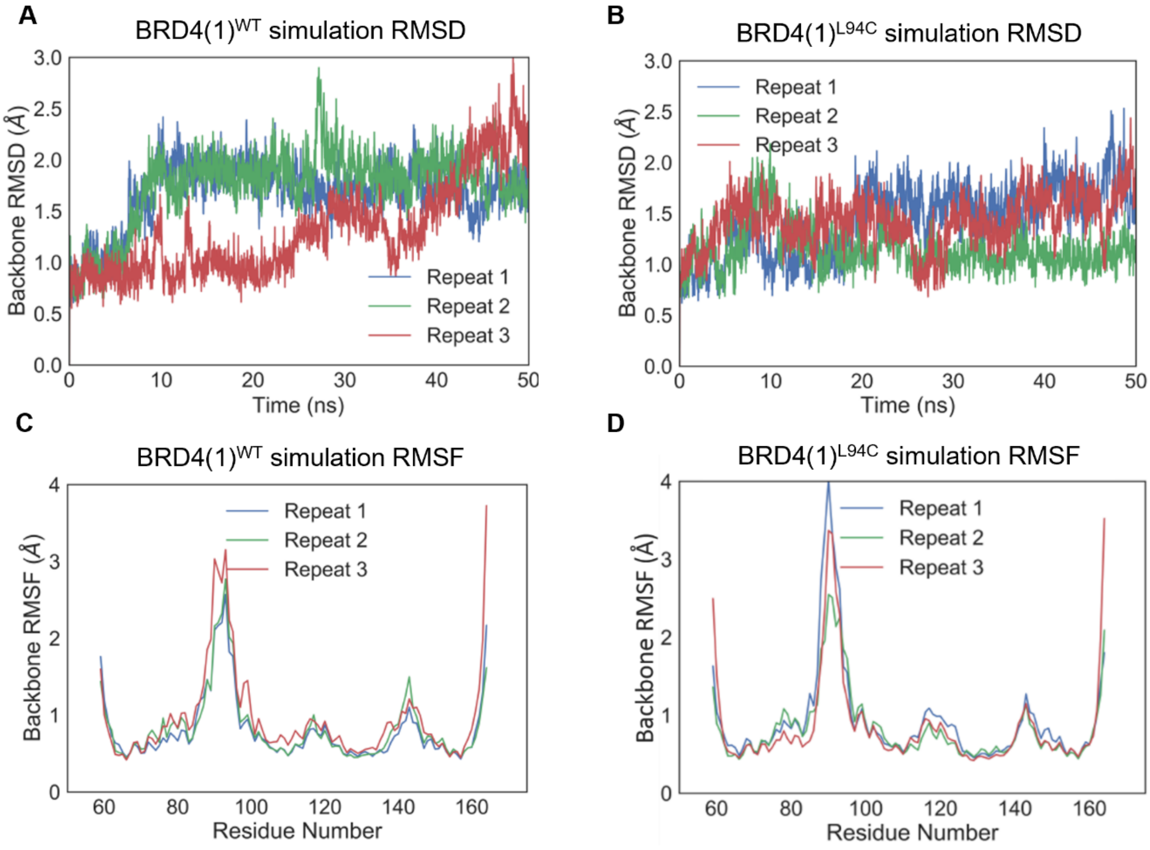

Figure S3. Molecular dynamics simulations were performed over 50 ns to compare the stability of BRD4(1)^{L94C} to BRD4(1)^{WT}. The experiments were carried out in triplicate, with each replica shown in either blue, green or red. **A**) RMSD simulation for BRD4(1)^{WT}. **B**) RMSD simulation for BRD4(1)^{L94C}. **C**) RMSF simulation for BRD4(1)^{WT}. **D**) RMSF simulation for BRD4(1)^{L94C}.

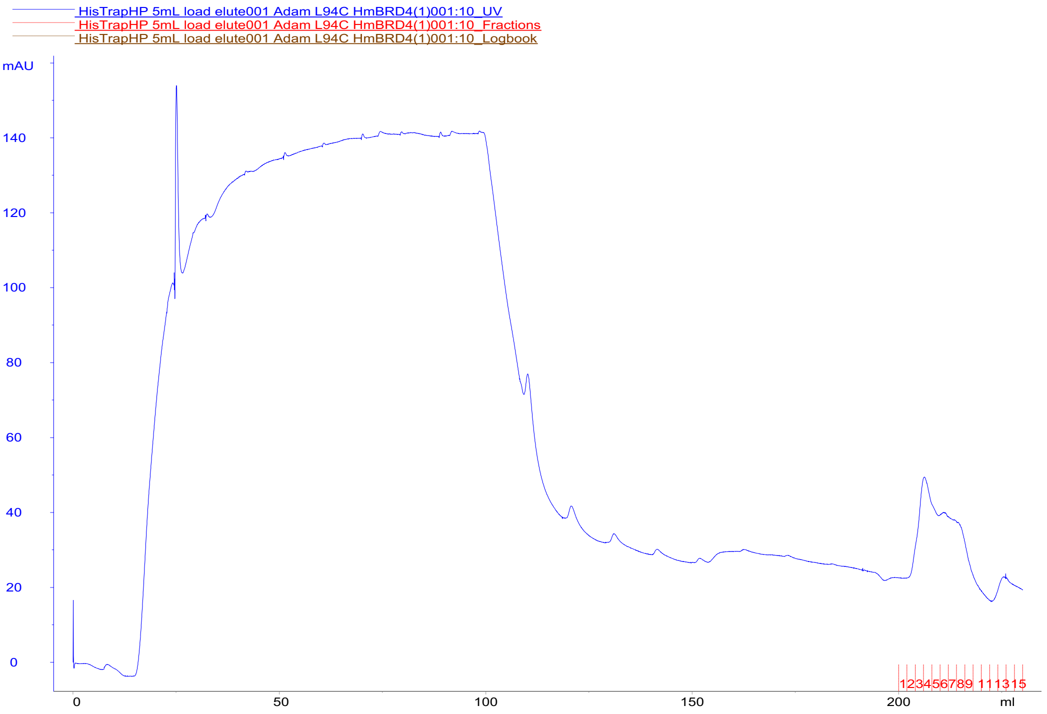

Figure S4. IMAC trace of BRD4(1)^{L94C} purification. Fractions 2–5 combined.

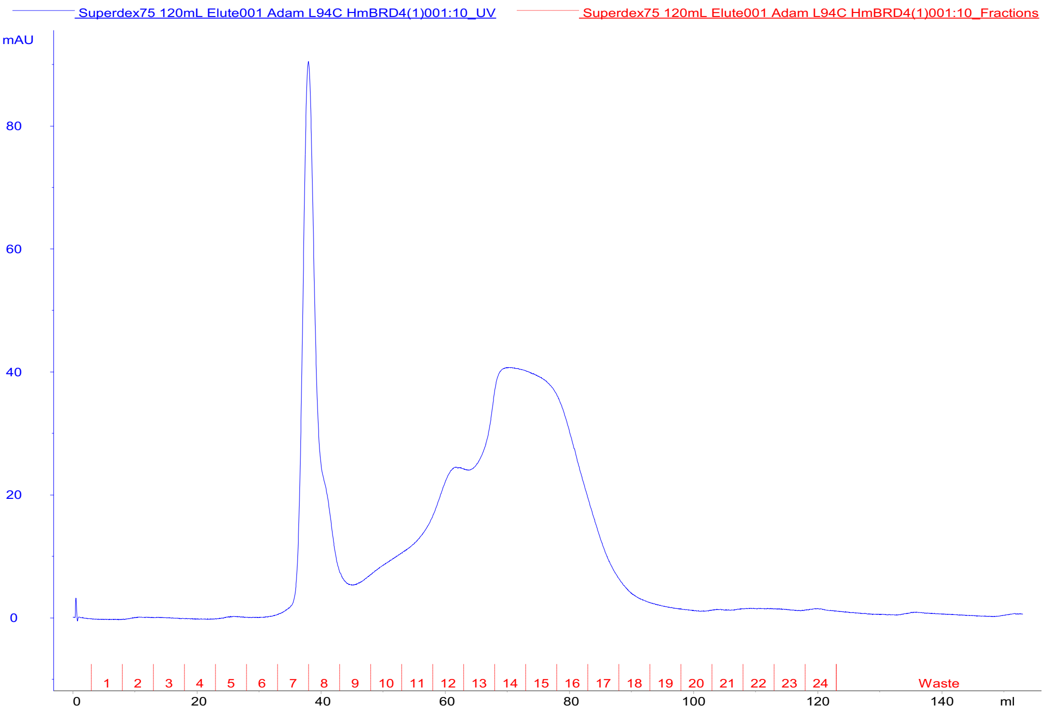

Figure S5. SEC trace of BRD4(1)^{L94C} purification. Fractions 13–16 combined.

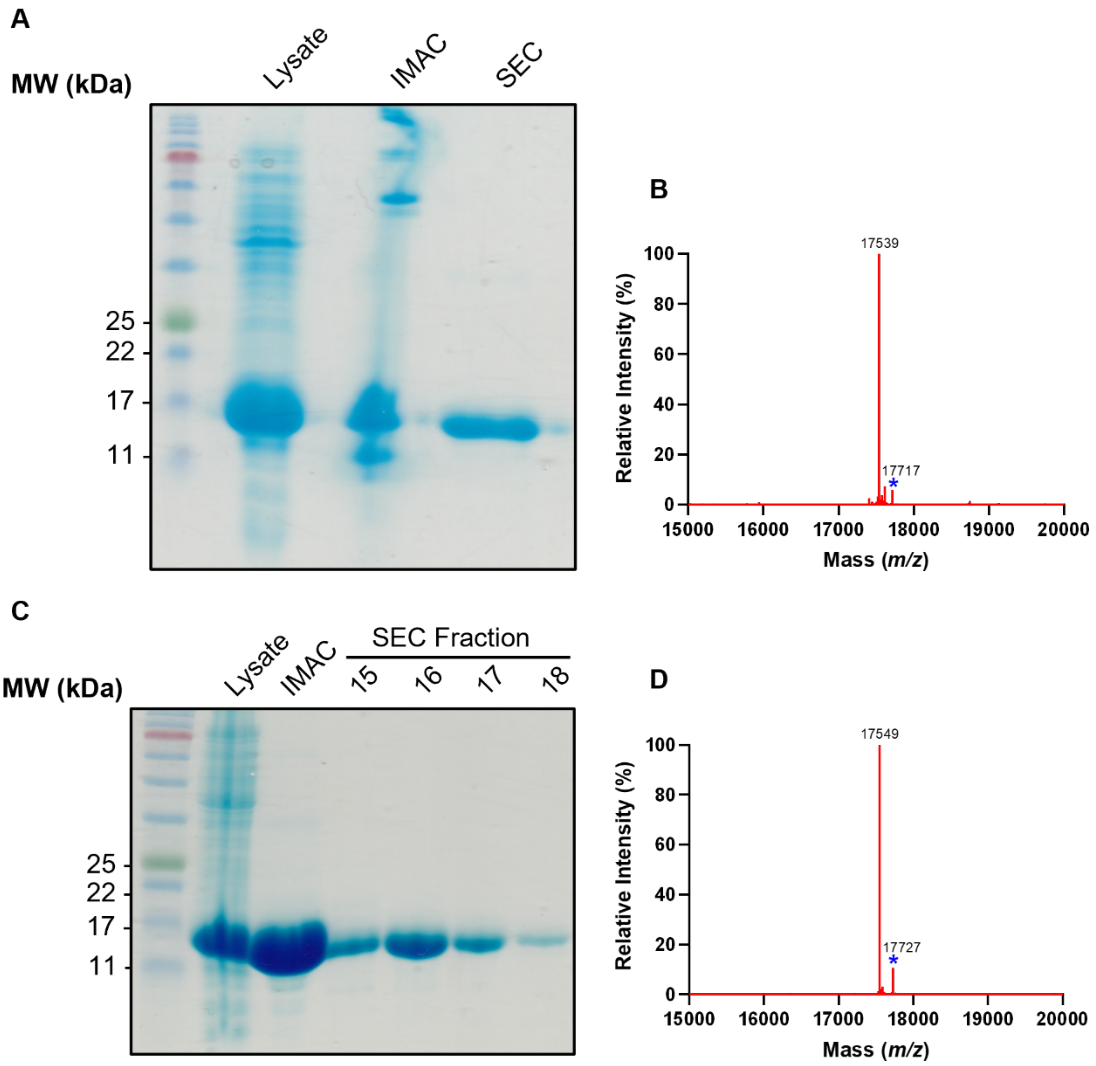

Figure S6. Purification of BRD4(1)^{L94C} and BRD4(1)^{WT}. **A**) SDS-PAGE analysis of the cell lysate, IMAC fractions, and SEC fractions for BRD4(1)^{L94C}. **B**) Protein mass spectrometry of BRD4(1)^{L94C} confirmed the expected mass. The blue asterisk highlights the α-*N*-6-phosphogluconoylation +178 Da adduct of the His_6-tag modification.[1] **C**) SDS-PAGE analysis of the cell lysate, IMAC fractions, and SEC fractions for BRD4(1)^{WT}. **D**) Protein mass spectrometry of BRD4(1)^{WT} confirmed the expected mass. The blue asterisk highlights the +178 Da adduct for the α-*N*-6-phosphogluconoylation of the His_6-tag.[1]

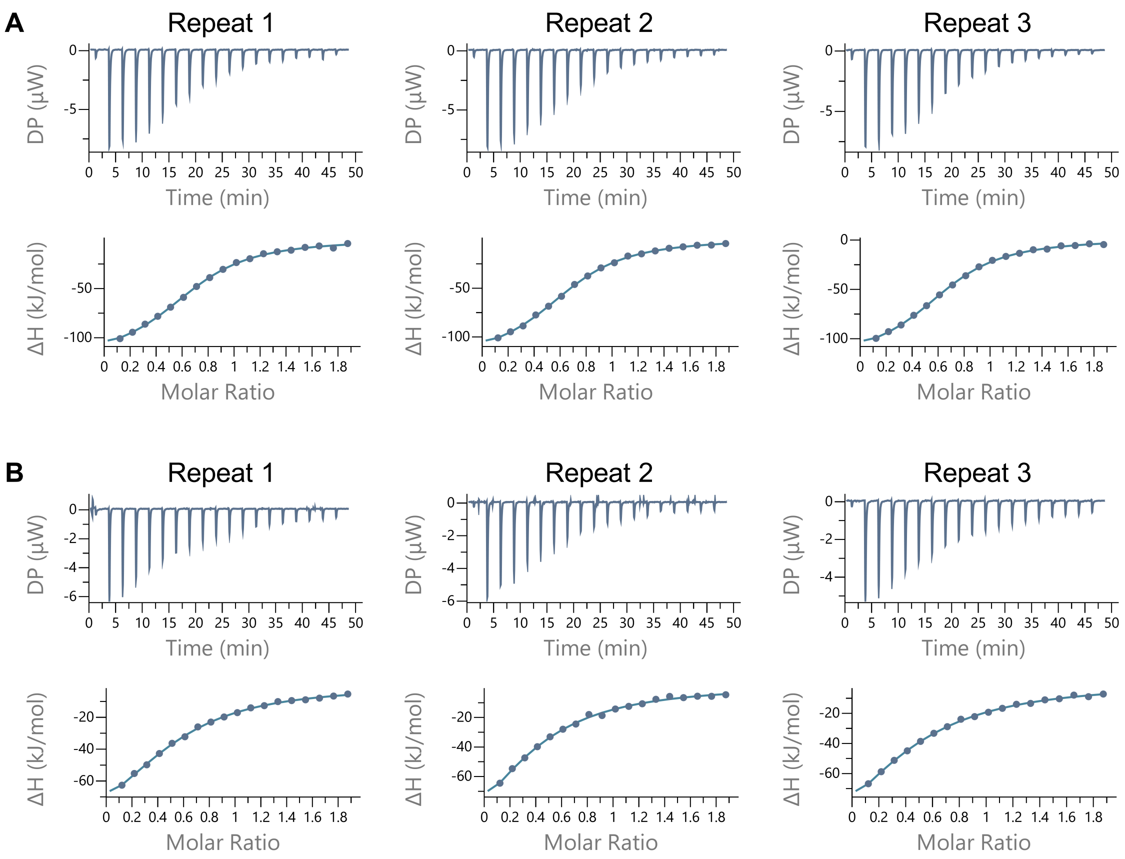

Figure S7. ITC traces for the tetra-acetylated H4 [H4$_{1\text{–}20}$(KAc)$_4$] peptide binding to BRD4(1)^{WT} (**A**) or BRD4(1)^{L94C} (**B**).

Table S1. ITC data for the tetra-acetylated H4 [H4$_{1\text{–}20}$(KAc)$_4$] peptide binding to BRD4(1)^{WT} or BRD4(1)^{L94C}.

		Repeat		
		1	2	3
BRD4(1)^{WT}	N	0.662	0.653	0.644
	K_d (μM)	6.07 ± 0.53	5.95 ± 0.35	5.73 ± 0.33
	ΔH (kJ/mol)	-122	-124	-122
	ΔG (kJ/mol)	-29.8	-29.9	-29.9
	-TΔS (kJ/mol)	92.5	94.3	92.1
BRD4(1)^{L94C}	N	0.517	0.389	0.452
	K_d (μM)	16.3 ± 2.04	20.9 ± 5.42	23.4 ± 3.63
	ΔH (kJ/mol)	-109	-150	-147
	ΔG (kJ/mol)	-27.3	-26.7	-26.5
	-TΔS (kJ/mol)	81.2	124	121

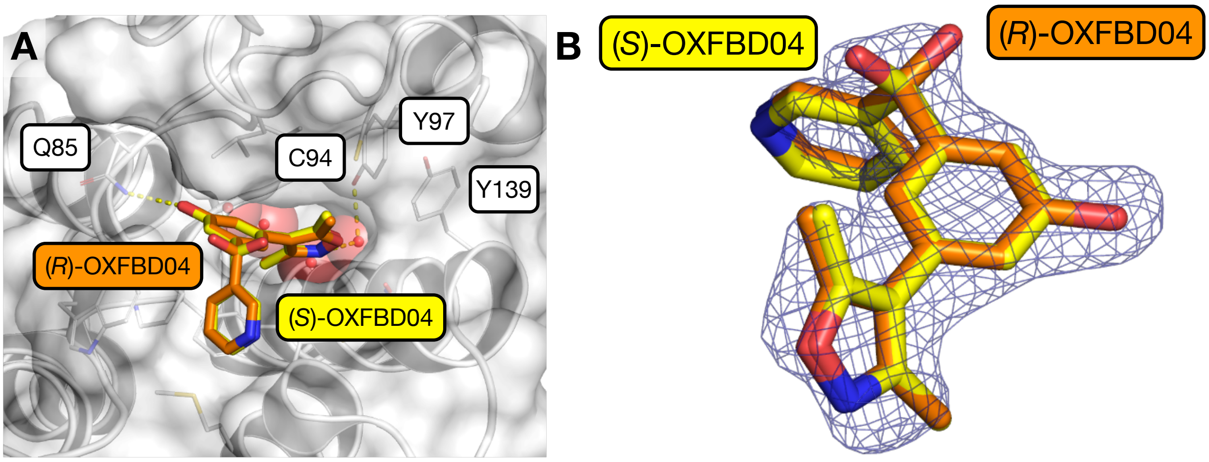

Figure S8. A) A view from the crystal structure of BRD4(1)^{L94C} (white cartoon and transparent surface, binding site residues shown as sticks) with OXFBD04 (**3**) (*R*-enantiomer orange sticks, *S*-enantiomer yellow sticks) in the binding site. Key interactions shown as yellow dashes. Important binding site water molecules shown as red spheres and key binding site residues discussed in the text are labeled. **B**) OMIT mFo-DFc electron density for OXFBD04 (**3**) contoured to 2σ clearly showing the presence of both enantiomers. The best agreement of the refined model with resulting difference density maps occurred when each enantiomer was included with 50% occupancy.

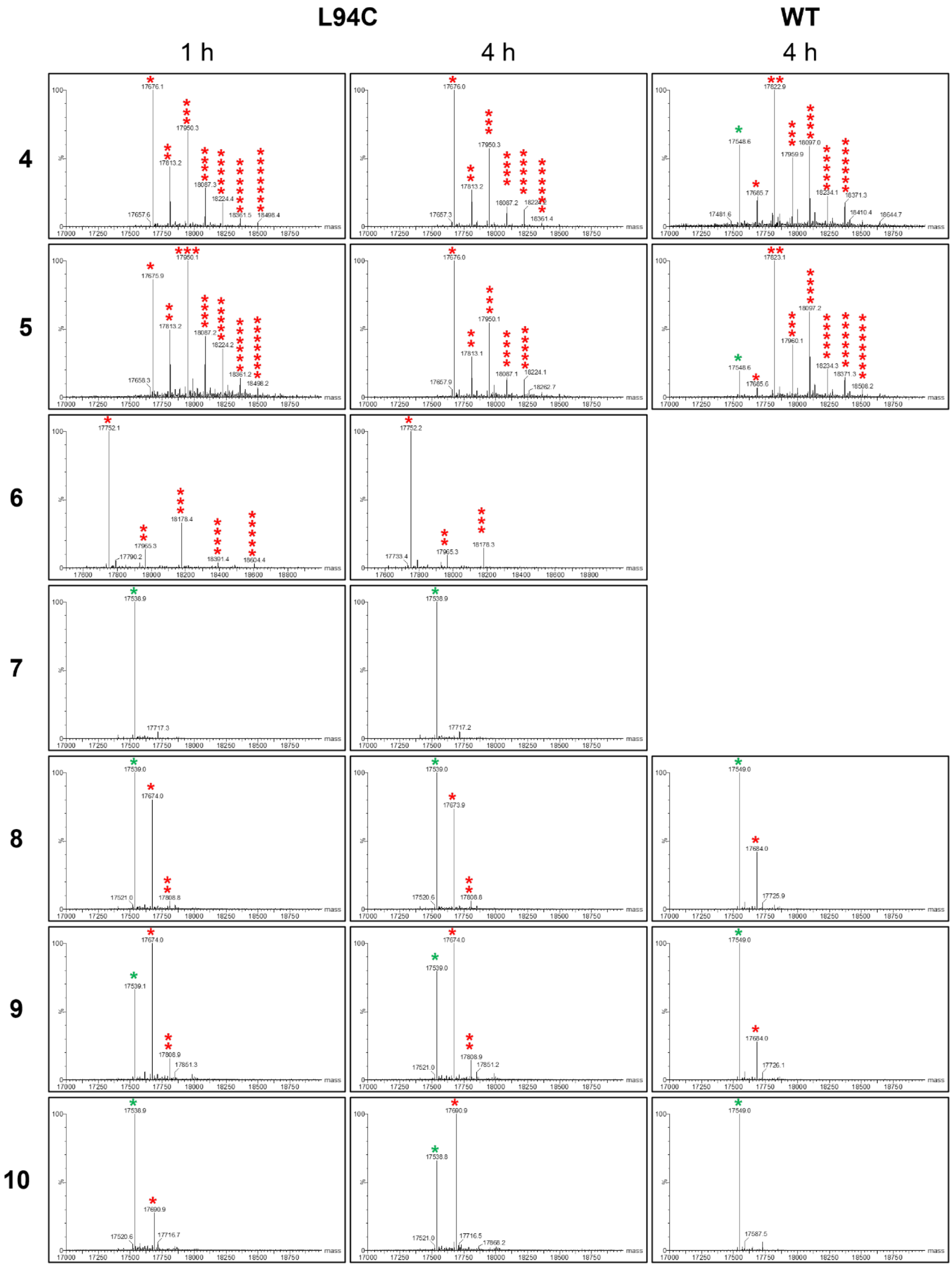
L94C
WT
1 h
4 h
4 h
4
5
6
7
8
9
10

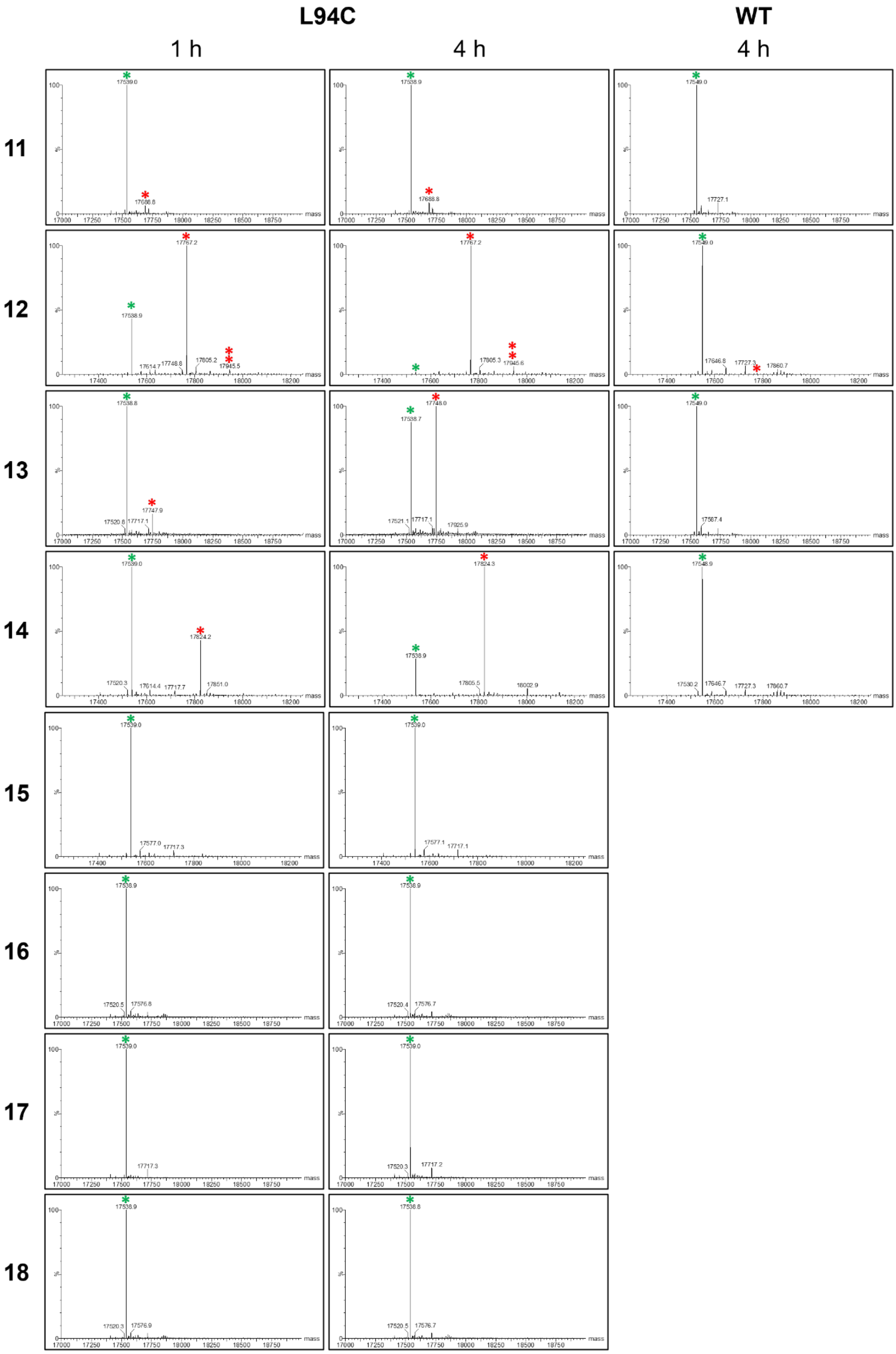
L94C
WT
1 h
4 h
4 h
11
12
13
14
15
16
17
18

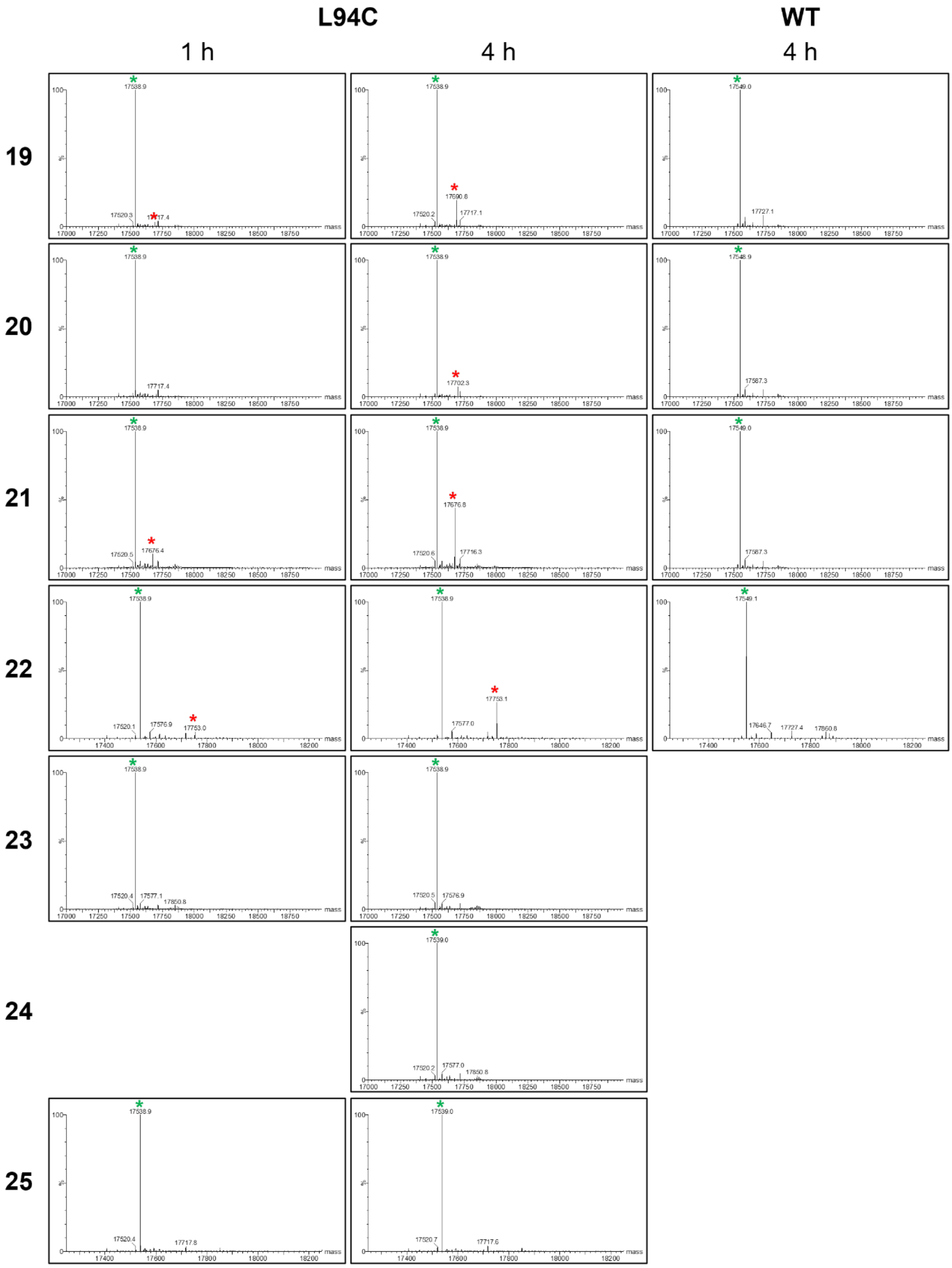

Figure S9. Deconvoluted protein mass spectra for the structure-guided fragments assessed (compound numbers shown on the left) in ‘labeling assay 1’. The Y-axis shows relative intensity (%). The green asterisk shows peaks corresponding to unlabeled protein (*), and the red asterisks show peaks corresponding to labeled protein (*) with the number of asterisks representing the number of labeling events.

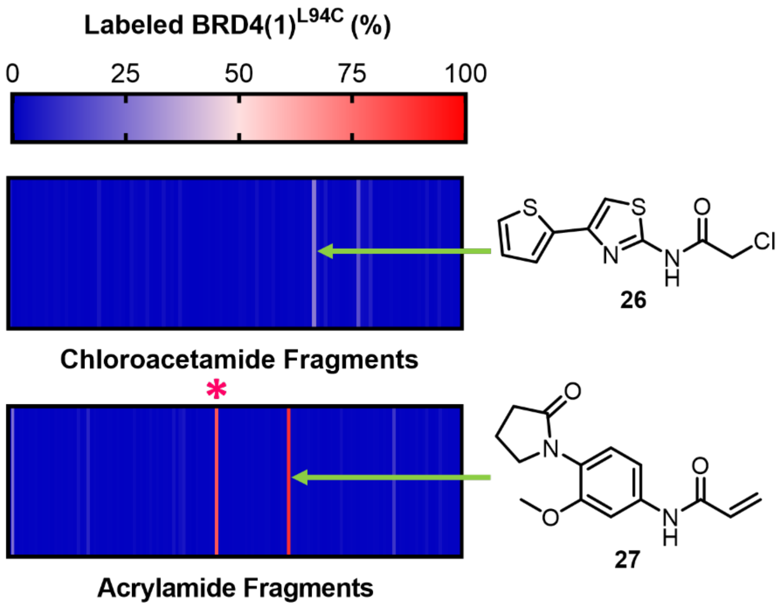

Figure S10. Heatmaps showing percentage labeling data obtained from the diverse reactive fragment screening against BRD4(1)^{L94C}, with 'hit' compound structures shown. The pink asterisks show fragments with significant labeling, but due to mass discrepancies did not fulfil the 'hit' criteria.

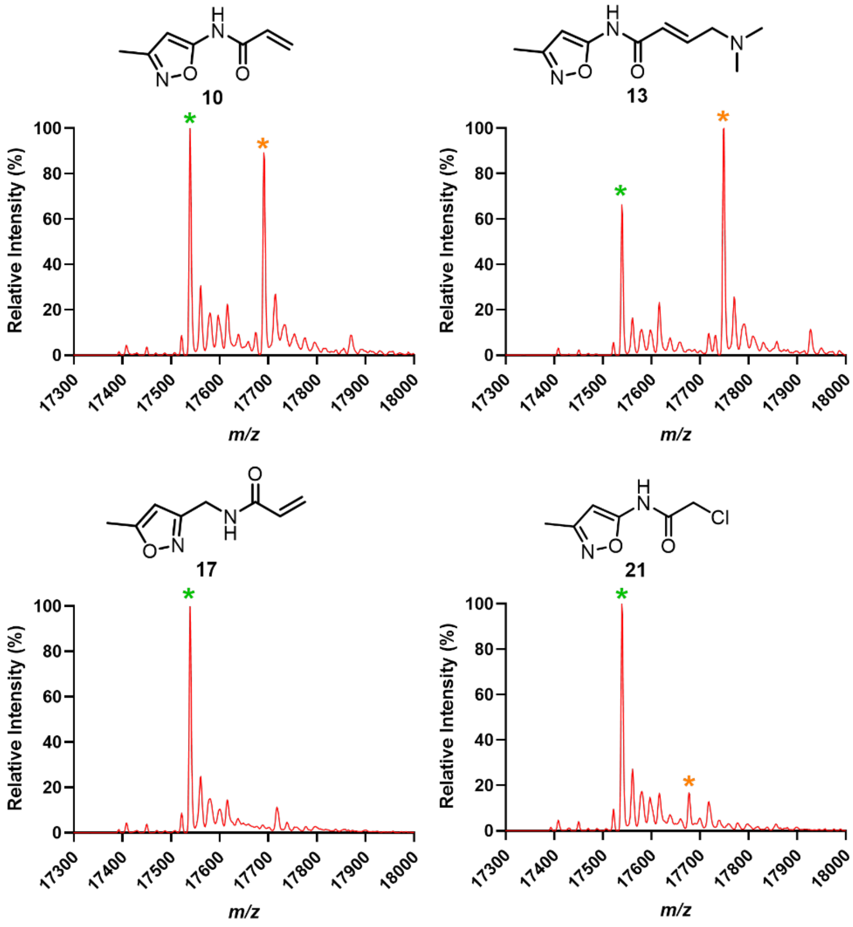

Figure S11. Four isoxazole fragments (**10**, **13**, **17**, and **21**), identified from the structure-guided reactive fragment screen, were used as positive and negative controls for the 'labeling assay 2' screening conditions.

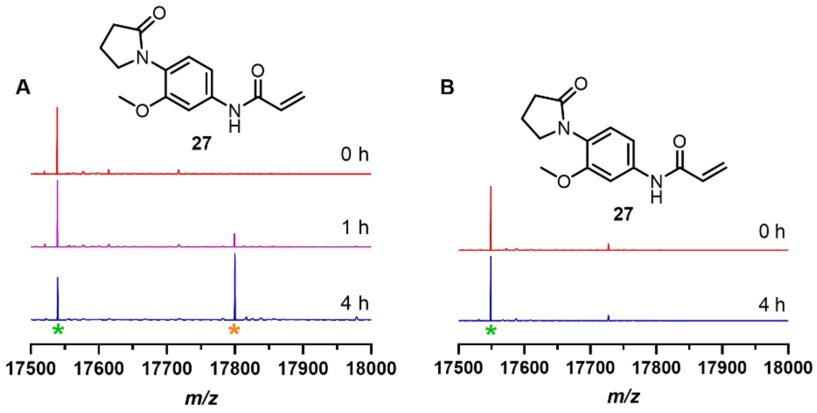

Figure S12. Time course of protein LCMS spectra for BRD4(1)^{L94C} (**A**) and BRD4(1)^{WT} (**B**) labeling by **27** using 'labeling assay 1' conditions. The Y-axis shows relative intensity (%). The green asterisk shows peaks corresponding to unlabeled protein, and the orange asterisks show peaks corresponding to labeled protein.

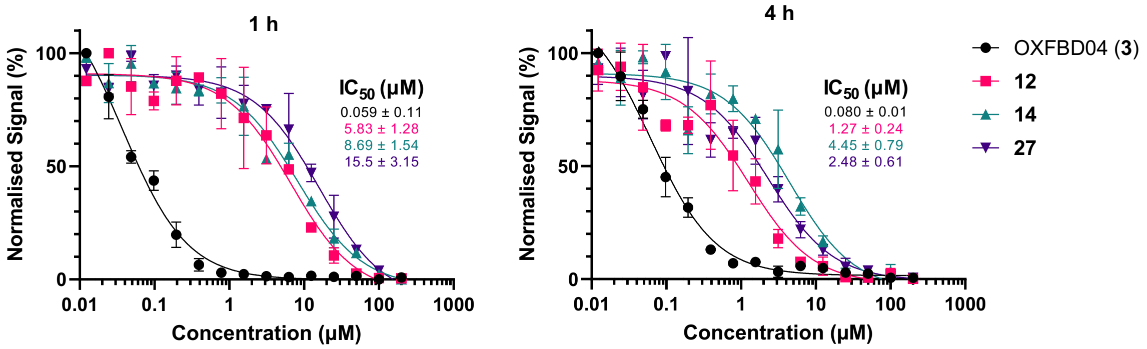

Figure S13. AlphaScreen™ data for OXFBD04 (**3**), **12**, **14**, and **27** against BRD4(1)^{L94C} at 1 and 4 h. The covalent fragments **12**, **14**, and **27** exhibit increasing inhibition of H4$_{1\text{–}20}$(KAc)$_4$ peptide binding in a time dependent manner, characteristic of covalent ligands, whereas the non-covalent control ligand OXFBD04 (**3**) does not. IC_{50} values for each time point are quoted as the mean value from a triplicate experiment ± s.e.m.

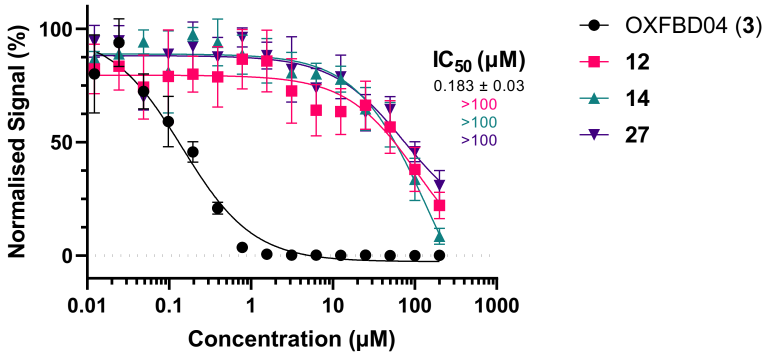

Figure S14. AlphaScreen™ data for OXFBD04 (**3**), **12**, **14**, and **27** against BRD4(1)^{WT} at 4 h. Compounds **12**, **14**, and **27** show no substantial binding to BRD4(1)^{WT}. IC_{50} values are quoted as the mean value from a triplicate experiment ± s.e.m.

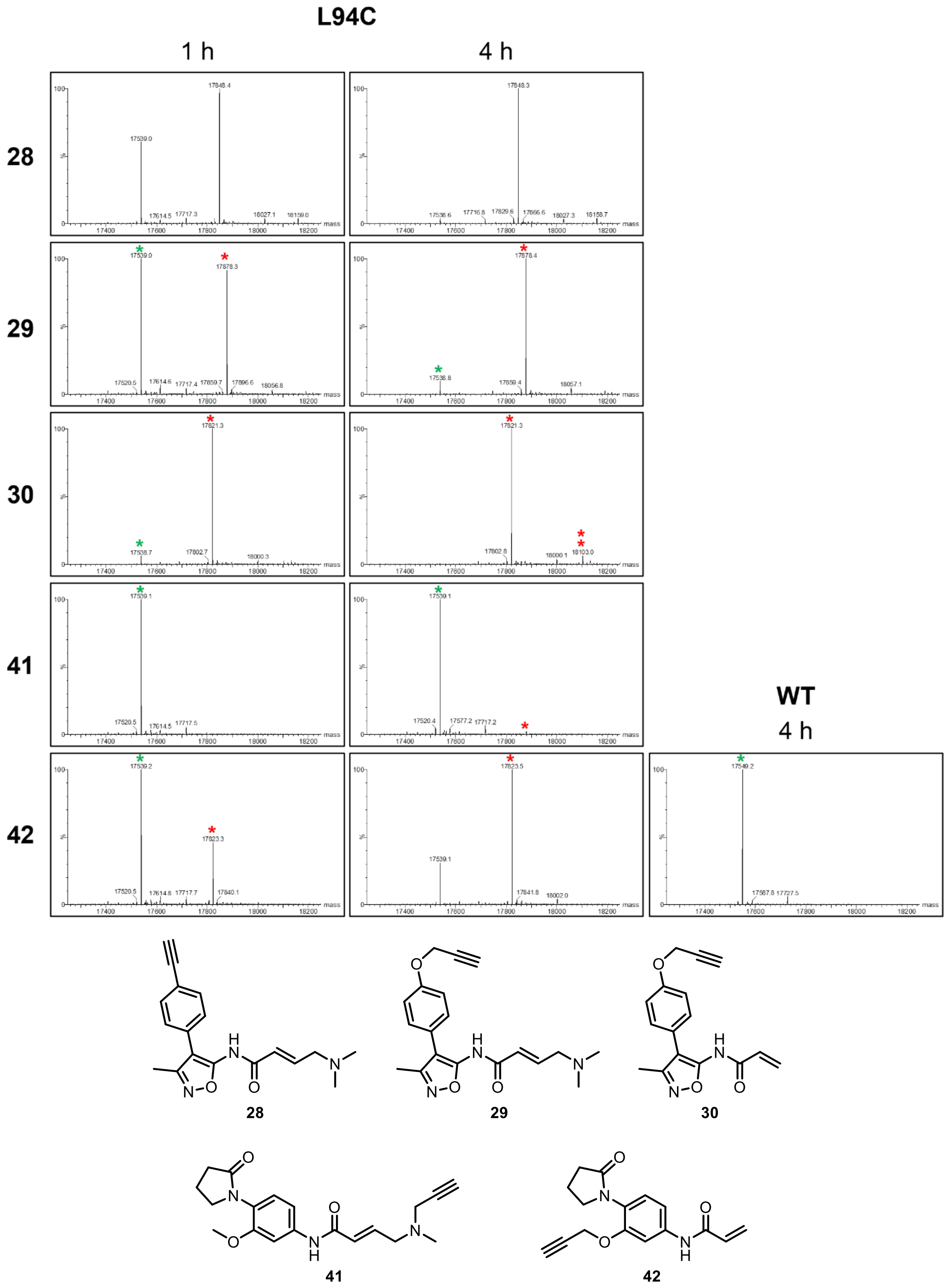

Figure S15. Deconvoluted protein mass spectra for the structure-guided fragments assessed (compound numbers shown on the left) in 'labeling assay 1'. The Y-axis shows relative intensity (%). The green asterisk shows peaks corresponding to unlabeled protein (*), and the red asterisks show peaks corresponding to labeled protein (*) with the number of asterisks representing the number of labeling events.

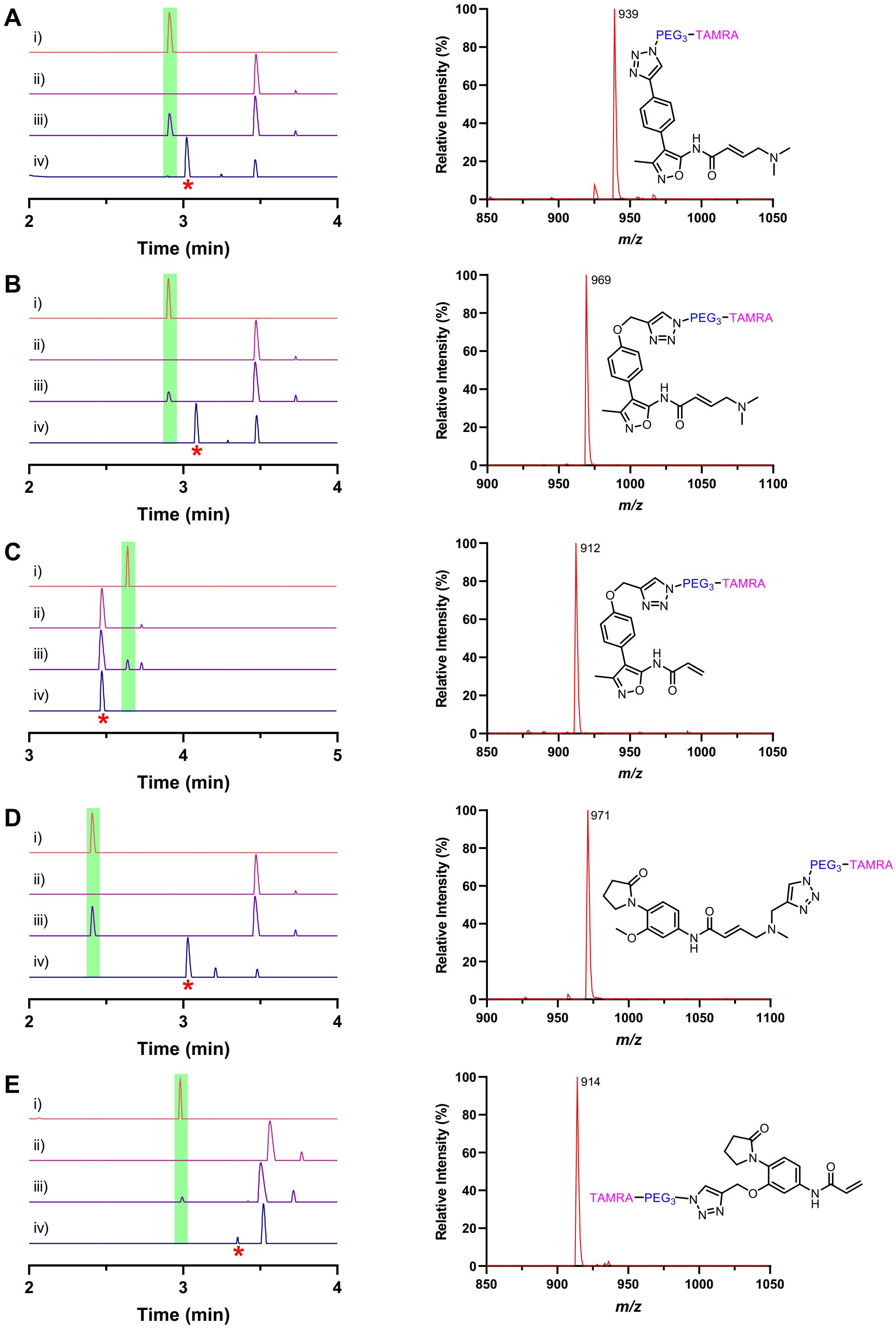

Figure S16. LCMS UV chromatograms (left) and mass spectra (right) for the CuAAC reaction between TAMRA-PEG$_3$-azide and click probes **28** (**A**), **29** (**B**), **30** (**C**), **41** (**D**), **42** (**E**). i) Clickable probe; ii) TAMRA-PEG$_3$-Azide; iii) Clickable probe and TAMRA-PEG$_3$-azide, no catalyst; iv) CuAAC reaction, 1 h, rt. The pale green box highlights the UV peak corresponding to the clickable probe. The red asterisk shows the peak corresponding to the triazole-linked clickable probe-TAMRA-PEG$_3$ conjugate.

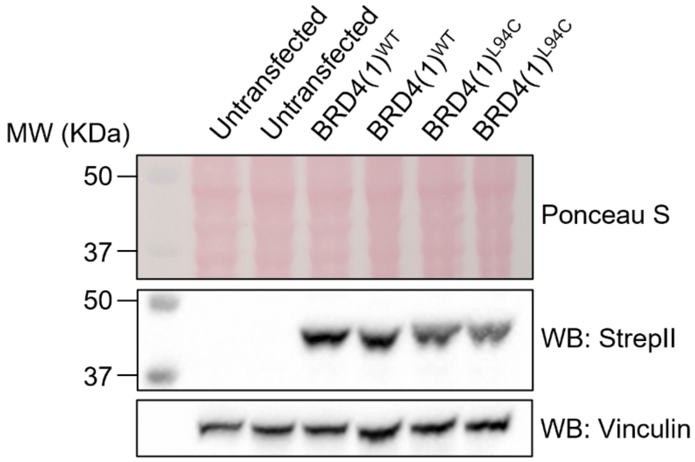

Figure S17. Validation of transfection and protein expression. Untransfected HEK293T cells, transfected HEK293T cells expressing BRD4(1)^{WT}, and transfected HEK293T cells expressing BRD4(1)^{L94C} were analyzed using western blotting (WB) to evaluate protein expression of the StrepII-tagged BRD4(1) proteins. Vinculin was used as a loading control (n=2).

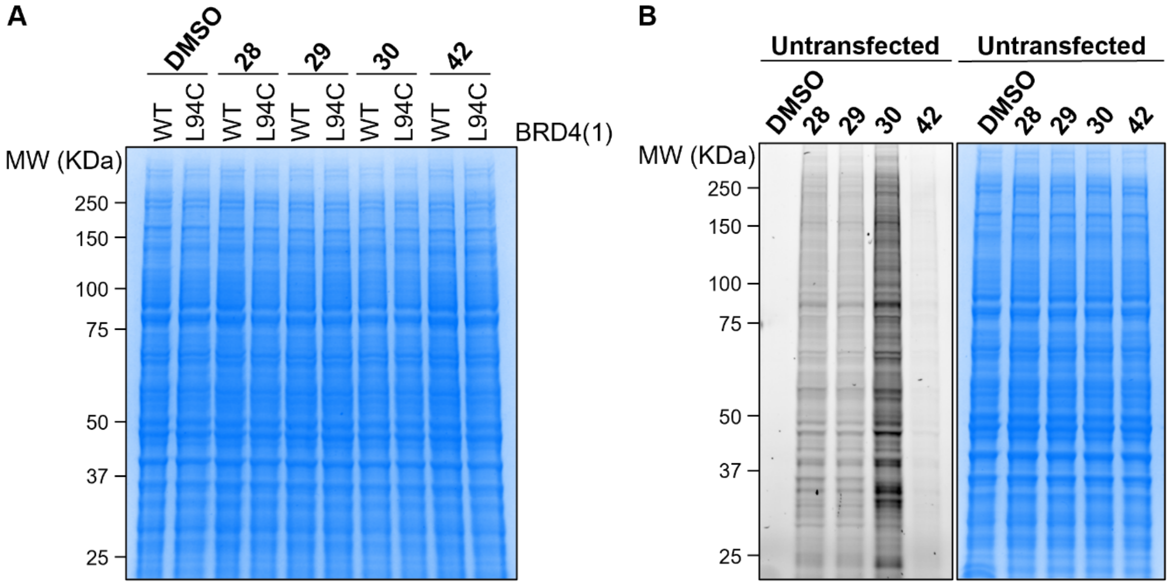

Figure S18. In-cell labeling by clickable probes **28**, **29**, **30**, and **42**. **A**) The coomassie stain of the gel shown in Figure 7B. **B**) In-cell labeling of untransfected HEK293T cells.

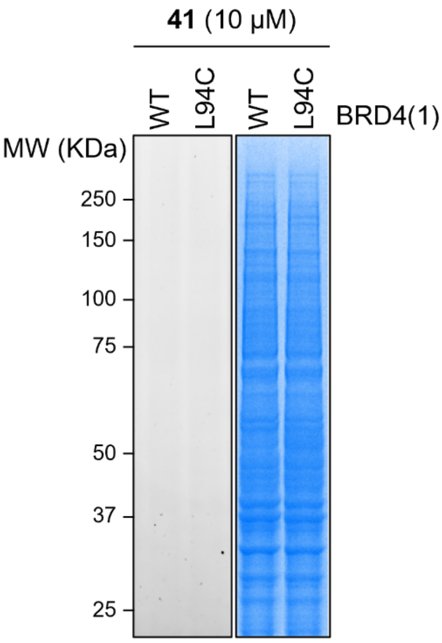

Figure S19. In-cell labeling of BRD4(1)^{WT} or BRD4(1)^{L94C} by clickable probe **41** in HEK293T cells transiently expressing the protein shown.

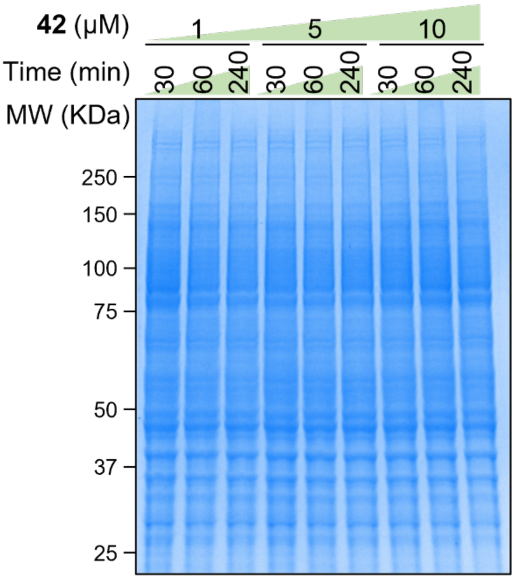

Figure S20. Clickable probe **42** covalently engages with BRD4(1)^{L94C} in a time and dose dependent manner, typical of covalent inhibitors. The coomassie stain of the gel shown in Figure 7C.

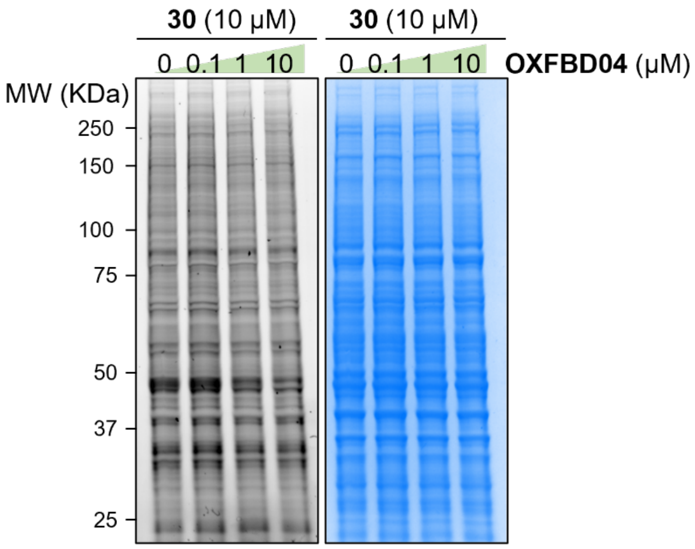

Figure S21. BRD4(1)^{L94C} engagement by **30** was validated using a competition assay with the BRD4(1)^{L94C} ligand, OXFBD04 (**3**). Full fluorescence and coomassie gel of Figure 7D.

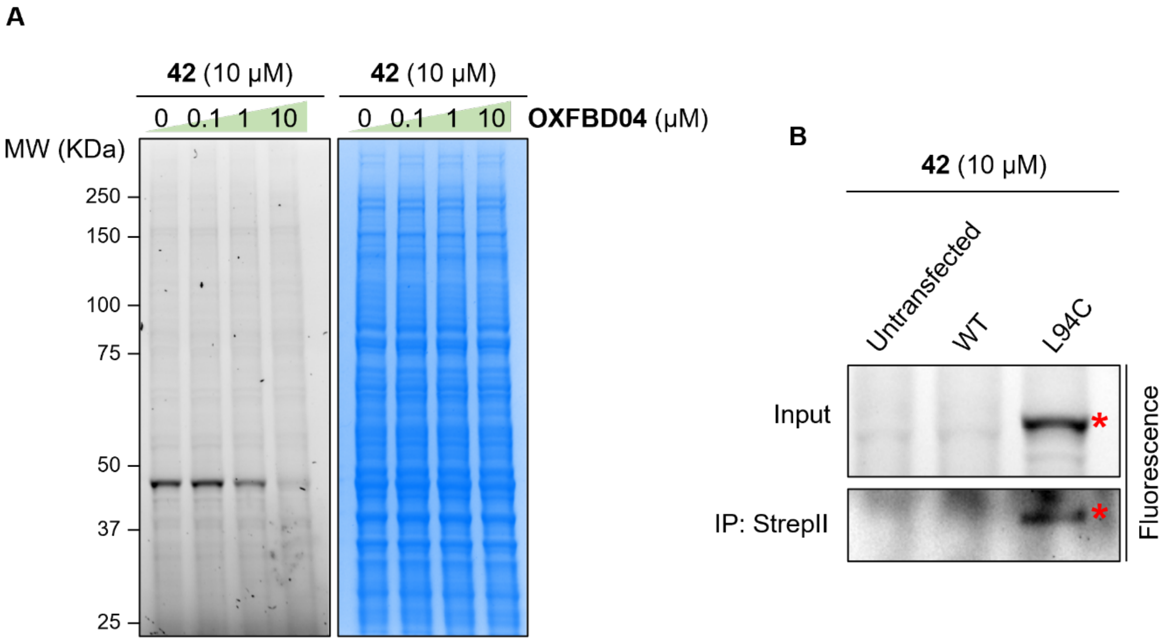

Figure S22. BRD4(1)^{L94C} engagement by **42** was validated using a competition assay with the known BRD4(1)^{L94C} ligand, OXFBD04 (**3**), and immunoprecipitation (IP). **A**) Full fluorescence and coomassie gel of Figure 7E. **B**) IP of StrepII-tagged proteins confirms labeling of BRD4(1)^{L94C}.

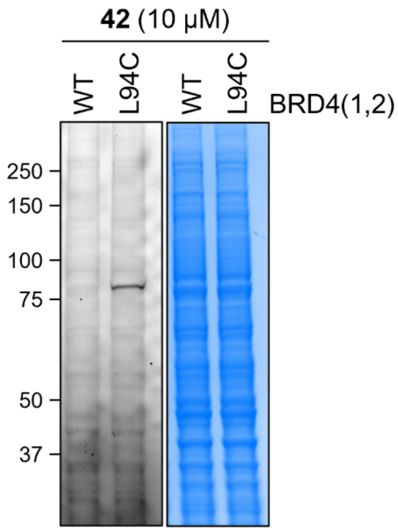

Figure S23. Clickable probe **42** selectively and covalently binds to the first bromodomain of BRD4(1,2)^{L94C}. Full fluorescence and coomassie gel of Figure 7F.

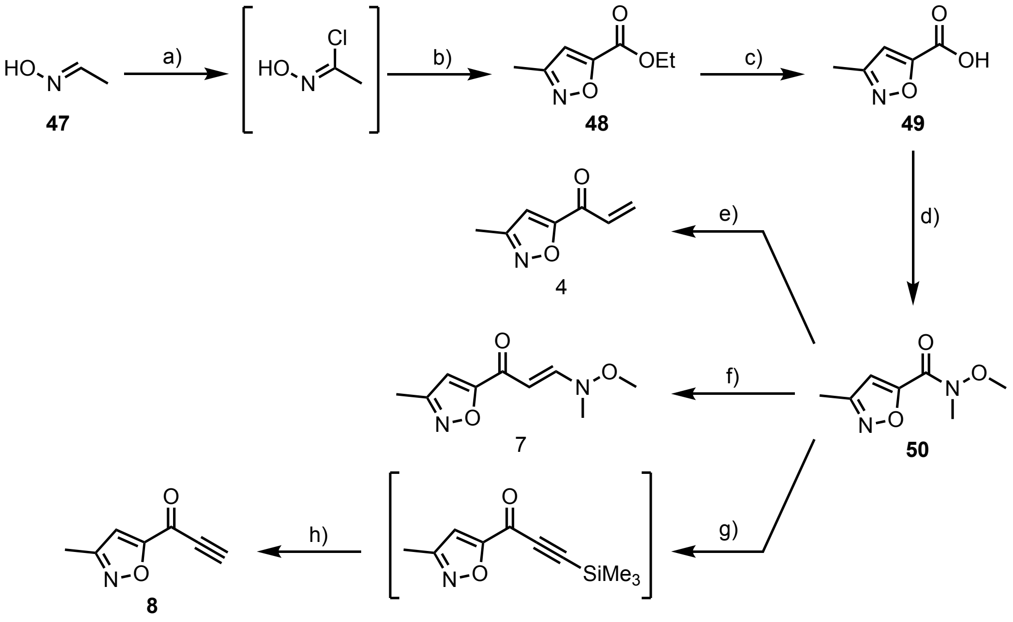

Scheme S1. Synthesis of **4**, **7**, and **8**. *Reagents and conditions*: a) *N*-Chlorosuccinimide, DMF, 0 °C to rt, 3 h; b) Ethyl propiolate, $KHCO_3$, CH_2Cl_2, H_2O, 16 h, 23–43%, n=2; c) LiOH, THF, H_2O (2:1), rt, 16 h, 55–100%, n=3; d) *N,O*-Dimethylhydroxylamine hydrochloride, PyBOP, Et_3N, CH_2Cl_2, 0 °C to rt, 16 h, 44–81%, n=4; e) Vinyl magnesium bromide, THF, −35 °C to 0 °C, 2 h, 56%, n=1; f) Ethynyl magnesium bromide, THF, −40 °C to rt, 2 h, 55%, n=1; g) i) Ethynyltrimethylsilane, isopropyl magnesium bromide, THF, −40 °C to 0 °C, 1.5 h; ii) **50**, THF, −40 °C to 0 °C, 1.5 h; h) Silica gel, 27% over two steps, n=1.

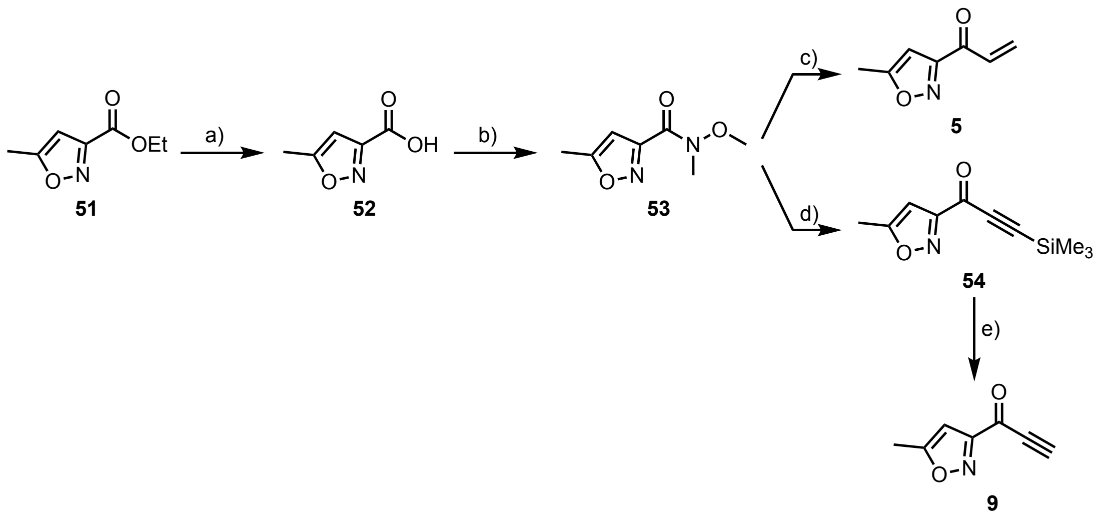

Scheme S2. Synthesis of **5** and **9**. *Reagents and conditions*: a) LiOH, THF:H_2O (2:1), rt, 16 h, 92–97%, n=3; *N,O*-Dimethylhydroxylamine hydrochloride, PyBOP, Et_3N, CH_2Cl_2, 0 °C to rt, 16 h, 62–82%, n=4; c) Vinyl magnesium bromide, THF, −35 °C to 0 °C, 2 h, 38–89%, n=2; d) i) Ethynyltrimethylsilane, isopropyl magnesium bromide, THF, −40 °C to 0 °C, 1.5 h; ii) **53**, THF, −40 °C to 0 °C, 1.5 h, 50–68%, n=2; e) K_2CO_3, MeOH, CH_2Cl_2, 0 °C, 20 min, 46%, n=1.

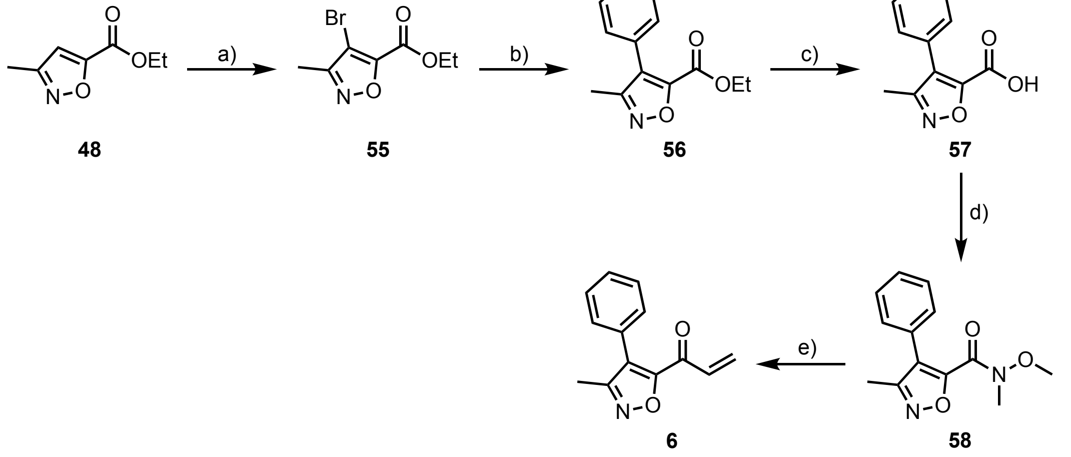

Scheme S3. Synthesis of **6**. *Reagents and conditions*: a) NBS, TFA, 150 °C, microwave, 50 min, 65%, n=1; b) Phenylboronic acid, Pd(dppf)Cl_2·CH_2Cl_2, $NaHCO_3$, DME, H_2O (4:1), 90 °C, 2.5 h, 49%, n=1. c) LiOH, THF, H_2O (2:1), rt, 2 h, 84%, n=1; d) *N*,*O*-Dimethylhydroxylamine hydrochloride, PyBOP, Et_3N, CH_2Cl_2, 0 °C to rt, 16 h, 98%, n=1; e) Vinyl magnesium bromide, THF, −35 °C to 0 °C, 1 h, 62%, n=1.

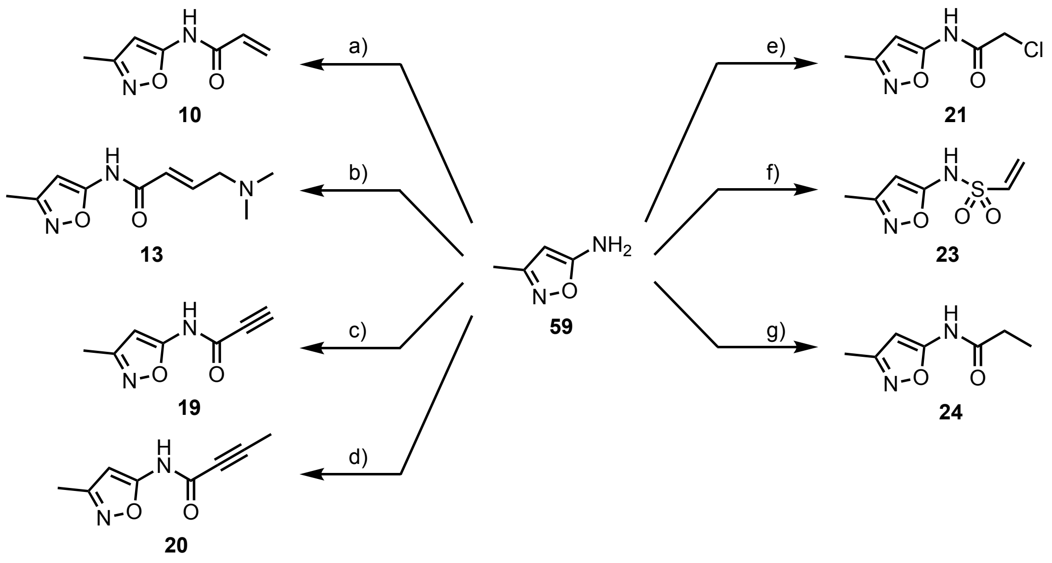

Scheme S4. Synthesis of **10**, **13**, **19**, **20**, **21**, **23**, and **24**. *Reagents and conditions*: a) Acryloyl chloride, Et_3N, CH_2Cl_2, 0 °C to rt, 16 h, 61%, n=1; b) i) (*E*)-4-Dimethylaminobut-2-enoic acid hydrochloride, oxalyl chloride, DMF, THF, 0 °C to rt, 2.5 h; ii) **59**, Et_3N, THF, 0 °C to rt, 2 h, 7%, n=1; c) Propiolic acid, DCC, DMAP, CH_2Cl_2, 0 °C to rt, 5 h, 31%, n=1; d) 2-Butynoic acid, DCC, DMAP, CH_2Cl_2, 0 °C to rt, 4 h, 16%, n=1; e) Chloroacetyl chloride, K_2CO_3, CH_2Cl_2, 0 °C to rt, 16 h, 46%, n=1; f) 2-Chloroethanesulfonyl chloride, Et_3N, CH_2Cl_2, −15 °C to 0 °C, 3 h, 4%, n=1; g) Propionyl chloride, Pyridine, CH_2Cl_2, 0 °C to rt, 2.5 h, 79%, n=1.

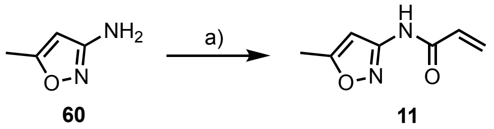

Scheme S5. Synthesis of **11**. *Reagents and conditions*: a) Acryloyl chloride, Et_3N, CH_2Cl_2, 0 °C to rt, 16 h, 72%, n=1.

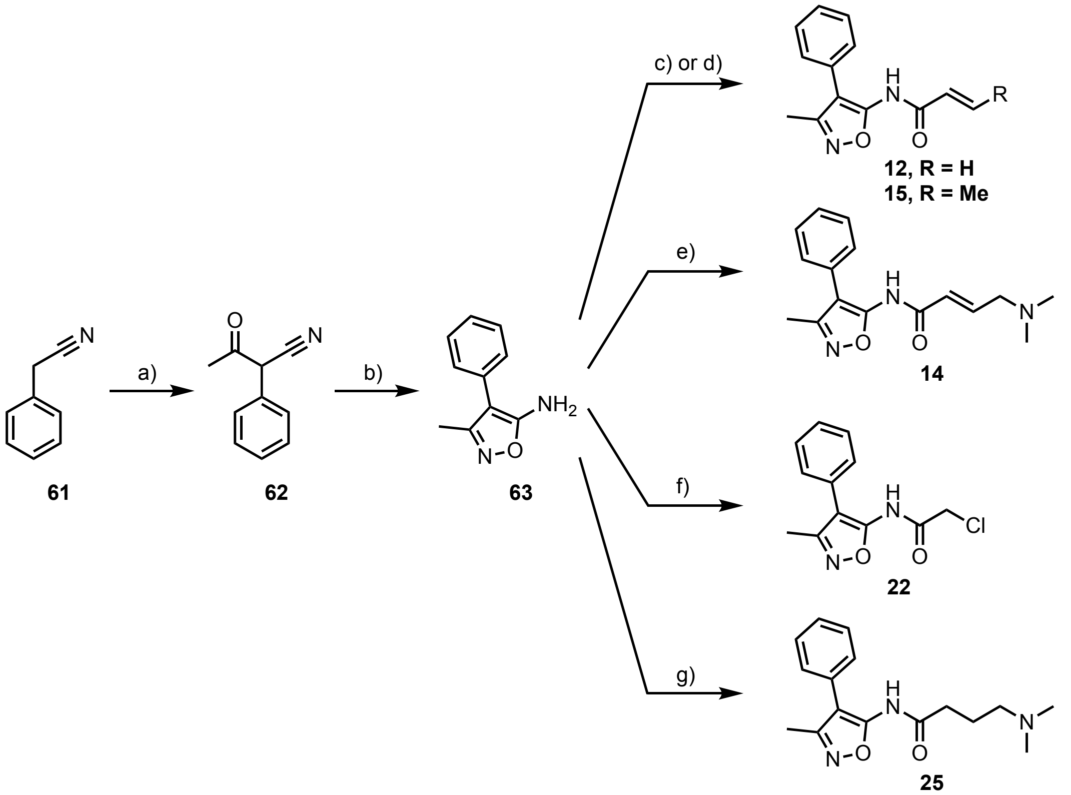

61 62 63 14 22 25

Scheme S6. Synthesis of **12**, **14**, **15**, **22**, and **25**. *Reagents and conditions*: a) EtOAc, NaH, THF, 10% *v/v* DMF, 0 °C to rt, 18 h, 60–69%, n=5; b) Hydroxylamine hydrochloride, 10% aq. Na_2CO_3, EtOH, reflux, 1 h, 81–100%, n=6; c) Acryloyl chloride, pyridine, CH_2Cl_2, 0 °C to rt, 20 h, 10–13%, n=3; d) Crotonoyl chloride, pyridine, CH_2Cl_2, 0 °C to rt, 20 h, 13%, n=1; e) i) (*E*)-4-Dimethylaminobut-2-enoic acid hydrochloride, oxalyl chloride, DMF, THF, 0 °C to rt, 1.5 h; ii) **63**, NMP, 0– 5 °C, 21 h, 33%, n=1; f) Chloroacetyl chloride, pyridine, CH_2Cl_2, 0 °C to rt, 3 h, 24%, n=1; g) 4-(Dimethylamino)butyric acid hydrochloride oxalyl chloride, DMF, THF, 0 °C to rt, 2 h; ii) **63**, NMP, 0–5 °C, 20 h, 60%, n=1.

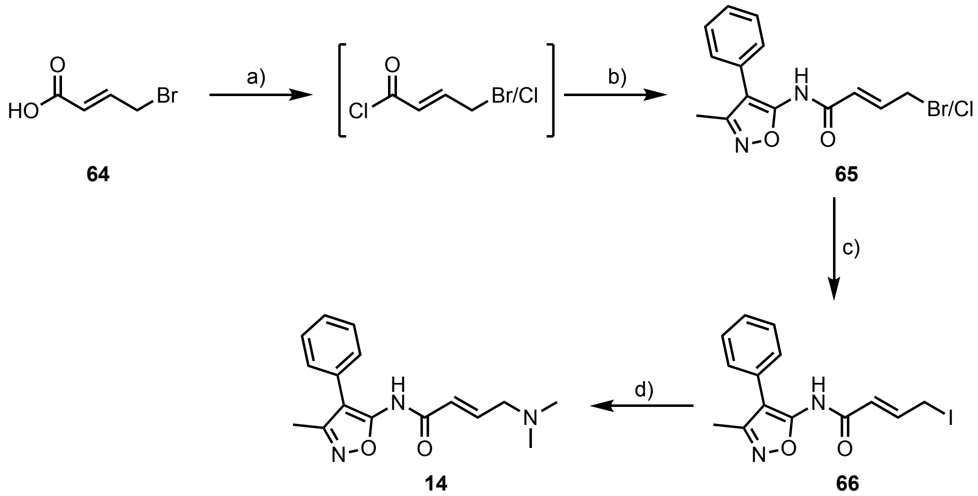

Scheme S7. Alternative synthesis of **14**. *Reagents and conditions*: a) Oxalyl chloride, DMF, THF, 0 °C to rt, 18 h; b) **63**, pyridine, CH_2Cl_2, 0–5 °C , 4 h, 39%, n=1; c) NaI, acetone, 50 °C, 50 min, used directly onto the next step; d) Dimethylamine (2.0 M in THF), K_2CO_3, DMF, 0 °C, 1 h, 31%, n=1.

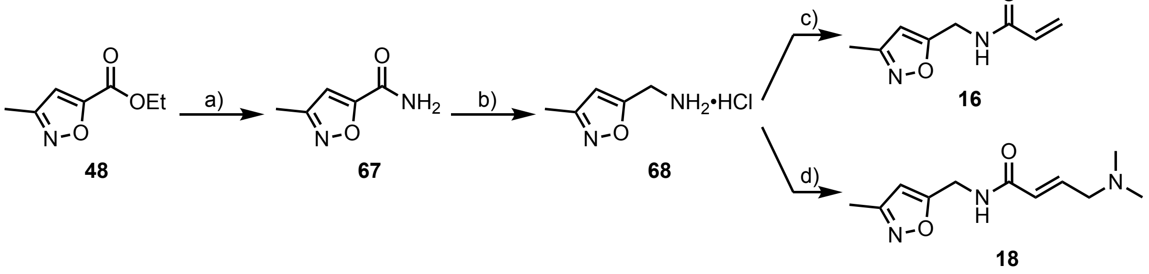

Scheme S8. Synthesis of **16** and **18**. *Reagents and conditions*: a) NH_3, MeOH, rt, 16 h, 38–89%, n=4; b) i) $BH_3{\cdot}SMe_2$ complex, THF, reflux, 6 h, ii) 1.0 M HCl aq, reflux, 1 h, 20–66%, n=2; c) Acryloyl chloride, K_2CO_3, CH_2Cl_2, 0 °C to rt, 3 h, 75%, n=1; d) i) (*E*)-4-Dimethylaminobut-2-enoic acid, oxalyl chloride, DMF, THF, 0 °C to rt, 2.5 h; ii) **68**, Et_3N, THF, 0 °C to rt, 16 h, 35%, n=1.

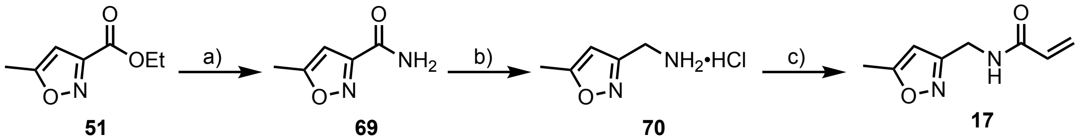

Scheme S9. Synthesis of **17**. *Reagents and conditions*: a) NH_3, MeOH, rt, 16 h, 95%, n=1; b) i) $BH_3{\cdot}SMe_2$ complex, THF, reflux, 6 h, ii) 1.0 M HCl aq, reflux, 1 h, 33%, n=1; c) Acryloyl chloride, Et_3N, CH_2Cl_2, 0 °C to rt, 3 h, 36%, n=1.

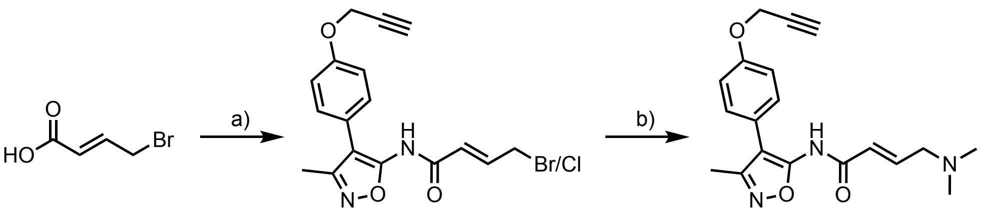

64 **71** **29**

Scheme S10. Alternative synthesis of **29**. *Reagents and conditions*: a) i) Oxalyl chloride, DMF, CH_2Cl_2, 0 °C to rt, 3 h, ii) **39**, pyridine, CH_2Cl_2, 0 °C, 2 h, 36% % (based on a 20% Cl : 80% Br ratio determined using ^{1}H NMR), n=1; b) i) NaI, acetone, 50 °C, 1 h; ii) Dimethylamine (2.0 M in THF), K_2CO_3, DMF, 0 °C, 1 h, 18% over two steps, n=1.

General Chemistry Experimental

Reagents and solvents were obtained from commercial sources (Sigma-Aldrich, Fluorochem, or Alfa Aesar) and were used without further purification, unless otherwise stated. The aqueous solution of NH_4OH used was 28% *w/v*. Compounds **6** and **22** were purchased from Enamine and were used without further purification. Anhydrous solvents were taken from an MBRAUN Solvent Purification System 5, and stored over 3 Å molecular sieves, under an inert atmosphere of Ar. All reactions were carried out in a flame-dried flask, and under an inert atmosphere of Ar, unless otherwise stated. Concentration *in vacuo* refers to solvent removal under reduced pressure at 40 °C using a Buchi™ rotary evaporator, unless otherwise stated. Saturated aqueous solution of NaCl is referred to as brine. Petroleum ether refers to the fractions boiling between 40–60 °C. The purity of all biologically tested compounds was ≥95% as determined by analytical HPLC and/or LCMS.

Analytical thin layer chromatography (TLC) was carried out using Merck silica gel 60 F_{254} aluminium-supported thin layer chromatography sheets. Visualization was by absorption of UV light (λ_{max} 254 nm), or thermal development after staining in either an: aqueous basic solution of potassium permanganate, or ethanolic solution of ninhydrin.

Preparative thin layer chromatography (Prep TLC) was carried out on Macherey-Nagel precoated 60 silica TLC plates. Visualization was by absorption of UV light (λ_{max} 254 nm).

Flash column chromatography was carried out using Merck silica gel (40–63 μm), eluting using solvents as supplied under a positive pressure of N_2 gas.

Semi-preparative high performance liquid chromatography (HPLC) was carried out an Agilent 1260 Infinity II® with an Agilent 5 Prep C18 column (5 μM, 21.2 × 50 mm); 95% H_2O / 5% MeCN + 0.1% formic acid modifier (1 min), 95% H_2O / 5% MeCN to 5% H_2O / 95% MeCN + 0.1% formic acid modifier (10 min), hold (5 min); flow rate 20 mL/min.

Analytical high performance liquid chromatography (HPLC) was carried out on a PerkinElmer Flexar system with a Binary LC Pump and UV/Vis LC detector. For determination of compound purity, a Dionex Acclaim® 120 column (C18, 5 μm, 120 Å, 4.6 × 150 mm) was employed, with a 10 min gradient of 95% H_2O / 5% MeCN + 0.1% TFA (solvent A) to 95% MeCN / 5% H_2O + 0.1% TFA (solvent B), flow rate 1 mL/min. Injected samples were prepared in MeCN and filtered. All samples were run with 0.1% TFA added, unless otherwise stated.

^{1}H NMR spectra were recorded using a Bruker AVII HD 400 (400 MHz), Bruker AVIII HD 500 (500 MHz) or Bruker NEO 600 (600 MHz) with broadband helium cryoprobe spectrometer using the stated solvents as a reference for internal deuterium lock. The chemical shift data for each signal are given as δ_H in units of parts per million (ppm). The spectra are calibrated using the solvent peak with the data provided by Fulmer *et al.*[2] The multiplicity of each signal is indicated by: s (singlet); br s (broad

singlet); d (doublet); t (triplet); q (quartet); quin (quintet); dd (doublet of doublets); dt (doublet of triplets); m (multiplet) or combinations thereof. The number of protons, n, for a given resonance signal is indicated by nH. Coupling constants (*J*) are expressed in Hz and are recorded to the nearest 0.1 Hz. Identical proton coupling constants (*J*) are averaged in each spectrum and reported to the nearest 0.1 Hz. MestReNova and TopSpin software were used for NMR analysis.

^{13}C NMR spectra were recorded using a Bruker AVIII HD 400 spectrometer (101 MHz), Bruker AVIII HD 500 (126 MHz) with broadband proton decoupling, or Bruker NEO 600 (151 MHz) with broadband helium cryoprobe spectrometer using the stated solvent as a reference for internal deuterium lock. The chemical shift data for each signal are quoted as δ_C in parts per million (ppm). The spectra are calibrated using the solvent peak with the data provided by Fulmer *et al.*[2]

Mass spectra were recorded using either an Agilent 6120 (low resolution) or a Bruker microToF (high resolution) spectrometer using electrospray ionization (ESI). Samples were submitted as solutions in either methanol or acetonitrile. *m/z* Values are given in Daltons (Da).

Melting points were obtained using a Leica Galen III hot stage microscope or a Griffin capillary tube melting apparatus and are uncorrected. The crystallization solvent is given in parentheses.

Infrared (IR) spectra were obtained from neat samples. The spectra were recorded using a Bruker Tensor 27 spectrometer with a diamond ATR module. Absorption maxima are given in wavenumbers (cm^{-1}) and reported as s (strong), m (medium), or w (weak).

Synthetic Procedures and Compound Data

Ethyl 3-methylisoxazole-5-carboxylate (48)[3,4]

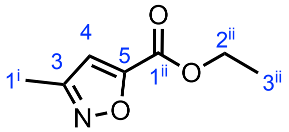

To a solution of acetaldoxime (**47**) (3.00 g, 50.8 mmol, 1.0 eq) in DMF (54 mL), was added *N*-chlorosuccinimide (8.82 g, 60.0 mmol, 1.3 eq) at 0 °C. The solution was slowly warmed to rt, stirred for 3 h, diluted with brine (200 mL) and extracted with Et_2O (5 × 150 mL). The organic components were combined, washed with brine (200 mL), dried over $MgSO_4$, filtered, and concentrated *in vacuo*. The residue was dissolved in CH_2Cl_2 (30 mL) and added to a suspension of ethyl propionate (5.66 mL, 55.9 mmol, 1.1 eq), $KHCO_3$ (13.2 g), H_2O (0.5 mL), CH_2Cl_2 (160 mL) and stirred for 16 h. The reaction was quenched by the addition of H_2O (40 mL) to dissolve the $KHCO_3$. The phases were separated, and the organic components were washed with H_2O (4 × 100 mL), brine (100 mL), dried over $MgSO_4$, filtered, and concentrated *in vacuo*. The crude oil was purified using silica gel column chromatography, eluting with 0–20% Et_2O/petroleum ether, to isolate the title compound as a pale-yellow oil (3.36 g, 43%); R_f 0.26 (10% Et_2O/petroleum ether); ^{1}H NMR (400 MHz, $CDCl_3$) δ_H 6.77 (1H, s, 4-C*H*), 4.40 (2H, q, *J* 7.2, 2^{ii}-CH_2), 2.36 (3H, s, 1^i-CH_3), 1.39 (3H, t, *J* 7.2, 3^{ii}-CH_3); LRMS *m/z* (ESI$^+$) 156 ([M+H]$^+$, 100%), 178 ([M+Na]$^+$, 63%). These data are in good agreement with the literature.[3,4]

3-Methylisoxazole-5-carboxylic acid (49)[5]

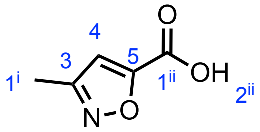

LiOH (541 mg, 12.9 mmol, 2.0 eq) was added to a solution of ethyl 3-methylisoxazole-5-carboxylate (**48**) (1.00 g, 6.45 mmol, 1.0 eq) in THF/H_2O (2:1, 37.5 mL) and the resulting solution was stirred at rt for 16 h. After this time, the THF was removed *in vacuo*, and the remaining solution was acidified to pH 1 with aqueous HCl (6.0 M). The resulting solid was collected by filtration, washed with cold H_2O (35 mL), and dried *in vacuo*, to afford the title compound as colorless solid (534 mg, 66%). R_f 0.13 (10% MeOH/EtOAc); m.p. 170–172 °C (from H_2O) [lit.[5] 170–171 °C]; ^{1}H NMR (400 MHz, D_6-DMSO) δ_H 7.03 (1H, s, 4-C*H*), 2.29 (3H, s, 1^i-CH_3); LRMS *m/z* (ESI$^-$) 126 ([M-H]$^-$, 100%). These data are in good agreement with the literature.[5]

N-Methoxy-N-3-dimethylisoxazole-5-carboxamide (50)

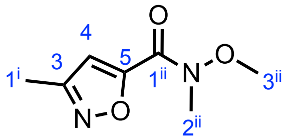

N,O-Dimethylhydroxylamine hydrochloride (299 mg, 3.07 mmol, 1.3 eq), and anhydrous triethylamine (981 µL, 7.08 mmol, 3.0 eq) were added to solution of 3-methylisoxazole-5-carboxylic acid (**49**) (300 mg, 2.36 mmol, 1.0 eq) in anhydrous CH_2Cl_2 (10 mL). In a separate flask, PyBOP (2.46 g, 4.72 mmol, 2.0 eq) was dissolved in anhydrous CH_2Cl_2 (10 mL), this solution was then added dropwise to the reaction mixture at 0 °C. The solution was slowly warmed to rt and stirred for 16 h. The reaction mixture was extracted with CH_2Cl_2 (20 mL) and washed with H_2O (2 × 30 mL), saturated aqueous $NaHCO_3$ solution (30 mL), and brine (30 mL), dried over Na_2SO_4, filtered, and concentrated *in vacuo*. The crude product was purified using silica gel column chromatography, eluting with 40–60% EtOAc/petroleum ether, to isolate the title compound as a colorless solid (325 mg, 81%); R_f 0.35 (60% EtOAc/petroleum ether); m.p. 66–68 °C (from 40% EtOAc/petroleum ether); $\tilde{\nu}_{max}$ (thin film)/cm^{-1} 2982 (C-H, m), 1650 (C=O, s), 1578 (N-O, m); ^{1}H NMR (400 MHz, $CDCl_3$) δ_H 6.69 (1H, s, 4-C*H*), 3.79 (3H, s, 3^{ii}-CH_3), 3.35 (3H, s, 2^{ii}-CH_3), 2.35 (3H, s, 1^{i}-CH_3); ^{13}C NMR (101 MHz, $CDCl_3$) δ_C 161.7 (1^{ii}-C), 160.1 (3-C), 157.5 (5-C), 109.6 (4-C), 62.2 (3^{ii}-C), 33.2 (2^{ii}-C), 11.5 (1^{i}-C); HRMS *m/z* (ESI$^+$) [Found: 171.0764, $C_7H_{11}O_3N_2$ ([M+H]$^+$) requires 171.0764]; LRMS *m/z* (ESI$^+$) 171 ([M+H]$^+$, 100%), 193 ([M+Na]$^+$, 21%); HPLC Retention time 5.7 min, >99.0%.

1-(3-Methylisoxazol-5-yl)prop-2-en-1-one (4)

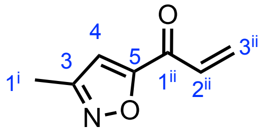

Vinyl magnesium bromide (0.7 M in THF, 1.90 mL, 1.32 mmol, 1.5 eq) was added dropwise over 5 min to a solution of *N*-methoxy-*N*-3-dimethylisoxazole-5-carboxamide (**50**) (150 mg, 0.88 mmol, 1.0 eq) in anhydrous THF (3 mL) at −35 °C. The solution was slowly warmed to 0 °C, and then stirred for 2 h. After this time the reaction was quenched by pouring over ice-cold aqueous HCl (2.0 M, 30 mL). The organic components were extracted with ice-cold EtOAc (3 × 30 mL). These extracts were washed with cold H_2O (50 mL), brine (50 mL), dried over $MgSO_4$, filtered, and concentrated *in vacuo*. The crude yellow solid was purified using silica gel column chromatography, eluting with 20–40% EtOAc/petroleum ether, to yield the title compound as a colorless solid (68 mg, 56%); R_f 0.62 (40% EtOAc/petroleum ether); m.p. 148–150 °C (from CH_2Cl_2); $\tilde{\nu}_{max}$ (thin film)/cm^{-1} 3117 (C-H, w), 2917 (C-H, w), 1674 (C=O, s); ^{1}H NMR (400 MHz, $CDCl_3$) δ_H 7.10 (1H, dd, *J* 17.2, 10.6, 2^{ii}-C*H*), 6.82 (1H, s, 4-C*H*), 6.65 (1H, dd, *J* 17.2, 1.6, 3^{ii}-CH_xH$_y$), 6.04 (1H, dd, *J* 10.6, 1.6, 3^{ii}-CH$_x$$H_y$), 2.39 (3H, s, 1^{i}-C$H_3$) ; ^{13}C NMR (101 MHz, $CDCl_3$) δ_C 178.6 (1^{ii}-C), 166.7 (5-C), 160.7 (3-C), 132.5 (3^{ii}-C), 131.5 (2^{ii}-

C), 109.2 (4-C), 11.6 (1^i-C); HRMS *m/z* (ESI^+) [Found: 138.0551, $C_7H_8O_2N$ ($[M+H]^+$) requires 138.0549]; LRMS *m/z* (ESI^+) 138 ($[M+H]^+$, 100%); HPLC Retention time 7.0 min, >99.0%.

5-Methylisoxazole-3-carboxylic acid (52)[6]

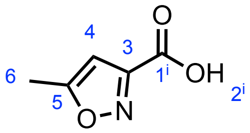

LiOH (195 mg, 4.64 mmol, 1.2 eq) was added to a solution of ethyl 5-methylisoxazole-3-carboxylate (**51**) (600 mg, 3.87 mmol, 1.0 eq) in THF/H_2O (2:1, 45 mL) and stirred for 16 h at rt. After this time the THF was removed *in vacuo*, and the remaining solution was acidified to pH 1 with aqueous HCl (6.0 M). The resulting solid was collected by filtration, washed with cold H_2O (25 mL), and dried *in vacuo*, to give the title compound as colorless solid (451 mg, 92%); R_f 0.10 (10% MeOH/EtOAc); m.p. 168–170 °C (from H_2O) [lit.[6] 172–174 °C]; ^{1}H NMR (400 MHz, $CDCl_3$) δ_H 6.47 (1H, s, 4-C*H*), 2.52 (3H, s, 6-C*H₃*); LRMS *m/z* (ESI^+) (128 $[M+H]^+$, 100%). These data are in good agreement with the literature.[6]

***N*-Methoxy-*N*-5-dimethylisoxazole-3-carboxamide (53)**

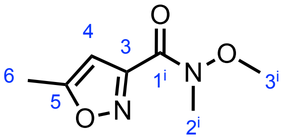

Anhydrous triethylamine (1.08 mL, 6.30 mmol, 4.0 eq) was added to a solution of 5-methylisoxazole-3-carboxylic acid (**52**) (200 mg, 1.57 mmol, 1.0 eq), and *N,O*-dimethylhydroxylamine hydrochloride (200 mg, 2.05 mmol, 1.3 eq), in anhydrous CH_2Cl_2 (10 mL). In a separate flask, PyBOP (2.13 g, 4.09 mmol, 2.6 eq) was dissolved in anhydrous CH_2Cl_2 (5 mL). The PyBOP solution was then added dropwise to the reaction solution at 0 °C, which was then warmed to rt and stirred for 16 h. After this time the solution was extracted with CH_2Cl_2 (20 mL), washed with H_2O (2 × 20 mL), saturated aqueous $NaHCO_3$ solution (20 mL), and brine (20 mL), dried over Na_2SO_4, filtered, and concentrated *in vacuo*. The crude yellow oil was further purified using silica gel column chromatography, eluting with 40–60% EtOAc/petroleum ether, to give the title compound as a colorless oil (218 mg, 82%); R_f 0.46 (60% EtOAc/petroleum ether); $\tilde{\nu}_{max}$ (thin film)/cm^{-1} 2938 (C-H, m), 1656 (C=O, s), 1599 (N-O, w); ^{1}H NMR (500 MHz, $CDCl_3$) δ_H 6.29 (1 H, s, 4-C*H*), 3.75 (3H, s, 3^i-CH_3), 3.35 (3H, s, 2^i-CH_3), 2.44 (3H, s, 6-CH_3); ^{13}C NMR (126 MHz, $CDCl_3$) δ_C 169.8 (5-C), 161.3 (1^i-C), 157.9 (3-C), 102.5 (4-C), 62.4 (3^i-C), 32.9 (2^i-C), 12.2 (6-C); HRMS *m/z* (ESI^+) [Found: 171.0764, $C_7H_{11}O_3N_2$ ($[M+H]^+$) requires 171.0764]; LRMS *m/z* (ESI^+) 171 ($[M+H]^+$, 33%), 193 ($[M+Na]^+$, 100%); HPLC Retention time 6.0 min, >99.0%.

1-(5-Methylisoxazol-3-yl)prop-2-en-1-one (5)

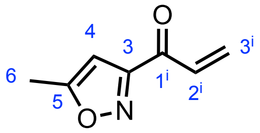

Vinyl magnesium bromide (0.7 M in THF, 2.83 mL, 1.98 mmol, 1.7 eq) was added dropwise over 5 min to *N*-methoxy-*N*-5-dimethylisoxazole-3-carboxamide (**53**) (189 mg, 1.16 mmol, 1.0 eq) dissolved in anhydrous THF (4 mL), at −35 °C. The resulting solution was slowly warmed to 0 °C, and stirred for 2 h. The reaction was quenched by pouring over ice-cold aqueous HCl (2.0 M, 30 mL), and extracted with ice-cold EtOAc (3 × 30 mL). The organic extracts were combined, washed with cold H_2O (50 mL), brine (50 mL), dried over $MgSO_4$, filtered, and concentrated *in vacuo*. The crude yellow solid was purified using silica gel column chromatography, eluting with 20–40% EtOAc/petroleum ether, to afford the title compound as a pale-yellow oil (103 mg, 65%); R_f 0.85 (40% EtOAc/petroleum ether); $\tilde{\nu}_{max}$ (thin film)/cm^{-1} 2981 (C-H, s), 2888 (C-H, m), 1658 (C=O, w), 955 (C=C, m); ^{1}H NMR (400 MHz, $CDCl_3$) δ_H 7.24 (1H, dd, *J* 17.3, 10.6, 2^i-C*H*), 6.65 (1H, dd, *J* 17.3, 1.5, 3^i-CH_xH$_y$), 6.43 (1H, q, *J* 1.0, 4-C*H*), 5.98 (1H, dd, *J* 10.6, 1.5, 3^i-CH$_x$$H_y$), 2.50 (3H, d, *J* 1.0, 6-CH_3); ^{13}C NMR (101 MHz, $CDCl_3$) δ_C 183.6 (1^i-C), 171.2 (5-C), 162.3 (3-C), 132.4 (2^i-C), 131.6 (3^i-C), 101.0 (4-C), 12.4 (6-C); HRMS *m/z* (ESI$^+$) [Found: 138.0549, $C_7H_8O_2N$ ([M+H]$^+$) requires 138.0550]; LRMS *m/z* (ESI$^+$) 138 [M+H]$^+$; HPLC Retention time 3.9 min, >99.0%.

Ethyl 4-bromo-3-methylisoxazole-5-carboxylate (55)[4]

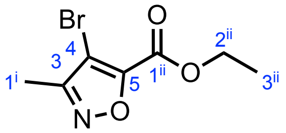

Following the procedure of Li *et al.*,[7] ethyl 3-methylisoxazole-5-carboxylate (**48**) (300 mg, 1.93 mmol, 1.0 eq), and *N*-bromosuccinimide (412 mg, 2.32 mmol, 1.2 eq) were dissolved in TFA (8 mL) in a microwave vial. The vial was sealed, and the solution was heated in a microwave reactor at 150 °C for 50 min. The solution was cooled, and TFA was removed by azeotropic distillation with toluene. The crude product was purified using silica gel chromatography, eluting with 0–10% Et_2O/petroleum ether, to give the title compound as a colorless oil (292 mg, 65%); R_f 0.30 (10% Et_2O/petroleum ether); ^{1}H NMR (400 MHz, $CDCl_3$) δ_H 4.44 (2H, q, *J* 7.2, 2ii-CH_2), 2.35 (3H, s, 1^i-CH_3), 1.42 (3H, t, *J* 7.2, 3^{ii}-CH_3); LRMS *m/z* (ESI$^+$) 234 ([^{79}M+H]$^+$, 100%), 236 ([^{81}M+H]$^+$, 95%). These data are in good agreement with the literature.[4,8]

Ethyl 3-methyl-4-phenylisoxazole-5-carboxylate (56)

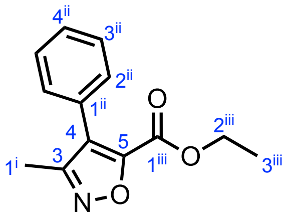

Ethyl 4-bromo-3-methylisoxazole-5-carboxylate (**55**) (350 mg, 1.50 mmol, 1.0 eq), phenylboronic acid (219 mg, 1.79 mmol, 1.2 eq), $NaHCO_3$ (378 mg, 4.50 mmol, 3.0 eq), and Pd(dppf)Cl_2·CH_2Cl_2 (37 mg, 0.045 mmol, 0.03 eq) were added to a microwave vial. The vial was sealed and purged with Ar. A degassed solution of in DME (4 mL), and H_2O (1 mL) was then added to the purged vial. The reaction was heated at 90 °C for 2.5 h. The reaction mixture was then cooled and filtered through a pad of Celite® and silica, washed with Et_2O (100 mL), and the filtrate concentrated *in vacuo*. The crude product was purified using silica gel column chromatography, eluting with 2–10% Et_2O/petroleum ether, to isolate the title compound as a colorless solid (170 mg, 49%); R_f 0.18 (15% Et_2O/petroleum ether); m.p. 56–58 °C (from $CHCl_3$) [lit.[8] 53 °C]; ^{1}H NMR (400 MHz, $CDCl_3$) δ_H 7.50–7.39 (3H, m, 3^{ii}, 4^{ii}-C*H*), 7.38–7.30 (2H, m, 2^{ii}-C*H*), 4.32 (2H, q, *J* 7.1, 2^{iii}-CH_2), 2.29 (3H, s, 1^{i}-CH_3), 1.27 (3H, d, *J* 7.1, 3^{iii}-CH_3); LRMS *m/z* (ESI$^+$) 232 ([M+H]$^+$, 100%). These data are in good agreement with the literature.[8]

3-Methyl-4-phenylisoxazole-5-carboxylic acid (57)

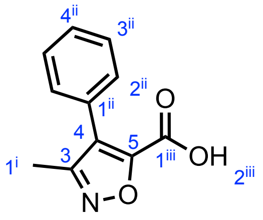

To a solution of ethyl 3-methyl-4-phenylisoxazole-5-carboxylate (**56**) (170 mg, 0.73 mmol, 1.0 eq) in THF (4 mL), and H_2O (2 mL), LiOH (70 mg, 2.94 mmol, 4.0 eq) was added at 0 °C. The reaction mixture was stirred and allowed to warm to rt. After 2 h, the THF was removed *in vacuo*, and the remaining solution was acidified to pH 2 with aqueous HCl (1.0 M). The suspension was cooled to 0 °C, filtered, and the solid washed with cold H_2O (10 mL). The solid was eluted from the filter with EtOAc (30 mL). The filtrate was concentrated *in vacuo* to isolate the title compound as a colorless solid (125 mg, 84%); R_f 0.63 (100% EtOH); m.p. 145–147 °C (from $CHCl_3$); $\tilde{\nu}_{max}$ (thin film)/cm^{-1} 1704 (C=O, m), 773 (C-H, s); ^{1}H NMR (500 MHz, $CDCl_3$) δ_H 7.46–7.41 (3H, m, 3^{ii}, 4^{ii}-C*H*), 7.34–7.32 (2H, m, 2^{ii}-C*H*), 2.29 (3H, s, 1^{i}-CH_3); ^{13}C NMR (126 MHz, $CDCl_3$) δ_C 160.9 (1^{ii}-C), 160.8 (3-C), 153.8 (5-C), 129.8 (2^{ii}-C), 129.1 (4^{ii}-C), 128.6 (3^{ii}-C), 127.9 (1^{ii}-C), 126.7 (4-C), 10.8 (1^{i}-C); HRMS *m/z* (ESI$^+$) [Found: 204.0657, $C_{11}H_{10}O_3N$ ([M+H]$^+$) requires 204.0655]; LRMS *m/z* (ESI$^+$) 204 ([M+H]$^+$, 100%); HPLC Retention time 8.0 min, >99.0%.

N-Methoxy-N,3-dimethyl-4-phenylisoxazole-5-carboxamide (58)

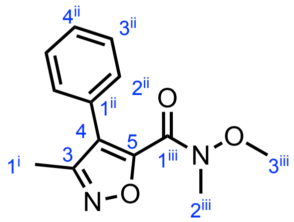

N,O-Dimethylhydroxylamine hydrochloride (68 mg, 0.69 mmol, 1.3 eq), and anhydrous triethylamine (221 µL, 1.60 mmol, 3.0 eq) were added to solution of 3-methyl-4-phenylisoxazole-5-carboxylic acid (**57**) (108 mg, 0.53 mmol, 1.0 eq) in anhydrous CH_2Cl_2 (3 mL). In a separate flask, PyBOP (554 mg, 1.06 mmol, 2.0 eq) was dissolved in anhydrous CH_2Cl_2 (2 mL), this solution was then added dropwise to the reaction mixture at 0 °C. The solution was slowly warmed to rt and stirred for 16 h. The reaction mixture was quenched with H_2O (20 mL), and extracted with CH_2Cl_2 (3 × 20 mL). The organic extracts were combined, washed with H_2O (20 mL), and brine (20 mL), dried over Na_2SO_4, filtered, and concentrated *in vacuo*. The crude product was purified using silica gel column chromatography, eluting with 10% EtOAc in petroleum ether, to isolate the title compound as a colorless oil (128 mg, 98%); R_f 0.63 (50% EtOAc/petroleum ether); $\tilde{\nu}_{max}$ (thin film)/cm^{-1} 2937 (C-H, m), 1662 (C=O); ^{1}H NMR (500 MHz, $CDCl_3$) δ_H 7.44–7.41 (2H, m, 3^{ii}-C*H*), 7.39–7.36 (3H, m, 2^{ii}, 4^{ii}-C*H*), 3.71 (3H, s, 3^{iii}-CH_3), 3.26 (3H, s, 2^{iii}-CH_3), 2.34 (3H, s, 1^{i}-CH_3); ^{13}C NMR (126 MHz, $CDCl_3$) δ_C 160.0 (1^{iii}-C), 159.1 (3-C), 158.1 (5-C), 129.3 (2^{ii}-C), 128.8 (3^{ii}-C), 128.7 (1^{ii}-C), 128.5 (4^{ii}-C), 121.5 (4-C), 62.4 (3^{iii}-C), 32.8 (2^{iii}-C), 10.8 (1^{i}-C); HRMS m/z (ESI$^+$) [Found: 247.1078, $C_{13}H_{15}O_3N_2$ ([M+H]$^+$) requires 247.1077]; LRMS m/z (ESI$^+$) 247 ([M+H]$^+$, 91%), 269 ([M+Na]$^+$, 100%); HPLC Retention time 9.2 min, >99.0%.

1-(3-Methyl-4-phenylisoxazol-5-yl)prop-2-en-1-one (6)

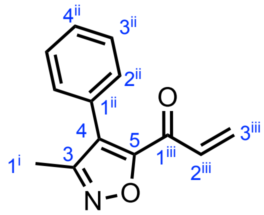

Vinyl magnesium bromide (1.0 M in THF, 446 µL, 0.447 mmol, 2.0 eq) was added dropwise over 5 min to a solution of N-methoxy-N-3-dimethyl-4-phenylisoxazole-5-carboxamide (**58**) (55 mg, 0.22 mmol, 1.0 eq) in anhydrous THF (3 mL) at −35 °C. The resulting solution was slowly warmed to 0 °C, and stirred for 1 h. The reaction was quenched by pouring over ice-cold aqueous HCl (1.0 M, 5 mL), and was then extracted with ice-cold EtOAc (2 × 20 mL). The organic extracts were washed with brine (10 mL), dried over Na_2SO_4, filtered, and concentrated *in vacuo*. The crude product was purified using silica gel column chromatography, eluting with 12–100% EtOAc/petroleum ether, to isolate the title compound as a colorless solid (24 mg, 62%); R_f 0.75 (50% EtOAc/petroleum ether); m.p. 37–39 °C (from $CHCl_3$); $\tilde{\nu}_{max}$ (thin film)/cm^{-1} 3062 (C-H, m), 1703 (C=O, s), 1607 (C=C, s); ^{1}H NMR (500 MHz,

$CDCl_3$) δ_H 7.48–7.42 (3H, m, 3^{ii}, 4^{ii}-C*H*), 7.38–7.35 (2H, m, 2^{ii}-C*H*), 7.05 (1H, dd, *J* 17.2, 10.5, 2^{iii}-C*H*), 6.54 (1H, dd, *J* 17.2, 1.5, 3^{iii}-CH_xH$_y$), 5.92 (1H, dd, *J* 10.5, 1.5, 3^{iii}-CH$_x$$H_y$), 2.32 (3H, s, 1^{i}-C$H_3$); ^{13}C NMR (126 MHz, $CDCl_3$) δ_C 179.3 (1^{iii}-C), 160.7 (5-C), 160.6 (3-C), 132.1 (2^{iii}-C), 131.8 (3^{iii}-C), 129.9 (2^{ii}-C), 129.1 (4^{ii}-C), 128.9 (3^{ii}-C), 128.4 (1^{ii}-C), 124.7 (4-C), 10.8 (1^{i}-C); HRMS *m/z* (ESI$^+$) [Found: 236.0681, $C_{13}H_{11}O_2NNa$ ([M+Na]$^+$ requires 236.0682]; LRMS *m/z* (ESI$^+$) 214 ([M+H]$^+$, 100%); HPLC Retention time 11.0 min, 96.4%.

***E*-3-[Methoxy(methyl)amino]-1-(3-methylisoxazol-5-yl)prop-2-en-1-one (7)**

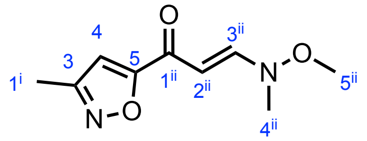

Ethynyl magnesium bromide (0.5 M in THF, 2.25 mL, 1.12 mmol, 2.0 eq) was added dropwise to a solution of *N*-methoxy-*N*-3-dimethylisoxazole-5-carboxamide (**50**) (95 mg, 0.56 mmol, 1.0 eq) in anhydrous THF (5 mL) at −40 °C. The solution was warmed to rt and stirred for 1 h, after which time it was cooled to −40 °C, and ethynyl magnesium bromide (0.5 M in THF, 1.13 mL, 0.56 mmol, 1.0 eq) was added dropwise. The reaction was quenched with H_2O (20 mL) and extracted with EtOAc (3 × 20 mL). The organic components were combined, washed with brine (20 mL), dried over $MgSO_4$, filtered, and concentrated *in vacuo*. The crude product was purified using silica gel column chromatography, eluting with 50–100% EtOAc/petroleum ether, to afford the title compound as a yellow solid (60 mg, 55%); R_f 0.23 (EtOAc); m.p. 60–62 °C (from $CHCl_3$); $\tilde{\nu}_{max}$ (thin film)/cm^{-1} 2981 (C-H, m), 1646 (C=O, m), 1557 (N-O, s), 1167 (N-H, m); ^{1}H NMR (400 MHz, $CDCl_3$) δ_H 7.74 (1H, d, *J* 12.4, 3^{ii}-C*H*), 6.66 (1H, s, 4-C*H*), 6.04 (1H, d, *J* 12.4, 2^{ii}-C*H*), 3.75 (3H, s, 5^{ii}-CH_3), 3.29 (3H, s, 4^{ii}-CH_3), 2.35 (3H, s, 1^{i}-CH_3); ^{13}C NMR (101 MHz, $CDCl_3$) δ_C 176.5 (1^{ii}-C), 169.0 (5-C), 160.5 (3-C), 149.3 (3^{ii}-C), 106.6 (4-C), 92.6 (2^{ii}-C), 60.2 (5^{ii}-C), 39.8 (4^{ii}-C), 11.7 (1^{i}-C); HRMS *m/z* (ESI$^+$) [Found: 197.0920, $C_9H_{13}O_3N_2$ ([M+H]$^+$) requires 197.0921]; LRMS *m/z* (ESI$^+$) 219 ([M+Na]$^+$, 100%), 197 ([M+H]$^+$, 98%); HPLC Retention time 4.9 min, 98.8%.

1-(3-Methylisoxazol-5-yl)prop-2-yn-1-one (8)

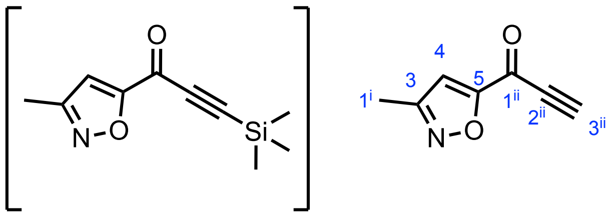

Isopropyl magnesium chloride (1.3 M, 1.22 mL, 1.59 mmol, 3.0 eq) was added dropwise over 5 min to a solution of ethynyltrimethylsilane (256 µL, 1.85 mmol, 3.5 eq) in anhydrous THF (5 mL) at −40 °C. The reaction mixture was slowly warmed to 0 °C. After 60 min, the solution was cooled to −40 °C and was added dropwise to a second flask containing *N*-methoxy-*N*-3-dimethylisoxazole-5-carboxamide

(**50**) (90 mg, 0.53 mmol, 1.0 eq) in anhydrous THF (5.0 mL) at −40 °C. The solution was warmed to 0 °C and stirred for 1.5 h. After this time the reaction was quenched slowly with aqueous NH_4Cl (15 mL), extracted with EtOAc (3 × 15 mL), washed with H_2O (20 mL), brine (20 mL), dried over Na_2SO_4, filtered, and concentrated *in vacuo*. The crude yellow oil was purified using silica gel column chromatography, eluting with toluene. During column chromatography the crude silylated product decomposed to reveal the de-silylated product as a pale-yellow solid (19 mg, 27%); R_f 0.16 (toluene); m.p. 60–62 °C (from $CHCl_3$) ; $\tilde{\nu}_{max}$ (thin film)/cm^{-1} 2982 (C-H, s), 1458 (C=O, w); ^{1}H NMR (500 MHz, $CDCl_3$) δ_H 6.91 (1H, s, 4-C*H*), 3.53 (1H, s, 3^{ii}-C*H*), 2.41 (3H, s, 1^i-CH_3); ^{13}C NMR (126 MHz, $CDCl_3$) δ_C 165.5 (1^{ii}-C), 163.8 (5-C), 160.9 (3-C), 111.2 (4-C), 82.5 (3^{ii}-C), 79.5 (2^{ii}-C), 11.6 (1^i-C); HRMS *m/z* (ESI$^-$) [Found: 134.0243, $C_7H_4O_2N$ ([M−H]$^-$) requires 134.0236]; LRMS *m/z* (ESI$^-$) 134 ([M−H]$^-$, 100%); HPLC Retention time 6.5 min, >99.0%.

1-(5-Methylisoxazol-3-yl)-3-(trimethylsilyl)prop-2-yn-1-one (54)

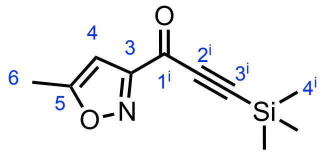

Isopropyl magnesium chloride (1.3 M, 3.38 mL, 4.41 mmol, 5.0 eq) was added dropwise, over a period of 5 min, to a solution of ethynyltrimethylsilane (794 µL, 5.73 mmol, 6.5 eq) in anhydrous THF (10 mL) at −40 °C. The solution was then slowly warmed to 0 °C. After 1 h the solution was cooled to −40 °C and was added dropwise to another flask containing *N*-methoxy-*N*-5-dimethylisoxazole-3-carboxamide (**53**) (150 mg, 0.88 mmol, 1.0 eq) dissolved in anhydrous THF (8 mL) at −40 °C. The solution was warmed to 0 °C and stirred for 1.5 h. The reaction was quenched slowly with aqueous NH_4Cl (20 mL), and extracted with EtOAc (3 × 25 mL). The combined organic components were washed with H_2O (20 mL), and brine (20 mL), dried over Na_2SO_4, filtered, and concentrated *in vacuo*. The crude product was purified using silica gel column chromatography, eluting with 0–10% Et_2O/petroleum ether, to isolate the title compound as a pale yellow solid (124 mg, 68%); R_f 0.80 (EtOAc); m.p. 42–44 °C (from $CHCl_3$); $\tilde{\nu}_{max}$ (thin film)/cm^{-1} 2981 (C-H, m), 2158 (C≡C, w), 1256 (Si-C, m); ^{1}H NMR (400 MHz, $CDCl_3$) δ_H 6.40 (1H, q, *J* 0.9, 4-C*H*), 2.49 (3H, d, *J* 0.9, 6-C*H*), 0.30 (9H, s, 4^i-CH_3); ^{13}C NMR (101 MHz, $CDCl_3$) δ_C 172.4 (1^i-C), 170.8 (3-C), 163.3 (5-C), 103.5 (2^i-C), 101.3 (4-C), 101.1 (3^i-C), 13.2 (6-C), 0.01 (4^i-C); HRMS *m/z* (ESI$^+$) [Found: 208.0789, $C_{10}H_{14}O_2N^{28}Si$ ([M+H]$^+$) requires 208.0788]; LRMS *m/z* (ESI$^+$) 230 ([M+Na]$^+$, 100%), 208 ([M+H]$^+$, 38%); HPLC Retention time 9.5 min, >99.0%.

1-(5-Methylisoxazol-3-yl)prop-2-yn-1-one (9)

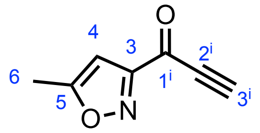

MeOH (137 µL, 3.40 mmol, 35 eq) was added dropwise to a suspension of 1-(5-methylisoxazol-3-yl)-3-(trimethylsilyl)prop-2-yn-1-one (**54**) (20 mg, 0.097 mmol, 1.0 eq), and K_2CO_3 (13 mg, 0.097, 1.0 eq) in CH_2Cl_2 (2 mL) at 0 °C. The suspension was stirred for 20 min and then diluted with CH_2Cl_2 (8 mL). The resulting solution was filtered through a silica plug, eluting with EtOAc (10 mL), and the eluate was concentrated *in vacuo*. The crude product was purified using silica gel column chromatography (toluene) to isolate the title compound as a colorless solid (6 mg, 46%); R_f 0.24 (toluene); m.p. 51–53 °C (from $CHCl_3$); $\tilde{\nu}_{max}$ (thin film)/cm^{-1} 2982 (C-H, s), 1458 (C=O, w); ^{1}H NMR (500 MHz, $CDCl_3$) δ_H 6.44 (1H, q, *J* 0.9, 4-C*H*), 3.52 (1H, s, 3^i-C*H*), 2.51 (3H, d, *J* 0.9, 6-CH_3); ^{13}C NMR (126 MHz, $CDCl_3$) δ_C 172.1 (1^i-C), 169.9 (5-C), 162.4 (3-C), 100.5 (4-C), 82.1 (3^i-C), 79.8 (2^i-C), 12.5 (6-C); HRMS *m/z* (ESI$^-$) [Found: 134.0243, $C_7H_4O_2N$ ([M−H]$^-$) requires 134.0236]; LRMS *m/z* (ESI$^-$) 134 ([M−H]$^-$, 100%); HPLC Retention time 6.4 min, 95.4%.

***N*-(3-Methylisoxazol-5-yl)prop-2-enamide (10)**

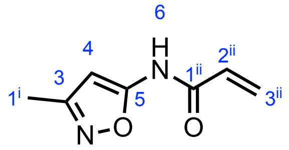

Acryloyl chloride (83 µL, 1.02 mmol, 1.0 eq) was added dropwise to a solution of 3-methylisoxazol-5-amine (**59**) (100 mg, 1.02 mmol, 1.0 eq), and anhydrous triethylamine (284 µL, 2.04 mmol, 2.0 eq) in anhydrous CH_2Cl_2 (10 mL) at 0 °C. The solution was warmed to rt and stirred for 16 h. After this time the reaction was quenched by the addition of H_2O (10 mL), and then extracted with CH_2Cl_2 (3 × 15 mL). The organic components were combined and washed with brine (20 mL), dried over Na_2SO_4, filtered, and concentrated *in vacuo*. The crude product was purified using silica gel column chromatography, eluting with 0–50% EtOAc/petroleum ether, to afford the title compound as a colorless solid (94 mg, 61%); R_f 0.44 (50% EtOAc/petroleum ether); m.p. 125–126 °C (from 50% EtOAc/petroleum ether); $\tilde{\nu}_{max}$ (thin film)/cm^{-1} 3213 (N-H, w), 3048 (C-H, w), 1680 (C=O, m), 1615 (C=C, m); ^{1}H NMR (500 MHz, $CDCl_3$) δ_H 8.16 (1H, s, 6-N*H*), 6.51 (1H, dd, *J* 16.9, 0.9, 3^{ii}-CH_xH$_y$), 6.30 (1H, s, 4-C*H*), 6.25 (1H, dd, *J* 16.9, 10.4, 2^{ii}-C*H*), 5.91 (1H, dd, *J* 10.4, 0.9, 3^{ii}-CH$_x$$H_y$), 2.29 (3H, s, 1^i-C$H_3$); ^{13}C NMR (126 MHz, $CDCl_3$) δ_C 161.8 (1^{ii}-C), 161.0 (3-C), 159.7 (5-C), 130.5 (3^{ii}-C), 129.2 (2^{ii}-C), 90.2 (4-C), 12.0 (1^i-C); HRMS *m/z* (ESI$^+$) [Found: 153.0658, $C_7H_9O_2N_2$ ([M+H]$^+$) requires 153.0658]; LRMS *m/z* (ESI$^+$) 153 ([M+H]$^+$, 100%); HPLC Retention time 4.9 min, >99.0%.

N-(5-Methylisoxazol-3-yl)prop-2-enamide (11)

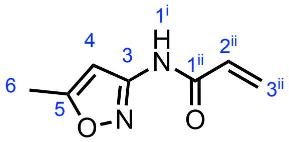

Acryloyl chloride (83 µL, 1.02 mmol, 1.0 eq) was added dropwise to a solution of 5-methylisoxazol-3-amine (**60**) (100 mg, 1.02 mmol, 1.0 eq), and anhydrous triethylamine (284 µL, 2.04 mmol, 2.0 eq) in anhydrous CH_2Cl_2 (10 mL) at 0 °C. The solution was warmed to rt and stirred for 16 h. After this time the reaction was quenched by the addition of H_2O (10 mL), and extracted with CH_2Cl_2 (3 × 15 mL). The organic components were combined, washed with brine (20 mL), dried over Na_2SO_4, filtered, and concentrated *in vacuo*. The crude material was purified using silica gel column chromatography, eluting with 0–50% EtOAc/petroleum ether, to isolate the title compound as a colorless solid (112 mg, 72%); R_f 0.52 (50% EtOAc/petroleum ether); m.p. 146–148 °C (from EtOAc); $\tilde{\nu}_{max}$ (thin film)/cm^{-1} 2924 (C-H, m), 1697 (C=O, s), 1622 (C=C, s); ^{1}H NMR (400 MHz, $CDCl_3$) δ_H 10.04 (1H, s, 1^i-N*H*), 6.83 (1H, s, 4-C*H*), 6.51 (1H, dd, *J* 17.0, 1.4, 3^{ii}-CH_xH$_y$), 6.39 (1H, dd, *J* 17.0, 10.1, 2^{ii}-C*H*), 5.87 (1H, dd, *J* 10.1, 1.4, 3^{ii}-CH$_x$$H_y$), 2.43 (3H, s, 6-C*H*); ^{13}C NMR (101 MHz, $CDCl_3$) δ_C 170.2 (5-C), 163.6 (1^{ii}-C), 158.6 (3-C), 130.4 (2^{ii}-C), 129.4 (3^{ii}-C), 97.0 (4-C), 12.8 (6-C); HRMS *m/z* (ESI$^+$) [Found: 153.0658, $C_7H_9O_2N_2$ ([M+H]$^+$) requires 153.0658]; LRMS *m/z* (ESI$^+$) 175 ([M+Na]$^+$, 100%); HPLC Retention time 4.5 min, >99.0%.

3-Oxo-2-phenylbutanenitrile (62)[9–12]

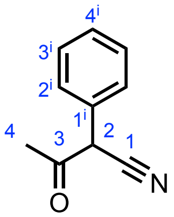

Using a modified procedure from Smith *et al.,*[9] sodium hydride (60% dispersion in mineral oil, 1.04 g, 26.0 mmol, 1.5 eq) was added portion-wise to a solution of benzyl cyanide (**61**) (2.00 mL, 17.3 mmol, 1.0 eq), and anhydrous DMF (3.5 mL) in anhydrous THF (35 mL) at 0 °C. The yellow suspension was stirred for 10 min at 0 °C before the dropwise addition of anhydrous EtOAc (6.77 mL, 69.2 mmol, 4.0 eq) at 0 °C. The suspension was stirred for 18 h at rt. The reaction was quenched at 0 °C by the addition of aqueous HCl (1.0 M, 15 mL), and extracted with EtOAc (4 × 50 mL). The organic components were combined, washed with brine (50 mL), dried over $MgSO_4$, filtered, and concentrated *in vacuo* to isolate the title compound as a pale-yellow solid. No further purification was required (1.87 g, 68%); R_f 0.50 (50% EtOAc/petroleum ether); m.p. 73–75 °C (from EtOAc) [lit.[12] 75–77 °C, lit.[11] 89–90 °C, lit.[10] 97 °C]; ^{1}H NMR (400 MHz, $CDCl_3$) δ_H 7.45–7.39 (5H, m, 2^i, 3^i, 4^i-C*H*), 4.67 (1H, s, 2-C*H*), 2.27 (3H, s, 4-CH_3); LRMS *m/z* (ESI$^+$) 160 ([M+H]$^+$, 100%). These data are in good agreement with the literature.[9–12]

3-Methyl-4-phenylisoxazol-5-amine (63)[13–15]

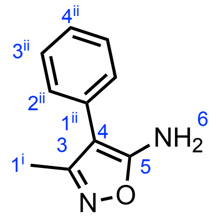

Using a modified procedure from Krasavin *et al.*,[13] aqueous Na_2CO_3 (10% *w/v*, 28 mL), and hydroxylamine hydrochloride (6.18 g, 89.0 mmol, 2.0 eq) were added to a solution of 3-oxo-2-phenylbutanenitrile (**62**) (7.08 g, 44.5 mmol, 1.0 eq) dissolved in EtOH (108 mL). The solution was heated under reflux for 40 min, then cooled and concentrated *in vacuo*. The crude material was dissolved in H_2O (20 mL) and extracted with EtOAc (3 × 100 mL). The organic components were combined, washed with brine (50 mL), dried over Na_2SO_4, filtered, and concentrated *in vacuo* to isolate the title compound as an orange solid. No further purification was required (6.81 g, 88%); R_f 0.53 (50% EtOAc/petroleum ether); ^{1}H NMR (400 MHz, $CDCl_3$) δ_H 7.47–7.38 (2H, m, 2^{ii}-C*H*), 7.34–7.26 (3H, m, 3^{ii}, 4^{ii}-C*H*), 4.51 (2H, s, 6-N*H₂*), 2.24 (3H, s, 1^{i}-C*H₃*); LRMS *m/z* (ESI$^+$) 175 ([M+H]$^+$, 100%). These data are in good agreement with the literature.[13–15]

***N*-(3-Methyl-4-phenylisoxazol-5-yl)prop-2-enamide (12)**

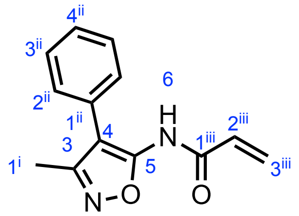

Acryloyl chloride (77 µL, 0.95 mmol, 1.1 eq) was added dropwise to a solution of 3-methyl-4-phenylisoxazol-5-amine (**63**) (150 mg, 0.861 mmol, 1.0 eq), and pyridine (77 µL, 0.95 mmol, 1.1 eq) in anhydrous CH_2Cl_2 (4.0 mL) at 0 °C. The solution was stirred at 0 °C for 2 h, and was then warmed to rt. After 8 h the reaction was cooled to 0 °C, and further portions of acryloyl chloride (35 µL, 0.43 mmol, 0.5 eq) and pyridine (35 µL, 0.43 mmol, 0.5 eq) were added. The reaction solution was stirred at 0 °C for a further 10 h, after which time the reaction was quenched by the addition of aqueous HCl (1.0 M, 10 mL), and then extracted with CH_2Cl_2 (2 × 20 mL). The organic extracts were washed with H_2O (20 mL), and brine (20 mL), dried over Na_2SO_4, filtered, and concentrated *in vacuo*. The crude product was purified using silica gel column chromatography, eluting with 25–50% Et_2O/petroleum ether, to isolate the title compound as a colorless solid (25 mg, 13%); R_f 0.11 (50% Et_2O/petroleum ether); m.p. 85– 87 °C (from $CHCl_3$); $\tilde{\nu}_{max}$ (thin film)/cm^{-1} 3228 (N-H, m), 3035 (C-H, w), 1685 (C=O, s), 1642 (C=C, s); ^{1}H NMR (500 MHz, D_6-DMSO) δ_H 10.74 (1H, s, 6-N*H*), 7.46–7.40 (2H, m, 3^{ii}-C*H*), 7.40–7.34 (3H m, 2^{ii}, 4^{ii}-C*H*), 6.37 (1H, dd, *J* 17.0, 10.1, 2^{iii}-C*H*), 6.27 (1H, dd, *J* 17.0, 1.8, 3^{iii}-CH_xH$_y$), 5.83 (1H, dd, *J* 10.1, 1.8, 3^{iii}-CH$_x$$H_y$), 2.28 (3H, s, 1^{i}-C*H₃*); ^{13}C NMR (126 MHz, D_6-

DMSO) δ_C 163.8 (1^{iii}-C), 159.8 (3-C), 156.9 (5-C), 129.7 (2^{iii}-C), 129.2 (3^{iii}-C), 129.0 (1^{ii}-C), 128.7 (3^{ii}-C), 128.4 (2^{ii}-C), 127.7 (4^{ii}-C), 110.0 (4-C), 11.3 (1^{i}-C); HRMS *m/z* (ESI^+) [Found: 229.0973, $C_{13}H_{13}O_2N_2$ ($[M+H]^+$) requires 229.0972]; LRMS *m/z* (ESI^+) 229 ($[M+H]^+$, 100%); HPLC Retention time 8.3 min, >99.0%.

(*E*)-4-(Dimethylamino)-*N*-(3-methylisoxazol-5-yl)but-2-enamide (13)

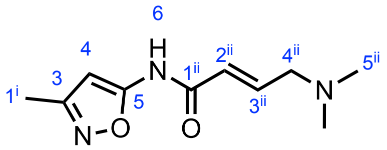

Oxalyl chloride (54 µL, 0.64 mmol, 1.05 eq) was added dropwise to a suspension of (*E*)-4-dimethylaminobut-2-enoic acid hydrochloride (100 mg, 0.61 mmol, 1.0 eq) in anhydrous THF (3 mL), and anhydrous DMF (1 drop) at 0 °C. The resulting suspension was stirred at 0 °C for a further 30 min and then warmed to rt and stirred for 2 h. The solution was then cooled to 0 °C, and 3-methylisoxazol-5-amine (**59**) (65 mg, 0.67 mmol, 1.1 eq) dissolved in anhydrous THF (3 mL) and anhydrous triethylamine (100 µL) were added dropwise. The resulting solution was stirred for 2 h at rt. After this time the reaction was quenched but the addition of a saturated aqueous $NaHCO_3$ solution until pH 10 was attained, and then extracted with EtOAc (3 × 30 mL). The organic components were combined, washed with brine (50 mL), dried over $MgSO_4$, filtered, and concentrated *in vacuo*. The crude product was purified using preparative TLC, eluting with 92% CH_2Cl_2/7% EtOH/1% NH_4OH, to give the title compound as a colorless solid (9 mg, 7%); R_f 0.10 (92% CH_2Cl_2/7% EtOH/1% NH_4OH); m.p. 96–98 °C (from $CHCl_3$); $\tilde{\nu}_{max}$ (thin film)/cm^{-1} 2980 (C-H, m), 1686 (C=O, m), 1613 (C=C, m), 1267 (C-N, w); ^{1}H NMR (500 MHz, $CDCl_3$) δ_H 9.13 (1H, br s, 6-N*H*), 7.05 (1H, dt, *J* 15.4, 6.0, 3^{ii}-C*H*), 6.28 (1H, s, 4-C*H*), 6.15 (1H, dt, *J* 15.4, 1.6, 2^{ii}-C*H*), 3.14 (2H, dd, *J* 6.0, 1.6, 4^{ii}-CH_2), 2.29 (6H, s, 5^{ii}-CH_3), 2.27 (3H, s, 1^{i}-CH_3); ^{13}C NMR (126 MHz, $CDCl_3$) δ_C 161.8 (1^{ii}-C), 161.3 (3-C), 160.2 (5-C), 145.0 (3^{ii}-C), 123.9 (2^{ii}-C), 90.0 (4-C), 60.3 (4^{ii}-C), 45.5 (5^{i}-C), 11.9 (1^{i}-C); HRMS *m/z* (ESI^+) [Found: 210.1238, $C_{10}H_{16}O_2N_3$ ($[M+H]^+$) requires 210.1237]; LRMS *m/z* (ESI^+) 210 ($[M+H]^+$, 100%); HPLC Retention time 7.8 min, 96.0%.

(*E*)-4-Bromo/Chloro-*N*-(3-methyl-4-phenylisoxazol-5-yl)but-2-enamide (65)

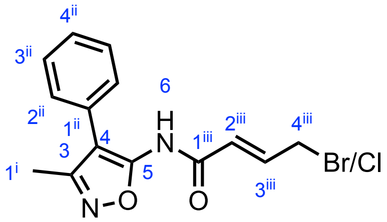

Oxalyl chloride (1.07 mL, 12.6 mmol, 2.2 eq) was added dropwise to a solution of (*E*)-4-bromocrotonic acid (**64**) (1.42 g, 8.61 mmol, 1.5 eq) in anhydrous CH_2Cl_2 (10 mL), and anhydrous DMF (2 drops) at 0 °C. The resulting solution was stirred at 0 °C for a further 30 min and then warmed to rt and stirred for 20 h. The solution was then concentrated *in vacuo*, and the pale-brown oil redissolved in anhydrous CH_2Cl_2 (8.6 mL).

The presumed acyl chloride solution was added dropwise to a solution of 3-methyl-4-phenylisoxazol-5-amine (**63**) (1.00 g, 5.74 mmol, 1.0 eq) and pyridine (557 μL, 6.89 mmol, 1.3 eq) in anhydrous CH_2Cl_2 (25 mL), at −15 °C. The reaction was stirred at −15 °C for 40 min before being quenched by the addition of aqueous HCl (1.0 M, 30 mL), and extracted with CH_2Cl_2 (3 × 100 mL). The organic extracts were washed with H_2O (50 mL), and brine (50 mL), dried over Na_2SO_4, filtered, and concentrated *in vacuo*. The crude brown oil was purified using silica gel column chromatography, eluting with 40–70% Et_2O/petroleum ether, to isolate a mixture of the bromo- or chloro-substituted title compound as a pale-brown solid (450 mg, 24%); R_f 0.29 (60% Et_2O/petroleum ether); ^{1}H NMR (500 MHz, $CDCl_3$) δ_H 7.76 (1H, s, 6-N*H*), 7.44–7.41 (2H, m, 3^{ii}-C*H*), 7.37–7.34 (1H, m, 4^{ii}-C*H*), 7.28–7.26 (2H, m, 2^{ii}-C*H*), 7.06–6.99 (2H, m, 3^{iii}-C*H*), 6.28 (0.7H, d, *J* 15.0, 2^{iii}-C$H_{(Cl)}$), 6.22 (0.3H, d, *J* 15.0, 2^{iii}-C$H_{(Br)}$), 4.17 (1.5H, dd, *J* 5.6, 1.3, 4^{iii}-C$H_{2(Cl)}$), 4.00 (0.5H, dd, *J* 7.0, 1.3, 4^{iii}-C$H_{2(Br)}$), 2.29 (3H, s, 1^{i}-CH_3); LRMS *m/z* (ESI$^+$) 277, 279 ([M+H]$^+$; ^{35}Cl, 100%; ^{37}Cl, 30%), 321, 323 ([M+H]$^+$; ^{79}Br, 100%; ^{81}Br, 95%).

(*E*)-4-Iodo-*N*-(3-methyl-4-phenylisoxazol-5-yl)but-2-enamide (66)

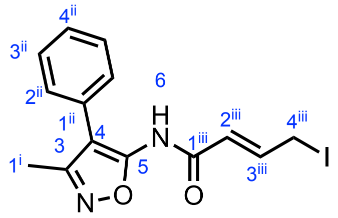

Sodium iodide (105 mg, 0.70 mmol, 1.5 eq) was added to a solution of (*E*)-4-bromo/chloro-*N*-(3-methyl-4-phenylisoxazol-5-yl)but-2-enamide (**65**) (150 mg, 0.47 mmol, 1.0 eq) in acetone (4.0 mL). The reaction mixture was heated at 50 °C for 40 min, after which time it was diluted with acetone (10 mL), filtered, and concentrated *in vacuo* to afford the title compound as a brown oil. This intermediate was used directly in the next step without further purification; R_f 0.29 (60% Et_2O/petroleum ether); ^{1}H NMR (400 MHz, $CDCl_3$) δ_H 7.57 (1H, br s, 6-N*H*), 7.46–7.41 (2H, m, 3^{ii}-C*H*), 7.39–7.35 (1H, m, 4^{iii}-C*H*), 7.30–7.27 (2H, m, 2^{ii}-C*H*), 7.09 (1H, dt, *J* 14.9, 8.3, 3^{iii}-C*H*), 6.13

(1H, d, J 14.9, 2^{iii}-CH), 3.92 (2H, dd, J 8.3, 1.0, 4^{iii}-CH_2), 2.30 (3H, s, 1^{i}-CH_3); LRMS m/z (ESI$^+$) 369 ([M+H]$^+$, 100%).

(*E*)-4-(Dimethylamino)-*N*-(3-methyl-4-phenylisoxazol-5-yl)but-2-enamide (14)

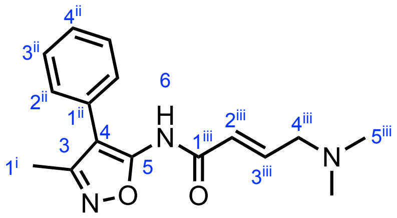

Method 1:

The crude (*E*)-4-iodo-*N*-(3-methyl-4-phenylisoxazol-5-yl)but-2-enamide (**66**) was dissolved in anhydrous DMF (3.0 mL), and K_2CO_3 (129 mg, 0.94 mmol, 2.0 eq) was added. The resulting suspension was cooled to 0 °C and dimethylamine solution (2.0 M in THF, 235 µL, 0.47 mmol, 1.0 eq) was added dropwise. After 1 h, the reaction was quenched by the addition of saturated aqueous $NaHCO_3$ solution (10 mL), extracted with EtOAc (3 × 30 mL), washed with brine (30 mL), dried over Na_2SO_4, filtered, and concentrated *in vacuo*. The crude product was purified using silica gel column chromatography, eluting with 0–50% 12:2:1 EtOAc: EtOH: NH_4OH/EtOAc, to give the title compound as a colorless oil (27 mg, 31% over two steps), the characterization data obtained were identical to those shown below.

Method 2:

Using a modified procedure from Chew *et al.*,[16] oxalyl chloride (284 µL, 3.35 mmol, 1.95 eq) was added dropwise to a suspension of (*E*)-4-(dimethylamino)but-2-enoic acid hydrochloride (570 mg, 3.44 mmol, 2.0 eq) in anhydrous THF (4.0 mL), and anhydrous DMF (2 drops) at 0 °C. The suspension was stirred at 0 °C for a further 20 min and then warmed to rt and stirred for 60 min. The solution was then cooled to 0 °C. 3-Methyl-4-phenylisoxazol-5-amine (**63**) (300 mg, 1.72 mmol, 1.0 eq) in anhydrous *N*-methyl-2-pyrrolidone (2 mL) was then added dropwise at 0 °C, and the reaction mixture stirred for 21 h at 5 °C. After this time, the reaction was quenched by the addition of aqueous HCl (1.0 M, 20 mL), and washed with EtOAc (30 mL). The acidic aqueous extract was basified with aqueous NaOH (2.0 M) until pH 11 was attained, extracted with EtOAc (3 × 30 mL), washed with brine (30 mL), dried over Na_2SO_4, filtered, and concentrated *in vacuo*. The crude product was purified using silica gel column chromatography, eluting with 0–50% 12:2:1 EtOAc:EtOH:NH_4OH/EtOAc, followed by further purification using semi-preparative HPLC (see general chemical methods) to isolate the title compound as the formic acid salt, which was then dissolved in aqueous NaOH (1.0 M), extracted with EtOAc (3 × 20 mL), and concentrated *in vacuo* to isolate the free base as a colorless oil (164 mg, 33%); R_f 0.21 (50% 12:2:1 EtOAc:EtOH:NH_4OH/EtOAc); $\tilde{\nu}_{max}$ (thin film)/cm^{-1} 3211 (N-H, w br), 3035 (C-H, w), 1675 (C=O); ^{1}H NMR (500 MHz, $CDCl_3$) δ_H 7.44–7.40 (2H, m, 3^{ii}-CH), 7.37–7.33 (1H, m, 4^{ii}-CH), 7.29–7.27 (2H, m, 2^{ii}-CH), 6.97 (1H, dt, J 15.5, 5.9, 3^{iii}-CH_2), 6.14 (1H, d, J 15.5, 2^{iii}-CH), 3.06 (2H,

dd, J 5.9, 1.6, 4^{iii}-CH_2), 2.30 (3H, s, 1^{i}-CH_3), 2.22 (6H, s, 5^{iii}-CH_3); ^{13}C NMR (126 MHz, $CDCl_3$) δ_C 163.5 (1^{iii}-C), 160.3 (3-C), 156.3 (5-C), 146.0 (3^{iii}-C), 129.2 (3^{ii}-C), 129.1 (1^{ii}-C), 128.8 (2^{ii}-C) 128.2 (4^{ii}-C), 122.9 (2^{ii}-C), 108.9 (4-C), 60.4 (4^{ii}-C), 45.7 (5^{ii}-C), 11.6 (1^{i}-C); HRMS *m/z* (ESI$^+$) [Found: 286.1548, $C_{16}H_{20}O_2N_3$ ([M+H]$^+$) requires 285.1561]; LRMS *m/z* (ESI$^+$) 286 ([M+H]$^+$, 100%); HPLC Retention time 6.2 min, >99.0%.

(*E*)-*N*-(3-methyl-4-phenylisoxazol-5-yl)but-2-enamide (15)

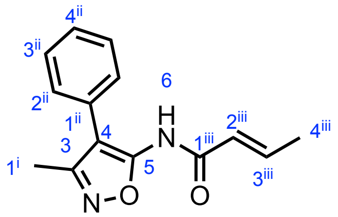

Crotonoyl chloride (319 µL, 3.00 mmol, 1.5 eq) was added dropwise to a solution of 3-methyl-4-phenylisoxazol-5-amine (**63**) (348 mg, 2.00 mmol, 1.0 eq), and pyridine (324 µL, 4.00 mmol, 2.0 eq) in anhydrous CH_2Cl_2 (10 mL) at 0 °C. The resulting solution was stirred at 0 °C for 2 h, before the reaction was quenched by the addition of aqueous HCl (1.0 M, 20 mL), and extracted with CH_2Cl_2 (3 × 30 mL). The organic extracts were combined, washed with aqueous NaOH (1.0 M, 20 mL) and brine (30 mL), dried over Na_2SO_4, filtered, and concentrated *in vacuo*. The crude product was purified using silica gel column chromatography, eluting with 20–70% Et_2O/petroleum ether, to afford the title compound as a colorless oil (25 mg, 13%); R_f 0.24 (60% Et_2O/petroleum ether); $\tilde{\nu}_{max}$ (thin film)/cm^{-1} 3220 (N-H, m br), 2967 (C-H, m), 1690 (C=O, m), 1643 (C=C, s); ^{1}H NMR (500 MHz, $CDCl_3$) δ_H 7.41–7.38 (2H, m, 3^{ii}-CH), 7.34–7.31 (2H, m, 6-NH, 4^{ii}-CH), 7.26–7.23 (2H, m, 2^{ii}-CH), 6.98 (1H, dq, J 15.1, 6.9, 3^{iii}-CH), 5.98 (1H, d, J 15.1, 2^{iii}-CH), 2.27 (3H, s, 1^{i}-CH_3), 1.85 (3H, dd, J 6.9, 1.6, 4^{iii}-CH_3); ^{13}C NMR (126 MHz, $CDCl_3$) δ_C 163.7 (1^{iii}-C), 160.3 (3-C), 156.4 (5-C), 145.3 (3^{iii}-C), 129.2 (3^{ii}-C), 129.1 (1^{ii}-C), 128.9 (2^{ii}-C), 128.2 (4^{ii}-C), 122.8 (2^{ii}-C), 108.9 (4-C), 18.2 (4^{iii}-C), 11.6 (1^{i}-C); HRMS *m/z* (ESI$^+$) [Found: 243.1130, $C_{14}H_{15}O_2N_2$ ([M+H]$^+$) requires 243.1128]; LRMS *m/z* (ESI$^+$) 243 ([M+H]$^+$, 100%); HPLC Retention time 8.7 min, 97.5%.

3-Methylisoxazole-5-carboxamide (67)[4]

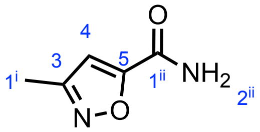

Ethyl 3-methylisoxazole-5-carboxylate (**48**) (800 mg, 5.16 mmol, 1.0 eq) was dissolved in a methanolic solution of NH_3 (3.0 M, 20 mL) and stirred for 16 h at rt. The solution was then concentrated *in vacuo* to obtain the crude product, which was purified using silica gel column chromatography, eluting with 20–50% EtOAc/petroleum ether, to afford the title compound as a colorless solid (575 mg, 89%); R_f 0.28 (50% EtOAc/petroleum ether); m.p. 142–145 °C dec. (from MeOH); ^{1}H NMR (400 MHz, $CDCl_3$)

δ_H 6.78 (1H, s, 4-C*H*), 6.45 (1H, br s, 2^{ii}-NH_AH_B), 5.83 (1H, br s, 2^{ii}-NH_AH_B), 2.37 (3H, s, 1^{i}-CH_3); LRMS *m/z* (ESI$^+$) 127 ([M+H]$^+$, 100%). These data are in good agreement with the literature.[4]

(3-Methylisoxazol-5-yl)methanamine hydrochloride (68)[4]

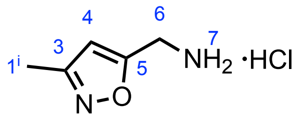

Following a procedure modified from Dannhardt *et al.*,[17] 3-methylisoxazole-5-carboxamide (**67**) (350 mg, 2.78 mmol, 1.0 eq) was added to a microwave vial, dissolved in anhydrous THF (28 mL), and the vial sealed. $BH_3 \cdot SMe_2$ complex in THF (2.0 M, 6.95 mL, 13.9 mmol, 5.0 eq) was added dropwise at 0 °C. The solution was heated under reflux for 6 h. After this time the solution was cooled to 0 °C, and MeOH (16.5 mL) was added dropwise with vigorous stirring. The solution was stirred for a further 16 h at rt, and then concentrated *in vacuo*. Aqueous HCl (1.0 M, 16 mL) was added to the resulting oil and the solution heated under reflux for 1 h. The solution was cooled to 0 °C, basified to pH 10 with NaOH (1.0 M), extracted with CH_2Cl_2 (3 × 30 mL), washed with brine (30 mL), dried over Na_2SO_4, filtered, and concentrated *in vacuo*. The resulting yellow oil was dissolved in Et_2O (5 mL) and HCl (2.0 M in Et_2O, 3 mL) was added. The resulting solid was collected by filtration and dried *in vacuo*, to isolate the title compound as colorless solid (270 mg, 66%). R_f 0.06 (10% MeOH/EtOAc); m.p. 172–174 °C dec. (from Et_2O) [lit.[4] 175 °C] ^{1}H NMR (400 MHz, DMSO-D$_6$) δ_H 8.70 (3H, br s, 7-NH_3), 6.50 (1H, s, 4-C*H*), 4.21 (2H, s, 6-C*H*), 2.25 (3H, s, 1^{i}-C*H*); LRMS *m/z* (ESI$^+$) 113 ([M+H]$^+$, 100%). These data are in good agreement with the literature.[4]

***N*-[(3-Methylisoxazol-5-yl)methyl]prop-2-enamide (16)**

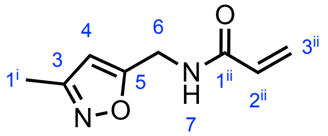

To a solution of (3-methylisoxazol-5-yl)methanamine hydrochloride (**68**) (90 mg, 0.61 mmol, 1.1 eq) in anhydrous CH_2Cl_2 (6 mL) was added K_2CO_3 (92 mg, 0.66 mmol, 1.2 eq). The solution was cooled to 0 °C and acryloyl chloride (56 µL, 0.55 mmol, 1.0 eq) was added dropwise. The solution was then slowly warmed to rt, and stirred for 3 h. After this time the reaction solution was diluted with CH_2Cl_2 (10 mL) and washed with H_2O (10 mL). The organic components were extracted with CH_2Cl_2 (3 × 10 mL), combined, washed with brine (20 mL), dried over Na_2SO_4, filtered, and concentrated *in vacuo.* The resulting yellow oil was purified using silica gel column chromatography, eluting with 0–50% EtOAc/petroleum ether, to isolate the title compound as a colorless solid (76 mg, 75%); R_f0.19 (50% EtOAc/petroleum ether); m.p. 55–57 °C (from $CHCl_3$) ; $\tilde{\nu}_{max}$ (thin film)/cm^{-1} 3277 (N-H, s br), 2980 (C-H, m), 1662 (C=O, s), 1608 (C=C, s); ^{1}H NMR (400 MHz, $CDCl_3$) δ_H 6.43 (1H, s, 7-N*H*), 6.32 (1H, dd, *J* 17.0, 1.4, 3^{ii}-CH_xH_y), 6.13 (1H, dd, *J* 17.0, 10.3, 2^{ii}-C*H*), 6.04 (1H, s, 4-C*H*), 5.69 (1H, dd, *J* 10.3,

1.4, 3^{ii}-CH_xH_y), 4.57 (2H, d, *J* 6.0, 6-C*H*), 2.25 (3H, s, 1^{i}-C*H*); ^{13}C NMR (101 MHz, $CDCl_3$) δ_C 168.4 (1^{ii}-C), 165.6 (5-C), 160.2 (C-3), 130.1 (2^{ii}-C), 127.7 (3^{ii}-C), 103.3 (4-C), 35.3 (6-C), 11.5 (1^{i}-C); HRMS *m/z* (ESI^+) [Found: 167.0815, $C_8H_{11}O_2N_2$ ($[M+H]^+$) requires 167.0815]; LRMS *m/z* (ESI^+) 167 ($[M+H]^+$, 100%), 189 ($[M+Na]^+$, 29%); HPLC Retention time 4.2 min, 98.6%.

5-Methylisoxazole-3-carboxamide (69)[18]

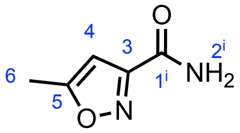

Ethyl 5-methylisoxazole-3-carboxylate (**51**) (500 mg, 3.22 mmol, 1.0 eq) was dissolved in a methanolic solution of NH_3 (3.0 M, 12 mL) and stirred for 16 h at rt. The solution was then concentrated *in vacuo* to afford the crude product, which was purified using silica gel column chromatography, eluting with 50% EtOAc/petroleum ether, to isolate the title compound as colorless solid (386 mg, 95%); R_f 0.42 (50% EtOAc/petroleum ether); m.p. 164–166 °C (from CH_2Cl_2) [lit.[18] 166 °C]; ^{1}H NMR (400 MHz, D_6-DMSO) δ_H 8.03 (1H, br s, 2^{i}-NH_AH_B), 7.75 (1H, br s, 2^{i}-NH_AH_B), 6.50 (1H, s, 4-C*H*), 2.45 (3H, s, 6-CH_3); LRMS *m/z* (ESI^+) 127 ($[M+H]^+$, 92%), 149 ($[M+Na]^+$, 100%). These data are in good agreement with the literature.[18]

1^{i}-(5-Methylisoxazol-3-yl methanamine) hydrochloride (70)

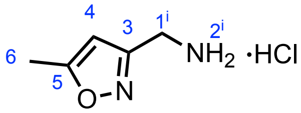

Following a procedure modified from Dannhardt *et al.*,[17] 5-methylisoxazole-3-carboxamide (**69**) (100 mg, 0.79 mmol, 1.0 eq) was added to a microwave vial, dissolved in anhydrous THF (5 mL), and the vial sealed. $BH_3\cdot SMe_2$ complex in THF (2.0 M, 1.70 mL, 3.17 mmol, 4.0 eq) was then added at 0 °Cs The solution was heated under reflux for 6 h, after which time the reaction was cooled to 0 °C, and MeOH (2.5 mL) was added dropwise with vigorous stirring. The vial was sealed and stirred for a further16 h at rt. After this time the solution was concentrated *in vacuo*. Aqueous HCl (1.0 M, 2.5 mL) was added to the resulting oil, and the solution was heated under reflux for 1 h. The solution was cooled to 0 °C, basified to pH 10 with aqueous NaOH (1.0 M), and extracted with CH_2Cl_2 (3 × 15 mL). The organic components were combined, washed with brine (20 mL), dried over Na_2SO_4, filtered, and concentrated *in vacuo*. The resulting yellow oil was dissolved in Et_2O (2 mL) and HCl (2.0 M in Et_2O, 0.70 mL) was added. The colorless solid was collected by filtration and dried *in vacuo*, to isolate the title compound as colorless solid (39 mg, 33%); R_f 0.08 (10% MeOH/EtOAc); m.p. 198–201 °C dec. (from Et_2O) [lit.[19] 202–203 °C (dec.)]; ^{1}H NMR (400 MHz, D_4-methanol) δ_H 6.28 (1 H, s, 4-C*H*), 4.23 (2H, s, 1^{i}-CH_2), 2.47 (3H, s, 6-CH_3); LRMS *m/z* (ESI^+) 113 ($[M+H]^+$, 100%). These data are in good agreement with the literature values.[19]

***N*-[(5-Methylisoxazol-3-yl)methyl]prop-2-enamide (17)**

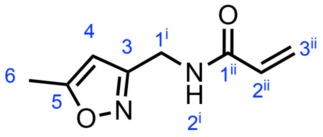

Triethylamine (50 µL, 0.50 mmol, 3.0 eq) and acryloyl chloride (27 µL, 0.34 mmol, 2.0 eq) were added to a solution of 1^{i}-(5-methylisoxazol-3-yl methanamine) hydrochloride (**70**) (25 mg, 0.17 mmol, 1.0 eq) in anhydrous CH_2Cl_2 (2 mL) at 0 °C. The solution was warmed to rt, stirred for 18 h, and then concentrated *in vacuo*, to obtain the crude product which was purified using silica gel column chromatography, eluting with 0–50% EtOAc/petroleum ether, to isolate the title compound as a colorless solid (10 mg, 36%); R_f 0.21 (50% EtOAc/petroleum ether); m.p. 158–160 °C (from CH_2Cl_2); $\tilde{\nu}_{max}$ (thin film)/cm^{-1} 3281 (N-H, m), 2927 (C-H, w), 1655 (C=O, s) 1610 (C=C, s); ^{1}H NMR (400 MHz, $CDCl_3$) δ_H 6.32 (1H, dd, *J* 17.0, 1.4, 3^{ii}-CH_yH$_x$), 6.20 (1H, br s, 2^{i}-N*H*), 6.14 (1H, dd, *J* 17.0, 10.3, 2^{ii}-C*H*), 5.99 (1H, q, *J* 0.8, 4-C*H*), 5.69 (1H, dd, *J* 10.3, 1.4, 3^{ii}-CH$_x$$H_y$), 4.54 (2H, d, *J* 5.7, 1^{i}-CH_2), 2.29 (3H, d, *J* 0.8, 6-CH_3); ^{13}C NMR (101 MHz, $CDCl_3$) δ_C 170.3 (5-C), 165.7 (1^{ii}-C), 161.1 (3-C), 130.4 (3^{ii}-C), 127.4 (2^{ii}-C), 101.3 (4-C), 35.7 (1^{i}-C), 12.4 (6-C); LRMS *m/z* (ESI$^+$) 167 ([M+H]$^+$, 7%), 189 ([M+Na]$^+$, 100%); HRMS *m/z* (ESI$^+$) [Found: 167.0815, $C_8H_{11}O_2N_2$ ([M+H]$^+$) requires 167.0815]; HPLC Retention time 5.1 min, 97.6%.

(*E*)-4-(Dimethylamino)-*N*-(3-methylisoxazol-5-yl)but-2-enamide (18)

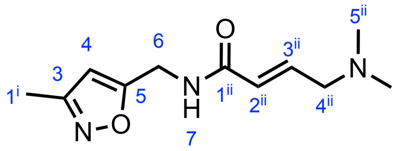

Oxalyl chloride (74 µL, 0.86 mmol, 1.6 eq) was added dropwise to a suspension of (*E*)-4-dimethylaminobut-2-enoic acid hydrochloride (124 mg, 0.75 mmol, 1.4 eq) in anhydrous THF (5 mL) at 0 °C. The suspension was stirred at 0 °C for a further 30 min and then warmed to rt and stirred for 2 h. The solution was concentrated *in* vacuo at 0 °C to remove the volatile components. The resulting brown oil was dissolved in anhydrous THF (2 mL) and cooled to 0 °C. A solution of (3-methylisoxazol-5-yl)methanamine hydrochloride (**68**) (80 mg, 0.54 mmol, 1.0 eq) dissolved in anhydrous THF (3 mL), and anhydrous triethylamine (186 µL, 1.35 mmol, 2.5 eq) was added dropwise to the previous solution. The solution was stirred for 16 h at rt. After this time the reaction was quenched by the addition of saturated aqueous $NaHCO_3$ solution, and extracted with EtOAc (3 × 30 mL). The organic components were combined and washed with brine (30 mL), dried over $MgSO_4$, filtered, and concentrated *in vacuo*. The crude product was purified using silica gel column chromatography, eluting with 0–100% 50:8:1 CH_2Cl_2:EtOH:aqueous NH_4OH/CH_2Cl_2, to isolate the title compound as a colorless oil (40 mg, 35%); R_f 0.37 (100:8:1 CH_2Cl_2:EtOH:aqueous NH_4OH); $\tilde{\nu}_{max}$ (thin film)/cm^{-1} 3274 (N-H, s br), 2974 (C-H, m), 1675 (C=O, s), 1641 (C=C, s), 1609 (C=C, m); ^{1}H NMR (400 MHz, $CDCl_3$) δ_H

6.84 (1H, dt, J 15.4, 6.0, 3^{ii}-CH), 6.56 (1H, t, J 6.0, 7-NH), 6.01 (1H, s, 4-CH), 5.98 (1H, dt, J 15.4, 1.6, 2^{ii}-CH), 4.60 (2H, d, J 6.0, 6-CH_2), 3.08 (2H, dd, J 6.0, 1.6, 4^{ii}-CH_2), 2.29 (3H, s, 1^{i}-CH_3), 2.27 (6H, s, 5^{ii}-CH_3); ^{13}C NMR (126 MHz, $CDCl_3$) δ_C 168.5 (1^{ii}-C), 165.5 (5-C), 160.2 (3-C), 142.7 (3^{ii}-C), 124.5 (2^{ii}-C), 103.2 (4-C), 60.5 (4^{ii}-C), 45.7 (5^{ii}-C), 35.3 (6-C), 11.5 (1^{i}-C); HRMS m/z (ESI$^+$) [Found: 224.1393, $C_{11}H_{18}O_2N_3$ ([M+H]$^+$) requires 224.1393]; LRMS m/z (ESI$^+$) 224 ([M+H]$^+$, 100%), 246 ([M+Na]$^+$, 20%); HPLC Retention time 7.4 min, 96.8%.

***N*-(3-Methylisoxazol-5-yl)prop-2-ynamide (19)**

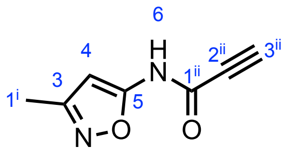

3-Methylisoxazol-5-amine (**59**) (100 mg, 1.02 mmol, 1.0 eq), DMAP (1.30 mg, 0.01 mmol, 0.01 eq), and *N,N'*-dicyclohexylcarbodiimide (273 mg, 1.33 mmol, 1.3 eq) were dissolved in anhydrous CH_2Cl_2 (5 mL). The solution was cooled to 0 °C. A solution of propiolic acid (82 µL, 1.33 mmol, 1.3 eq) in anhydrous CH_2Cl_2 (5 mL) was added dropwise to the previous solution. Upon addition, the reaction turned to a bright yellow suspension. The resulting suspension was slowly warmed to rt, stirred for 5 h, diluted with CH_2Cl_2 (20 mL) and filtered. The resulting filtrate was concentrated *in vacuo*, and purified using silica gel column chromatography, eluting with 10–30% EtOAc/petroleum ether, to isolate the title compound as a colorless solid (47 mg, 31%); R_f 0.53 (50% EtOAc/petroleum ether); m.p. 125 °C dec (from $CHCl_3$); $\tilde{\nu}_{max}$ (thin film)/cm^{-1} 3219 (C-H, m), 2109 (C≡C, m), 1684 (C=O, s); ^{1}H NMR (400 MHz, $CDCl_3$) δ_H 8.71 (1H, br s, 6-NH), 6.26 (1H, s, 4-CH), 3.06 (1H, s, 3^{ii}-CH), 2.28 (3H, s, 1^{i}-CH_3); ^{13}C NMR (101 MHz, $CDCl_3$) δ_C 161.8 (3-C), 158.7 (5-C), 147.1 (1^{ii}-C), 91.4 (4-C), 76.7 (3^{ii}-C), 76.2 (2^{ii}-C), 12.0 (1^{i}-C); HRMS m/z (ESI$^+$) [Found 151.0503, $C_7H_7O_2N_2$ ([M+H]$^+$) requires 151.0502]; LRMS m/z (ESI$^+$) 151 ([M+H]$^+$, 100%); HPLC Retention time 6.1 min, 96.9%.

***N*-(3-Methylisoxazol-5-yl)but-2-ynamide (20)**

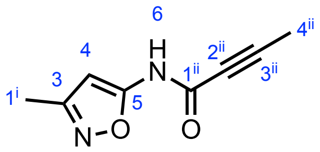

3-Methylisoxazol-5-amine (**59**) (150 mg, 1.53 mmol, 1.0 eq), 2-butynoic acid (167 mg, 1.99 mmol, 1.3 eq), and DMAP (18 mg, 0.15 mmol, 0.1 eq), were dissolved in CH_2Cl_2 (13 mL). In a separate flask *N,N'*-dicyclohexylcarbodiimide (410 mg, 1.99 mmol, 1.3 eq) was dissolved in CH_2Cl_2 (2 mL). The *N,N'*-dicyclohexylcarbodiimide solution was then added dropwise to the reaction mixture at 0 °C. The resulting solution was stirred at 0 °C for 15 min, and then warmed to rt to stir for 4 h. After this time the reaction mixture was filtered through Celite®, which was washed with EtOAc (20 mL). The filtrate was washed with H_2O (20 mL), and extracted with EtOAc (3 × 30 mL). The combined organic

components were washed with brine (30 mL), dried over $MgSO_4$, filtered, and concentrated *in vacuo*. The crude product was purified using silica gel chromatography, eluting with 10–40% EtOAc/petroleum ether, to isolate the title compound as a pale-yellow solid (40 mg, 16%); R_f 0.32 (40% EtOAc/petroleum ether); m.p. 123–125 °C; $\tilde{\nu}_{max}$ (thin film)/cm^{-1} 3185 (C-H, w), 2925 (C-H, m), 2248 (C≡C, m), 1647 (C=O, m), 1292 (C-N, s); ^{1}H NMR (400 MHz, $CDCl_3$) δ_H 8.66 (1H, s, 6-N*H*), 6.22 (1H, s, 4-C*H*), 2.27 (3H, s, 1^i-CH_3), 2.02 (3H, s, 4^{ii}-CH_3); ^{13}C NMR (101 MHz, $CDCl_3$) δ_C 161.6 (3-C), 159.4 (5-C), 148.5 (1^{ii}-C), 90.8 (4-C), 87.6 (2^{ii}-C), 74.2 (3^{ii}-C), 12.0 (1^i-C), 4.0 (4^{ii}-C); HRMS *m/z* (ESI$^+$) [Found: 165.0660, $C_8H_9O_2N_2$ ([M+H]$^+$) requires 165.0659]; LRMS *m/z* (ESI$^+$) 165 ([M+H]$^+$, 100%); HPLC Retention time 4.8 min, 98.9%.

2-Chloro-*N*-(3-methylisoxazol-5-yl)acetamide (21)

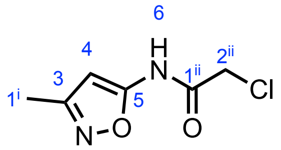

Chloroacetyl chloride (81 µL, 1.02 mmol, 1.0 eq) was added dropwise to a suspension of 3-methylisoxazol-5-amine (**59**) (100 mg, 1.02 mmol, 1.0 eq) and K_2CO_3 (563 mg, 4.08 mmol, 4.0 eq), in anhydrous CH_2Cl_2 (10 mL) at 0 °C, and the mixture stirred for 16 h at rt. The reaction was quenched by the addition of H_2O (20 mL), extracted with CH_2Cl_2 (3 × 20 mL), and concentrated *in vacuo*. The crude product was purified using silica gel column chromatography, eluting with 20–50% EtOAc/petroleum ether, to isolate the title compound as a colorless solid (81 mg, 46%); R_f 0.58 (50% EtOAc/petroleum ether); m.p. 110–111 °C (from $CHCl_3$); $\tilde{\nu}_{max}$ (thin film)/cm^{-1} 3296 (N-H, m), 2991 (C-H, w), 1702 (C=O, s), 1624 (N-H, m), 1404 (C-H, m), 790 (C-Cl, m); ^{1}H NMR (500 MHz, $CDCl_3$) δ_H 9.25 (1H, br s, 6-N*H*), 6.27 (1H, s, 4-C*H*), 4.22 (2H, s, 2^{ii}-CH_2), 2.28 (3H, s, 1^i-CH_3); ^{13}C NMR (126 MHz, $CDCl_3$) δ_C 162.5 (1^{ii}-C), 161.8 (3-C), 159.1 (5-C), 90.5 (4-C), 42.4 (2^i-C), 12.0 (1^i-C); HRMS *m/z* (ESI$^-$) [Found: 173.0120, $C_6H_6O_2N_2{}^{35}Cl^-$ ([M-H]$^-$) requires 173.0123, Found: 175.0090 $C_6H_6O_2N_2{}^{37}Cl$ ([M−H]$^-$) requires 175.0094]; LRMS *m/z* (ESI$^-$) 173 ([^{35}M-H]$^-$, 100%), 175 ([^{37}M−H]$^-$, 33%); HPLC Retention time 5.5 min, >99.0%.

2-Chloro-*N*-(3-methyl-4-phenylisoxazol-5-yl)acetamide (22)

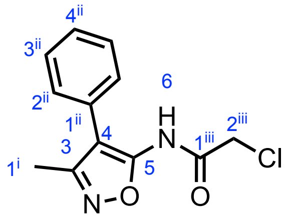

Chloroacetyl chloride (50 µL, 0.63 mmol, 1.1 eq) was added dropwise to a solution of 3-methyl-4-phenylisoxazol-5-amine (**63**) (100 mg, 0.57 mmol, 1.0 eq) and pyridine (51 µL, 0.63 mmol,

1.1 eq) in anhydrous CH_2Cl_2 (5 mL) at 0 °C. The reaction mixture was stirred at 0 °C for 1 h, and a further 2 h at rt. The reaction was then quenched by the addition of H_2O (20 mL), and extracted with CH_2Cl_2 (3 × 20 mL). The organic extracts were washed with brine (20 mL), dried over Na_2SO_4, filtered, and concentrated *in vacuo.* The crude orange solid was purified using silica gel column chromatography, eluting with 20–50% Et_2O/petroleum ether, to isolate the title compound as a colorless solid (34 mg, 24%); R_f 0.21 (50% Et_2O/petroleum ether); m.p. 77–78 °C (from $CHCl_3$); $\tilde{\nu}_{max}$ (thin film)/cm^{-1} 3214 (N-H, w), 1701 (C=O, m); ^{1}H NMR (500 MHz, $CDCl_3$) δ_H 8.48 (1H, br s, 6-N*H*), 7.46–7.43 (2H, m, 3^{ii}-C*H*), 7.40–7.36 (1H, m, 4^{ii}-C*H*), 7.29–7.27 (2H, m, 2^{ii}-C*H*), 4.15 (2H, s, 2^{iii}-C*H₂*), 2.31 (3H, s, 1^{i}-C*H₃*); ^{13}C NMR (126 MHz, $CDCl_3$) δ_C 163.9 (1^{iii}-C), 160.4 (3-C), 154.9 (5-C), 129.2 (3^{ii}-C), 128.7 (1^{ii}-C), 128.7 (2^{ii}-C), 128.4 (4^{ii}-C), 110.0 (4-C), 42.4 (2^{iii}-C), 11.5 (1^{i}-C); HRMS *m/z* (ESI$^+$) [Found: 251.0583, $C_{12}H_{12}O_2N_2Cl$ ([M+H]$^+$) requires 251.0582]; LRMS *m/z* (ESI$^+$) 251 ([^{35}M+H]$^+$, 100%), 253 ([^{37}M+H]$^+$, 33%); HPLC Retention time 7.5 min, >99.0%.

***N*-(3-Methylisoxazol-5-yl)ethenesulfonamide (23)**

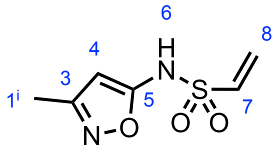

2-Chloroethanesulfonyl chloride (176 µL, 1.68 mmol, 1.1 eq) and anhydrous triethylamine (466 µL, 3.36 mmol, 2.2 eq) were added dropwise to a solution of 3-methylisoxazol-5-amine (**59**) (150 mg, 1.53 mmol, 1.0 eq) in anhydrous CH_2Cl_2 (15 mL), at −15 °C. The resulting solution was slowly warmed to 0 °C, and stirred for 3 h. The reaction was quenched by the addition of aqueous HCl (1.0 M, 3.0 mL), extracted with CH_2Cl_2 (3 × 20 mL), washed with H_2O (30 mL), and brine (30 mL), dried over $MgSO_4$, filtered, and concentrated *in vacuo*. The crude product was purified using silica gel column chromatography, eluting with 0–10% EtOAc/CH_2Cl_2 and 0.1% AcOH, to isolate the title compound as a yellow oil (10 mg, 4%); R_f 0.11 (10% EtOAc/CH_2Cl_2 + 0.1% AcOH); $\tilde{\nu}_{max}$ (thin film)/cm^{-1} 3057 (C-H, m), 1615 (N-H, m), 1352 (S=O, m); ^{1}H NMR (500 MHz, $CDCl_3$) δ_H 9.10 (1H, s, 6-N*H*), 6.60 (1H, dd, *J* 16.5, 9.9, 7-C*H*), 6.38 (1H, dd, *J* 16.5, 0.6, 8-CH_xH$_y$), 6.07 (1H, dd, *J* 9.9, 0.6, CH$_x$$H_y$), 5.77 (1H, s, 4-C*H*), 2.28 (3H, s, 1^{i}-C*H₃*); ^{13}C NMR (126 MHz, $CDCl_3$) δ_C 162.0 (3-C), 160.1 (5-C), 134.6 (7-C), 129.5 (8-C), 89.6 (4-C), 11.9 (1^{i}-C); LRMS *m/z* (ESI$^+$) 189 ([M+H]$^+$, 100%); HRMS *m/z* (ESI$^+$) [Found 189.0328, $C_6H_9O_3N_2{}^{32}S$ ([M+H]$^+$) requires 189.0328]; HPLC Retention time 5.5 min, 96.0%.

***N*-(3-Methyl-isoxazol-5-yl)propanamide (24)**

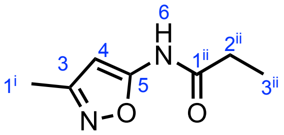

Propionyl chloride (196 µL, 2.24 mmol, 1.1 eq) was added dropwise to a solution of 3-methyl-isoxazol-5-amine (**59**) (200 mg, 2.04 mmol, 1.0 eq) and pyridine (182 µL, 2.24 mmol, 1.1 eq) in anhydrous CH_2Cl_2 (20 mL) at 0 °C. The reaction mixture was stirred at rt for 2.5 h, after which another portion of propionyl chloride (53 µL, 0.612 mmol, 0.3 eq) and pyridine (33 µL, 0.408 mmol, 0.2 eq) were added dropwise. The reaction mixture was stirred for a further 1 h, before being quenched by the addition of aqueous HCl (1.0 M, 20 mL), and extracted with CH_2Cl_2 (3 × 30 mL). The organic extracts were combined, washed with H_2O (30 mL) and brine (30 mL), dried over Na_2SO_4, filtered, and concentrated *in vacuo*. The crude product was purified using silica gel column chromatography, eluting with 10–30% EtOAc/ petroleum ether, to isolate the title compound as a colorless solid (247 mg, 79%); R_f 0.58 (100% EtOAc); m.p. 90–92 °C (from $CHCl_3$); $\tilde{\nu}_{max}$ (thin film)/cm^{-1} 3203 (N-H, m), 3025 (C-H, m), 1690 (C=O, s); ^{1}H NMR (500 MHz, $CDCl_3$) δ_H 8.25 (1H, br s, 6-N*H*), 6.21 (1H, s, 4-C*H*), 2.46 (2H, q, *J* 7.5, 2^{ii}-C*H_2*), 2.26 (3H, s, 1^{i}-C*H_3*), 1.25 (3H, t, *J* 7.5, 3^{ii}-C*H_2*); ^{13}C NMR (126 MHz, $CDCl_3$) δ_C 169.9 (1^{ii}-C), 161.8 (3-C), 160.0 (5-C), 89.6 (4-C), 30.0 (2^{ii}-C), 12.0 (1^{i}-C), 9.2 (3^{ii}-C); HRMS *m/z* (ESI$^+$) [Found: 177.0635, $C_7H_{10}O_2N_2{}^{23}Na$ ([M+Na]$^+$) requires 177.0634]; LRMS *m/z* (ESI$^+$) 155 ([M+H]$^+$, 56%), 177 ([M+Na]$^+$, 100%); HPLC Retention time 6.0 min, >99.0%.

4-(Dimethylamino)-*N*-(3-methyl-4-phenylisoxazol-5-yl)butanamide (25)

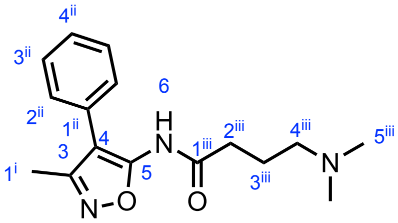

Using a procedure modified from Chew *et al.*,[16] oxalyl chloride (284 µL, 3.36 mmol, 1.95 eq) was added dropwise to a suspension of 4-(dimethylamino)butyric acid hydrochloride (577 mg, 3.44 mmol, 2.0 eq) in anhydrous THF (3.5 mL), and anhydrous DMF (2 drops) at 0 °C. The suspension was stirred at 0 °C for a further 30 min and then warmed to rt and stirred for a further 30 min. The solution was then cooled to 0 °C. 3-Methyl-4-phenylisoxazol-5-amine (**63**) (300 mg, 1.72 mmol, 1.0 eq) in anhydrous *N*-methyl-2-pyrrolidone (2 mL) was added dropwise at 0 °C, and the reaction mixture stirred for 20 h at 5 °C. The reaction was quenched by the addition of aqueous HCl (1.0 M, 30 mL), and washed with EtOAc (30 mL). The acidic aqueous extract was basified with aqueous NaOH (2.0 M) until pH 11 was attained, extracted with EtOAc (3 × 30 mL), washed with brine (20 mL), dried over Na_2SO_4, filtered, and concentrated *in vacuo*. The crude product was purified using silica gel column chromatography, eluting with 0–60% 12:2:1 EtOAc:EtOH:NH_4OH/EtOAc, to isolate the title

compound as a pale-yellow oil (295 mg, 60%); R_f 0.20 (50% 12:2:1 EtOAc:EtOH:NH_4OH/EtOAc); $\tilde{\nu}_{max}$ (thin film)/cm^{-1} 3197 (N-H, w), 3049 (C-H, m), 1661 (C=O, s); ^{1}H NMR (600 MHz, $CDCl_3$) δ_H 7.41–7.38 (2H, m, 3^{ii}-C*H*), 7.33–7.30 (1H, m, 4^{ii}-C*H*), 7.29–7.27 (2H, m, 2^{ii}-C*H*), 2.52–2.48 (2H, m, 2^{iii}-CH_2), 2.40 (2H, t, *J* 5.7, 4^{iii}-CH_2), 2.25 (3H, s, 1^{i}-CH_3), 2.04 (6H, s, 5^{iii}-CH_3), 1.80–1.76 (2H, m, 3^{ii}-CH_2); ^{13}C NMR (151 MHz, $CDCl_3$) δ_C 171.7 (1^{iii}-C), 160.1 (3-C), 157.8 (5-C), 130.0 (1^{ii}-C), 129.0 (2^{ii}-C), 128.8 (3^{ii}-C), 127.7 (4^{ii}-C), 108.0 (4-C), 59.6 (4^{iii}-C), 44.5 (5^{iii}-C), 37.2 (2^{iii}-C), 22.0 (3^{iii}-C), 11.5 (1^{i}-C); HRMS *m/z* (ESI$^+$) [Found: 288.1707, $C_{16}H_{22}O_2N_3$ ([M+H]$^+$) requires 288.1707]; LRMS *m/z* (ESI$^+$) 288 ([M+H]$^+$, 100%); HPLC Retention time 6.1 min, >99.0%.

2-(4-Iodophenyl)-3-oxobutanenitrile (32)

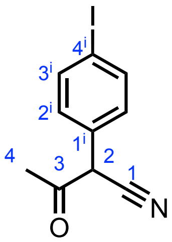

Using a procedure modified from Smith *et al.*,[9] sodium hydride (60% dispersion in mineral oil, 757 mg, 18.9 mmol, 1.15 eq) was added portion-wise to a solution of 4-iodophenylacetonitrile (**31**) (4.00 g, 16.5 mmol, 1.0 eq), and anhydrous DMF (5.5 mL) in anhydrous THF (49.5 mL) at 0 °C. The resulting dark purple suspension was stirred for 30 min at 0 °C before the dropwise addition of anhydrous EtOAc (6.44 mL, 65.8 mmol, 4.0 eq) at 0 °C. The suspension was stirred for 4 h at rt, after which time an additional portion of anhydrous EtOAc (6.44 mL, 65.8 mmol, 4.0 eq) was added dropwise. The reaction was stirred for a further 50 min and then quenched at 0 °C by the addition of aqueous HCl (1.0 M, 30 mL), and extracted with EtOAc (3 × 100 mL). The organic components were combined, washed with aqueous LiCl solution (0.5 M, 3 × 30 mL), and brine (50 mL), dried over Na_2SO_4, filtered, and concentrated *in vacuo*. The crude orange solid was purified using silica gel column chromatography, eluting with 10–50% EtOAc/petroleum ether, to isolate the title compound as a yellow solid (4.06 g, 86%); R_f 0.35 (40% EtOAc/petroleum ether); m.p. 113–115 °C (from $CHCl_3$); $\tilde{\nu}_{max}$ (thin film)/cm^{-1} 2215 (C≡N, s), 1678 (C=O, m); ^{1}H NMR (400 MHz, methanol-D_4) δ_H 7.68–7.64 (2H, m, 3^{i}-C*H*), 7.48–7.44 (2H, m, 2^{ii}-C*H*), 4.85 (1H, s, 2-C*H*), 2.35 (3H, s, 4-CH_3); HRMS *m/z* (ESI$^-$) [Found: 283.9575, $C_{10}H_7ONI$ ([M−H]$^-$) requires 283.9578]; LRMS *m/z* (ESI$^-$) 284 ([M−H]$^-$, 100%); HPLC Retention time 10.1 min, >99.0%.

4-(4-Iodophenyl)-3-methylisoxazol-5-amine (33)

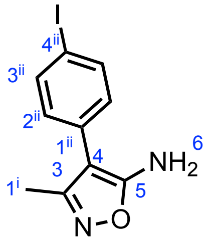

Using a procedure modified from Krasavin *et al.*,[13] aqueous Na_2CO_3 (10% *w/v*, 5 mL), and hydroxylamine hydrochloride (975 mg, 14.0 mmol, 2.0 eq) were added to a solution of 2-(4-iodophenyl)-3-oxobutanenitrile (**32**) (2.00 g, 7.02 mmol, 1.0 eq) dissolved in EtOH (20 mL). The solution was heated at 60 °C for 75 min, then cooled and concentrated *in vacuo*. The crude material was dissolved in H_2O (30 mL) and extracted with EtOAc (3 × 50 mL). The organic components were combined, washed with brine (30 mL), dried over Na_2SO_4, filtered, and concentrated *in vacuo* to isolate the title compound as a pale-yellow solid. No further purification was required (2.10 g, 99%); R_f 0.55 (50% EtOAc/petroleum ether); m.p. 106–108 °C (from $CHCl_3$); $\tilde{\nu}_{max}$ (thin film)/cm^{-1} 3310 (N-H, m), 3174 (C-H, m), 1637 (C-N, s), 1506 (C-C, m); ^{1}H NMR (400 MHz, $CDCl_3$) δ_H 7.76–7.72 (2H, m, 3^{ii}-C*H*), 7.05–7.01 (2H, m, 2^{ii}-C*H*), 4.57 (1H, br s, 6-NH_2), 2.20 (3H, s, 1^{i}-CH_3); ^{13}C NMR (101 MHz, $CDCl_3$) δ_C 164.7 (5-C), 159.6 (3-C), 138.4 (3^{ii}-C), 130.6 (1^{ii}-C), 130.1 (2^{ii}-C), 94.4 (4-C), 91.9 (4^{ii}-C), 11.3 (1^{i}-C); HRMS *m/z* (ESI$^+$) [Found: 300.9833, $C_{10}H_{10}ON_2I$ ([M+H]$^+$) requires 300.9832]; LRMS *m/z* (ESI$^+$) 301 ([M+H]$^+$, 100%); HPLC Retention time 9.8 min, >99.0%.

3-Methyl-4-{4-[(trimethylsilyl)ethynyl]phenyl}isoxazol-5-amine (34)

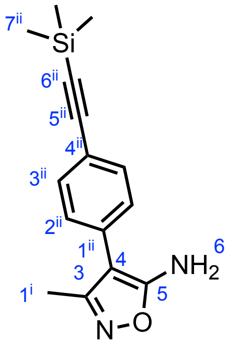

4-(4-Iodophenyl)-3-methylisoxazol-5-amine (**33**) (1.00 g, 3.33 mmol, 1.0 eq), $Pd(PPh_3)_2Cl_2$ (140 mg, 0.20 mmol, 0.06 mmol), and CuI (76 mg, 0.40 mmol, 0.12 eq) were added to a microwave vial, and the vial sealed and purged with Ar. Degassed and anhydrous triethylamine (4.0 mL) and DMF (1.0 mL) were added, followed by ethynyltrimethylsilane (1.39 mL, 10.0 mmol, 3.0 eq). The reaction mixture was stirred for 1 h at rt, after which a constant stream of N_2 was applied to remove the triethylamine, and the residue filtered through a pad of Celite®, which was washed with Et_2O (50 mL). The filtrate was concentrated *in vacuo*, and the crude brown oil purified using silica gel column chromatography, eluting with 6–35% EtOAc/petroleum ether, to isolate the title compound as a pale-orange solid

(666 mg, 74%); R_f 0.63 (50% Et_2O/toluene); m.p. 134–135 °C (from EtOAc); $\tilde{\nu}_{max}$ (thin film)/cm^{-1} 3299 (N-H, w), 2155 (C≡C), 1641 (C-N, s), 1520 (C-C, m); ^{1}H NMR (600 MHz, $CDCl_3$) δ_H 7.51–7.49 (2H, m, 3^{ii}-C*H*), 7.23–7.21 (2H, m, 2^{ii}-C*H*), 4.59 (1H, br s, 6-NH_2), 2.22 (3H, s, 1^{i}-CH_3), 0.26 (9H, s, 7^{ii}-CH_3); ^{13}C NMR (151 MHz, $CDCl_3$) δ_C 164.8 (5-C), 159.7 (3-C), 132.8 (3^{ii}-C), 131.4 (1^{ii}-C), 127.8 (2^{ii}-C), 121.5 (4^{ii}-C), 104.8 (5^{ii}-C), 95.0 (6^{ii}-C), 94.9 (4-C), 11.4 (1^{i}-C), 0.1 (7^{ii}-C); HRMS *m/z* (ESI$^+$) [Found: 271.1262, $C_{15}H_{19}ON_2Si$ ([M+H]$^+$) requires 271.1261]; LRMS *m/z* (ESI$^+$) 271 ([M+H]$^+$, 100%); HPLC Retention time 11.9 min, >99.0%.

4-(4-Ethynylphenyl)-3-methylisoxazol-5-amine (35)

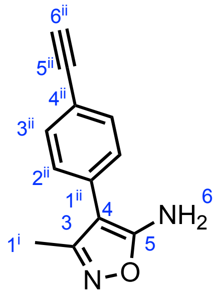

To a solution of 3-ethyl-4-{4-[(trimethylsilyl)ethynyl]phenyl}isoxazol-5-amine (**34**) (550 mg, 2.03 mmol, 1.0 eq) in MeOH (20 mL) was added K_2CO_3 (309 mg, 2.24 mmol, 1.1 eq) portion-wise, and the mixture stirred at rt for 50 min, and then concentrated *in vacuo*. The resulting crude brown solid was purified using silica gel column chromatography, eluting with 5–30% EtOAc/petroleum ether, to isolate the title compound as a colorless solid (372 mg, 92%); R_f 0.24 (25% EtOAc/petroleum ether); m.p. 137–138 °C (from EtOAc); $\tilde{\nu}_{max}$ (thin film)/cm^{-1} 3307 (N-H, m), 3255 (C-H, s), 1638 (C-N, s), 1511 (C-C, m); ^{1}H NMR (600 MHz, $CDCl_3$) δ_H 7.54–7.52 (2H, m, 3^{ii}-C*H*), 7.26–7.24 (2H, m, 2^{ii}-C*H*), 4.61 (1H, br s, 6-NH_2), 3.11 (1H, s, 6^{ii}-C*H*), 2.22 (3H, s, 1^{i}-CH_3); ^{13}C NMR (151 MHz, $CDCl_3$) δ_C 164.9 (5-C), 159.7 (3-C), 133.0 (3^{ii}-C), 131.8 (1^{ii}-C), 127.9 (2^{ii}-C), 120.4 (4^{ii}-C), 94.8 (4-C), 83.4 (5^{ii}-C), 77.8 (6^{ii}-C), 11.4 (1^{i}-C); HRMS *m/z* (ESI$^+$) [Found: 199.0866, $C_{12}H_{11}ON_2$ ([M+H]$^+$) requires 199.0866]; LRMS *m/z* (ESI$^+$) 199 ([M+H]$^+$, 100%); HPLC Retention time 8.8 min, >99.0%.

(*E*)-4-Bromo/Chloro-*N*-[4-(4-ethynylphenyl)-3-methylisoxazol-5-yl]but-2-enamide (40)

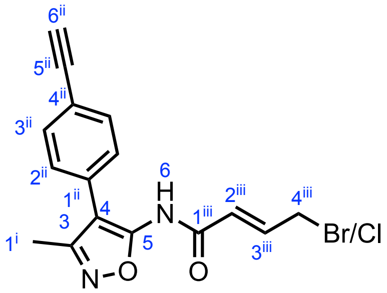

Oxalyl chloride (238 µL, 2.82 mmol, 1.5 eq) was added dropwise to a solution of (*E*)-4-bromocrotonic acid (**64**) (403 mg, 2.44 mmol, 1.3 eq) in anhydrous CH_2Cl_2 (5 mL), and anhydrous DMF (1 drops) at

0 °C. The solution was stirred at 0 °C for a further 30 min and then warmed to rt and stirred for 15 h. The solution was then concentrated *in vacuo*, and the resulting pale-brown oil redissolved in anhydrous CH_2Cl_2 (5.6 mL).

The acyl chloride solution was then added dropwise to a solution of 4-(4-ethynylphenyl)-3-methylisoxazol-5-amine (**35**) (372 mg, 1.88 mmol, 1.0 eq) and pyridine (228 µL, 2.85 mmol, 1.3 eq) in anhydrous CH_2Cl_2 (9 mL), at 0 °C. After 5 h the reaction was quenched by the addition of HCl (1.0 M, 20 mL), and extracted with CH_2Cl_2 (3 × 50 mL). The organic extracts were combined, washed with saturated aqueous $NaHCO_3$ solution (20 mL), and brine (20 mL), dried over Na_2SO_4, filtered, and concentrated *in vacuo*. The crude product was purified using silica gel column chromatography, eluting with 25–60% Et_2O/petroleum ether, to isolate a mixture of the bromo- or chloro-substituted title compound as a pale-yellow solid (235 mg, 37% on a 20% Cl : 80% Br ratio determined using ^{1}H NMR); R_f 0.25 (50% Et_2O/petroleum ether); ^{1}H NMR (400 MHz, $CDCl_3$) δ_H 7.61 (1H, s, 6-N*H*), 7.56–7.53 (2H, m, 3^{ii}-C*H*), 7.26–7.23 (2H, m, 2^{ii}-C*H*), 7.08–7.00 (2H, m, 3^{iii}-C*H*), 6.27 (0.2H, d, *J* 15.0, 2^{iii}-C$H_{(Cl)}$), 6.20 (0.8H, d, *J* 14.8, 2^{iii}-C$H_{(Br)}$), 4.18 (0.4H, dd, *J* 5.5, 1.7, 4^{iii}-C$H_{2(Cl)}$), 4.00 (1.6H, dd, *J* 7.1, 1.3, 4^{iii}-C$H_{2(Br)}$), 3.13 (1H, s, 6^{ii}-C*H*), 2.30 (3H, s, 1^{i}-CH_3); LRMS *m/z* (ESI$^+$) 301, 303 ([M+H]$^+$; ^{35}Cl, 100%; ^{37}Cl, 30%), 345, 347 ([M+H]$^+$; ^{79}Br, 100%; ^{81}Br, 95%).

(*E*)-4-(Dimethylamino)-*N*-[4-(4-ethynylphenyl)-3-methylisoxazol-5-yl]but-2-enamide (28)

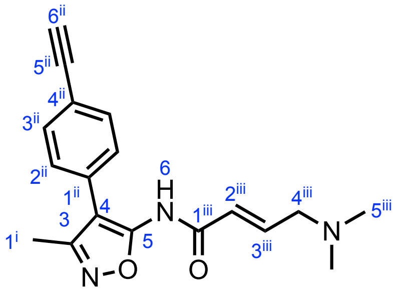

Step 1: Sodium iodide (154 mg, 1.03 mmol, 1.5 eq) was added to a solution of (*E*)-4-bromo/chloro-*N*-[4-(4-ethynylphenyl)-3-methylisoxazol-5-yl]but-2-enamide (**40**) (235 mg, 0.68 mmol, 1.0 eq) in acetone (4 mL). The reaction mixture was heated at 50 °C for 1 h, after which it was diluted with acetone (10 mL), filtered, and concentrated *in vacuo* to afford the title compound as a yellow solid. This intermediate was used in the next step without further purification. R_f 0.25 (50% Et_2O/petroleum ether); LRMS *m/z* (ESI$^+$) 393 ([M+H]$^+$, 100%).

Step 2: The crude (*E*)-*N*-[4-(4-ethynylphenyl)-3-methylisoxazol-5-yl]-4-iodobut-2-enamide from *step 1* was dissolved in anhydrous DMF (4 mL), and K_2CO_3 (189 mg, 1.37 mmol, 2.0 eq) was added. The suspension was cooled to 0 °C and dimethylamine solution (2.0 M in THF, 376 µL, 0.75 mmol, 1.1 eq) was added dropwise. After stirring for 1 h at 0 °C a further portion of dimethylamine solution (2.0 M in THF, 68 µL, 0.14 mmol, 0.2 eq) was added dropwise. The reaction mixture was stirred for a further 30 min before it was quenched by the addition of aqueous HCl (1.0 M, 30 mL), and washed with EtOAc

(30 mL). The acidic aqueous extract was basified with aqueous NaOH (2.0 M) until pH 11 was attained, extracted with EtOAc (3 × 30 mL), washed with brine (30 mL), dried over Na_2SO_4, filtered, and concentrated *in vacuo*. The crude product was purified using silica gel column chromatography, eluting with 0–50% 12:2:1 EtOAc:EtOH:NH_4OH/EtOAc, to isolate the title compound as a pale-yellow solid (60 mg, 28% over two steps); R_f 0.33 (10% MeOH/CH_2Cl_2); m.p. 132–134 °C (from $CHCl_3$); $\tilde{\nu}_{max}$ (thin film)/cm^{-1} 3276 (N-H, w), 3184 (C-H, w), 1670 (C=O, m), 1646 (C=C, m); ^1H NMR (500 MHz, $CDCl_3$) δ_H 7.54–7.52 (2H, m, 3^{ii}-C*H*), 7.26–7.24 (2H, m, 2^{ii}-C*H*), 6.96 (1H, dt, *J* 15.3, 5.8, 3^{iii}-C*H*), 6.12 (1H, dt, *J* 15.3, 1.6, 2^{iii}-C*H*), 3.12 (1H, s, 6^{ii}-C*H*), 3.06 (2H, dd, *J* 5.8, 1.6, 4^{iii}-CH_2), 2.29 (3H, s, 1^{i}-C*H*), 2.22 (6H, s, 5^{iii}-CH_3); ^{13}C NMR (126 MHz, $CDCl_3$) δ_C 163.3 (1^{iii}-C), 160.2 (3-C), 156.5 (5-C), 146.2 (3^{iii}-C), 132.8 (3^{ii}-C), 129.8 (1^{ii}-C), 128.7 (2^{ii}-C), 122.9 (2^{iii}-C), 121.9 (4^{iii}-C), 108.5 (4-C), 83.2 (5^{ii}-C), 78.4 (6^{ii}-C), 60.4 (4^{iii}-C), 45.7 (5^{iii}-C), 11.6 (1^{i}-C); HRMS *m/z* (ESI^+) [Found: 310.1550, $C_{18}H_{20}O_2N_3$ ($[M+H]^+$) requires 310.1550]; LRMS *m/z* (ESI^+) 310 ($[M+H]^+$, 100%); HPLC Retention time 6.7 min, >99.0%.

2-(4-Prop-2-ynoxyphenyl)acetonitrile (37)

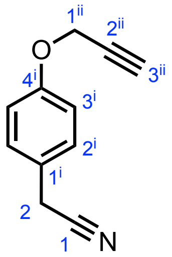

To a solution of 4-hydroxyphenylacetonitrile (**36**) (2.50 g, 18.8 mmol, 1.0 eq) in anhydrous DMF (50 mL) was added K_2CO_3 (10.4 g, 75.1 mmol, 4.0 eq). The suspension was stirred at rt for 30 min before being cooled to 0 °C. Propargyl bromide solution (80 % *w/v* in toluene, 3.14 mL, 28.2 mmol, 1.5 eq) was then added dropwise to the suspension at 0 °C, and stirred for 15 min before being raised to rt. After 2.5 h the reaction was concentrated *in vacuo*, resuspended in aqueous NaOH (1.0 M, 50 mL), and extracted with EtOAc (3 × 75 mL). The organic extracts were combined, washed with aqueous LiCl solution (0.5 M, 3 × 50 mL), and brine (50 mL), dried over Na_2SO_4, filtered, and concentrated *in vacuo* to isolate the title compound as a yellow solid. No further purification was required (3.21 g, 99%); R_f 0.62 (40% EtOAc/petroleum ether); m.p. 47–48 °C (from $CHCl_3$); $\tilde{\nu}_{max}$ (thin film)/cm^{-1} 3289 (C-H, m), 2251 (C≡N, w), 2121 (C≡C, w); ^1H NMR (600 MHz, $CDCl_3$) δ_H 7.17–7.13 (2H, m, 2^{i}-C*H*), 6.89–6.86 (2H, m, 3^{i}-C*H*), 4.59 (2H, d, *J* 2.4, 1^{ii}-CH_2), 3.58 (2H, s, 2-CH_2), 2.43 (1H, t, *J* 2.4, 3^{ii}-C*H*); ^{13}C NMR (151 MHz, $CDCl_3$) δ_C 157.3 (4^{i}-C), 129.2 (2^{i}-C), 122.9 (1^{i}-C), 118.2 (1-C), 115.6 (3^{i}-C), 78.3 (2^{ii}-C), 75.9 (3^{ii}-C), 55.9 (1^{ii}-C), 22.9 (2-C); LRMS *m/z* (ESI^-) 170 ($[M-H]^-$, 100%); HPLC Retention time 9.0 min, >99.0%.

3-Oxo-2-(4-prop-2-ynoxyphenyl)butanenitrile (38)

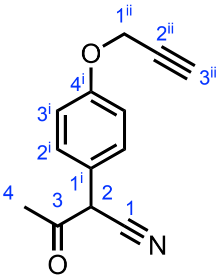

Using a procedure modified from Smith *et al.,*[9] sodium hydride (60% dispersion in mineral oil, 257 mg, 6.43 mmol, 1.1 eq) was added portion-wise to a solution of 2-(4-prop-2-ynoxyphenyl)acetonitrile (**37**) (1.00 g, 5.84 mmol, 1.0 eq), and anhydrous DMF (1.6 mL) in anhydrous THF (14.4 mL) at 0 °C. The suspension was stirred for 30 min at 0 °C before the dropwise addition of anhydrous EtOAc (4.57 mL, 46.7 mmol, 9.0 eq) at 0 °C. The resulting suspension was stirred for a further 3.5 h at rt. The reaction was quenched at 0 °C by the addition of aqueous HCl (1.0 M, 20 mL), and the THF removed *in vacuo*. The solution was extracted with EtOAc (3 × 30 mL) and the organic components combined, washed with H_2O (50 mL), and brine (50 mL), dried over Na_2SO4, filtered, and concentrated *in vacuo*. The crude product was purified using silica gel column chromatography, eluting with 30–80% Et_2O/petroleum ether, to isolate the title compound as a pale-yellow solid (1.14 g, 91%); R_f 0.31 (40% EtOAc/petroleum ether); m.p. 54–56 °C (from $CHCl_3$); $\tilde{\nu}_{max}$ (thin film)/cm^{-1} 3289 (C-H, m), 2208 (C≡N, w), 2123 (C≡C, w), 1728 (C=O, m); ^{1}H NMR (600 MHz, $CDCl_3$) δ_H 7.32–7.29 (2H, m, 2^i-C*H*), 7.03–7.00 (2H, m, 3^i-C*H*), 4.70 (2H, d, *J* 2.4, 1^{ii}-C*H₂*), 4.64 (1H, br s, 2-C*H*), 2.54 (1H, t, *J* 2.4, 3^{ii}-C*H*), 2.23 (3H, s, 4-C*H₃*); ^{13}C NMR (151 MHz, $CDCl_3$) δ_C 196.8 (3-C), 158.3 (4^i-C), 129.4 (2^i-C), 122.6 (1^i-C), 116.4 (1-C), 116.1 (3^i-C), 78.1 (2^{ii}-C), 76.2 (3^{ii}-C), 56.0 (1^{ii}-C), 50.7 (2-C), 27.0 (4-C); HRMS *m/z* (ESI$^+$) [Found: 212.0710, $C_{13}H_{10}O_2N$ ([M+H]$^+$) requires 212.0706]; LRMS *m/z* (ESI$^+$) 212 ([M+H]$^+$, 100%); HPLC Retention time 8.9 min, >99.0%.

3-Methyl-4-(4-prop-2-ynoxyphenyl)-isoxazol-5-amine (39)

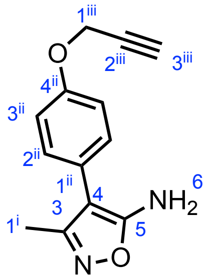

Using a procedure modified from Krasavin *et al.,*[13] aqueous Na_2CO_3 (10%, 3.0 mL), and hydroxylamine hydrochloride (525 mg, 7.55 mmol, 2.0 eq) were added to a solution of 3-oxo-2-(4-prop-2-ynoxyphenyl)butanenitrile (**38**) (805 mg, 3.78 mmol, 1.0 eq) dissolved in EtOH (12 mL). The solution was heated at 80 °C for 1 h, then cooled and concentrated *in vacuo*. The crude material was dissolved in H_2O (30 mL) and extracted with EtOAc (3 × 30 mL). The organic components were combined,

washed with brine (30 mL), dried over Na_2SO_4, filtered, and concentrated *in vacuo*. The crude product was purified using silica gel column chromatography, eluting with 0–5% EtOAc/CH_2Cl_2, to isolate the title compound as a yellow solid (711 mg, 83%); R_f 0.51 (50% EtOAc/petroleum ether); m.p. 57–59 °C (from EtOAc); $\tilde{\nu}_{max}$ (thin film)/cm^{-1} 3359 (N-H, w), 2121 (C≡C, w), 1642 (C-N, s), 1516 (C-C, m); ^{1}H NMR (500 MHz, $CDCl_3$) δ_H 7.24–7.21 (2H, m, 2^{ii}-C*H*), 7.06–7.03 (2H, m, 3^{ii}-C*H*), 4.72 (2H, d, *J* 2.4, 1^{iii}-CH_2), 4.44 (2H, br s, 6-NH_2), 2.54 (1H, t, *J* 2.4, 3^{iii}-C*H*), 2.20 (3H, s, 1^{i}-CH_3); ^{13}C NMR (126 MHz, $CDCl_3$) δ_C 164.6 (5-C), 160.0 (3-C), 156.6 (4^{ii}-C), 129.7 (2^{ii}-C), 124.1 (1^{ii}-C), 115.8 (3^{ii}-C), 95.0 (4-C), 78.6 (2^{iii}-C), 75.9 (3^{iii}-C), 56.0 (1^{iii}-C), 11.3 (1^{i}-C); HRMS *m/z* (ESI$^+$) [Found: 229.0973 $C_{13}H_{13}O_2N_2$ ([M+H]$^+$) requires 229.0983]; LRMS *m/z* (ESI$^+$) 229 ([M+H]$^+$, 100%); HPLC Retention time 8.5 min, >99.0%.

(*E*)-4-Bromo/Chloro-*N*-[3-methyl-4-(4-prop-2-ynoxyphenyl)-isoxazol-5-yl]but-2-enamide (71)

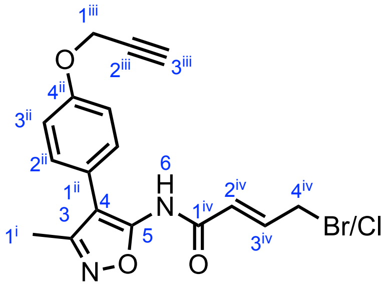

Oxalyl chloride (668 µL, 7.89 mmol, 3.0 eq) was added dropwise to a solution of (*E*)-4-bromocrotonic acid (**64**) (868 mg, 5.26 mmol, 1.6 eq) in anhydrous CH_2Cl_2 (5 mL), and anhydrous DMF (2 drops) at 0 °C. The solution was stirred at 0 °C for a further 15 min and then warmed to rt and stirred for 3 h. The solution was then concentrated *in vacuo*, and the pale-brown oil redissolved in anhydrous CH_2Cl_2 (2 mL).

The acyl chloride solution (1.1 mL) was then added dropwise to a solution of 3-methyl-4-(4-prop-2-ynoxyphenyl)-isoxazol-5-amine (**39**) (600 mg, 2.63 mmol, 1.0 eq) and pyridine (319 µL, 3.95 mmol, 1.5 eq) in anhydrous CH_2Cl_2 (13 mL), at 0 °C and stirred. After 2 h the reaction was quenched by the addition of aqueous HCl (1.0 M, 20 mL), and extracted with CH_2Cl_2 (3 × 50 mL). The organic extracts were combined, washed with saturated aqueous $NaHCO_3$ solution (20 mL), and brine (20 mL), dried over Na_2SO_4, filtered, and concentrated *in vacuo*. The crude product was purified using silica gel column chromatography, eluting with 40–80% Et_2O/petroleum ether, to isolate a mixture of bromo- or chloro-substituted title compound as a pale-brown oil (350 mg, 36%); R_f 0.19 (60% Et_2O/petroleum ether); ^{1}H NMR (400 MHz, $CDCl_3$) δ_H 8.52 (1H, br s, 6-N*H*), 7.20–7.16 (2H, m, 2^{ii}-C*H*), 7.00–6.90 (3H, m, 3^{ii}-C*H*, 3^{iv}-C*H*), 6.26 (0.3H, dt, *J* 15.0, 1.6, 2^{iv}-C$H_{(Cl)}$), 6.20 (0.7H, dt, *J* 15.0, 1.3, 2^{iv}-C$H_{(Br)}$), 4.68 (2H, d, *J* 2.3, 1^{iii}-CH_2), 4.15 (0.6H, dd, *J* 5.7, 1.6, 4^{iv}-C$H_{2(Cl)}$), 3.99 (1.4H, dd, *J* 7.2, 1.3, 4^{iv}-C$H_{2(Br)}$), 2.54 (1H, t, *J* 2.3, 3^{iii}-C*H*), 2.23 (3H, s, 1^{i}-CH_3); LRMS *m/z* (ESI$^+$) 331, 333 ([M+H]$^+$; ^{35}Cl, 100%; ^{37}Cl, 30%), 375, 377 ([M+H]$^+$; ^{79}Br, 100%; ^{81}Br, 97%).

(*E*)-4-(Dimethylamino)-*N*-[3-methyl-4-(4-prop-2-ynoxyphenyl)-isoxazol-5-yl]but-2-enamide (29)

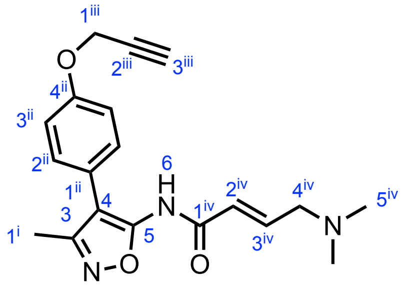

Method 1:

Step 1: Sodium iodide (120 mg, 0.800 mmol, 1.5 eq) was added to a solution of (*E*)-4-bromo/chloro-*N*-[3-methyl-4-(4-prop-2-ynoxyphenyl)-isoxazol-5-yl]but-2-enamide (**71**) (200 mg, 0.533 mmol, 1.0 eq) in acetone (5 mL). The reaction mixture was heated at 50 °C for 1 h, after which time it was diluted with acetone (10 mL), filtered, and concentrated *in vacuo* to isolate the title compound as a yellow oil. This intermediate was used in the next step without further purification; R_f 0.19 (60% Et_2O/petroleum ether); LRMS *m/z* (ESI^+) 423 ($[M+H]^+$, 100%).

Step 2: The crude (*E*)-4-iodo-*N*-[3-methyl-4-(4-prop-2-ynoxyphenyl)-isoxazol-5-yl]but-2-enamide from *step 1* was dissolved in anhydrous DMF (5 mL), and K_2CO_3 (147 mg, 1.07 mmol, 2.0 eq) was added. The suspension was cooled to 0 °C and dimethylamine solution (2.0 M in THF, 293 µL, 0.59 mmol, 1.1 eq) was added dropwise. After stirring for 1 h, the reaction was quenched by the addition of aqueous NaOH (1.0 M, 30 mL), and extracted with EtOAc (3 × 30 mL). The organic extracts were combined, washed with brine (2 × 30 mL), dried over Na_2SO_4, filtered, and concentrated *in vacuo*. The crude product was purified using silica gel column chromatography, eluting with 1– 12% EtOH/CH_2Cl_2, followed by another round of silica gel column chromatography (0–50% 12:2:1 EtOAc: EtOH: NH_4OH/EtOAc) to isolate the title compound as a pale-yellow oil (30 mg, 18% over two steps).

Method 2:

Using a procedure modified from Chew *et al.*,[16] oxalyl chloride (98 µL, 1.16 mmol, 1.95 eq) was added dropwise to a suspension of (*E*)-4-(dimethylamino)but-2-enoic acid hydrochloride (198 mg, 1.19 mmol, 2.0 eq) in anhydrous THF (3 mL), and anhydrous DMF (1 drop) at 0 °C. The suspension was stirred at 0 °C for a further 15 min and then warmed to rt and stirred for 80 min. The solution was then cooled to 0 °C. 3-Methyl-4-(4-prop-2-ynoxyphenyl)-isoxazol-5-amine (**39**) (136 mg, 0.60 mmol, 1.0 eq) in anhydrous *N*-methyl-2-pyrrolidone (1.2 mL) was added dropwise at 0 °C, and the reaction mixture stirred for 22 h at 5 °C. The reaction was quenched by the addition of aqueous HCl (1.0 M, 30 mL), and washed with EtOAc (30 mL). The acidic aqueous extract was basified with aqueous NaOH (2.0 M) until pH 11 was attained, extracted with EtOAc (3 × 30 mL), washed with brine (20 mL), dried over Na_2SO_4, filtered, and concentrated *in vacuo*. The crude product was purified using silica gel column chromatography, eluting with 0–50% 12:2:1 EtOAc:EtOH:NH_4OH/EtOAc, followed by

additional purification using semi-preparative HPLC (see general chemical methods) to isolate the title compound as the formic acid salt, which was then dissolved in aqueous NaOH (1.0 M), extracted with EtOAc (3 × 20 mL), and concentrated *in vacuo* to isolate the free base as a pale-yellow oil (75 mg, 37%); R_f 0.31 (50% 12:2:1 EtOAc:EtOH:NH_4OH/EtOAc); $\tilde{\nu}_{max}$ (thin film)/cm^{-1} 3263 (N-H, w), 3036 (C-H, w), 2122 (C≡C, w), 1673 (C=O, s); ^{1}H NMR (600 MHz, $CDCl_3$) δ_H 7.23–7.21 (2H, m, 2^{ii}-C*H*), 7.04–7.01 (2H, m, 3^{ii}-C*H*), 6.97 (1H, dt, *J* 15.4, 5.9, 3^{iv}-C*H*), 6.14 (1H, d, *J* 15.4, 2^{iv}-C*H*), 4.71 (2H, d, *J* 2.4, 1^{iii}-CH_2), 3.06 (2H, dd, *J* 5.9, 1.5, 4^{iv}-CH_2), 2.54 (1H, t, *J* 2.4, 3^{iii}-C*H*), 2.28 (3H, s, 1^{i}-CH_3), 2.22 (6H, s, 5^{iv}-CH_3); ^{13}C NMR (151 MHz, $CDCl_3$) δ_C 163.6 (1^{iv}-C), 160.4 (3-C), 157.5 (4^{ii}-C), 156.1 (5-C), 145.9 (3^{iv}-C), 130.1 (2^{ii}-C), 123.0 (2^{iv}-C), 122.1 (1^{ii}-C), 115.6 (3^{ii}-C), 108.7 (4-C), 78.4 (2^{iii}-C), 76.0 (3^{iii}-C), 60.4 (4^{iv}-C), 56.0 (1^{iii}-C), 45.6 (5^{iv}-C), 11.6 (1^{i}-C); HRMS *m/z* (ESI$^+$) [Found: 340.1656, $C_{19}H_{22}O_3N_3$ ([M+H]$^+$) requires 340.1656]; LRMS *m/z* (ESI$^+$) 340 ([M+H]$^+$, 100%); HPLC Retention time 6.6 min, 95.7%.

N-[3-Methyl-4-(4-prop-2-ynoxyphenyl)-isoxazol-5-yl]prop-2-enamide (30)

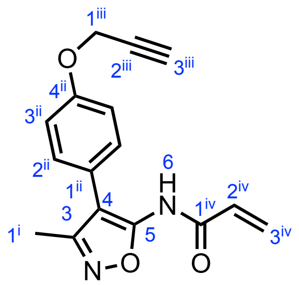

Acryloyl chloride (108 µL, 1.34 mmol, 1.3 eq) was added dropwise to a solution of 3-methyl-4-(4-prop-2-ynoxyphenyl)-isoxazol-5-amine (**39**) (235 mg, 1.03 mmol, 1.0 eq) and pyridine (125 µL, 1.55 mmol, 1.5 eq) in anhydrous CH_2Cl_2 (5 mL) at 0 °C. The reaction mixture was stirred at 0 °C for 90 min, after which a further portion of acryloyl chloride (108 µL, 1.34 mmol, 1.3 eq) was added dropwise at 0 °C. The reaction mixture was stirred for another 1 h before being quenched by the addition of aqueous HCl (1.0 M, 20 mL), and extracted with CH_2Cl_2 (3 × 30 mL). The organic extracts were combined, washed with H_2O (30 mL) and brine (30 mL), dried over Na_2SO_4, filtered, and concentrated *in vacuo*. The crude product was purified using silica gel column chromatography, eluting with 45–70% Et_2O/petroleum ether, to isolate the title compound as a yellow oil (52 mg, 18%); R_f 0.16 (60% Et_2O/petroleum ether); $\tilde{\nu}_{max}$ (thin film)/cm^{-1} 3284 (N-H, m), 3030 (C-H, w), 2118 (C≡C, w), 1683 (C=O, m), 1646 (C=C, m); ^{1}H NMR (600 MHz, $CDCl_3$) δ_H 8.21 (1H, br s, 6-N*H*), 7.21–7.17 (2H, m, 2^{ii}-C*H*), 7.01–6.97 (2H, m, 3^{ii}-C*H*), 6.37 (1H, dd, *J* 16.9, 1.0, 3^{iv}-CH_xH$_y$), 6.26 (1H, dd, *J* 16.9, 10.4, 2^{iv}-C*H*), 5.77 (1H, dd, *J* 10.4, 1.0, 3^{iv}-CH$_x$$H_y$), 4.68 (2H, d, *J* 2.3, 1^{iii}-CH_3), 2.53 (1H, t, *J* 2.3, 3^{iii}-C*H*), 2.24 (3H, s, 1^{i}-CH_3); ^{13}C NMR (151 MHz, $CDCl_3$) δ_C 163.8 (1^{iv}-C), 160.4 (3-C), 157.4 (4^{ii}-C), 155.9 (5-C), 130.4 (3^{iv}-C), 130.1 (2^{ii}-C), 128.8 (2^{iv}-C), 122.0 (1^{ii}-C), 115.4 (3^{ii}-C), 109.2 (4-C), 78.4 (2^{iii}-C), 75.9 (3^{iii}-C), 55.9 (1^{iii}-C), 11.5 (1^{i}-C); HRMS *m/z* (ESI$^+$) [Found: 283.1078, $C_{16}H_{15}O_3N_2$ ([M+H]$^+$) requires 283.1077]; LRMS *m/z* (ESI$^+$) 283 ([M+H]$^+$, 100%); HPLC Retention time 8.7 min, >99.0%.

(*E*)-4-Bromo/Chloro-*N*-(3-methoxy-4-(2-oxopyrrolidin-1-yl)phenyl)but-2-enamide (44)

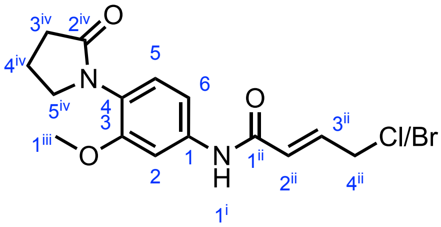

Using a procedure modified from Chew *et al.*,[16] oxalyl chloride (40 µL, 0.47 mmol, 1.95 eq) was added dropwise to a solution of (*E*)-4-bromocrotonic acid (80 mg, 0.48 mmol, 2.0 eq) in anhydrous THF (1.5 mL), and anhydrous DMF (1 drop) at 0 °C. The reaction mixture was stirred at 0 °C for 15 min and then warmed to rt and stirred for a further 30 min. The solution was then cooled to 0 °C, 1-(4-amino-2-methoxyphenyl)pyrrolidin-2-one (**43**) (50 mg, 0.24 mmol, 1.0 eq) in anhydrous *N*-methyl-2-pyrrolidone (1 mL) was added dropwise at 0 °C, and the reaction mixture stirred for 75 min at 0 °C. The reaction was quenched by the addition of H_2O (20 mL), and extracted with EtOAc (3 × 30 mL). The organic extracts were combined, washed with aqueous NaOH (1.0 M, 20 mL), aqueous LiCl solution (0.5 M, 2 × 30 mL), and brine (30 mL), dried over Na_2SO_4, filtered, and concentrated *in vacuo* to isolate the title compound as a pale purple solid. No further purification was required (76 mg, 94% based on a 40% Cl : 60% Br ratio determined using ^{1}H NMR); R_f 0.38 (10% MeOH/CH_2Cl_2); ^{1}H NMR (400 MHz, $CDCl_3$) δ_H 9.02 (1H, br s, 1^i-N*H*), 7.40 (1H, d, *J* 2.2, 2-C*H*), 7.03–6.92 (1H, m, 3^{ii}-C*H*), 6.88 (1H, dd, *J* 8.4, 3.4, 5-C*H*), 6.59 (1H, dd, *J* 8.4, 2.2, 6-C*H*), 6.26 (0.4H, dt, *J* 15.0, 1.6, 2^{ii}-$CH_{(Cl)}$), 6.19 (0.6H, dt, *J* 15.0, 1.1, 2^{ii}-$CH_{(Br)}$), 4.22 (0.8H, dd, *J* 5.9, 1.6, 4^{ii}-$CH_{2(Cl)}$), 4.07 (1.2H, dd, *J* 7.3, 1.1, 4^{ii}-$CH_{2(Br)}$), 3.73–3.67 (5H, m, 1^{iii}-CH_3, 5^{iv}-CH_2), 2.62 (2H, t, *J* 8.1, 3^{iv}-CH_2), 2.26–2.17 (2H, m, 4^{iv}-CH_2); LRMS *m/z* (ESI$^+$) 309, 311 ([M+H]$^+$; ^{35}Cl, 100%; ^{37}Cl, 33%), 353, 355 ([M+H]$^+$; ^{79}Br, 100%; ^{81}Br, 97%).

(*E*)-*N*-[3-Methoxy-4-(2-oxopyrrolidin-1-yl)phenyl]-4-[methyl(prop-2-ynyl)amino]but-2-enamide (41)

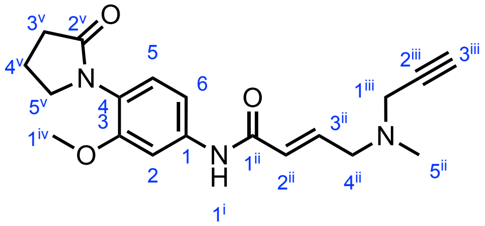

Potassium iodide (71 mg, 0.43 mmol, 2.0 eq) was added to a solution of (*E*)-4-bromo/chloro-*N*-(3-methoxy-4-(2-oxopyrrolidin-1-yl)phenyl)but-2-enamide (**44**) (75 mg, 0.21 mmol, 1.0 eq) in anhydrous DMF (2 mL) at rt. The reaction mixture was stirred at rt for 10 min, after which it was cooled to 0 °C. *N*-Methylpropargylamine (36 µL, 0.43 mmol, 2.0 eq) was added then added dropwise at 0 °C, followed by K_2CO_3 (59 mg, 0.43 mmol, 2.0 eq), and the suspension stirred at 0 °C for 5 min before slowly warming to rt. The reaction mixture was stirred at rt for 2 h before being quenched by the addition of

aqueous HCl (1.0 M, 20 mL), and washed with EtOAc (20 mL). The acidic aqueous extract was basified with aqueous NaOH (2.0 M) until pH 11 was attained, extracted with EtOAc (3 × 20 mL), washed with aqueous LiCl solution (0.5 M, 20 mL), and brine (20 mL), dried over Na_2SO_4, filtered, and concentrated *in vacuo*. The crude product was purified using semi-preparative HPLC (see general chemical methods) to isolate the formic acid salt, which was then dissolved in aqueous NaOH (1.0 M), extracted with EtOAc (3 × 20 mL), and concentrated *in vacuo* to isolate the free base as a colorless solid (20 mg, 28%); R_f 0.30 (10% MeOH/CH_2Cl_2); m.p. 130–132 °C (from $CHCl_3$); $\tilde{\nu}_{max}$ (thin film)/cm^{-1} 3279 (N-H, m), 2944 (C-H, w), 1667 (C=O, s), 1609 (C=C, s); ^{1}H NMR (600 MHz, $CDCl_3$) δ_H 8.77 (1H, br s, 1^i-N*H*), 7.45 (1H, d, *J* 1.9, 2-C*H*), 6.91 (1H, d, *J* 8.3, 5-C*H*), 6.88 (1H, dt, *J* 15.3, 6.3, 3^{ii}-C*H*), 6.64 (1H, dd, *J* 8.3, 1.9, 6-C*H*), 6.16 (1H, dt, *J* 15.3, 1.5, 2^{ii}-C*H*), 3.71–3.67 (5H, m, 1^{iv}-CH_3, 5^v-CH_2), 3.39 (2H, d, *J* 2.2, 1^{iii}-CH_2), 3.25 (2H, dd, *J* 6.3, 1.5, 4^{ii}-C*H*), 2.59 (2H, t, *J* 8.1, 3^v-CH_2), 2.37 (3H, s, 5^{ii}-CH_3), 2.27 (1H, t, *J* 2.2, 3^{iii}-C*H*), 2.22–2.16 (2H, m, 4^v-CH_2); ^{13}C NMR (151 MHz, $CDCl_3$) δ_C 176.3 (2^v-C), 163.8 (1^{ii}-C), 154.9 (3-C), 140.7 (3^{ii}-C), 139.5 (1-C), 128.3 (5-C), 127.1 (2^{ii}-C), 122.0 (4-C), 112.3 (6-C), 104.6 (2-C), 78.3 (2^{iii}-C), 73.8 (3^{iii}-C), 56.7 (4^{ii}-C), 55.6 (1^{iv}-C), 50.5 (5^v-C), 45.5 (1^{iii}-C), 41.9 (5^{ii}-C), 31.5 (3^v-C), 18.9 (4^v-C); HRMS *m/z* (ESI$^+$) [Found: 342.1814, $C_{19}H_{24}O_3N_3$ ([M+H]$^+$) requires 342.1812]; LRMS *m/z* (ESI$^+$) 342 ([M+H]$^+$, 100%); HPLC Retention time 5.6 min, >99.0%.

1-(4-Amino-2-hydroxyphenyl)pyrrolidin-2-one (45)

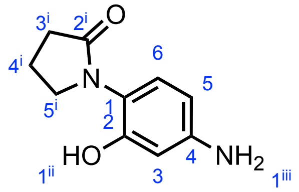

Boron tribromide (1.0 M in CH_2Cl_2, 7.27 mL, 7.27 mmol, 3.0 eq) was added dropwise to a solution of 1-(4-amino-2-methoxyphenyl)pyrrolidin-2-one (**43**) (500 mg, 2.42 mmol, 1.0 eq) in anhydrous CH_2Cl_2 (10 mL) at 0 °C. The reaction mixture was slowly warmed to rt and stirred for 3 h before being cooled back to 0 °C and a further portion of boron tribromide (1.0 M in CH_2Cl_2, 3.63 mL, 3.63 mmol, 1.5 eq) was added dropwise. The reaction mixture was then stirred at rt for a further 20 h before being quenched by the addition of saturated aqueous $NaHCO_3$ solution (50 mL) and extracted with EtOAc (3 × 50 mL). The combined organic layers were dried over Na_2SO_4, filtered, and concentrated *in vacuo*. The crude product was purified using silica gel column column chromatography, eluting with EtOAc, to isolate the title compound as a pale pink solid (361 mg, 78%); R_f 0.26 (EtOAc); m.p. 128–130 °C (from EtOAc); $\tilde{\upsilon}_{max}$ (neat)/cm^{-1} 3425 (O-H, w), 3351 (N-H, w), 3248 (N-H, w), 2980 (C-H, w), 1649 (C=O, s), 1441 (N-O, s), 1246 (w), 832 (C-H, s), 800 (w); ^{1}H NMR (600 MHz, D_6-DMSO) δ_H 8.96 (1H, s, 1^{ii}-O*H*), 6.70 (1H, d, *J* 8.3, 6-C*H*), 6.10 (1H, d, *J* 2.4, 3-C*H*), 6.01 (1H, dd, *J* 8.3, 2.4, 5-C*H*), 4.99 (2H, s, 1^{iii}-NH_2), 3.57 (2H, t, *J* 7.0, 5^i-CH_2), 2.33 (2H, t, *J* 8.0, 3^i-CH_2), 2.02 (2H, m, 4^i-CH_2); ^{13}C NMR (NMR 151 MHz; D_6-DMSO) δ_C 174.1 (2^i-C), 153.0 (3-C), 148.8 (4-C), 127.8 (6-C), 115.1 (1-C), 105.3 (5-C),

101.7 (3-C), 49.7 (5^{i}-C), 30.8 (3^{i}-C), 18.1 (4^{i}-C); HRMS *m/z* (ESI$^+$) [Found: 193.0970, $C_{10}H_{13}N_2O_2$ requires $[M+H]^+$ 193.0970]; LRMS *m/z* (ESI$^+$) 193 ($[M+H]^+$, 100%), 215 ($[M+Na]^+$, 57%); HPLC Retention time 2.6 min, 88.6%.

N-[3-Hydroxy-4-(2-oxopyrrolidin-1-yl)phenyl]prop-2-enamide (46)

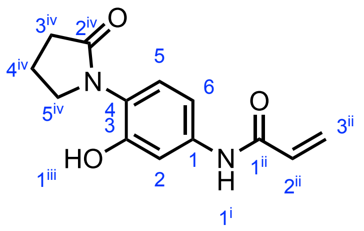

Acryloyl chloride (10 µL, 0.12 mmol, 1.1 eq) was added dropwise to a solution of 1-(4-amino-2-hydroxyphenyl)pyrrolidin-2-one (**45**) (21 mg, 0.11 mmol, 1.0 eq) and pyridine (10 µL, 0.12 mmol, 1.1 eq) in anhydrous CH_2Cl_2 (0.6 mL) at 0 °C. The reaction solution was slowly warmed to rt and stirred for 2 h before being re-cooled to 0 °C and a further portion of pyridine (10 µL, 0.12 mmol, 1.1 eq) and acryloyl chloride (10 µL, 0.12 mmol, 1.1 eq) being added dropwise. The reaction solution was then stirred at rt for a further 2 h before being quenched by the addition of saturated aqueous $NaHCO_3$ solution (5 mL) and extracted with CH_2Cl_2 (2 × 5 mL). The combined organic phases were washed with aqueous HCl (1.0 M, 2 × 5 mL), dried over Na_2SO_4, filtered, and concentrated *in vacuo* to afford the title compound as a yellow solid (12 mg, 43%); R_f 0.26 (EtOAc); m.p. 162–164 °C (from CH_2Cl_2); $\tilde{\upsilon}_{max}$ (neat)/cm^{-1} 3109 (O-H, w), 2920 (C-H, w), 2850 (C-H, w), 1660 (C=O, s), 1609 (C=O, s), 1524 (C=C, s), 1421 (s), 1246 (N-O, w), 975 (C=C-H, w), 855 (w); ^{1}H NMR (600 MHz, D$_6$-DMSO) δ_H 10.07 (1H, s, 1^{i}-N*H*), 9.61 (1H, s, 1^{iii}-O*H*), 7.42 (1H, d, *J* 2.1, 2-C*H*), 7.06–7.01 (2H, m, 5-C*H*, 6-C*H*), 6.42 (1H, dd, *J* 16.9, 10.0, 2^{ii}-C*H*), 6.24 (1H, dd, *J* 16.9, 1.9, 3^{ii}-CH_xH$_y$), 5.74 (1H, dd, *J* 10.0, 1.9, 3^{ii}-CH$_x$$H_y$), 3.66 (2H, t, *J* 6.9, 5^{iv}-CH_2), 2.37 (2H, t, *J* 8.1, 3^{iv}-CH_2), 2.07 (2H, m, 4^{iv}-CH_2); ^{13}C (NMR 151 MHz; DMSO-D$_6$) δ_C 174.2 (2^{iv}-C), 163.0 (1^{ii}-C), 152.5 (3-C), 138.6 (1-C), 131.9 (2^{ii}-C), 127.8 (5-C), 126.8 (3^{ii}-C), 121.7 (4-C), 110.3 (6-C), 107.7 (2-C), 49.3 (5^{iv}-C), 30.7 (3^{iv}-C), 18.3 (4^{iv}-C); HRMS *m/z* (ESI$^+$) [Found: 247.1078, $C_{13}H_{15}N_2O_3$ requires $[M+H]^+$ 247.1077]; LRMS *m/z* (ESI$^+$) 247 ($[M+H]^+$, 93%), 269 ($[M+Na]^+$, 100%); HPLC Retention time 5.5 min, 83.5%.

N-[4-(2-Oxopyrrolidin-1-yl)-3-prop-2-ynoxyphenyl]prop-2-enamide (42)

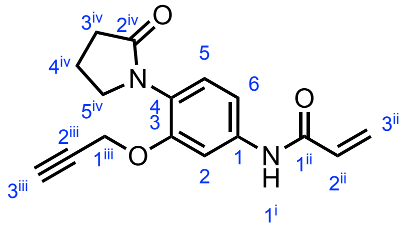

To a solution of *N*-[3-hydroxy-4-(2-oxopyrrolidin-1-yl)phenyl]prop-2-enamide (**46**) (25 mg, 0.10 mmol, 1.0 eq) in anhydrous DMF (2 mL), was added K_2CO_3 (21 mg, 0.15 mmol, 1.5 eq). After

10 min propargyl bromide (80% *v/v* in toluene, 12 μL, 0.12 mmol, 1.2 eq) was added. The mixture was stirred at rt for 20 h, then diluted with water (5 mL) and extracted with EtOAc (3 × 5 mL). The organic phases were combined, dried over Na_2SO_4, filtered, and concentrated *in vacuo*. The crude product was purified using silica gel column chromatography, eluting with 70–100% EtOAc/petroleum ether, to isolate the title compound as a yellow solid (10 mg, 35%); R_f 0.51 (10% MeOH/EtOAc); m.p. 110–112 °C (from EtOAc); $\tilde{\upsilon}_{max}$ (neat)/cm^{-1} 3291 (N-H, w), 2918 (C-H, w), 2850 (C-H, w), 2114 (C≡C, w), 1673 (C=O, s), 1609 (C=O, s), 1546 (C=C, s), 1516 (w), 1416 (s), 1269 (N-O, w), 1036 (C=C-H, w), 851 (w), 637 (C≡C-H, w); ^{1}H NMR (600 MHz, $CDCl_3$) δ_H 9.05 (1H, s, 1^{i}-N*H*), 7.47 (1H, d, *J* 1.9, 2-C*H*), 6.91 (1H, d, *J* 8.2, 5-C*H*), 6.71 (1H, dd, *J* 8.2, 1.9, 6-C*H*), 6.39 (1H, dd, *J* 16.7, 1.4, 3^{ii}-CH_xH$_y$), 6.27 (1H, dd, *J* 16.7, 9.9, 2^{ii}-C*H*), 5.68 (1H, d, *J* 9.9, 1.4, 3^{ii}-CH$_x$$H_y$), 4.54 (2H, d, *J* 2.4, 1^{iii}-CH_2), 3.71 (2H, t, *J* 7.0, 5^{iv}-CH_2), 2.59 (2H, t, *J* 8.0, 3^{iv}-CH_2), 2.49 (1H, t, *J* 2.4, 3^{iii}-C*H*), 2.20 (2H, m, 4^{iv}-CH_2); ^{13}C NMR (151 MHz; $CDCl_3$) δ_C 176.5 (2^{iv}-C), 164.0 (1^{ii}-C), 152.8 (3-C), 139.4 (1-C), 131.5 (2^{ii}-C), 128.5 (5-C), 127.2 (3^{ii}-C), 122.4 (4-C), 113.5 (6-C), 105.9 (2-C), 78.1 (2^{iii}-C), 75.9 (3^{iii}-C), 56.3 (1^{iii}-C), 50.7 (5^{iv}-C), 31.5 (3^{iv}-C), 19.0 (4^{iv}-C); HRMS *m/z* (ESI$^+$) [Found: 285.1231, $C_{16}H_{17}N_2O_3$ requires [M+H]$^+$ 285.1234]; LRMS *m/z* (ESI$^+$) 285 ([M+H]$^+$, 100%), 307 ([M+Na]$^+$, 71%); HPLC Retention time 6.5 min, 96.9%.

General Biology Methods

Chemicals and cell culture reagents

Table S2. Materials were obtained from the following suppliers:

Material	Supplier
Acetic Acid	Sigma Aldrich
Acetonitrile	Sigma Aldrich
Agarose	Sigma Aldrich
Bacto agar	Fisher Scientific
β-Mercaptoethanol	Fisher Scientific
Bovine serum albumin (BSA)	Sigma Aldrich
Bromophenol blue	Fisher Scientific
Calcium chloride	Sigma Aldrich
Copper Sulfate ($CuSO_4$)	Sigma Aldrich
DMSO	Sigma Aldrich
DMEM (Dulbecco's modified eagle medium)	Gibco
Ethanol	Sigma Aldrich
Ethylenediaminetetraacetic acid (EDTA)	Sigma Aldrich
Fetal bovine serum (FBS)	Gibco
GlutaMAX™	Gibco
Glycerol	Sigma Aldrich
Glycine	Sigma Aldrich
Imidazole	Sigma Aldrich
InstantBlue® coomassie protein stain	Abcam
Lipofectamine 2000	Invitrogen
Methanol	Sigma Aldrich
MOPS SDS running buffer	Invitrogen
Nickel sulfate hexahydrate ($NiSO_4 \cdot 6H_2O$)	Sigma Aldrich

Material	Supplier
Opti-MEM™ Reduced Serum Medium	Gibco
Phosphate-buffered saline (PBS)	Gibco
Ponceau S	Sigma Aldrich
SOC media	New England Biolabs
Sodium chloride (NaCl)	Sigma Aldrich
Sodium dodecyl sulphate (SDS)	Fisher Scientific
SYBR Safe™	Invitrogen
TAMRA-PEG$_3$-Azide	Sigma Aldrich
Tris(benzyltriazolylmethyl)amine (TBTA)	Sigma Aldrich
Tris(2-carboxyethyl)phosphine hydrochloride (TCEP)	Sigma Aldrich
Tris(hydroxymethyl)aminomethane hydrochloride) (Tris-HCl)	Thermo Fisher Scientific
Triton X-100	Thermo Fisher Scientific
Trypsin 0.5% EDTA	Gibco
Tryptone	Sigma Aldrich
Tween 20	Fisher Scientific
Urea	Fisher Scientific
Yeast Extract	Sigma Aldrich
Zinc	Sigma Aldrich

Bacterial cell culture

Standard sterile techniques were followed thoroughly. Media and equipment were sterilized by autoclaving at 121 °C for 20 min. Solutions of IPTG, antibiotics and other labile compounds were sterilized by filtration through 0.22 µm filters (Millex®GP, Merck Millipore).

Kanamycin was prepared as a stock solution in water of 50 mg/mL. Final concentrations of antibiotics, unless otherwise stated, were kanamycin 50 µg/mL.

Incubation of bacteria was performed in a New Brunswick G-25 or Innova-44 Shaker Incubator.

Optical density at 600 nm (OD_{600}) was measured with a Novaspec III visible spectrophotometer.

Escherichia coli (*E. coli*) strains of the following genotypes were used:

- **BL21 (DE3) Gold**: F$^-$ ompT hsdSB (rB$^-$mB$^-$) gal dcm (DE3). DE3 denotes a chromosomal copy of the T7 RNA polymerase gene. – For protein expression
- **NEB 5-alpha** (New England Biolabs Inc.); genotype: *fhuA2 Δ(argF-lacZ)U169 phoA glnV44 Φ80Δ(lacZ)M15 gyrA96 recA1 relA1 endA1 thi-1 hsdR17* – For plasmid amplification

Cells were grown in 2×TY broth, according to the experimental requirements.

Mammalian cell culture

HEK293T cells were cultured in DMEM supplemented with 10% FBS and GlutaMAX in a standard humidified incubator for mammalian tissue culture maintained at 37 °C, 21% O_2 and 5% CO_2.

Cells were passaged 2–3 times per week. In each case, the medium was removed, and the cells washed with rt PBS. Cells were detached by incubating with 37 °C Trypsin-EDTA solution for 2–4 min. The trypsin was quenched by the addition of fresh 37 °C DMEM supplemented with 10% FBS and GlutaMAX. Cells were counted using a Neubauer haemocytometer chamber.

Protein expression and purification

Cloning

DNA amplification - PCR

DNA amplification was carried out in a thermal cycler (Labnet Multigene II Personal Thermal Cycler). An initial denaturation step at 98 °C for 3 min was followed by 30 cycles of denaturing at 98 °C for 10 sec, annealing at 63 °C for 30 sec, and extension at 72 °C for 30 sec was carried out. A final extension of 2 min at 72 °C was followed by the resulting samples being cooled to 4 °C.

Table S3. Components for DNA amplification.

**Component**	**Volume per 50 µL Reaction (µL)**	**Final Concentration**
5× Q5 Reaction Buffer	10	1×
10 mM dNTPs	1	200 µM
10 µM Forward Primer	2.5	0.5 µM
10 µM Reverse Primer	2.5	0.5 µM
Template DNA	2	< 1,000 ng
Q5 High-Fidelity DNA Polymerase	0.5	0.02 U/µL
Nuclease-Free Water	31.5	n.a.

DNA purification - Agarose gel electrophoresis

DNA samples were analyzed and purified by 2% agarose gel electrophoresis prepared with SYBR® safe DNA gel stain (Invitrogen). DNA samples were loaded after mixing with 6 × loading buffer and run in a TAE buffer (Table S4). The gels were run at 100 V until the desired fragments had separated. Gels were visualized using a UV transilluminator (Gel logic 200). A GeneRuler DNA Ladder, 1 kb (Thermo Scientific) was used molecular weight reference.

Digested DNA was purified using a GeneJET Gel Extraction Kit (Thermo Scientific) according to manufacturer's instructions. Plasmids were purified using a GeneJET Plasmid Miniprep Kit (Thermo Scientific) according to manufacturer's instructions. DNA concentration was estimated by measuring absorbance at 260 nm using a Nanodrop® ND-1000 spectrophotometer (Nanodrop® Technologies Inc.).

Table S4. Buffer compositions for DNA purification.

Buffer	Reagent	Amount required
50× TAE Running Buffer (per 1 L)	Tris·HCl	242.0 g
	Acetic acid (glacial)	57.0 mL
	EDTA (0.5 M)	100 mL
6× DNA Loading Buffer (per 10 mL)	Milli-Q H_2O	6.7 mL
	Bromophenol blue	25.0 mg
	Glycerol	3.30 mL
	Xylene cyanol FF	25.0 mg
DNA electrophoresis gel (per gel)	Agarose	1.0 g
	TAE buffer (1×)	50.0 mL
	SYBR® Safe DNA gel stain	5.0 µL

Gibson Assembly

Cloning was performed using a one-step isothermal *in vitro* recombination reaction consisting of T5 exonuclease, Phusion DNA polymerase and Taq DNA ligase. Linear vectors (~0.03 pmol) and gene inserts (~0.1 pmol) with complementary regions were incubated at 50 °C for 15-60 min in a 1:3 ratio with Gibson Assembly master mix (NEB): 20 µL reaction volume, 10 µL 2 × GA master mix. 2 µL of the reaction mixture was used directly to transform NEB 5-alpha (#C2987) competent cells.

Table S5. Gene inserts were generated by PCR from parental DNA templates using the following primers:

**Primer**	**Direction**	**Sequence (5' to 3')**	**Tm (°C)**
GA_f	Forward	AGAACCTGTACTTCCAATCC	61
GA_r	Reverse	CGGAGCTCGAATTCG	60

DNA Sequencing

Plasmid sequencing was performed by Source BioScience Sequencing facility in the Department of Biochemistry (University of Oxford). T7 promoter and terminator sequence primers were used, on a 3730xl DNA Analyser-Titania.

Competent cells

Competent cells were thawed slowly on ice for 20 min prior to use. 1–5 µL containing 50–100 ng of plasmid DNA was added to 20 µL of the competent cells. The tubes were placed on ice for 30 min before incubated at 42 °C for 30 sec. The tubes were returned to ice for 5 min. Gently 0.5 mL of SOC outgrowth medium (New England Biolabs Inc.) was added to each tube. The tubes were incubated at 37 °C for 1 h prior to 100-400 µL of the transformation mixture being spread over agar plates containing kanamycin (50 µg/mL) and incubated overnight, under aseptic conditions.

Fermentation conditions for human bromodomain containing proteins

For the expression and purification of the human bromodomain containing proteins the published procedure from Filippakopoulos *et al.* was followed.[20] Single colonies of transformed *E. coli* BL21 (DE3) were transferred, under aseptic conditions, to 100 mL of 2× TY medium containing kanamycin (50 µg/mL) (Table S6). Cultures were incubated over night at 37 °C and 180 rpm. From the overnight night preculture 10 mL was transferred to 1 L of 2× TY medium containing kanamycin (50 µg/mL), and incubated at 37 °C and 180 rpm until an $O.D_{600}$ of 0.8 was reached. $O.D_{600}$ readings were taken in 1.6 mL cuvettes with 2× dilution in 2× TY medium against a reference of 2× TY medium, using a Novaspec® II spectrophotometer measuring at 600 nm. The cultures were then cooled to 16 °C, 60 rpm, and IPTG (final concentration of 0.1 mM) was added. The cultures were then further incubated overnight at 16 °C and 180 rpm. Cells were pelleted by centrifugation (11,325 ×g, 4 °C, 10 min), and the pellets collected, combined, and stored at −80 °C.

Table S6. Media composition for bacterial cell culture.

Media	Reagent	Amount per liter (g)
2× TY	Tryptone	16.0
	Yeast Extract	10.0
	NaCl	5.0

Purification conditions for human bromodomain containing proteins

Cell pellets were re-suspended in extraction buffer (100 mL per 20 g cell mass) with 250 Units (U) of Benzonase at 4 °C (Table S7). The cells were lysed by sonication (60 × 5 sec burst, 10 sec pause), followed by the addition of polyethyleneimine (PEI, 0.15% *v/v*) to precipitate DNA. The lysate was incubated over ice for 15 min. The lysate was centrifuged (25,000 ×g, 4 °C, 15 min) to pellet cell debris, the resulting supernatant was decanted, filtered through a 0.45 μm filter and applied to a purification column. The protein was initially purified at 4 °C using immobilized metal affinity chromatography (IMAC) with a HisTrap™ (5 mL) column (GE Healthcare) rinsed with 10 CV of Milli-Q water, then charged with 4 CV of 100 mM $NiSO_4$, and subsequently equilibrated with 10 CV of binding buffer (supplemented with 10 mM β-ME). The column was then loaded with the lysate at a rate of 1 mL/min. The column was washed with a step gradient of 10 CV 9% elution buffer (45 mM imidazole), and 10 CV 12% elution buffer (60 mM imidazole). The polyhistidine-tagged protein of interest was batch eluted with 4 CV of 60% elution buffer (300 mM imidazole) followed by 2 CV of 100% elution buffer (500 mM imidazole). Eluted protein was collected by fractionation in 2 mL volumes. Fractions containing the highest levels of pure protein, as determined by UV trace and SDS-PAGE gels, were collected, combined, and concentrated using 5k molecular weight cut-off concentrators (GE Healthcare).

Further purification of the desired protein was achieved using size exclusion chromatography (SEC). Concentrated IMAC fractions (2 mL) were loaded onto a 120 mL Superdex 75 size exclusion column (Amersham) equilibrated with 150 mL of gel filtration buffer, *via* a 2 mL injection loop. The protein was eluted with a further 150 mL of gel filtration buffer, and collected by fractionation in 2 mL volumes. Fractions containing protein were analyzed using SDS-PAGE gel, and the fractions containing the highest levels of pure protein were collected, combined, and concentrated using 5k molecular weight cut-off concentrators (GE Healthcare). Protein concentration was determined using a Nanodrop® ND-1000 spectrophotometer (Nanodrop® Technologies Inc.) measuring absorbance at 280 nm. Protein molecular weight and molar extinction coefficient were calculated using ProtParam on the ExPASy Bioinformatics Resource Portal. Proteins were stored at −80 °C.

Table S7. Buffer compositions for protein purification.

Buffer[α]	Reagent	Concentration	Amount per 1 L
Extraction Buffer (pH = 7.6)	HEPES	50 mM	11.9 g
	NaCl	500 mM	29.2 g
	Glycerol	10% (*v/v*)	100 mL
	Sigma*FAST*[β]	1 tab/ 100 mL	10 tabs
Binding Buffer (pH = 7.6)	HEPES	50 mM	11.9 g
	NaCl	500 mM	29.2 g
	Glycerol	10% (*v/v*)	100 mL
Elution Buffer (pH = 7.6)	HEPES	50 mM	11.9 g
	NaCl	500 mM	29.2 g
	Glycerol	10% (*v/v*)	100 mL
	Imidazole	500 mM	34.0 g
Re-Charge	$NiSO_4 \cdot 6H_2O$	100 mM	26.3 g
Strip Buffer (pH = 7.6)	HEPES	50 mM	11.9 g
	NaCl	500 mM	29.2 g
	EDTA	50 mM	18.6 g
Gel Filtration Buffer	HEPES	50 mM	11.9 g
	NaCl	500 mM	29.2 g

[α] All buffers were filtered through a 0.22 µm filter and degassed

[β] Sigma*FAST* Protease Inhibitor Cocktail Tablet, EDTA Free

Sodium dodecyl sulfate polyacrylamide gel electrophoresis (SDS-PAGE)

Protein purity was analyzed using SDS-PAGE gel electrophoresis. Gels were prepared using 70 mm × 100 mm glass plates, with 0.75 mm spacers. TEMED and freshly prepared ammonium persulfate were added just prior to pouring the gels (Table S8). The resolving gel was cast with the addition of a separate layer of isopropyl alcohol (to ensure a level surface). Once the resolving layer had polymerized, isopropyl alcohol was removed, and the stacking gel was cast. For protein denaturation samples were prepared by mixing with sample loading buffer (4×) and incubation at 100 °C for 10 min (Table S9). A color protein standard broad range (NEB) molecular weight ladder was used a reference. Gels were run on a mini-PROTEAN Tetra Electrophoresis System (Bio-Rad) at a constant potential of 100 V. Following electrophoresis, gels were stained for 30 min with InstantBlue™ Coomasie® stain (Abcam) and afterwards destained for 5 h.

Table S8. Gel compositions for a single gel.

Reagent	Resolving Gel (16%)	Stacking Gel (4%)
Tris-HCl (1.5 M, pH 8.8)	1.125 mL	-
Tris-HCl (0.5 M, pH 6.8)	-	0.65 mL
Milli-Q Water	1.05 mL	1.45 mL
30% (*w/v*) Acrylamide	2.56 mL	0.35 mL
SDS (10% *w/*v)	50 µL	25.0 µL
10 % APS	50 µL	25.0 µL
TEMED	4 µL	2.5 µL

Table S9. Buffer compositions for SDS-PAGE.

Buffer	Reagent	Composition	Amount per 1 L
SDS-PAGE Running Buffer	Tris-HCl	-	30.0 g
	Glycine	-	144 g
	SDS	-	10.0 g
SDS-PAGE Sample Loading Buffer (pH 6.8)	Tris-HCl	0.5 M	10.0 mL
	Bromophenol Blue	0.2% *w/v*	0.20 g
	SDS	10% *w/v*	20.0 mL
	Glycerol	-	12.0 mL
	β-mercaptoethanol	-	5.0 mL
SDS-PAGE Stain	Coomassie® Brilliant Blue	0.25%	2.5 mL
	Acetic Acid	10%	100 mL
	Methanol	30%	300 mL
SDS-PAGE Destain	Acetic Acid	10%	100 mL
	Methanol	40%	400 mL

Table S10. Characterization of His$_6$-BRD4(1)^{WT}.

**Protein:**	**His$_6$-BRD4(1)^{WT}**
**Plasmid vector:**	pNIC28-Bsa4 (Kanamycin)
**DNA Sequence:**	ATG CAC CAT CAT CAT CAT CAT TCT TCT GGT GTA GAT CTG GGT ACC GAG AAC CTG TAC TTC CAA TCC ATG AAC CCC CCG CCC CCA GAG ACC TCC AAC CCT AAC AAG CCC AAG AGG CAG ACC AAC CAA CTG CAA TAC CTG CTC AGA GTG GTG CTC AAG ACA CTA TGG AAA CAC CAG TTT GCA TGG CCT TTC CAG CAG CCT GTG GAT GCC GTC AAG CTG AAC CTC CCT GAT TAC TAT AAG ATC ATT AAA ACG CCT ATG GAT ATG GGA ACA ATA AAG AAG CGC TTG GAA AAC AAC TAT TAC TGG AAT GCT CAG GAA TGT ATC CAG GAC TTC AAC ACT ATG TTT ACA AAT TGT TAC ATC TAC AAC AAG CCT GGA GAT GAC ATA GTC TTA ATG GCA GAA GCT CTG GAA AAG CTC TTC TTG CAA AAA ATA AAT GAG CTA CCC ACA GAA GAA
**Protein Sequence:**	MHHHHHHSSGVDLGTENLYFQSMNPPPPETSN PNKPKRQTNQ LQYLLRVVLK TLWKHQFAWP FQQPVDAVKL NLPDYYKIIK TPMDMGTIKK RLENNYYWNA QECIQDFNTM FTNCYIYNKP GDDIVLMAEA LEKLFLQKIN ELPTEE
**Molecular Weight:**	17549 Da
**Extinction coefficient:**	28545 M^{-1} cm^{-1}

Table S11. Characterization of His$_6$-BRD4(1)^{L94C}. The bold red letters show the mutation.

**Protein:**	**His$_6$-BRD4(1)^{L94C}**
**Plasmid vector:**	pNIC28-Bsa4 (Kanamycin)
**DNA Sequence:**	ATG CAC CAT CAT CAT CAT CAT TCT TCT GGT GTA GAT CTG GGT ACC GAG AAC CTG TAC TTC CAA TCC ATG AAC CCC CCG CCC CCA GAG ACC TCC AAC CCT AAC AAG CCC AAG AGG CAG ACC AAC CAA CTG CAA TAC CTG CTC AGA GTG GTG CTC AAG ACA CTA TGG AAA CAC CAG TTT GCA TGG CCT TTC CAG CAG CCT GTG GAT GCC GTC AAG CTG AAC **TGT** CCT GAT TAC TAT AAG ATC ATT AAA ACG CCT ATG GAT ATG GGA ACA ATA AAG AAG CGC TTG GAA AAC AAC TAT TAC TGG AAT GCT CAG GAA TGT ATC CAG GAC TTC AAC ACT ATG TTT ACA AAT TGT TAC ATC TAC AAC AAG CCT GGA GAT GAC ATA GTC TTA ATG GCA GAA GCT CTG GAA AAG CTC TTC TTG CAA AAA ATA AAT GAG CTA CCC ACA GAA GAA
**Protein Sequence:**	MHHHHHHSSGVDLGTENLYFQSMNPPPPETSN PNKPKRQTNQ LQYLLRVVLK TLWKHQFAWP FQQPVDAVKL N**C**PDYYKIIK TPMDMGTIKK RLENNYYWNA QECIQDFNTM FTNCYIYNKP GDDIVLMAEA LEKLFLQKIN ELPTEE
**Molecular Weight:**	17539 Da
**Extinction coefficient:**	28545 M^{-1} cm^{-1}

Mammalian protein expression

DNA transfection

HEK293T cells at ~40% confluency were transfected using Lipofectamine 2000 (Invitrogen) according to the manufacturer's instructions. The media was changed after 24 h, and the cells left to grow for a further 24 h before being used at a confluency of ~90%.

Table S12. Quantities of Lipofectamine 2000 and DNA used for transfection of HEK293T cells.

Mixture	Component	10 cm Dish	12 Well Plate
Diluted Lipofectamine	Opti-MEM	500 µL	72 µL
	Lipofectamine 2000	45 µL	3 µL
Diluted DNA	Opti-MEM	500 µL	74 µL
	DNA (~1 µg/mL)	15 µL	1 µL

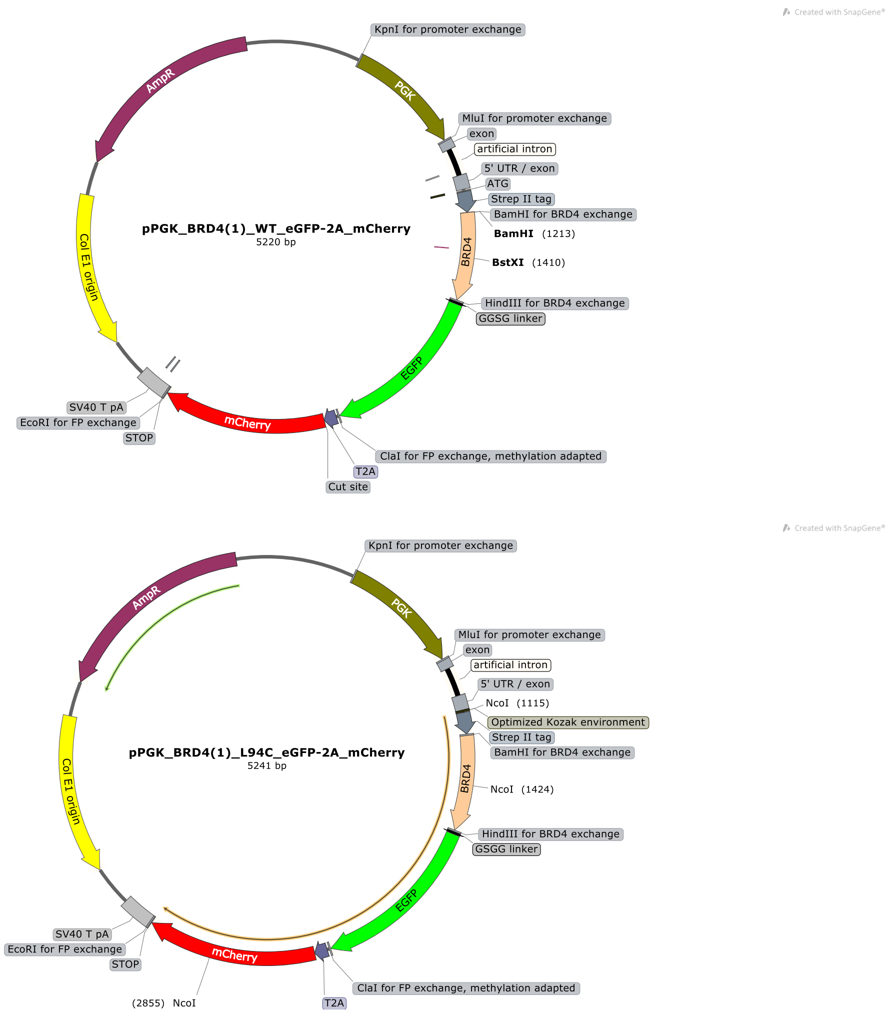

Figure S24. Plasmid maps for the constructs used to transiently express BRD4(1) in HEK293T cells.

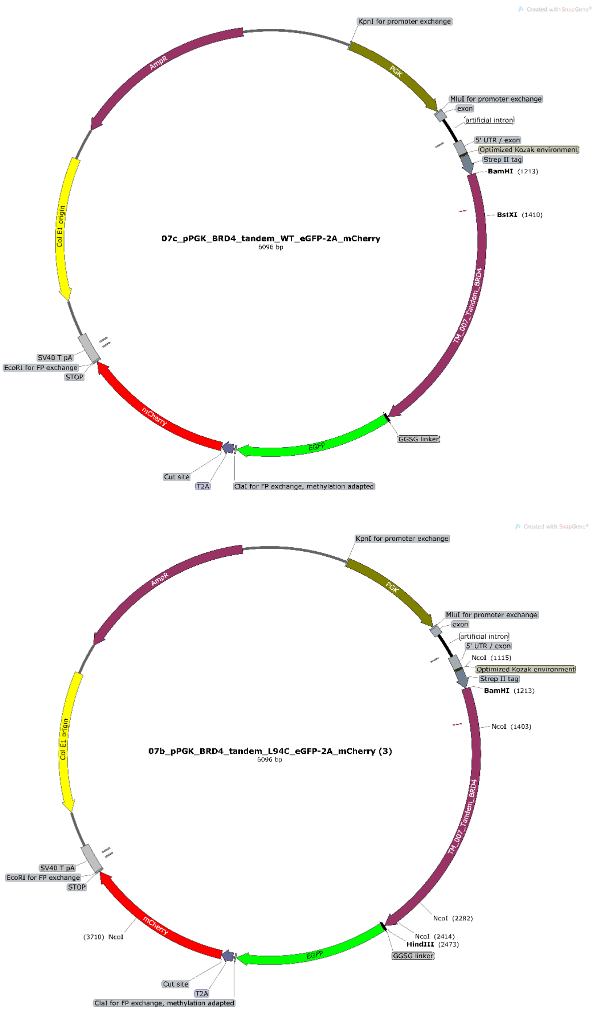

Figure S25. Plasmid maps for the constructs used to transiently express tandem BRD4(1,2) in HEK293T cells.

Cell harvesting and lysis

To harvest adherent HEK293T cells, the medium was removed, and the cells washed with rt PBS. Cells were detached by incubating with 37 °C trypsin-EDTA solution for 2–4 min. The trypsin was quenched by the addition of fresh 37 °C DMEM supplemented with 10% FBS and GlutaMAX. The detached cells were then pelleted (500 ×g, rt, 5 min) and washed with PBS, before being pelleted again (500 ×g, rt, 5 min).

Cell pellets were lysed by resuspending the pellet in lysis buffer (1% Triton X-100, 0.1% SDS, and 1:200 Protease inhibitor cocktail) and incubating at 4 °C for 20–30 min. The lysate was centrifuged (13,000 ×g, 4 °C, 10 min) to pellet cell debris, and the resulting supernatant was transferred to fresh Eppendorf tubes.

Determination of total protein concentration

Total protein concentration of the lysate was determined by a BCA protein assay (Thermo Scientific) and the absorbance at 562 nm measured and plotted using a NanoDrop One. Cell lysates were diluted to 2 mg/mL with lysis buffer, unless otherwise stated.

Sodium dodecyl sulfate polyacrylamide gel electrophoresis (SDS-PAGE)

Cell lysate proteins were separated by SDS-PAGE gel electrophoresis. 4× Loading buffer (4× LDS sample buffer and 2% β-mercaptoethanol) was added to the diluted protein samples and then heated at 95 °C for 5 min. Protein samples (15–20 µL) were loaded into NuPAGE™ 4-12% Bis-Tris Gels and electrophoresed for 70–80 min at 180V in MOPS buffer. The Precision Plus Protein Kaleidoscope Prestained Protein Standards (Bio-rad) (5 µL) was used as a molecular weight reference.

Western blotting

Proteins in the electrophoresed gels were transferred onto a PVDF membrane for 1 h at 100 V, at 4 °C. The primary antibodies were diluted (Table S13) in 5% milk in TBS-Tween (TBS-T, 0.1% Tween). The membrane was incubated with the primary antibodies for 16 h at 4 °C. The membrane was washed with TBS-T (3 × 5 min), and then treated with the appropriate secondary antibody diluted (Table S14) in 5% milk in TBS-Tween (TBS-T, 0.1% Tween), and then incubated for 1 h at rt. The membrane was washed with TBS-T (3 × 5 min). The membrane was then covered with ECL Prime Western Blotting Detection Reagent for 1−3 min before being imaged on a Bio-rad molecular imager. Bio-rad Image Lab 6.1 was used to process the data.

Table S13. Primary antibodies used for Western blotting.

Name	Species	Dilution	Supplier
Strep-tag	Mouse	1:1000	Qiagen/34850
Vinculin	Rabbit	1:10,000	Abcam/129002

Table S14. Secondary antibodies used for Western blotting.

Name	Dilution	Supplier
Anti-Mouse IgG Peroxidase	1:15,000	Sigma/A4416
Anti-Rabbit IgG Peroxidase	1:15,000	Sigma/A6667

Assay procedures

Differential scanning fluorimetry (DSF)

The differential scanning fluorimetry or thermal stability assays were carried out following the protocol described by Niesen *et al.*[21] and data was analysed as described previously.[21] Thermal melting experiments were carried out using the Mx3005p real-time PCR machine (Agilent) and employing a protein concentration of 2 µM for BRD4(1)^{WT} and BRD4(1)^{L94C}. Buffer conditions were 50 mM HEPES buffer, pH 7.5, 500 mM NaCl, 0.5% DMSO and a 1:1000 dilution of SYPRO Orange (Invitrogen, CA) for BRD4(1)^{WT} and BRD4(1)^{L94C}. The 96-well PCR plates were sealed and centrifuged for 2 min at 1000 rpm at 25 °C before measurement. Temperature was raised with a step of 1 °C per 30 s from 25 °C to 85 °C, and fluorescence readings were taken at each interval. Excitation and emission filters for the SYPRO Orange dye were set to 465 and 590 nm, respectively.

Isothermal titration calorimetry (ITC)

Peptide titration

Experiments were performed on a MicroCal PEAQ-ITC (Malvern) and analysed with the MicroCal PEAQ-ITC Analysis software (Malvern 1.1.0.1262) using a single binding site model. The first data point was excluded from the analysis. The proteins were dialysed at 4 °C overnight in a Slide-A-Lyzer MINI Dialysis Device (2000 MWCO; Thermo Scientific Life Technologies) into 50 mM HEPES, 150 mM NaCl; pH 7.4. Proteins were centrifuged to remove aggregates (3 min, 3000 rpm, 4 °C). Protein and peptide concentrations were determined by measuring the absorbance at 280 nm using a NanoDrop Lite spectrophotometer (NanodropR Technologies Inc.). The peptide was supplied by GenScript and were dissolved in Milli-Q water and diluted to the required concentration using dialysis buffer. The cell was stirred at 750 rpm, reference power set to 5 µcal/s and temperature held at 25 °C. After an initial

delay of 60 s, 19 x 2 µL injections (first injection 0.4 µL) were performed with a spacing of 180 s. Protein solutions in the calorimetric cell (420 µL, 50 µM) were titrated with the peptide solutions in the syringe (150 µL, 500 µM).

Table S15. Amino acid sequences of the peptide used in ITC.

Peptide	Amino Acid Sequence
H4KAc$_4$	H_2N-YSGRGK(Ac)GGK(Ac)GLGK(Ac)GGAK(Ac)RHRK-CONH$_2$

Protein LCMS assays

Labeling assay 1

Assays were performed in 1.5 mL Eppendorf tubes at 37 °C and gently shaken, unless otherwise stated. Experiments were performed in a protein LCMS assay buffer, with 10 µM protein, and 50 µM of the desired compound with a final DMSO concentration of 0.5% (Table S16). Time points were taken at 0, 1 and 4 h, unless otherwise stated, where a 20 µL sample of the reaction mix was taken, diluted with 100 µL Milli-Q water, and flash frozen. Samples were thawed immediately prior to LCMS analysis on a Waters Xevo G2-S system (Waters) fitted with a ProSwift™ RP-2H PSDVB Reversed Phase HPLC Column (Thermofisher). Data was analysed and processed using MassLynx. The total ion chromatograms (TIC) were extracted (region containing protein) and the summed scans were deconvoluted (using MaxEnt). Covalent labeling was calculated using intensities of the unlabeled and labeled protein (**Equation 1**). Figures were made using GraphPad Prism 7.0.

Table S16. Buffer composition for protein LCMS.

Buffer	Reagent	Composition	Amount per 1 L
Protein LCMS Assay Buffer (pH 7.4)	HEPES	25 mM	6.0 g
	NaCl	100 mM	5.8 g

Equation 1.

$$Protein\ Labelling = \frac{Intensity\ of\ Labelled\ Peak}{(Intensity\ of\ Unlabelled\ Peak +\ Intensity\ of\ Labelled\ Peak)} \times 100$$

Labeling assay 2

Chloroacetamide (50 µM) or acrylamide (200 µM) fragments were incubated with BRD4(1)^{L94C} (1 µM) in buffer (25 mM HEPES, 50 mM NaCl, pH 7.5) at 4 °C for 24 h. Intact protein masses were recorded by LCMS using an Agilent G6224 time-of-flight (ToF) Accurate Mass Series mass spectrometer, interfaced with an Agilent 1200 series liquid chromatography and sample handling system. The protein

sample was injected using an Agilent 1200 series AutoSampler (Model No. G1367B) with a 10 µL injection volume and maintained at a temperature of 10 °C. Chromatography was carried out on an Agilent Bio-HPLC PLRP-S (1000Å, 5 µm × 50 mm × 1.0 mm, PL1312-1502) reverse phase HPLC column at 70 °C. Using an Agilent 1200 series binary pump system (Model No. G1312B). Data acquisition was carried out in 2 GHz Extended Dynamic range mode. Spectra were processed using Mass Hunter Qualitative Analysis™ B06.00 (Agilent) software with the Maximum Entropy method employed. The total ion chromatograms (TIC) were extracted (region containing protein) and the summed scans were deconvoluted (using a maximum entropy algorithm).[22]

Dose response AlphaScreen™

The peptide (Table S17) was supplied at >95% purity by GenScript and was stored as 1 mM stocks in Milli-Q water at –80 °C.

Table S17. Amino acid sequences of peptides used in AlphaScreen™.

Peptide	Amino Acid Sequence
H4KAc$_4$	H_2N-YSGRGK(Ac)GGK(Ac)GLGK(Ac)GGAK(Ac)RHRK(Biotin)-CONH_2

The assay buffer (Table S18, pH 7.6) was filtered through a 0.22 µm filter before use. The buffer is stored at 4 °C as a 5× solution without bovine serum albumin (BSA), which was added on the day of use. The compounds, proteins, and peptides were dispensed into a ProxiPlate-384 Plus (Perkin Elmer) using a Thermo-Fisher electronic multi-channel pipette. For the incubation steps, the plates were sealed, shaken for 10 seconds at 600 rpm on a plate oscillator, and incubated at room temperature in darkness for 30 min or 3.5 h. Ni^{2+} chelate acceptor and streptavidin donor beads were prepared as a mixture in a 1:300 dilution (0.007 mg/mL final assay concentration, added to the plates and incubated for a further 30 min at room temperature. Plates were read using a SynergyTM 2 MultiMode Microplate Reader using the inbuilt AlphaScreen™ 384 ProxiPlate function: excitation 680 nm, 0.18 sec; emission 570 nm, 0.37 sec.

Table S18. Composition of assay buffer used in AlphaScreen™ experiments, adjusted to pH 7.6 using an aqueous NaOH solution.

Reagent	Composition	Mass (mg) for 50 mL
HEPES	25 mM	297.9
NaCl	100 mM	292.2
CHAPS	0.05% (*w/v*)	25
BSA	0.1% (*w/v*)	50

50 mM DMSO stock solutions of the compounds were serially diluted, and dispensed into wells (5 µL). The protein-peptide mixes (7 µL) for each combination were prepared following the specified final assay concentration (Table S19), and dispensed into wells. Assay beads (8 µL) were added to attain a final assay volume of 20 µL. Dose response curves were obtained for each compound in triplicate, with serial 1:2 dilutions. Data was processed by fitting a four-parameter equation (**Equation 2**) to calculate IC_{50} values using Prism software.

Table S19. Final assay concentrations of each peptide-protein combination.

Protein	Peptide	Protein FAC (nM)	Peptide FAC (nM)
BRD4(1)^{WT}	H4KAc$_4$	10	4
BRD4(1)^{L94C}	H4KAc$_4$	10	4

Equation 2.

$$\text{Response} = \text{Bottom} + \frac{\text{Top} - \text{Bottom}}{1 + 10^{((\text{logIC50} - \text{X}) \times \text{HillSlope})}}$$

X = Log of concentration

Click probe CuAAC efficiency

Assays were performed in 1.5 mL Eppendorf tubes at room temperature, in the dark, and gently shaken, unless otherwise stated. The desired click probe (50 µM) was incubated with TAMRA-PEG$_3$-Azide (200 µM), TCEP·HCl (1 mM), TBTA (100 µM), and $CuSO_4$ (1 mM) in protein LCMS buffer. Time points were taken at 0 and 1 h, unless otherwise stated, where a 25 µL sample of the reaction mix was taken, diluted with 25 µL Milli-Q water, and flash frozen. Samples were thawed immediately prior to LCMS analysis on a Waters LCT Premier bench-top orthogonal acceleration time-of-flight LCMS system. Data was analysed and processed using MassLynx. Figures were made using GraphPad Prism 7.0.

Fluorescent labeling of purified proteins

Assays were performed in 1.5 mL Eppendorf tubes at 37 °C, and gently shaken, unless otherwise stated. The desired click probe (50 µM) was incubated with protein (10 µM) in protein LCMS buffer for 4 h, after which TAMRA-PEG$_3$-Azide (200 µM), TCEP·HCl (1 mM), TBTA (100 µM), and $CuSO_4$ (1 mM) were added and incubated for a further 1 h at room temperature in the dark. A 25 µL sample of the

reaction mix was taken and 8 µL of Lamelli buffer added, and the samples denatured by heating at 95 °C for 10 min. Samples were separated by gel electrophoresis on NuPAGE™ 4–12%, Bis-Tris precast polyacrylamide gels (Thermofisher). Following electrophoresis, gels were stained for 15 min with InstantBlue™ Coomasie® stain (Abcam), destained, and imaged using Bio-rad molecular imager. Bio-rad Image Lab 6.1 was used to process the data.

In-cell target engagement

In-cell labeling by clickable probes

Transfected HEK293T cells at ~90% confluency were incubated for 1 h with a DMSO control (0.5% DMSO final concentration) or a click probe (10 µM, 0.5% DMSO final concentration) diluted in Opti-MEM media.

Cells were then harvested and lysed using the previously described protocol above. The resulting cell lysates were diluted to 2 mg/mL using the previously described protocol above.

Cell lysates (50 µL) were then incubated with TAMRA-PEG$_3$-Azide (200 µM), TCEP·HCl (2 mM), TBTA (200 µM), and $CuSO_4$ (2 mM), for 1 h at rt in the dark. Samples were then prepared and separated by gel electrophoresis using the previously described protocol above.

Gels were imaged for fluorescence using Bio-rad molecular imager and then stained for 15 min with InstantBlue™ Coomasie® stain (Abcam), destained, and imaged using Bio-rad molecular imager. Typically, the very bottom of the gel was removed prior to imaging due to residual, unreacted, TAMRA-PEG$_3$-Azide, which would cause oversaturation and poor image quality.

*OXFBD04 (**3**) Competition assay*

Transfected HEK293T cells at ~90% confluency were incubated for 30 min with a DMSO control or varying concentrations of OXFBD04 diluted in Opti-MEM media. Click probes (10 µM) were then added to the media (0.5% DMSO concentration) and incubated for a further 1 h. Cells were then harvested, processed, and analysed using the previously described protocol above.

Immunoprecipitation

Transfected HEK293T cells at ~90% confluency were incubated for 1 h with a DMSO control (0.5% DMSO final concentration) or a click probe (10 µM, 0.5% DMSO final concentration) diluted in Opti-MEM media. Cells were then harvested and lysed using the previously described protocol above. Samples of the resulting cell lysates were diluted to make a 30 µL input sample at 2 mg/mL, using the previously described protocol above. The remainder of the cell lysates were diluted to 500 µL with 4 °C

IP buffer (0.1% SDS, 1% Triton X-100, 10 mM Tris·HCl pH 8, 200 mM NaCl, 2 mM EDTA, 1× Protease inhibitor cocktail) and 5 µL of Strep-tag antibody (Qiagen/34850) added to each sample and incubated at 4 °C for 16 h. After this time, 20 µL of Protein A Dynabeads (Invitrogen) were added and incubated at 4 °C for a further 5 h. The beads were then washed with RIPA buffer (3 × 1 mL), and wash buffer (10 mM Tris·HCl pH 8, 50 mM NaCl) (1 mL), before being pelleted (1000 ×g, 2 min). The beads were resuspended in lysis buffer (15 µL) and incubated with TAMRA-PEG$_3$-Azide (200 µM), TCEP·HCl (2 mM), TBTA (200 µM), and $CuSO_4$ (2 mM) for 1 h at rt in the dark.

4× Loading buffer (4× LDS sample buffer and 2% β-mercaptoethanol) was added to the IP samples and then heated at 95 °C for 5 min. The beads were pelleted and the IP supernatant transferred to a fresh Eppendorf tube. 4× Loading buffer (4× LDS sample buffer and 2% β-mercaptoethanol) was added to the input samples and then heated at 95 °C for 5 min. Samples were then separated by electrophoresis using the previously described protocol above. The gels were then imaged and stained using the previously described protocol above.

Protein X-ray Crystallography

BRD4(1)^{L94C} Crystallography

BRD4(1)^{L94C} was co-crystallised with OXFBD04 (E5Q/V0R) by the sitting drop vapor diffusion method using high throughput (HT) crystallisation screening methods. All HT screens were performed in CrystalMation Intelli-Plate 96-3 low-profile plates (Hampton Research, HR3-119). Reservoirs and drops were dispensed using an Art Robbins Phoenix automatic liquid handler. Reservoirs contained 80 µL of sparse matrix precipitant solution and crystallisation drops (200 - 300 nL total volume) were placed in each of the three subwells; subwell 1, 200 nL protein : 100 nL well solution; subwell 2, 100 nL protein : 100 nL well solution ; subwell 3, 100 nL protein : 200 nL well solution. Plates were sealed using optically clear Xtra-Clear Advanced Polyolefin StarSeal (StarLab) seals and incubated at 19 °C. Three commercially available precipitant kits (JCSG-plus MD1-37 (Molecular Dynamics), Crystal Screen (HR2-110, 1-50, Hampton), and Crystal Screen 2 (HR2-112, 1-46, Hampton)) were screened for suitable crystallization conditions.

Crystals of the BRD4(1)^{L94C} : OXFBD04 complex were grown by co-crystallisation in conditions containing 0.15 M ammonium sulfate, 0.1 M Bis-Tris, pH 5.5 (sitting drop, protein to-well ratio 2:1) at a protein concentration of 9.0 mg/mL and a final OXFBD04 concentration of 1 mM added directly to the protein prior to crystallization from a 100 mM stock solution in DMSO. Crystals formed after 6 weeks (maximum crystal size 100 x 150 x 150 mm).

The resultant crystals were cryoprotected by soaking crystals in reservoir solution diluted with 25% (*v/v*) glycerol before being flash cooled in liquid nitrogen using nylon loops.

Data collection and processing

Data were collected using a single crystal at Diamond Light Source beamlines i04 Dectris Eiger X 16 detector, respectively. Diffraction data for BRD4(1)^{L94C} : OXFBD04 were autoprocessed by the beamline autoprocessing pipeline using the XIA2 strategy. [23]The diffraction data processed in space group $P\ 2_12_12_1$ (Table S20).

Structure solution and refinement

BRD4(1)^{L94C}:OXFBD04 structure was solved by molecular replacement (MR) using PHASER with a structure of human BRD4 as the search model (PDB ID 6FSY). One molecule was identified in the asymmetric unit. Visual inspection of initial electron density maps indicated bound OXFBD04 inhibitor. The (*S*)- stereoisomer inhibitor was modelled to the density and a round of refinement carried out. After refinement it was clear that the (*R*)- stereoisomer was also present in the crystal and was

included as 50% occupancy in the remaining iterative refinement cycles of model fitting in COOT and refinement using PHENIX the converging R_{work} and R_{free} no longer decreased. Data collection and refinement statistics for all structures can be found in Table S20.

Table S20. Protein crystallography data collection and refinement statistics.

⌘Values in brackets indicate the outermost shell.
$R_{merge} = \sum_j \sum_h | I_{hj} - \langle I_h \rangle | / \sum_j \sum_h \langle I_h \rangle \times 100$.
$R_{work} = \sum ||Fobs| - |Fcalc|| / |Fobs| \times 100$.
R_{free}, based on 4.84% of the total reflections.

**Structure**	**hBRD4 L94C:E5Q/V0R**
PDB ID	8CKF
Data Collection and Processing	
Synchrotron Radiation source	Diamond Beamline I04
Detector	Dectris EIGER X 16M
X-ray Wavelength (Å)	0.9795
Resolution Range (Å)*	39.29-1.88 (1.947-1.88)
Space Group	$P\ 2_1\ 2_1\ 2_1$
Unit Cell Dimensions (*a* Å, *b* Å, *c* Å, α°, β°, γ°)	33.540 46.889 78.577 90 90 90
Total Number of Reflections Observed	134593 (13282)*
Number of Unique Reflections	10563 (1026) *
Multiplicity⌘	12.7 (12.9)*
Completeness (%)⌘	99.46 (98.36) *
$I/\sigma(I)$	6.0 (1.1)*
R_{merge} (%)	0.2288 (2.194) *
CC1/2⌘	0.995 (0.723) *
**Refinement**	
R_{work} (%)	0.2219 (0.3607)
R_{free} (%)	0.2597 (0.3970)
RMS Deviation (Bonds/Angle)	0.002/0.540
Average *B* Factor (Å^2)	36.56
Wilson B Factor (Å^2)	25.63
Number of Water Molecules	65

The structure has been deposited in the RSCB as PDB ID 8CKF. We thank the staff at Diamond Light Source beamline i04-1 visit mx18069-94 for providing beamtime.

Computational Studies

Molecular Dynamics (MD) Simulations

Protein co-ordinates were taken from the crystal structure BRD4(1) (PDB ID: 3SVG). The protein was parameterized using the AMBER99SB-IDLN forcefield.[24] The L94C mutant version of BRD4(1) was generated using the mutation wizard in PyMOL (Version 1.8). All crystallographic water molecules were retained and used the TIP3P (TIP 3-Point) water model.[25] The system was solvated within a dodecahedral box, with a minimum boundary distance of 1.2 nm. Water molecules were substituted with a sodium ion to neutralize the net charge and to maintain an overall salt concentration of 150 nM sodium chloride.

The systems were subject to energy minimization using the steepest decent algorithm, with a maximum force cut off of 100 kJ mol^{-1} nm^{-1}. They then continued equilibration under the NVT and NPT ensembles for a total of 200 ps. Simulations were then carried out for 50 ns using GROMACS 2016.4, in triplicate.[26]

Ligand Docking Studies

Docking was performed on a *holo* structure of BRD4(1) (PDB ID: 6CZV). All organic molecules and crystallographic waters outside of the binding site were subsequently removed before the protein structure was checked using the WHAT-IF web-interface.[27] The protein was protonated at pH 7.4 and the water network optimized using the MOE software package (v. 2018.2). Finally the structure was converted to the required PDBQT file type using AutoDockTools within the MGLTools package (v. 1.5.6). Ligand models were protonated and given an initial geometry using cxcalc from ChemAxon (https://www.chemaxon.com) (v. 16.7.11.0), before conversion to the PDBQT file type using the prepare_ligand4.py script available within the MGLTools package.[28,29] Molecular docking was performed using AutoDock4,[29] with 2,500,000 energy evaluations and 100 poses generated. Docked ligands were clustered using a 2 Å cut-off and underwent manual inspection.

NMR Spectra for Novel Compounds

N-Methoxy-*N*-3-dimethylisoxazole-5-carboxamide (50)

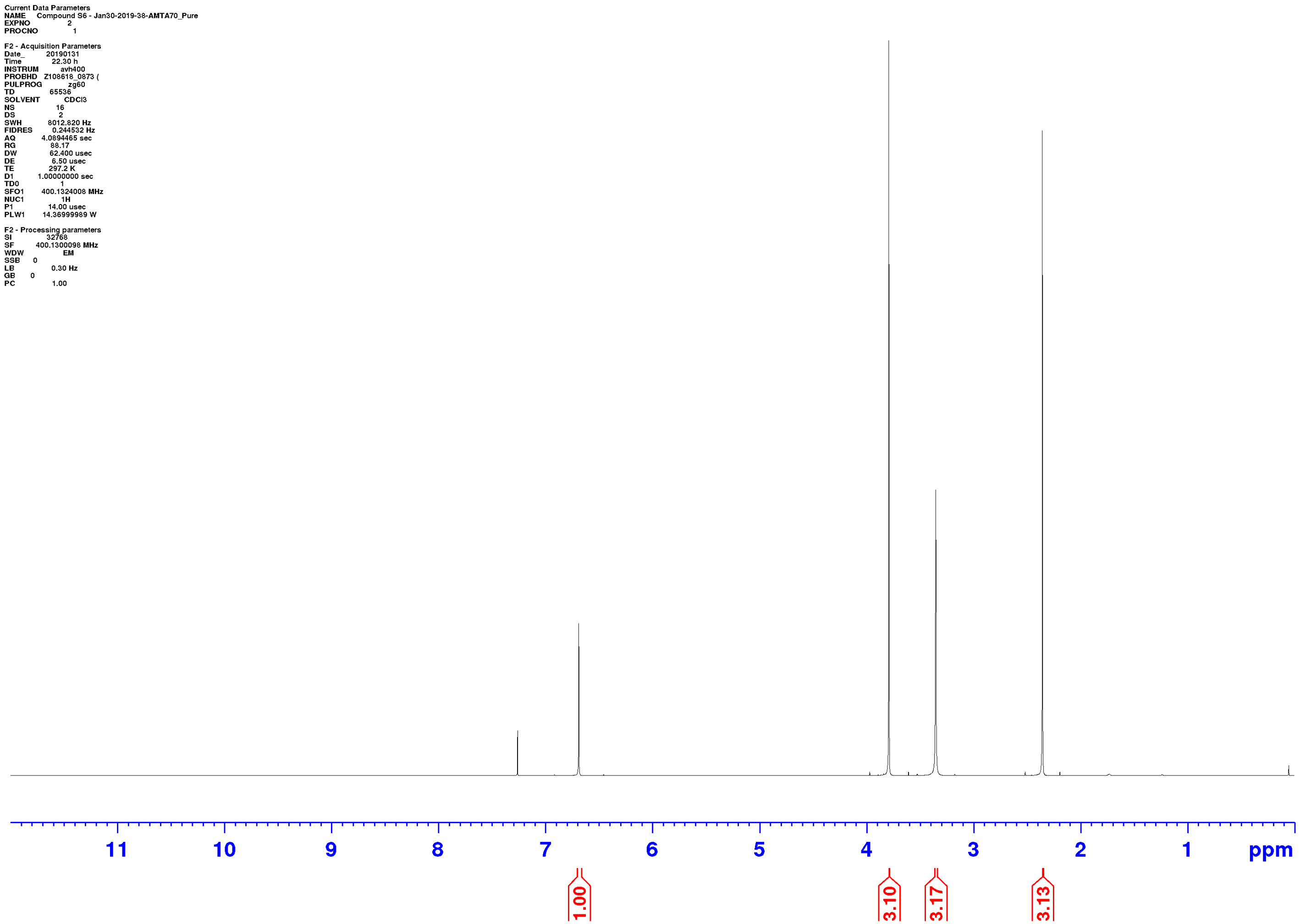

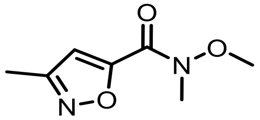

N-Methoxy-*N*-3-dimethylisoxazole-5-carboxamide (50)

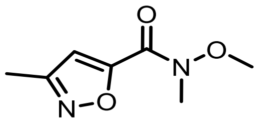

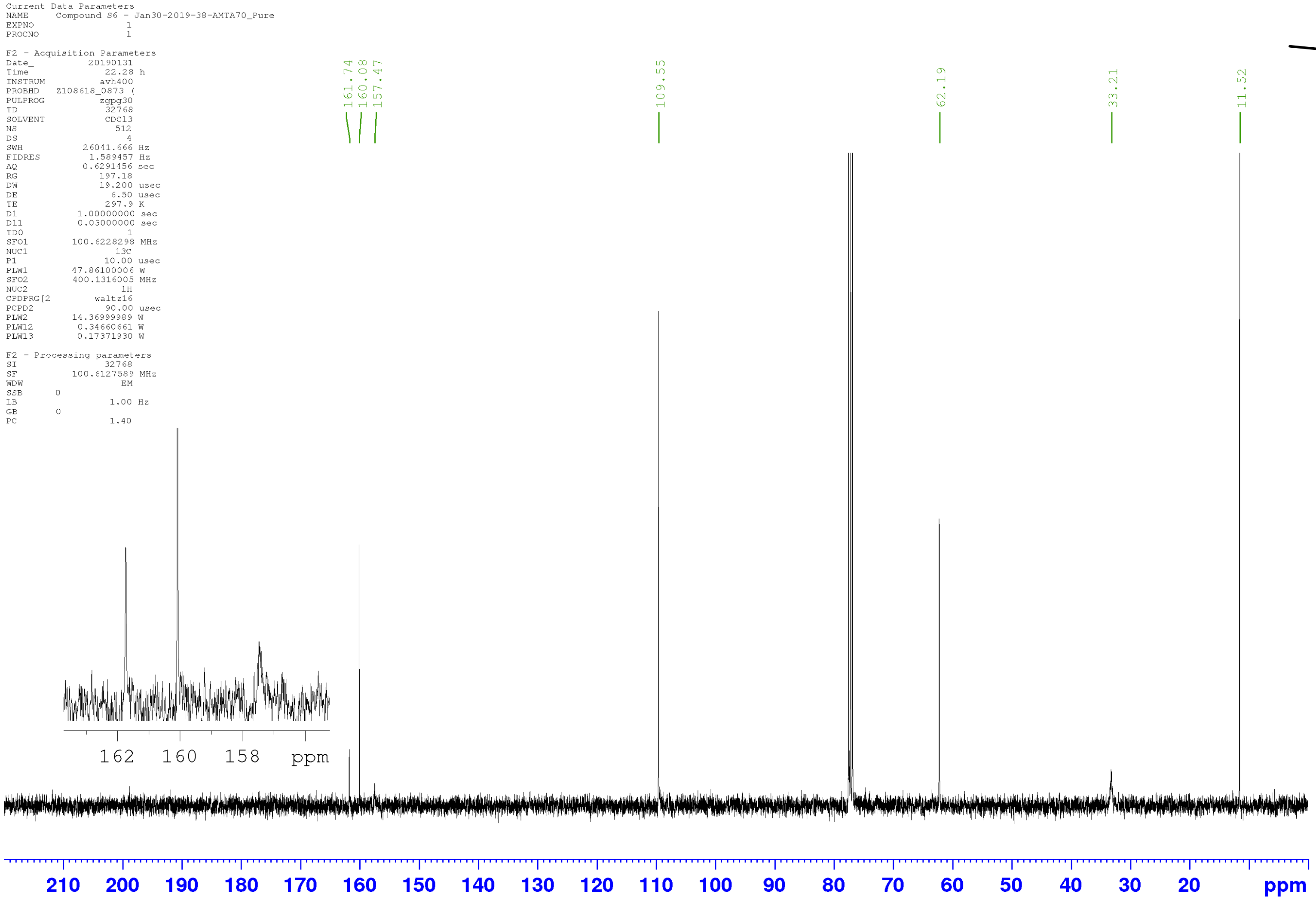

1-(3-Methylisoxazol-5-yl)prop-2-en-1-one (4)

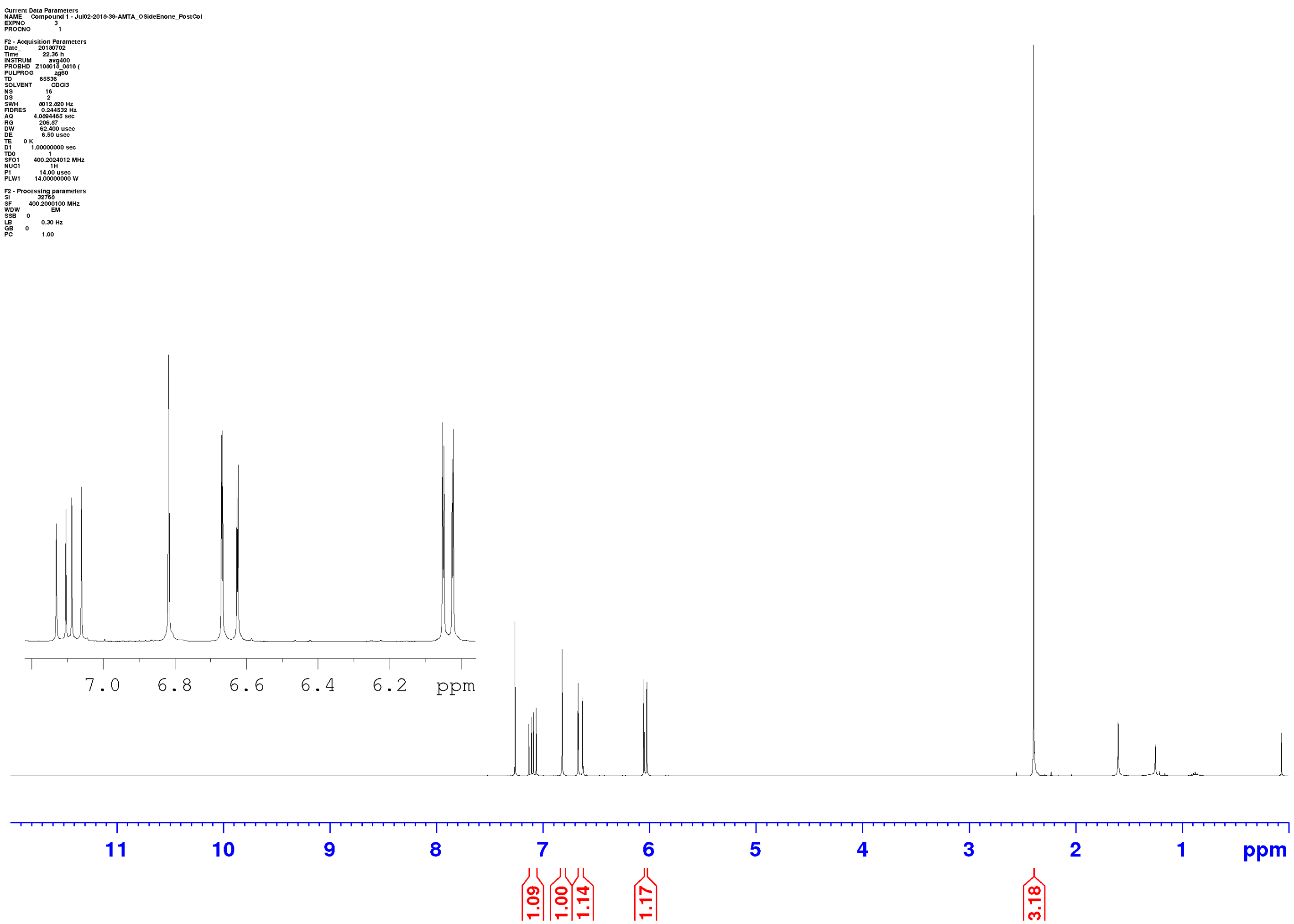

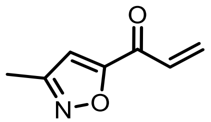

1-(3-Methylisoxazol-5-yl)prop-2-en-1-one (4)

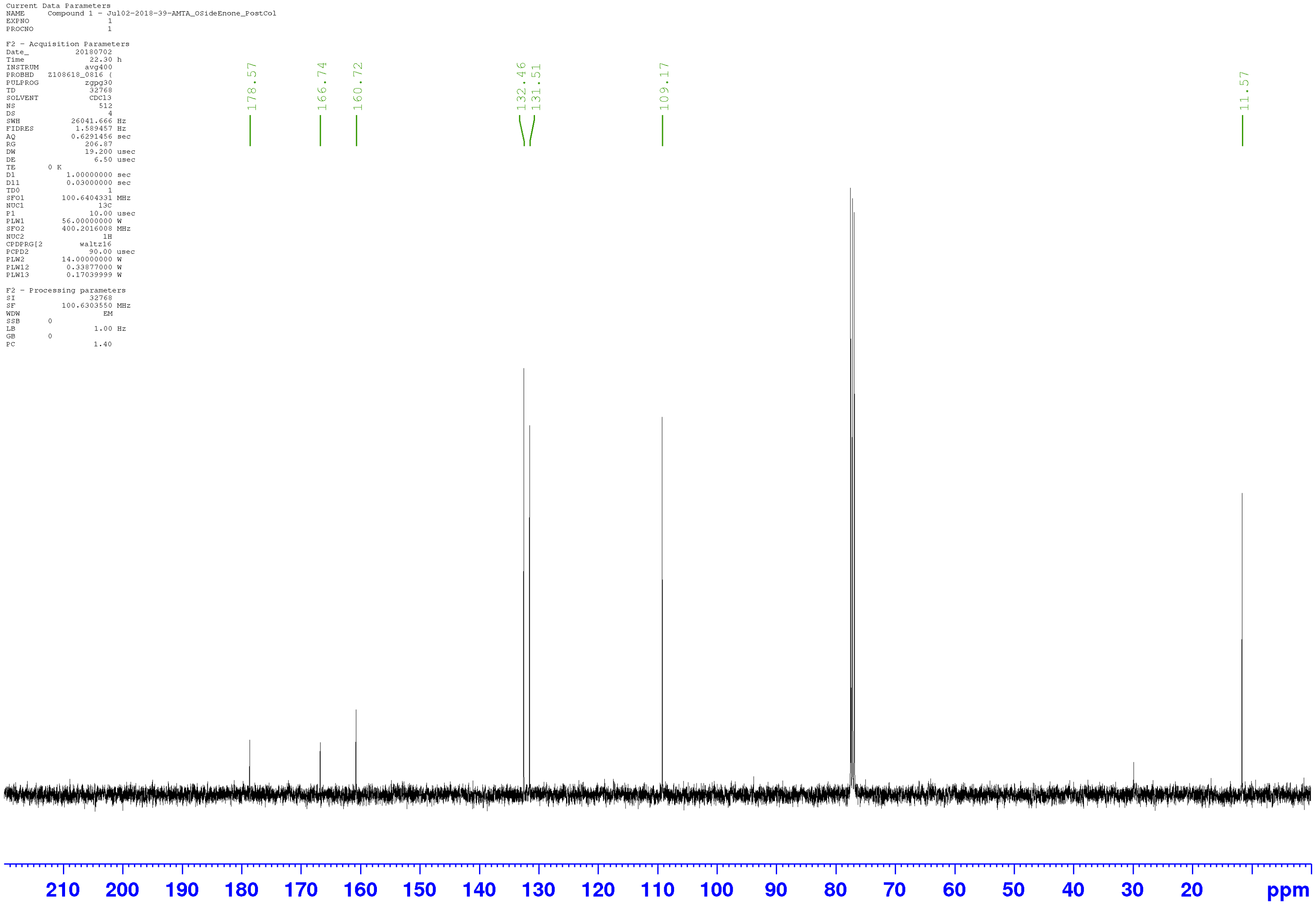

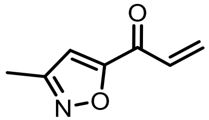

N-Methoxy-*N*-5-dimethylisoxazole-3-carboxamide (53)

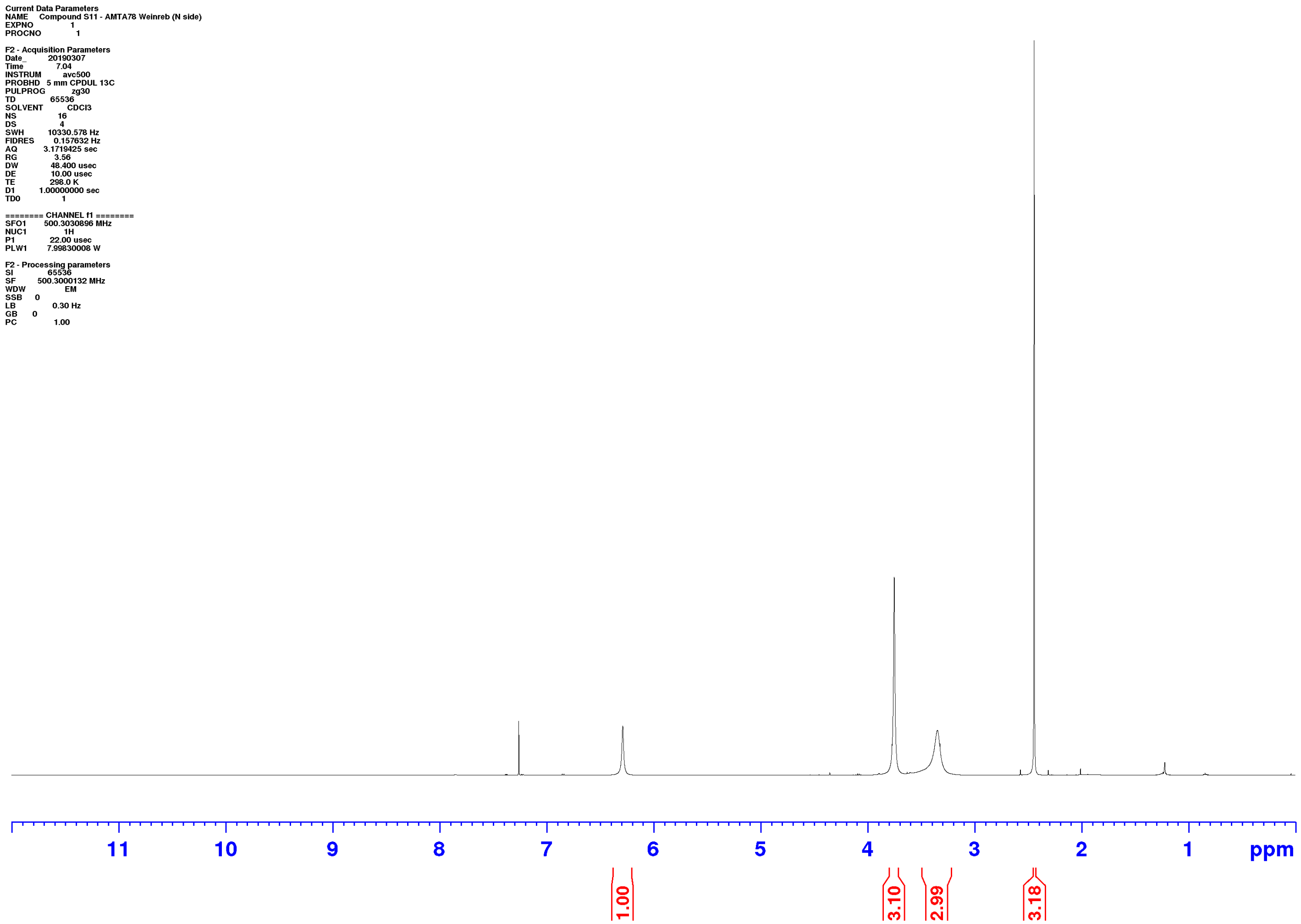

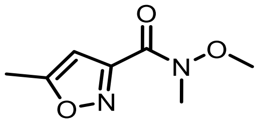

N-Methoxy-*N*-5-dimethylisoxazole-3-carboxamide (53)

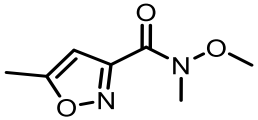

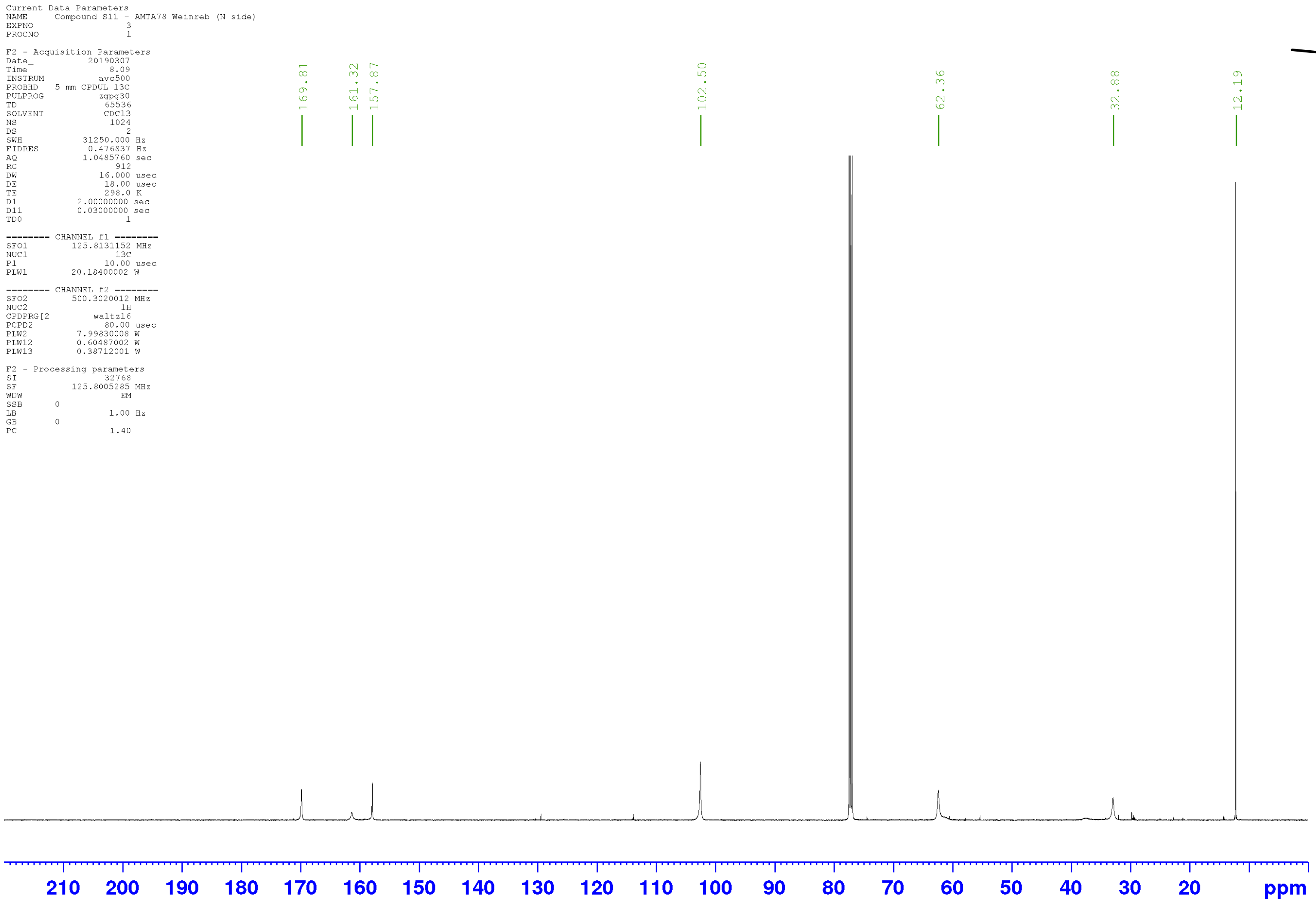

1-(5-Methylisoxazol-3-yl)prop-2-en-1-one (5)

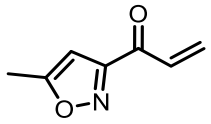

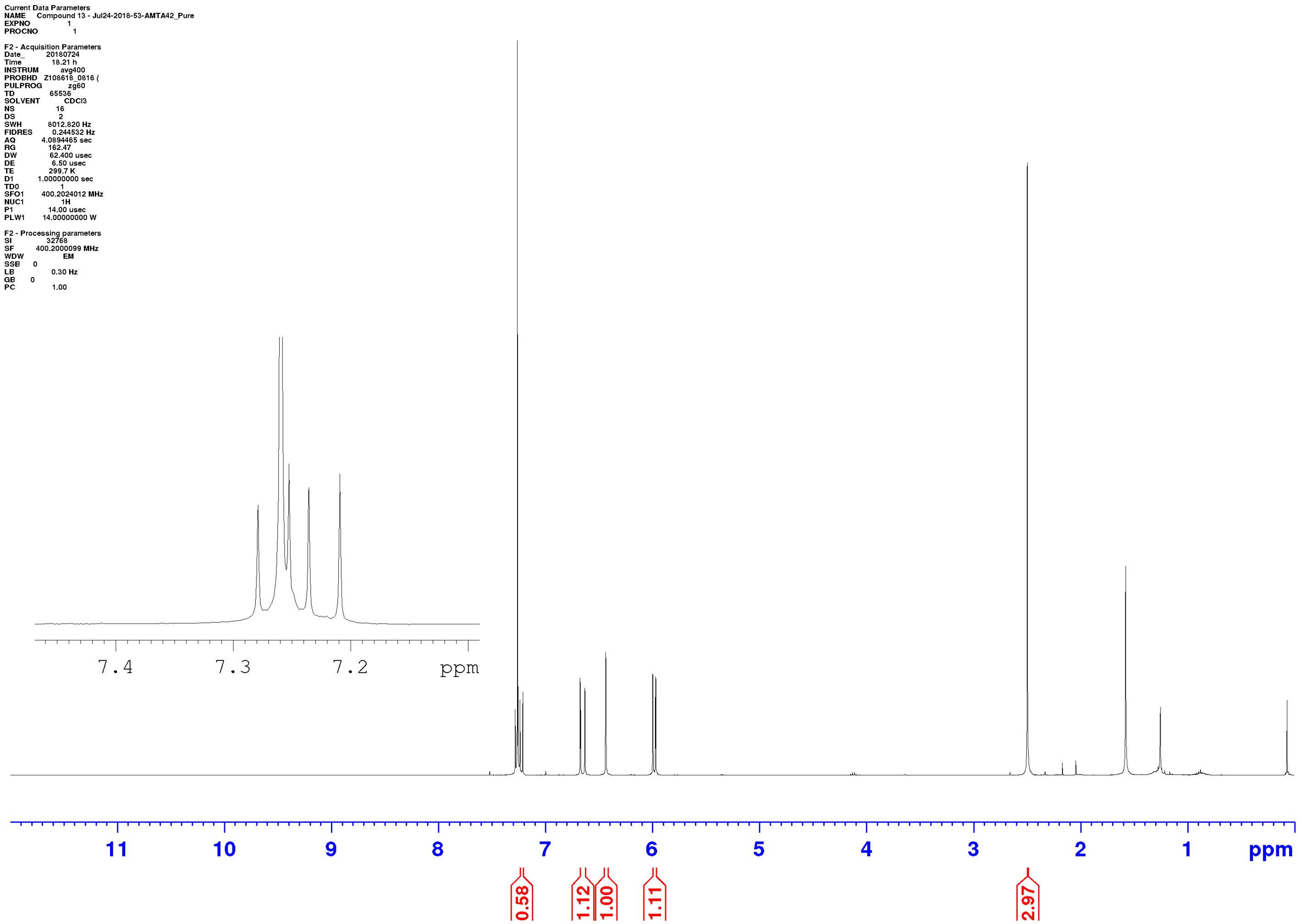

1-(5-Methylisoxazol-3-yl)prop-2-en-1-one (5)

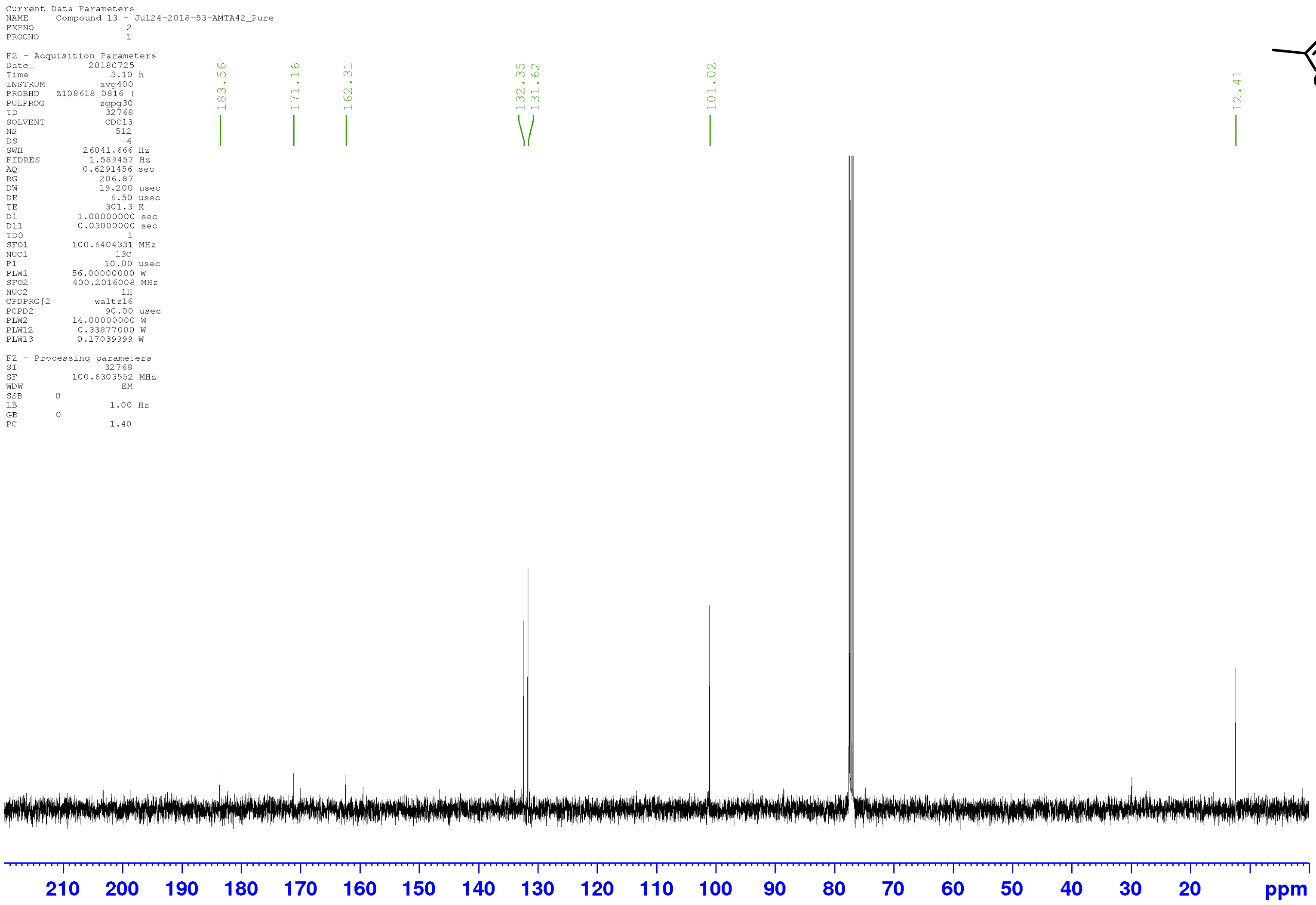

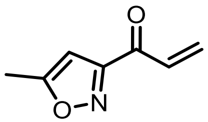

3-Methyl-4-phenylisoxazole-5-carboxylic acid (57)

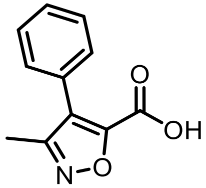

Current Data Parameters
NAME at73421612
EXPNO 1
PROCNO 1

F2 - Acquisition Parameters
Date_ 20191219
Time 21.47
INSTRUM avc500
PROBHD 5 mm CPDUL 13C
PULPROG zg30
TD 65536
SOLVENT CDCl3
NS 16
DS 4
SWH 10330.578 Hz
FIDRES 0.157632 Hz
AQ 3.1719425 sec
RG 4
DW 48.400 usec
DE 10.00 usec
TE 298.0 K
D1 1.00000000 sec
TD0 1

======== CHANNEL f1 ========
SFO1 500.3030896 MHz
NUC1 1H
P1 22.00 usec
PLW1 7.99830008 W

F2 - Processing parameters
SI 65536
SF 500.3000133 MHz
WDW EM
SSB 0
LB 0.30 Hz
GB 0
PC 1.00

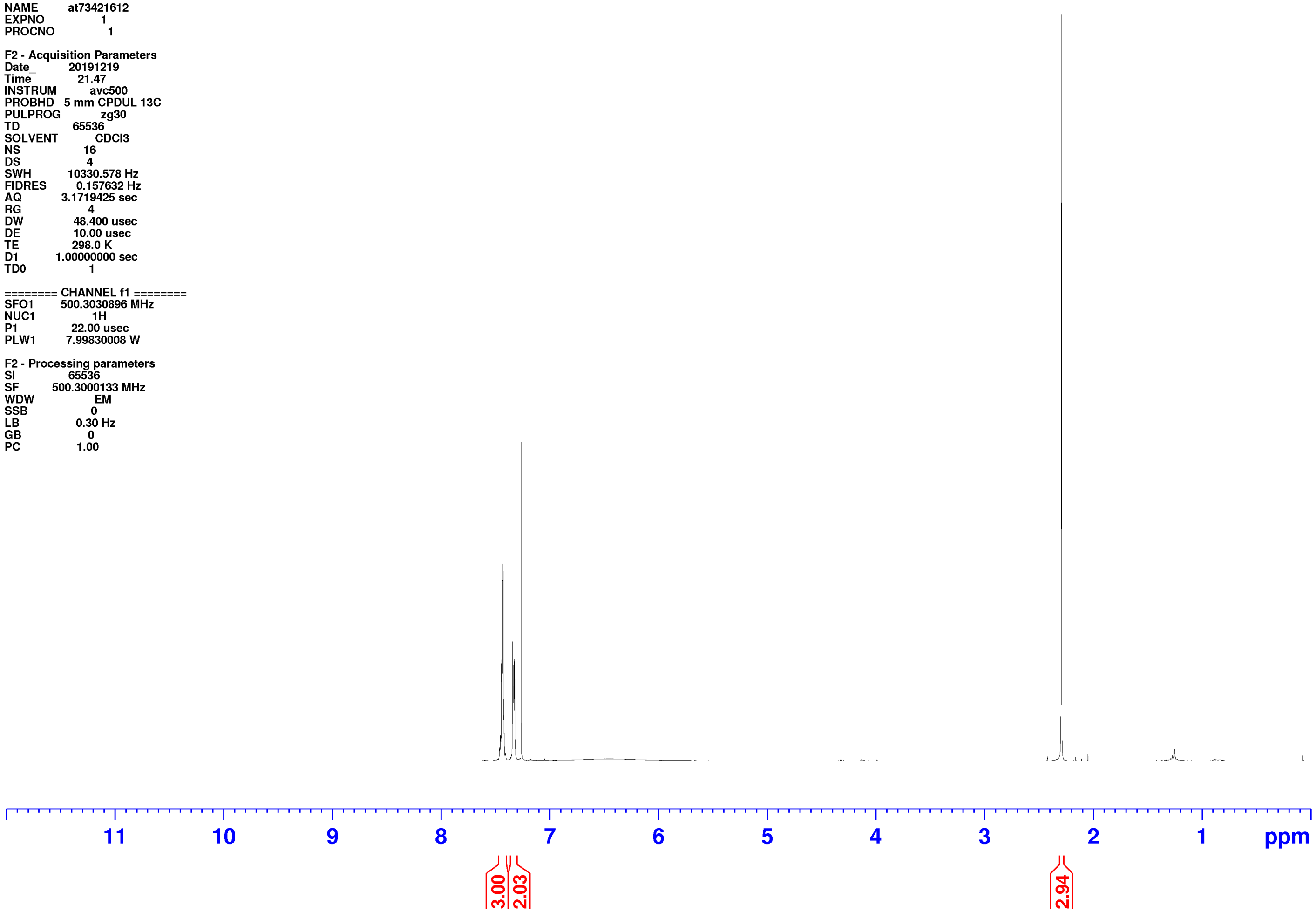

3-Methyl-4-phenylisoxazole-5-carboxylic acid (57)

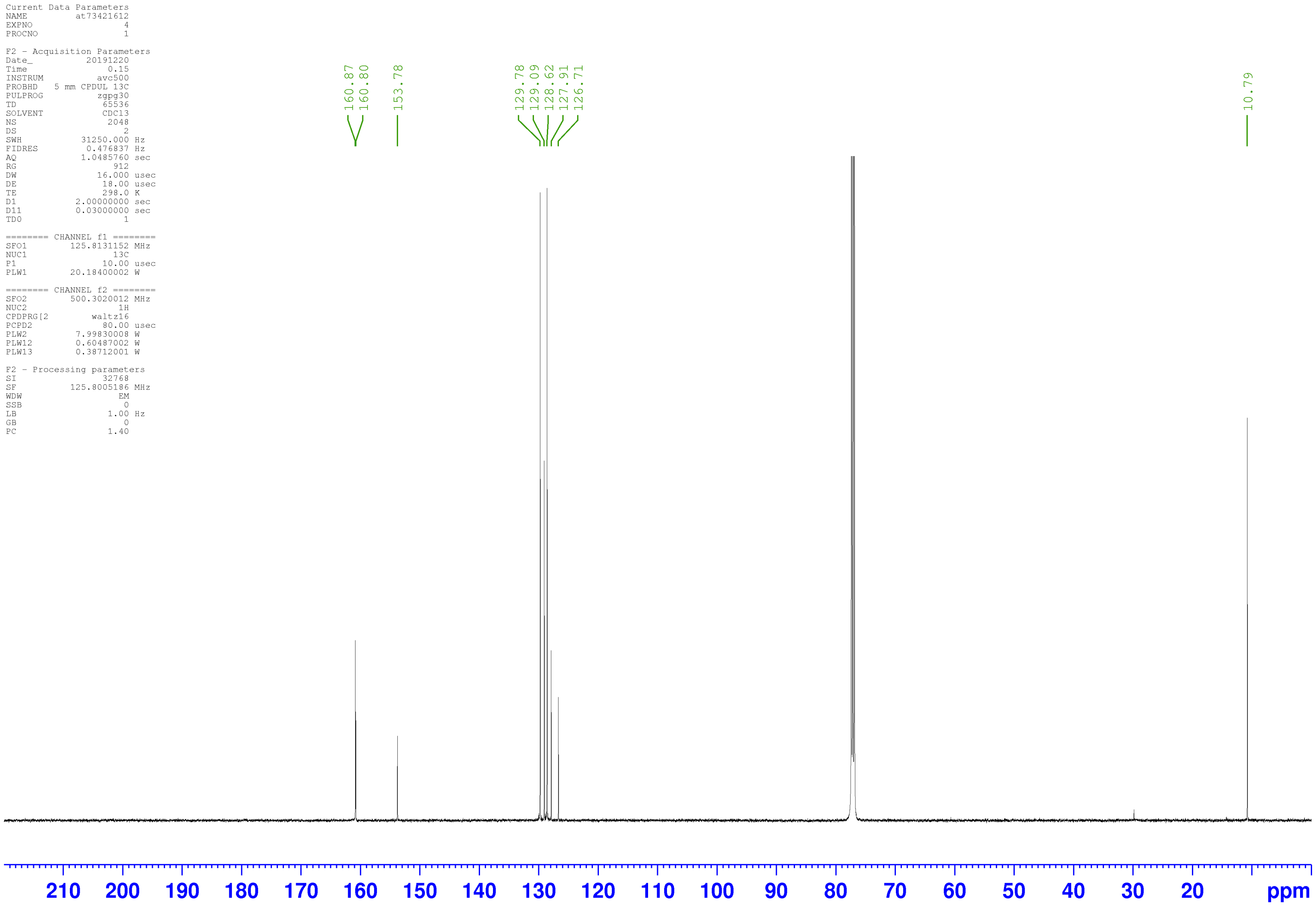

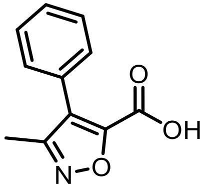

N-Methoxy-*N*,3-dimethyl-4-phenylisoxazole-5-carboxamide (58)

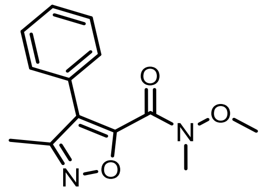

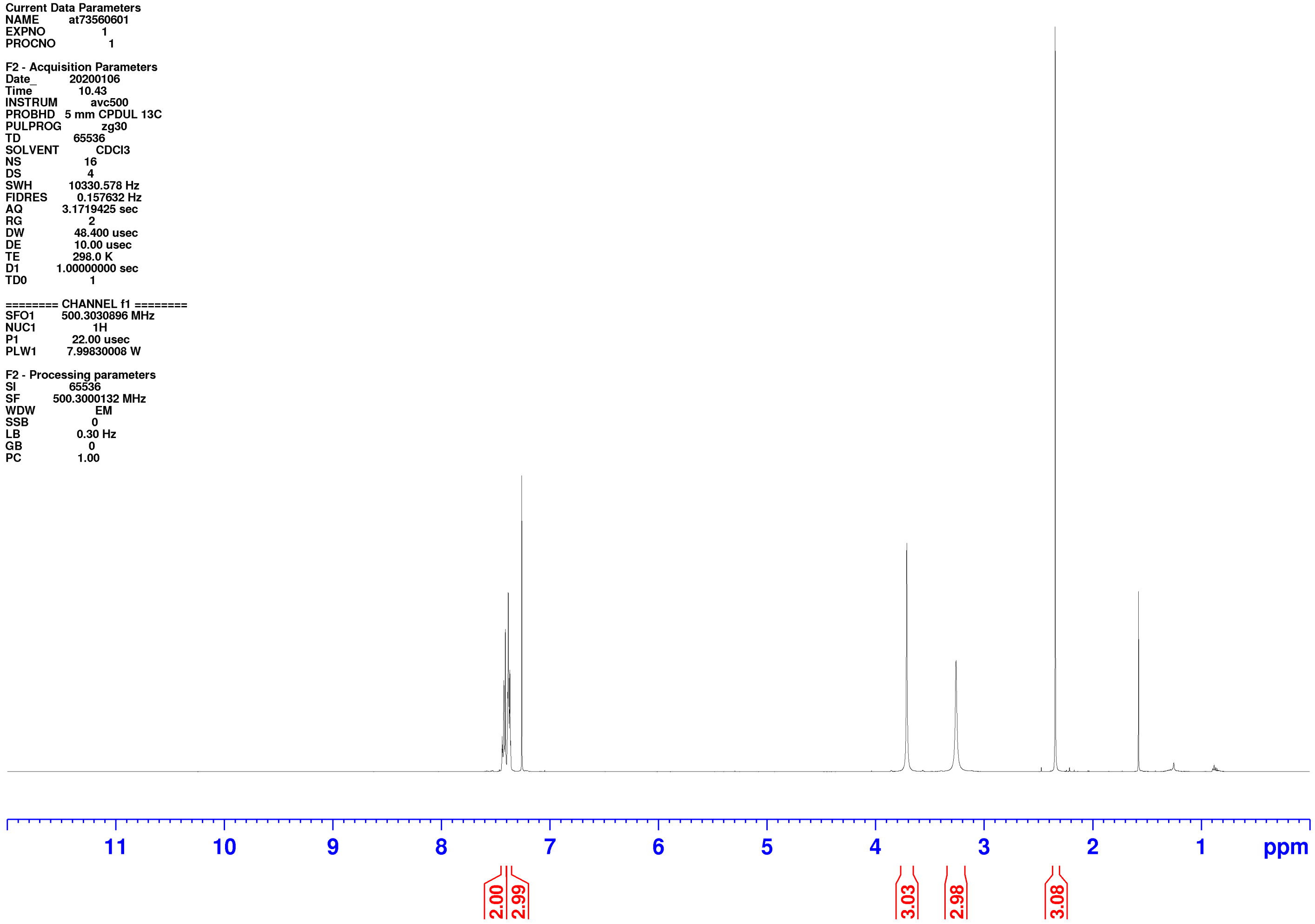

N-Methoxy-*N*,3-dimethyl-4-phenylisoxazole-5-carboxamide (58)

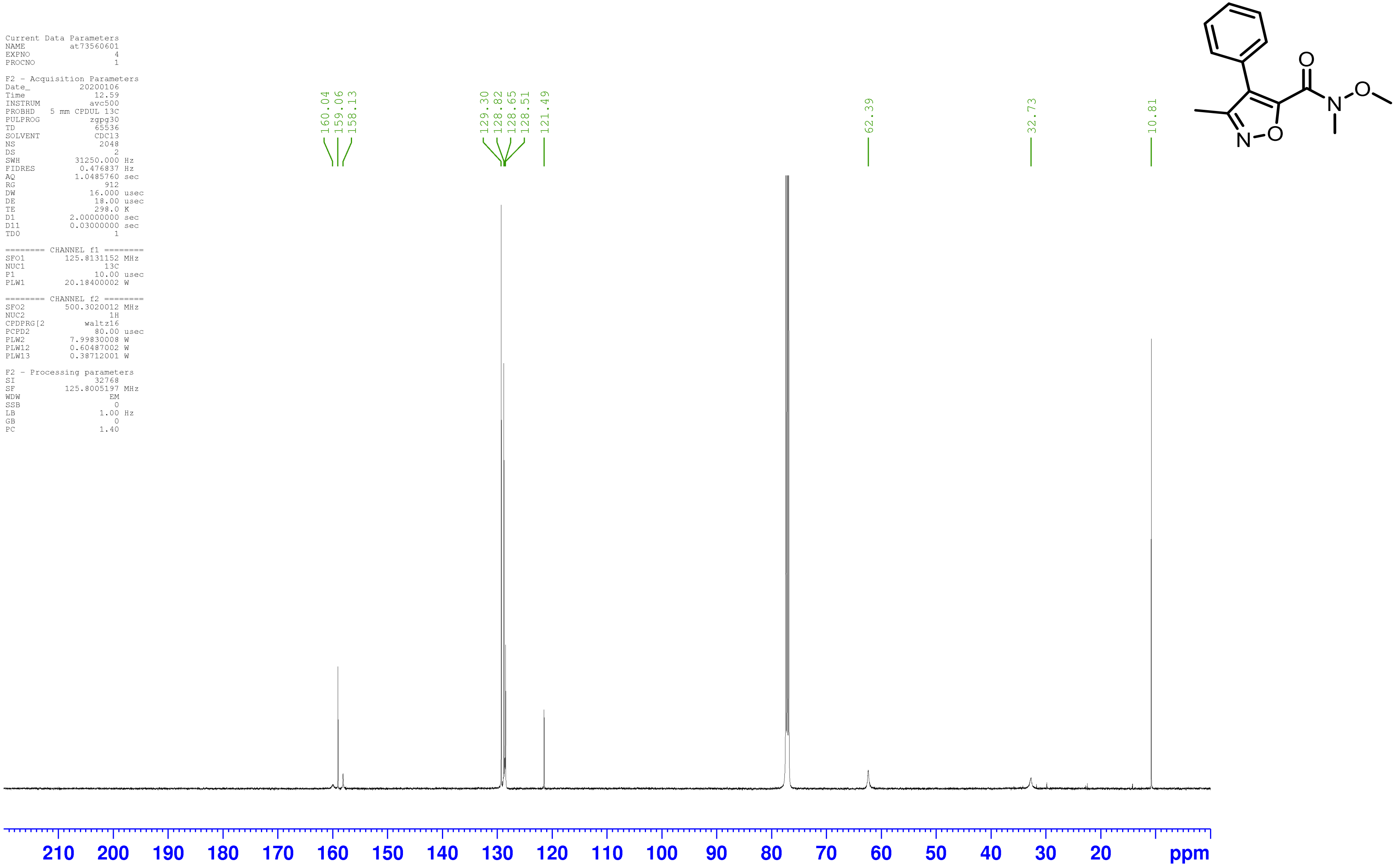

1-(3-Methyl-4-phenylisoxazol-5-yl)prop-2-en-1-one (6)

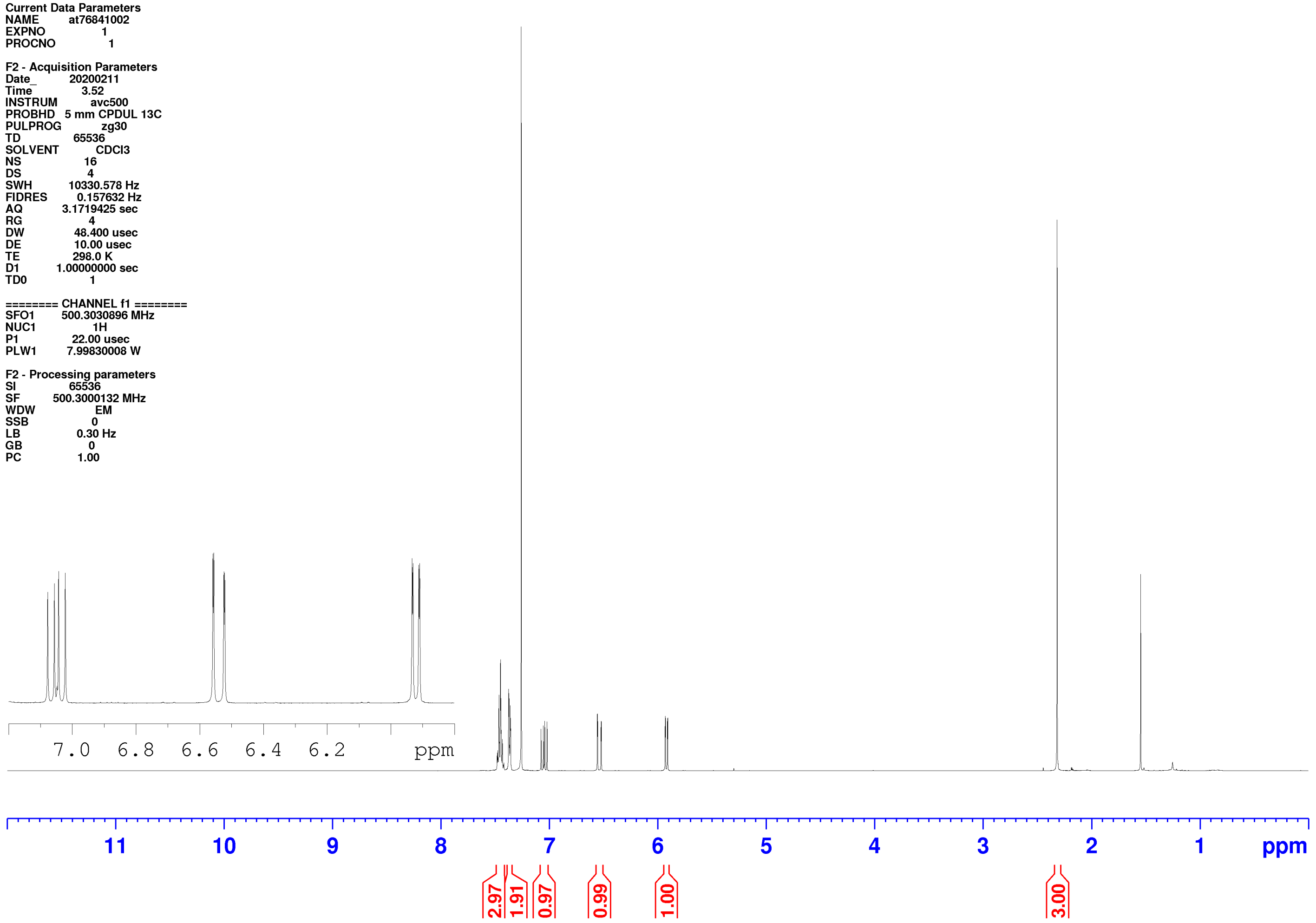

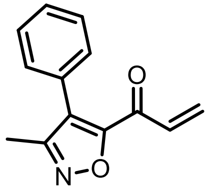

1-(3-Methyl-4-phenylisoxazol-5-yl)prop-2-en-1-one (6)

```
Current Data Parameters
NAME          at76841002
EXPNO                  4
PROCNO                 1

F2 - Acquisition Parameters
Date_           20200211
Time                5.06
INSTRUM           avc500
PROBHD   5 mm CPDUL 13C
PULPROG          zgpg30
TD                 65536
SOLVENT            CDCl3
NS                  1024
DS                     2
SWH            31250.000 Hz
FIDRES          0.476837 Hz
AQ             1.0485760 sec
RG                   912
DW                16.000 usec
DE                 18.00 usec
TE                 298.0 K
D1            2.00000000 sec
D11           0.03000000 sec
TD0                    1

======== CHANNEL f1 ========
SFO1        125.8131152 MHz
NUC1                 13C
P1                 10.00 usec
PLW1         20.18400002 W

======== CHANNEL f2 ========
SFO2        500.3020012 MHz
NUC2                  1H
CPDPRG[2         waltz16
PCPD2              80.00 usec
PLW2          7.99830008 W
PLW12         0.60487002 W
PLW13         0.38712001 W

F2 - Processing parameters
SI                 32768
SF           125.8005187 MHz
WDW                   EM
SSB                    0
LB                  1.00 Hz
GB                     0
PC                  1.40
```

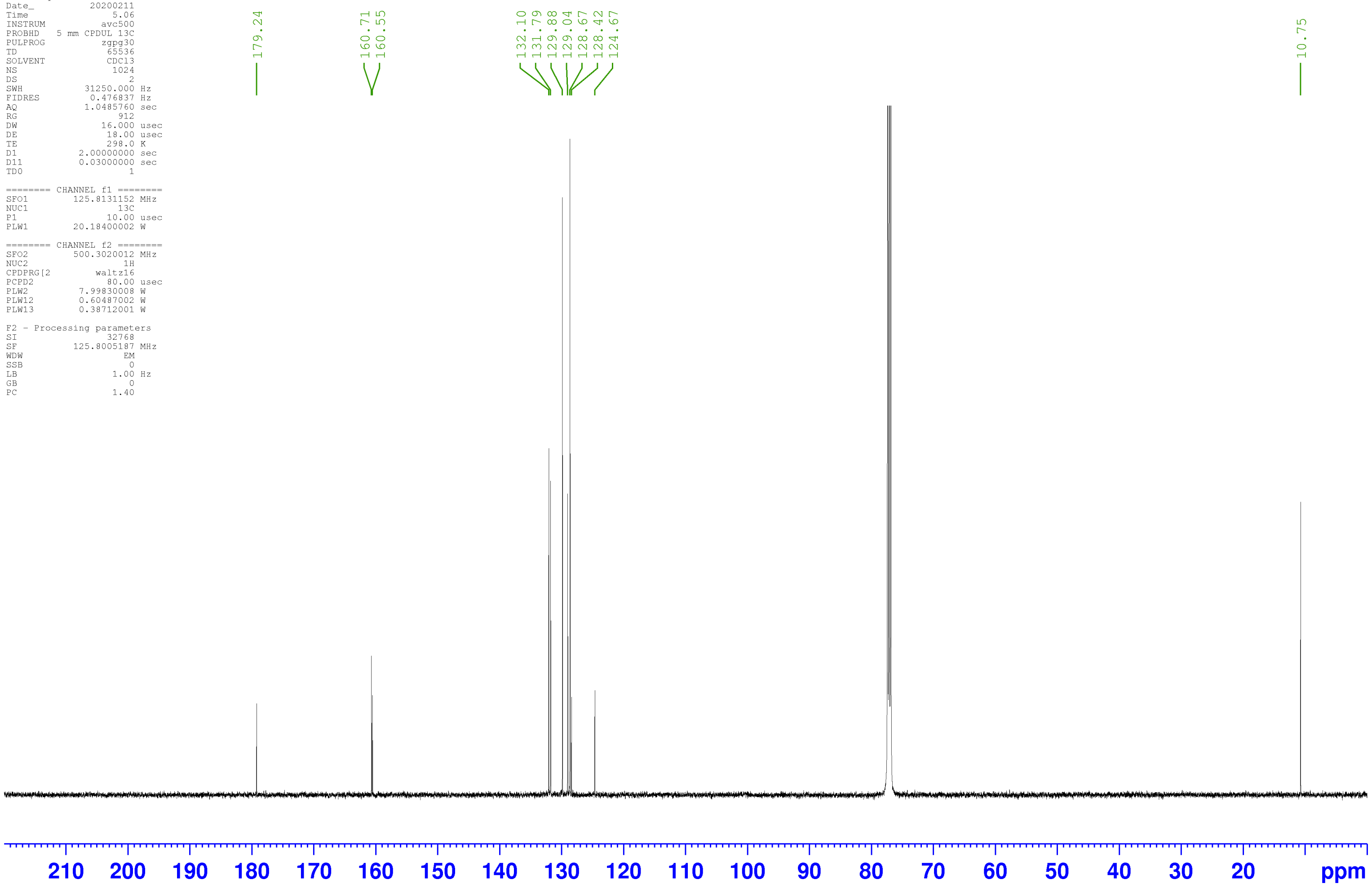

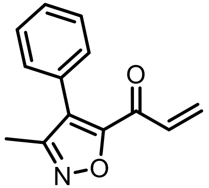

E-3-[Methoxy(methyl)amino]-1-(3-methylisoxazol-5-yl)prop-2-en-1-one (7)

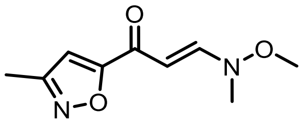

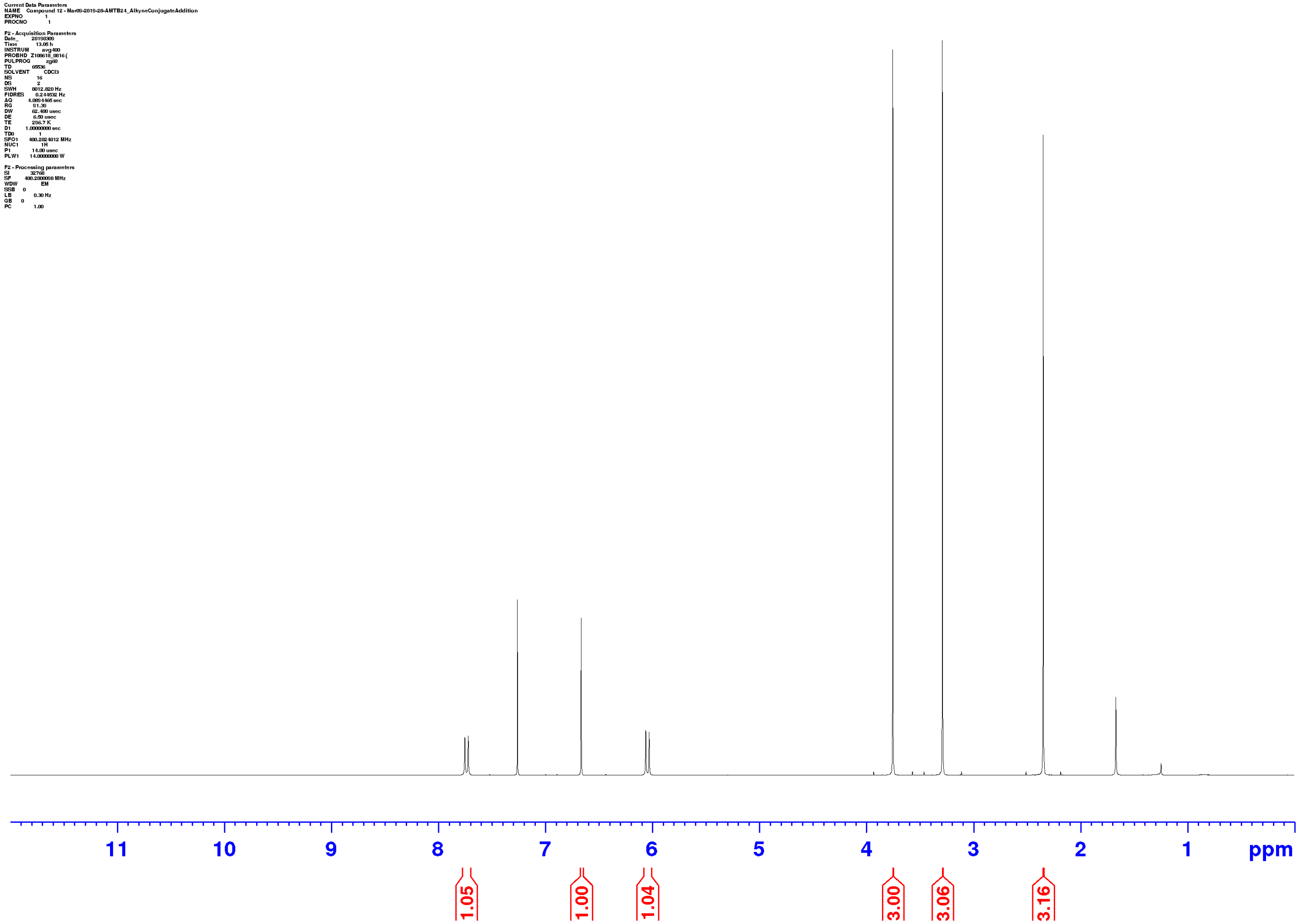

E-3-[Methoxy(methyl)amino]-1-(3-methylisoxazol-5-yl)prop-2-en-1-one (7)

```
Current Data Parameters
NAME     Compound 12 - Mar09-2019-28-AMTB24_AlkyneConjugateAdditio
EXPNO                    2
PROCNO                   1

F2 - Acquisition Parameters
Date_             20190309
Time                 13.21 h
INSTRUM             avg400
PROBHD   Z108618_0816 (
PULPROG            zgpg30
TD                   32768
SOLVENT              CDCl3
NS                     512
DS                       4
SWH              26041.666 Hz
FIDRES            1.589457 Hz
AQ               0.6291456 sec
RG                  206.87
DW                  19.200 usec
DE                    6.50 usec
TE                   297.8 K
D1              1.00000000 sec
D11             0.03000000 sec
TD0                      1
SFO1           100.6404331 MHz
NUC1                   13C
P1                   10.00 usec
PLW1           56.00000000 W
SFO2           400.2016008 MHz
NUC2                    1H
CPDPRG[2           waltz16
PCPD2                90.00 usec
PLW2           14.00000000 W
PLW12           0.33877000 W
PLW13           0.17039999 W

F2 - Processing parameters
SI                   32768
SF             100.6303579 MHz
WDW                     EM
SSB      0
LB                    1.00 Hz
GB       0
PC                    1.40
```

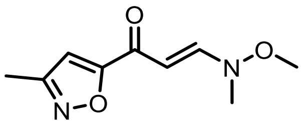

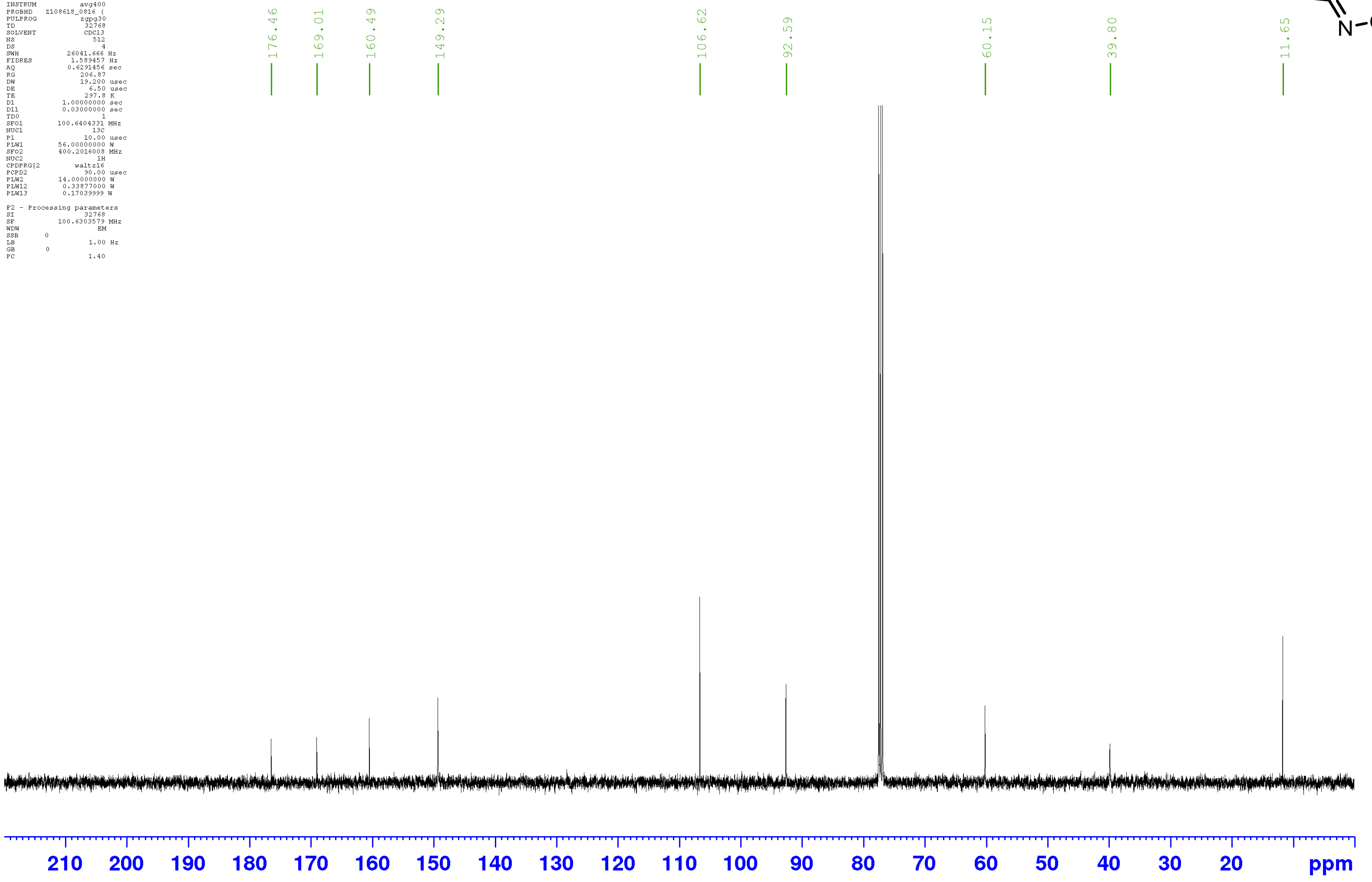

1-(3-Methylisoxazol-5-yl)prop-2-yn-1-one (8)

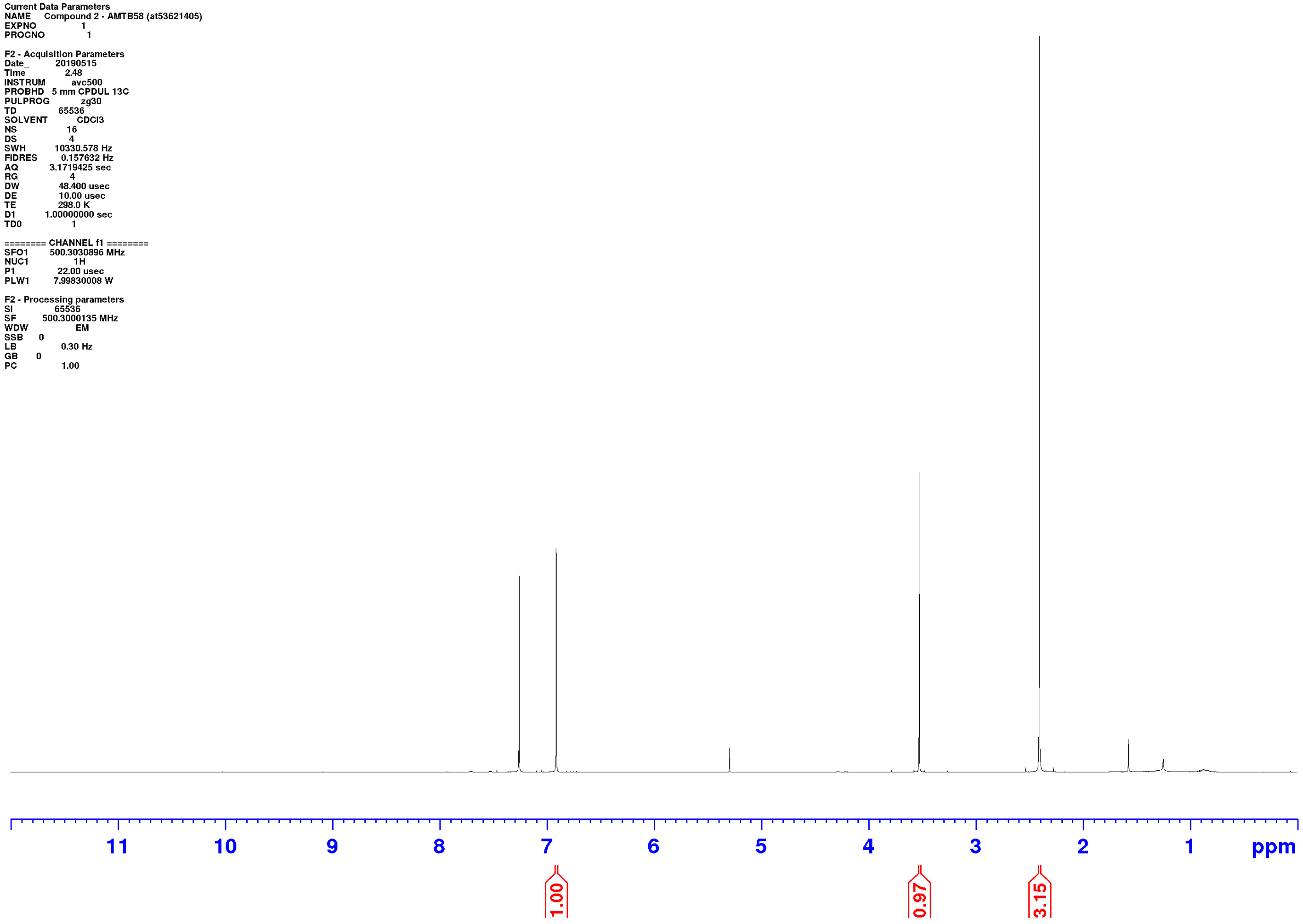

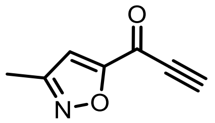

1-(3-Methylisoxazol-5-yl)prop-2-yn-1-one (8)

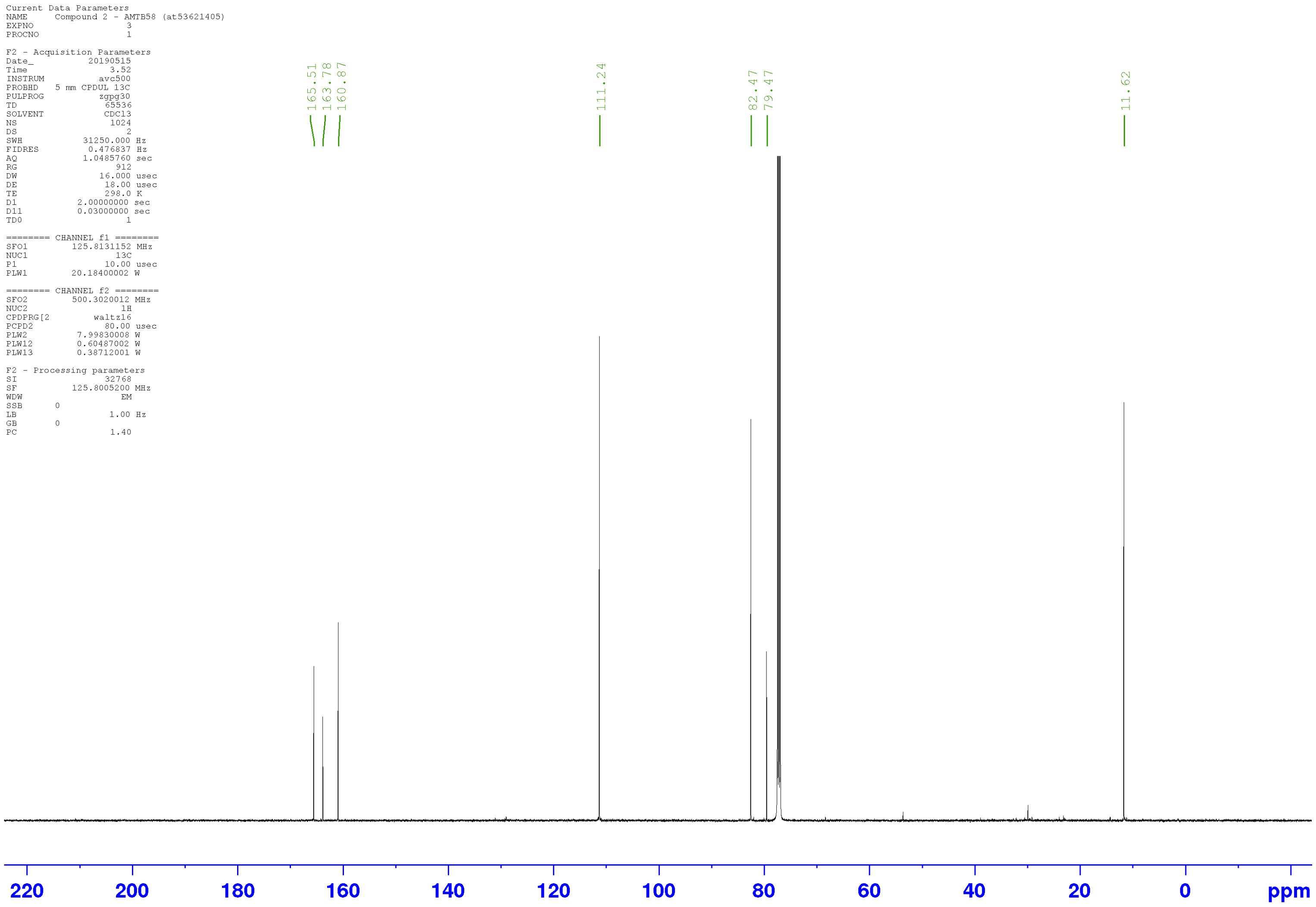

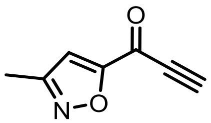

1-(5-Methylisoxazol-3-yl)-3-(trimethylsilyl)prop-2-yn-1-one (54)

Current Data Parameters
NAME Compound S12 - Apr02-2019-20-AMTB37
EXPNO 1
PROCNO 1

F2 - Acquisition Parameters
Date_ 20190403
Time 5.12 h
INSTRUM avg400
PROBHD Z108618_0816 (
PULPROG zg60
TD 65536
SOLVENT CDCl3
NS 16
DS 2
SWH 8012.820 Hz
FIDRES 0.244532 Hz
AQ 4.0894465 sec
RG 91.39
DW 62.400 usec
DE 6.50 usec
TE 298.5 K
D1 1.00000000 sec
TD0 1
SFO1 400.2024012 MHz
NUC1 1H
P1 14.00 usec
PLW1 14.00000000 W

F2 - Processing parameters
SI 32768
SF 400.2000097 MHz
WDW EM
SSB 0
LB 0.30 Hz
GB 0
PC 1.00

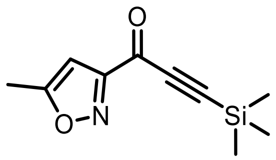

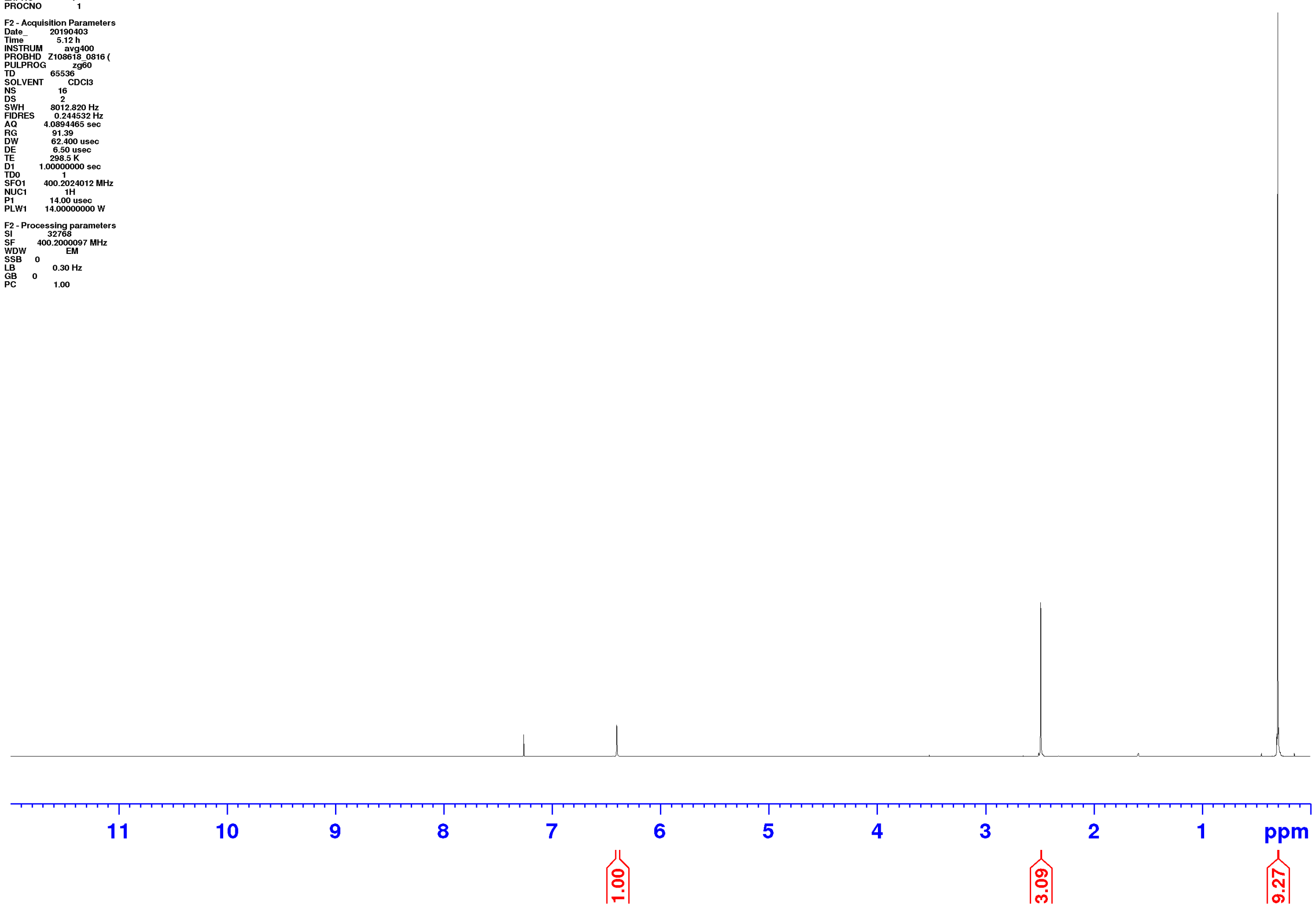

1-(5-Methylisoxazol-3-yl)-3-(trimethylsilyl)prop-2-yn-1-one (54)

```
Current Data Parameters
NAME     Compound S12 - Apr02-2019-20-AMTB37
EXPNO                  2
PROCNO                 1

F2 - Acquisition Parameters
Date_          20190403
Time               5.28 h
INSTRUM          avg400
PROBHD   Z108618_0816 (
PULPROG          zgpg30
TD                32768
SOLVENT           CDCl3
NS                  512
DS                    4
SWH          26041.666 Hz
FIDRES        1.589457 Hz
AQ           0.6291456 sec
RG               206.87
DW               19.200 usec
DE                 6.50 usec
TE                299.4 K
D1           1.00000000 sec
D11          0.03000000 sec
TD0                   1
SFO1        100.6404331 MHz
NUC1                13C
P1                10.00 usec
PLW1        56.00000000 W
SFO2        400.2016008 MHz
NUC2                 1H
CPDPRG[2        waltz16
PCPD2             90.00 usec
PLW2        14.00000000 W
PLW12        0.33877000 W
PLW13        0.17039999 W

F2 - Processing parameters
SI                32768
SF          100.6302852 MHz
WDW                  EM
SSB      0
LB                 1.00 Hz
GB       0
PC                 1.40
```

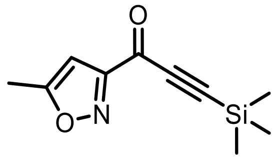

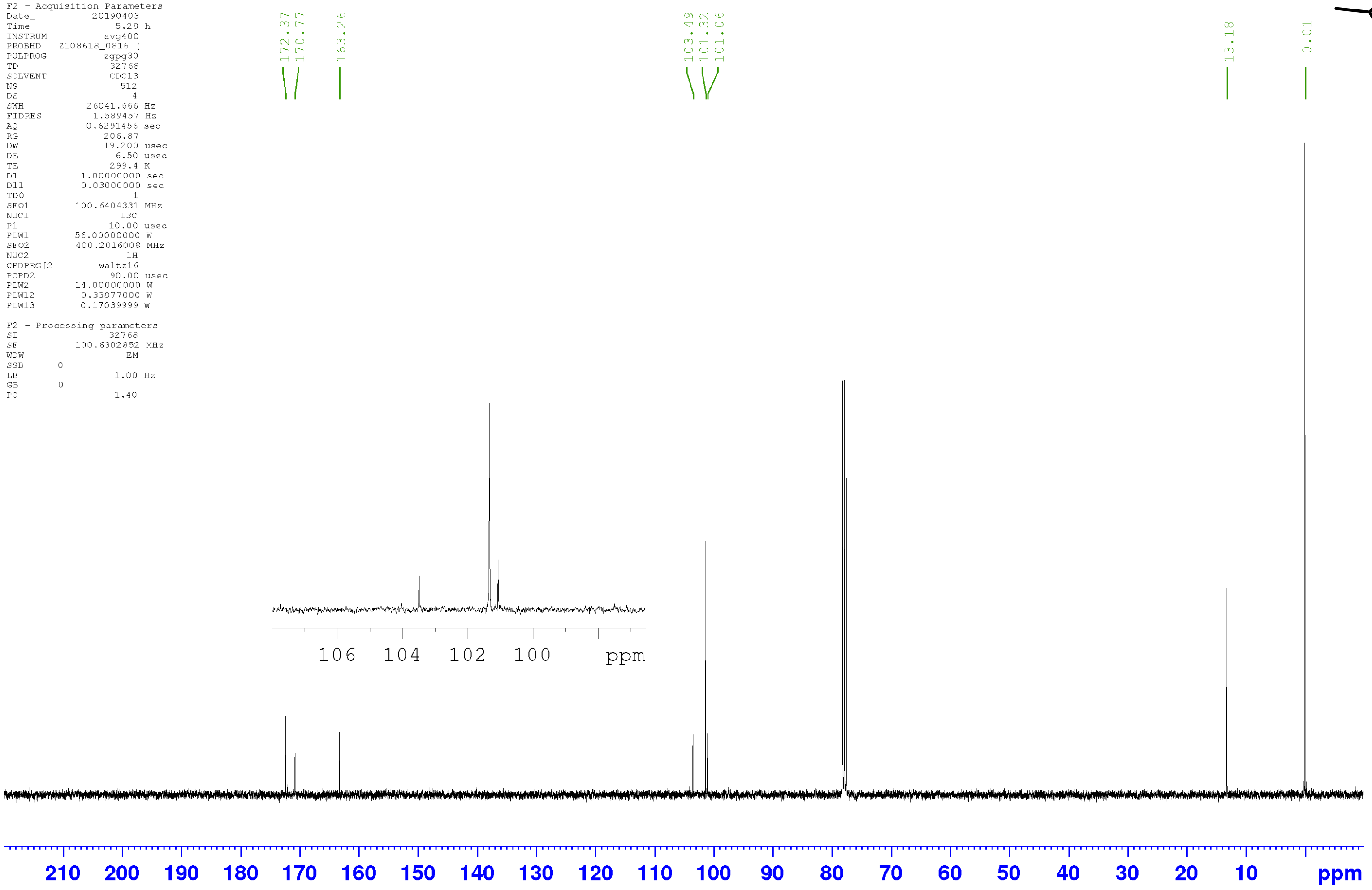

1-(5-Methylisoxazol-3-yl)prop-2-yn-1-one (9)

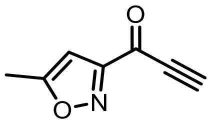

Current Data Parameters
NAME Compound 14 - AMTB51 (at51161804)
EXPNO 1
PROCNO 1

F2 - Acquisition Parameters
Date_ 20190421
Time 8.34
INSTRUM avc500
PROBHD 5 mm CPDUL 13C
PULPROG zg30
TD 65536
SOLVENT CDCl3
NS 16
DS 4
SWH 10330.578 Hz
FIDRES 0.157632 Hz
AQ 3.1719425 sec
RG 4
DW 48.400 usec
DE 10.00 usec
TE 298.0 K
D1 1.00000000 sec
TD0 1

======== CHANNEL f1 ========
SFO1 500.3030896 MHz
NUC1 1H
P1 22.00 usec
PLW1 7.99830008 W

F2 - Processing parameters
SI 65536
SF 500.3000132 MHz
WDW EM
SSB 0
LB 0.30 Hz
GB 0
PC 1.00

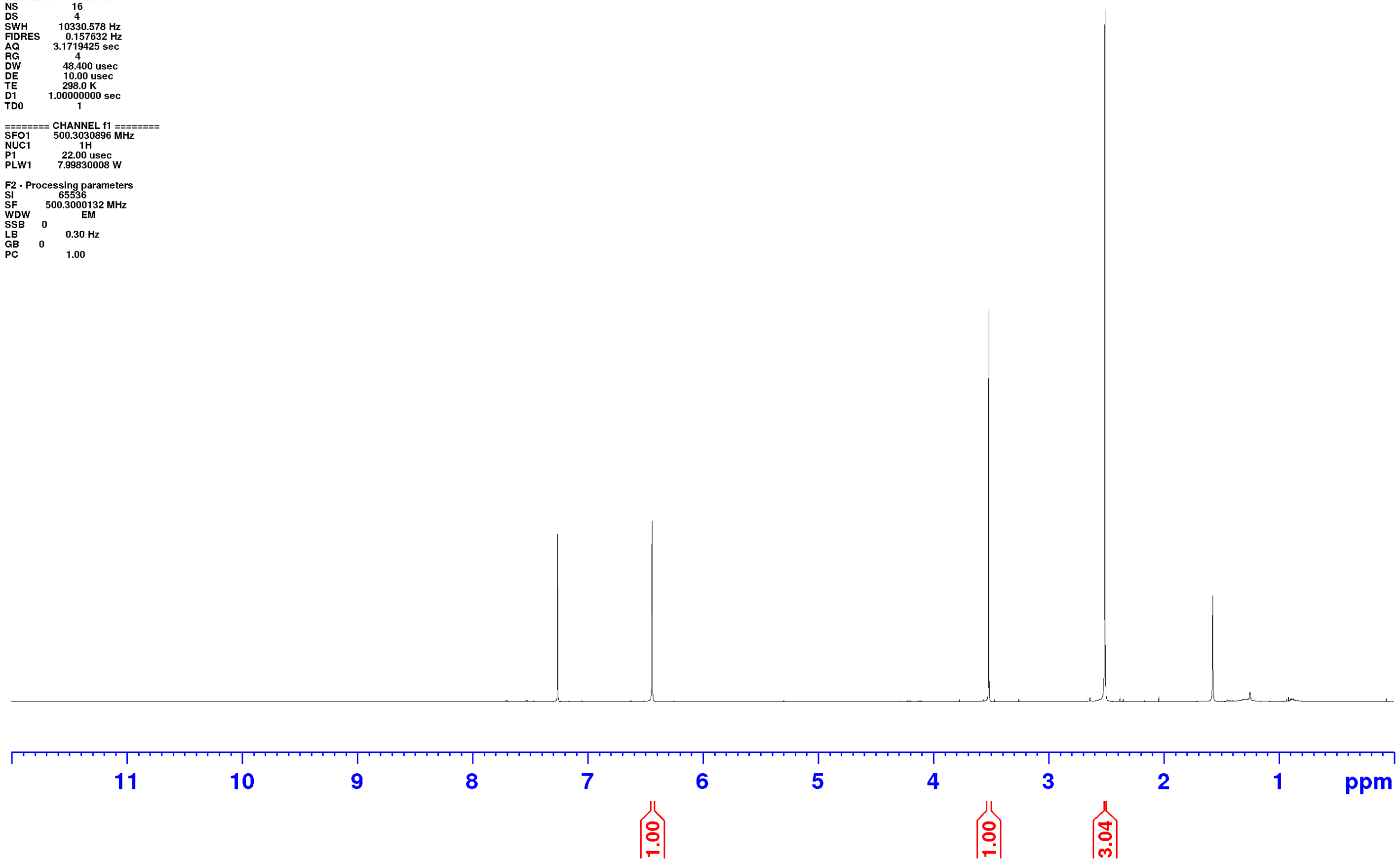

1-(5-Methylisoxazol-3-yl)prop-2-yn-1-one (9)

```
Current Data Parameters
NAME     Compound 14 - AMTB51 (at51161804)
EXPNO                  4
PROCNO                 1

F2 - Acquisition Parameters
Date_           20190421
Time               10.41
INSTRUM           avc500
PROBHD   5 mm CPDUL 13C
PULPROG           zgpg30
TD                 65536
SOLVENT            CDCl3
NS                  2048
DS                     2
SWH            31250.000 Hz
FIDRES          0.476837 Hz
AQ             1.0485760 sec
RG                   912
DW                16.000 usec
DE                 18.00 usec
TE                 298.0 K
D1            2.00000000 sec
D11           0.03000000 sec
TD0                    1

======== CHANNEL f1 ========
SFO1         125.8131152 MHz
NUC1                 13C
P1                 10.00 usec
PLW1         20.18400002 W

======== CHANNEL f2 ========
SFO2         500.3020012 MHz
NUC2                  1H
CPDPRG[2         waltz16
PCPD2              80.00 usec
PLW2          7.99830008 W
PLW12         0.60487002 W
PLW13         0.38712001 W

F2 - Processing parameters
SI                 32768
SF           125.8005199 MHz
WDW                   EM
SSB      0
LB                  1.00 Hz
GB       0
PC                  1.40
```

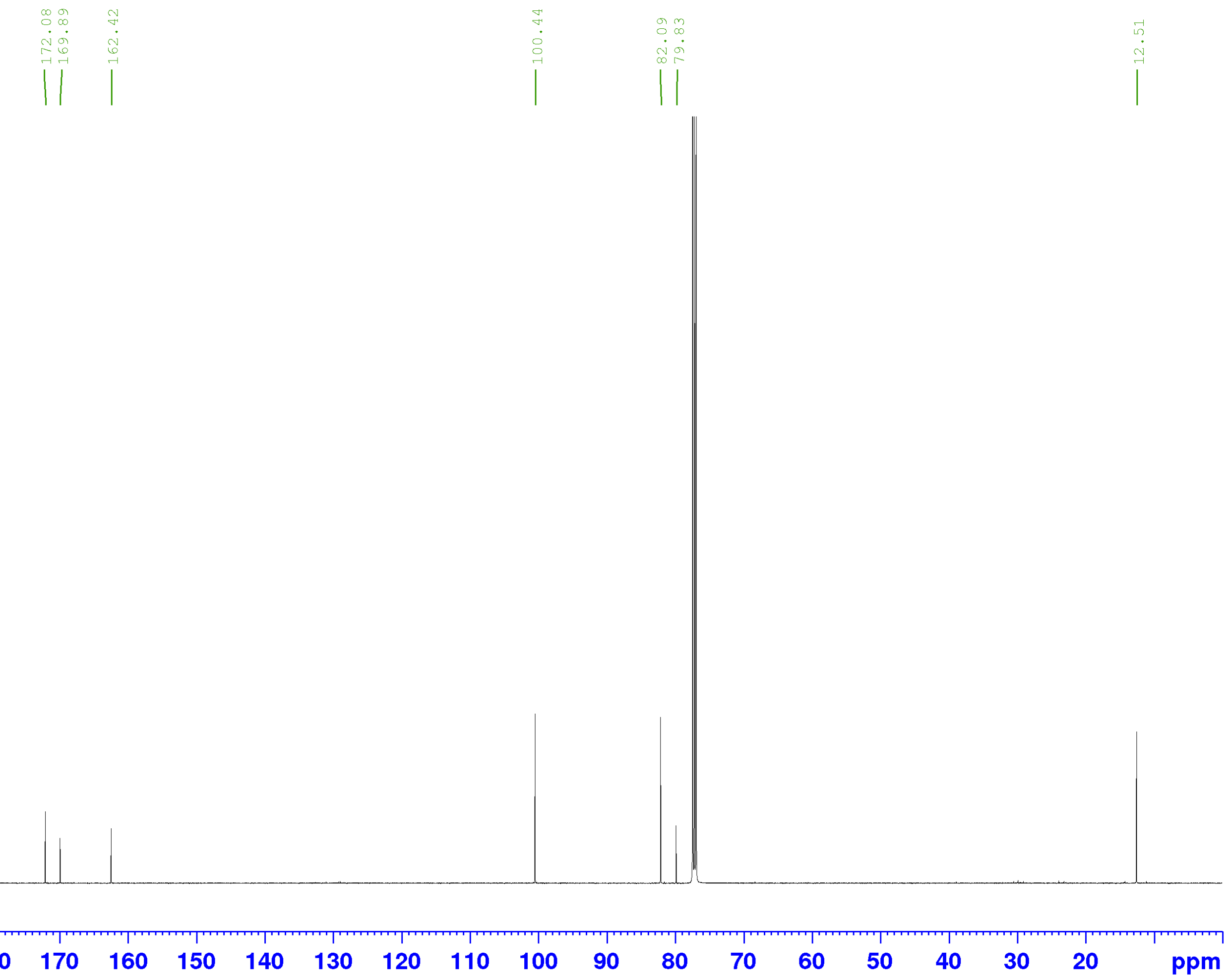

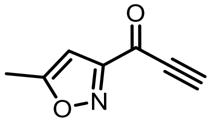

N-(3-Methylisoxazol-5-yl)prop-2-enamide (10)

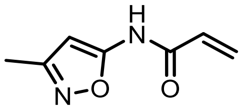

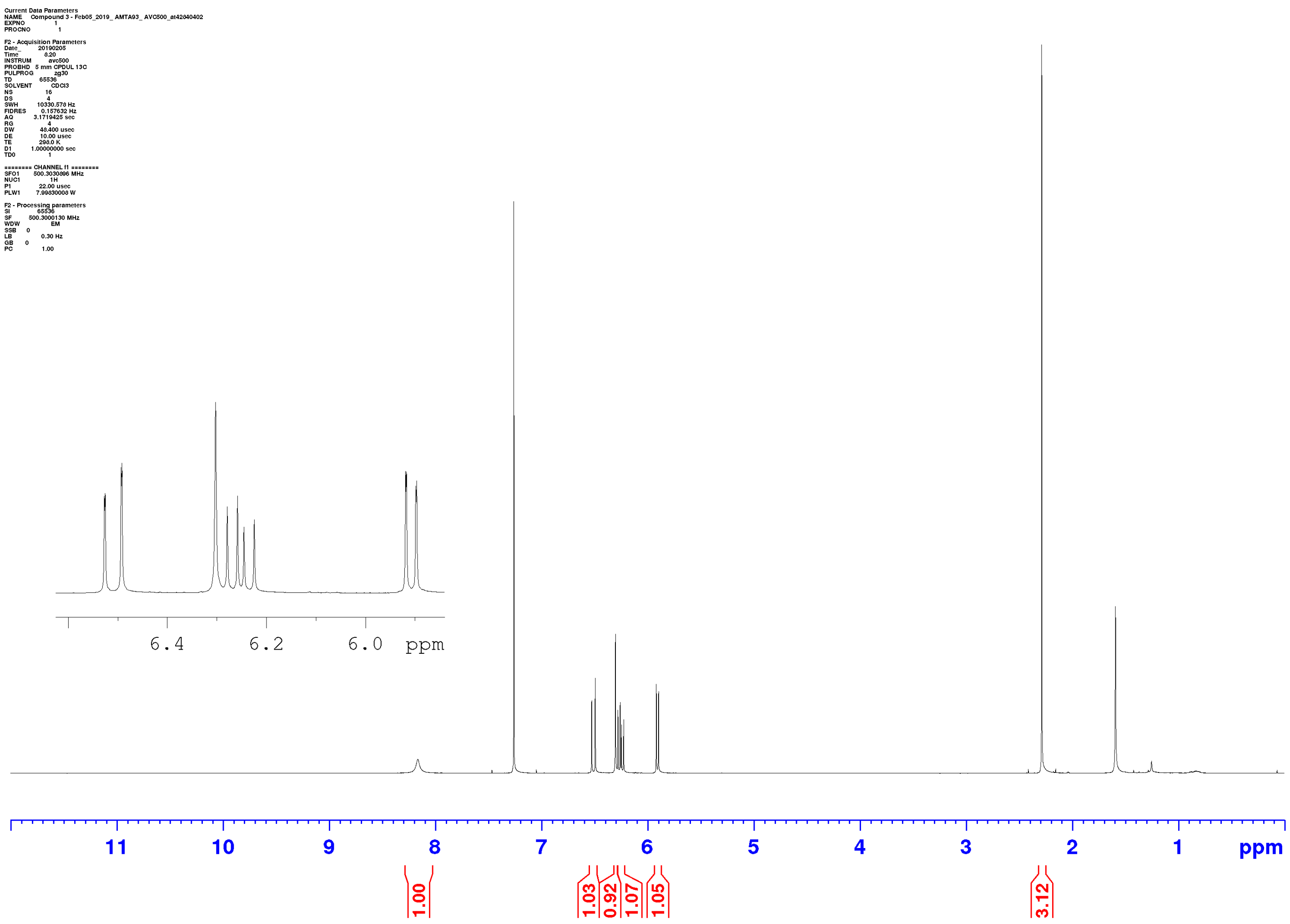

N-(3-Methylisoxazol-5-yl)prop-2-enamide (10)

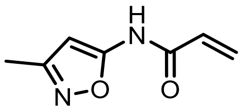

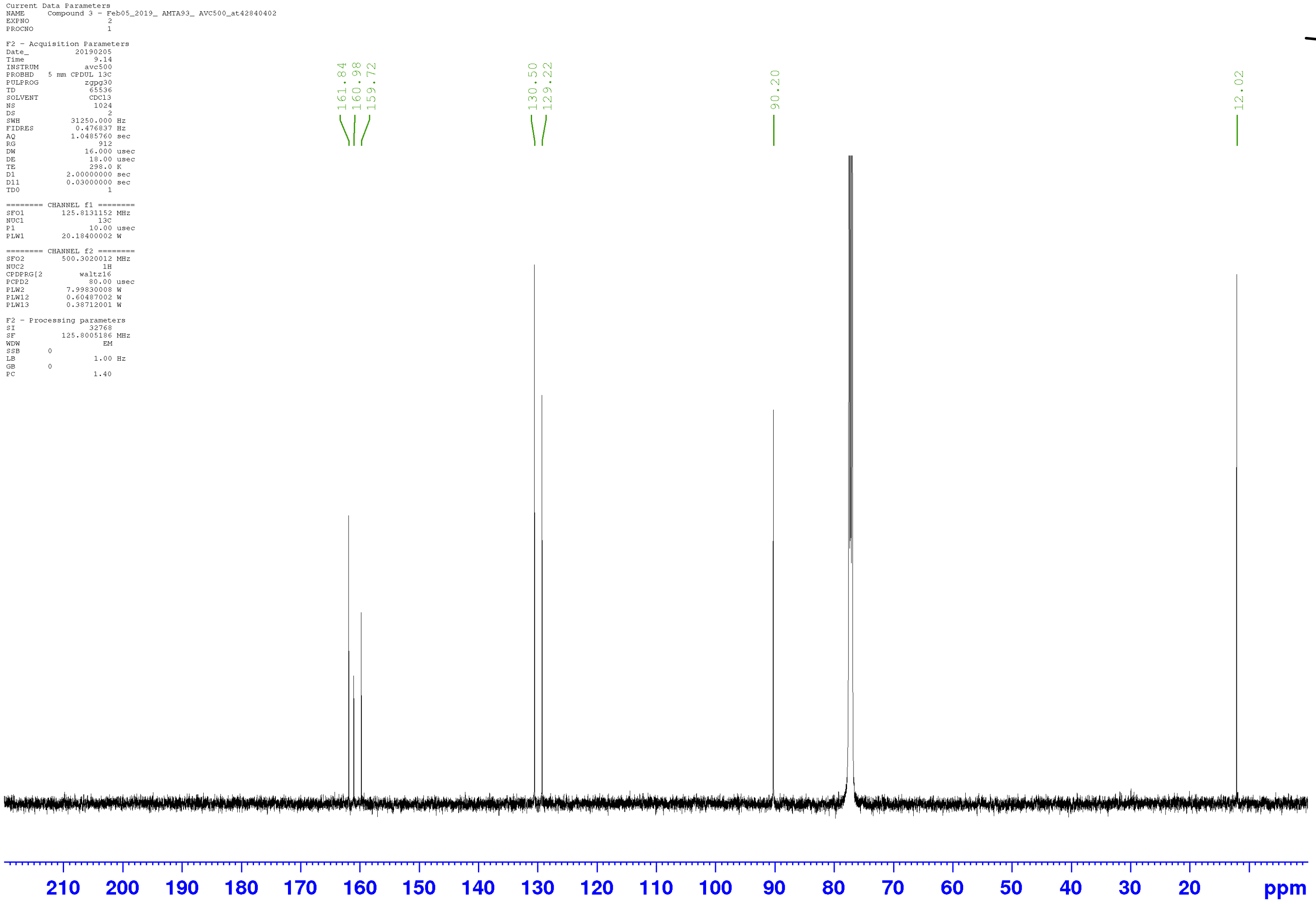

N-(5-Methylisoxazol-3-yl)prop-2-enamide (11)

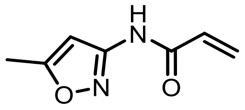

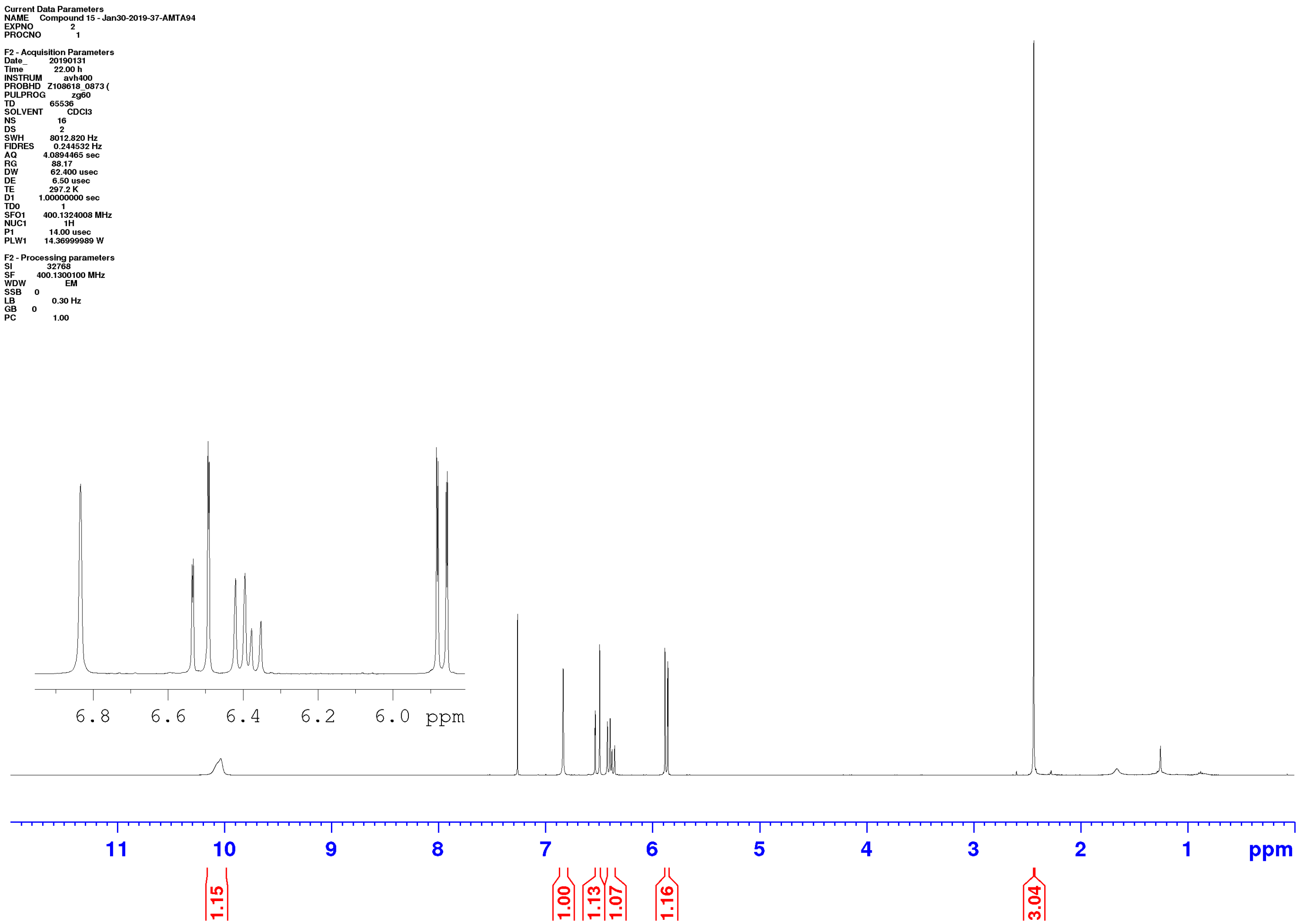

N-(5-Methylisoxazol-3-yl)prop-2-enamide (11)

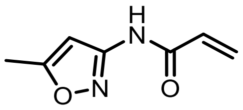

```
Current Data Parameters
NAME      Compound 15 - Jan30-2019-37-AMTA94
EXPNO                 1
PROCNO                1

F2 - Acquisition Parameters
Date_          20190131
Time              21.58 h
INSTRUM          avh400
PROBHD   Z108618_0873 (
PULPROG          zgpg30
TD                32768
SOLVENT           CDCl3
NS                  512
DS                    4
SWH           26041.666 Hz
FIDRES         1.589457 Hz
AQ            0.6291456 sec
RG               197.18
DW               19.200 usec
DE                 6.50 usec
TE                297.9 K
D1           1.00000000 sec
D11          0.03000000 sec
TD0                   1
SFO1        100.6228298 MHz
NUC1                13C
P1                10.00 usec
PLW1        47.86100006 W
SFO2        400.1316005 MHz
NUC2                 1H
CPDPRG[2        waltz16
PCPD2             90.00 usec
PLW2        14.36999989 W
PLW12        0.34660661 W
PLW13        0.17371930 W

F2 - Processing parameters
SI                32768
SF          100.6127553 MHz
WDW                  EM
SSB      0
LB                 1.00 Hz
GB       0
PC                 1.40
```

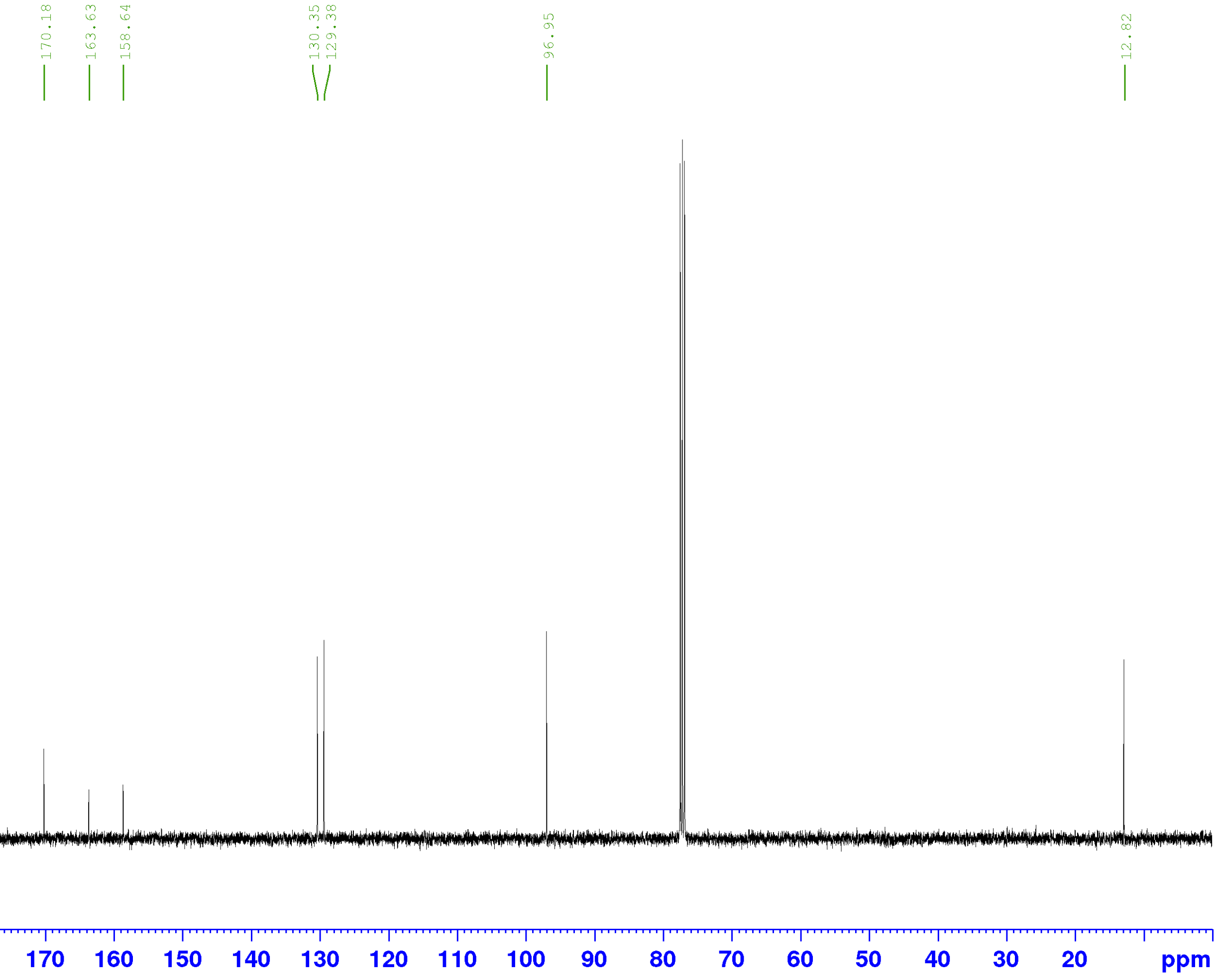

N-(3-Methyl-4-phenylisoxazol-5-yl)prop-2-enamide (12)

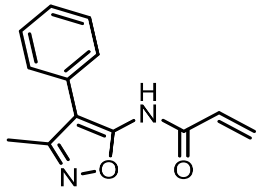

Current Data Parameters
NAME at602461112
EXPNO 1
PROCNO 1

F2 - Acquisition Parameters
Date_ 20201213
Time 19.49
INSTRUM avc500
PROBHD 5 mm CPDUL 13C
PULPROG zg30
TD 65536
SOLVENT DMSO
NS 16
DS 4
SWH 10330.578 Hz
FIDRES 0.157632 Hz
AQ 3.1719425 sec
RG 4
DW 48.400 usec
DE 10.00 usec
TE 298.0 K
D1 1.00000000 sec
TD0 1

======== CHANNEL f1 ========
SFO1 500.3030896 MHz
NUC1 1H
P1 22.00 usec
PLW1 7.99830008 W

F2 - Processing parameters
SI 65536
SF 500.3000053 MHz
WDW EM
SSB 0
LB 0.30 Hz
GB 0
PC 1.00

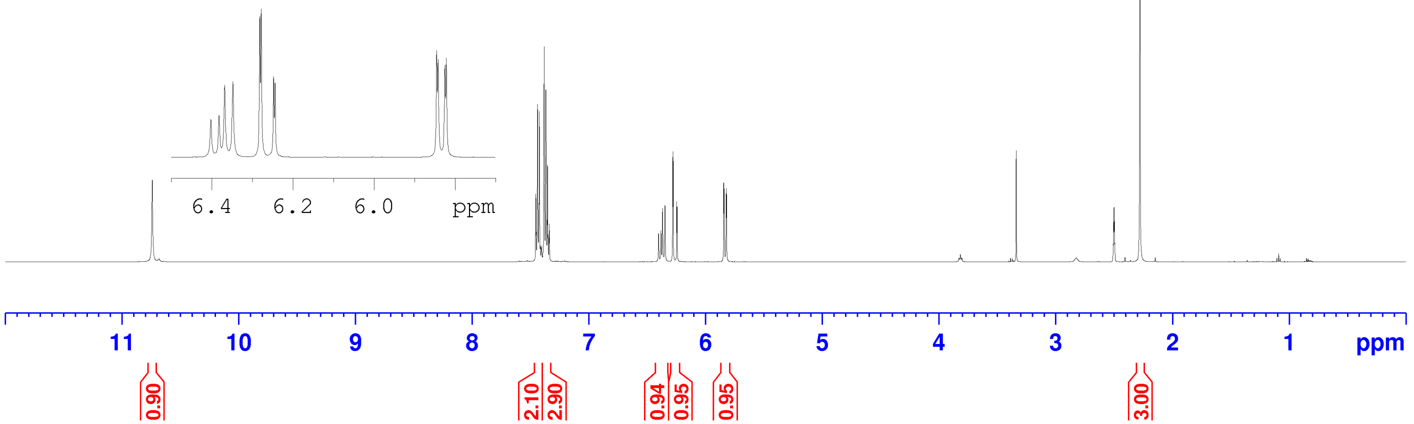

N-(3-Methyl-4-phenylisoxazol-5-yl)prop-2-enamide (12)

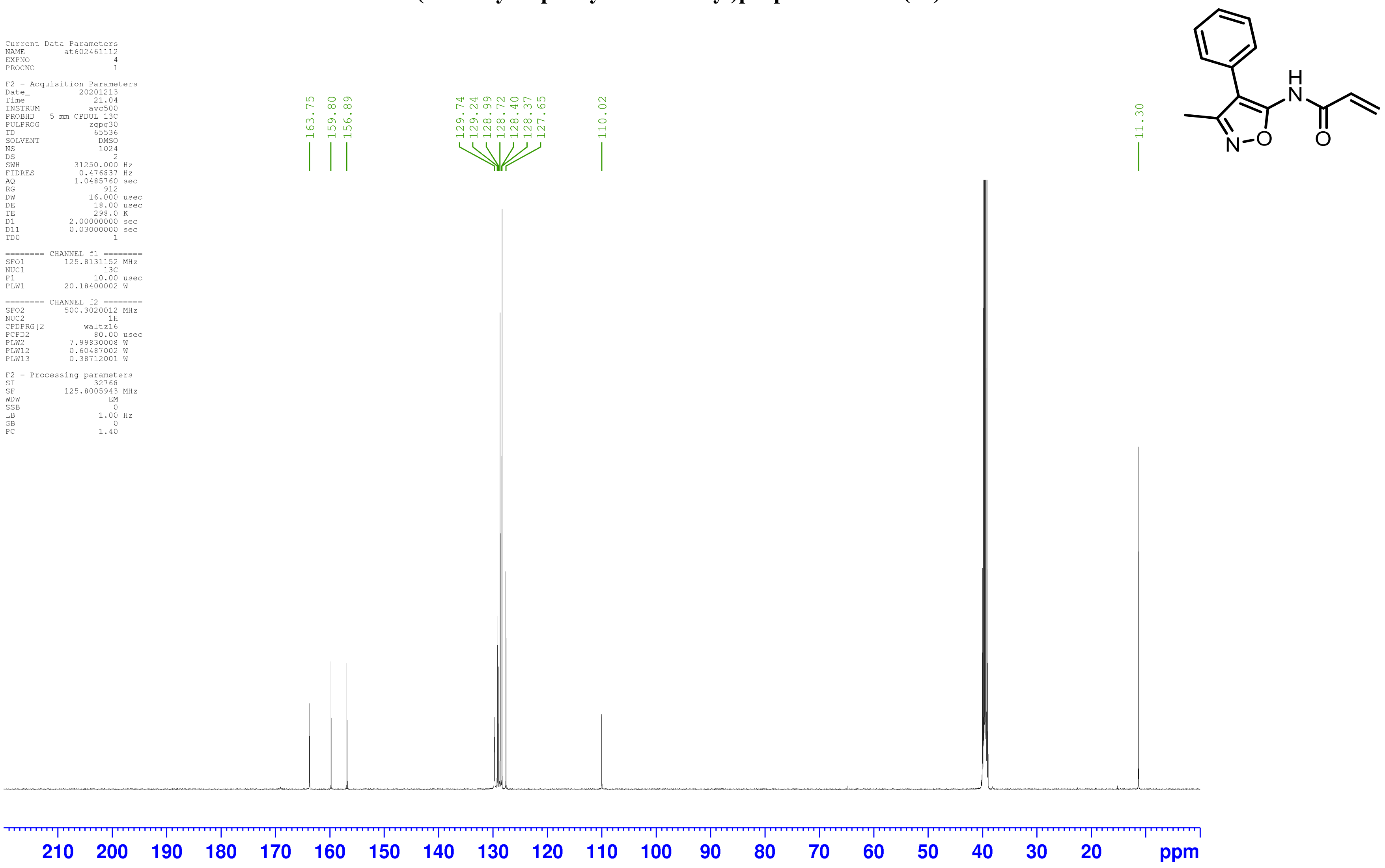

(*E*)-4-(Dimethylamino)-*N*-(3-methylisoxazol-5-yl)but-2-enamide (13)

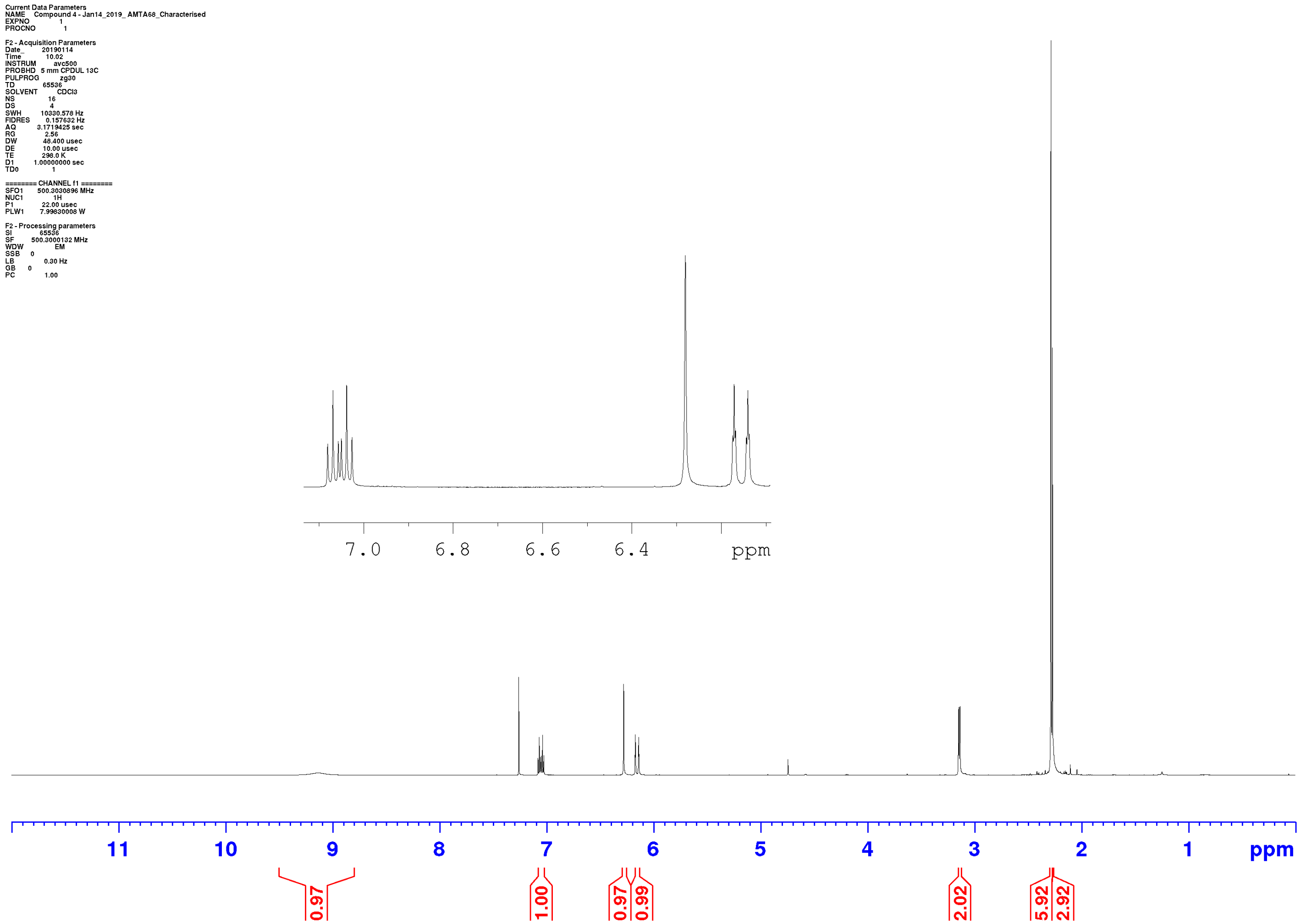

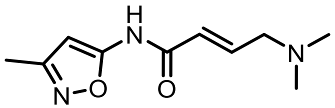

(*E*)-4-(Dimethylamino)-*N*-(3-methylisoxazol-5-yl)but-2-enamide (13)

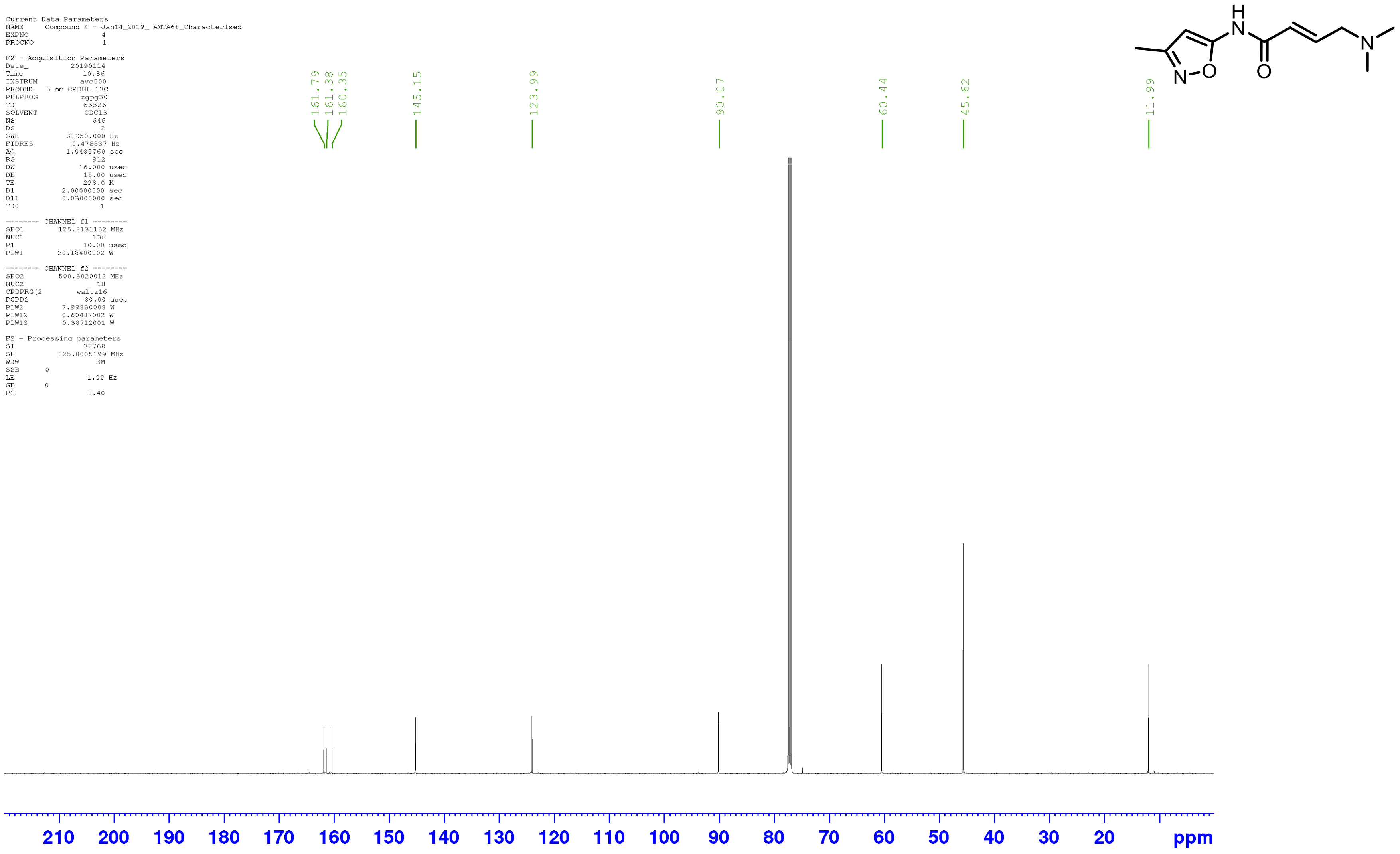

(*E*)-4-Bromo/Chloro-*N*-(3-methyl-4-phenylisoxazol-5-yl)but-2-enamide (65)

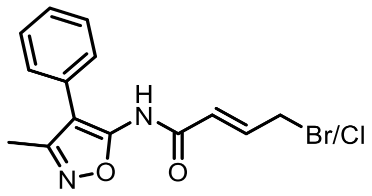

Current Data Parameters
NAME at76740702
EXPNO 1
PROCNO 1

F2 - Acquisition Parameters
Date_ 20200209
Time 12.44
INSTRUM avc500
PROBHD 5 mm CPDUL 13C
PULPROG zg30
TD 65536
SOLVENT CDCl3
NS 16
DS 4
SWH 10330.578 Hz
FIDRES 0.157632 Hz
AQ 3.1719425 sec
RG 4
DW 48.400 usec
DE 10.00 usec
TE 298.0 K
D1 1.00000000 sec
TD0 1

======== CHANNEL f1 ========
SFO1 500.3030896 MHz
NUC1 1H
P1 22.00 usec
PLW1 7.99830008 W

F2 - Processing parameters
SI 65536
SF 500.3000132 MHz
WDW EM
SSB 0
LB 0.30 Hz
GB 0
PC 1.00

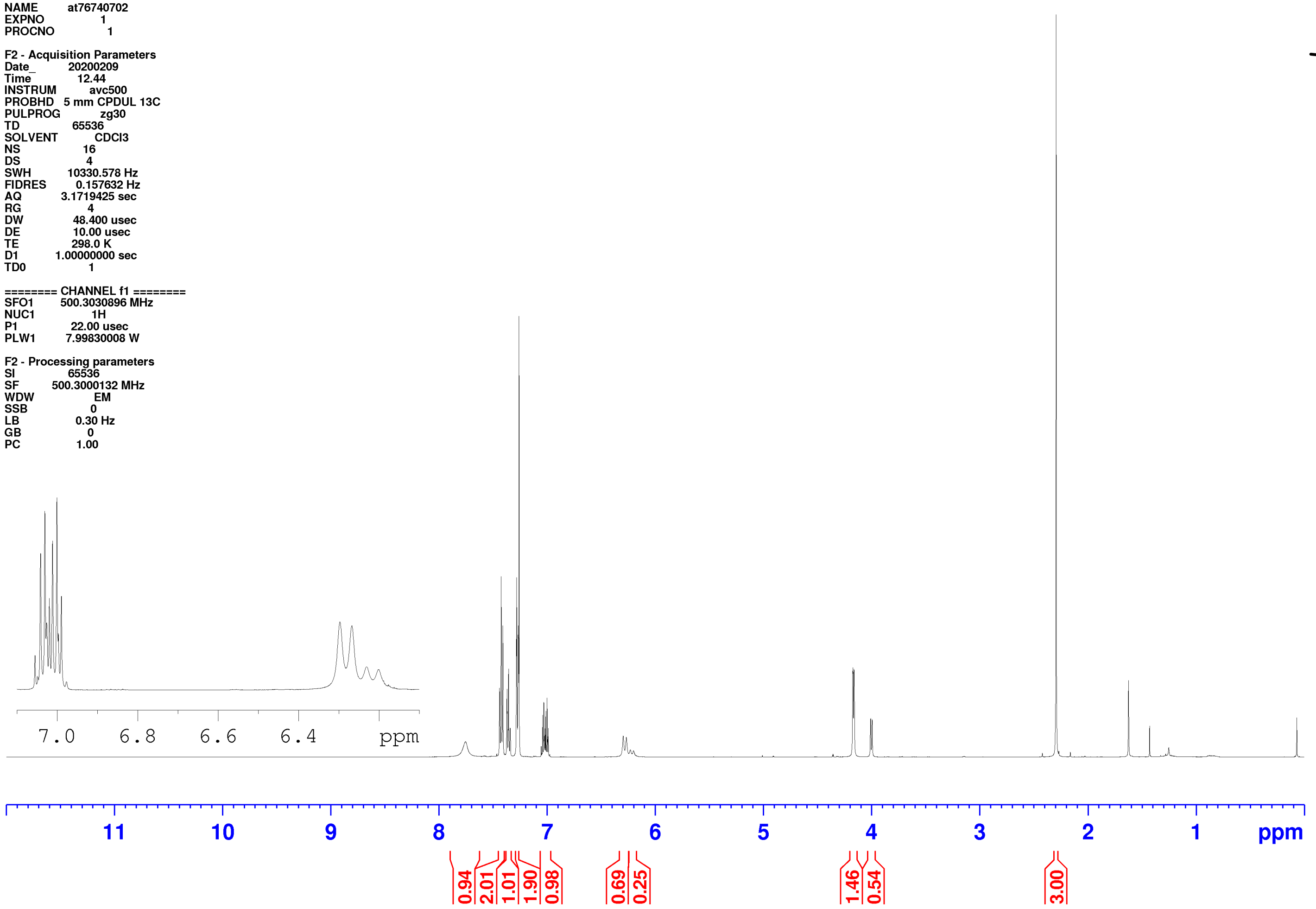

(*E*)-4-(Dimethylamino)-*N*-(3-methyl-4-phenylisoxazol-5-yl)but-2-enamide (14)

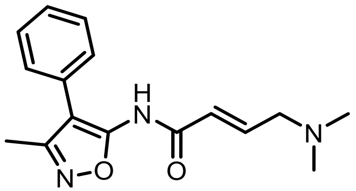

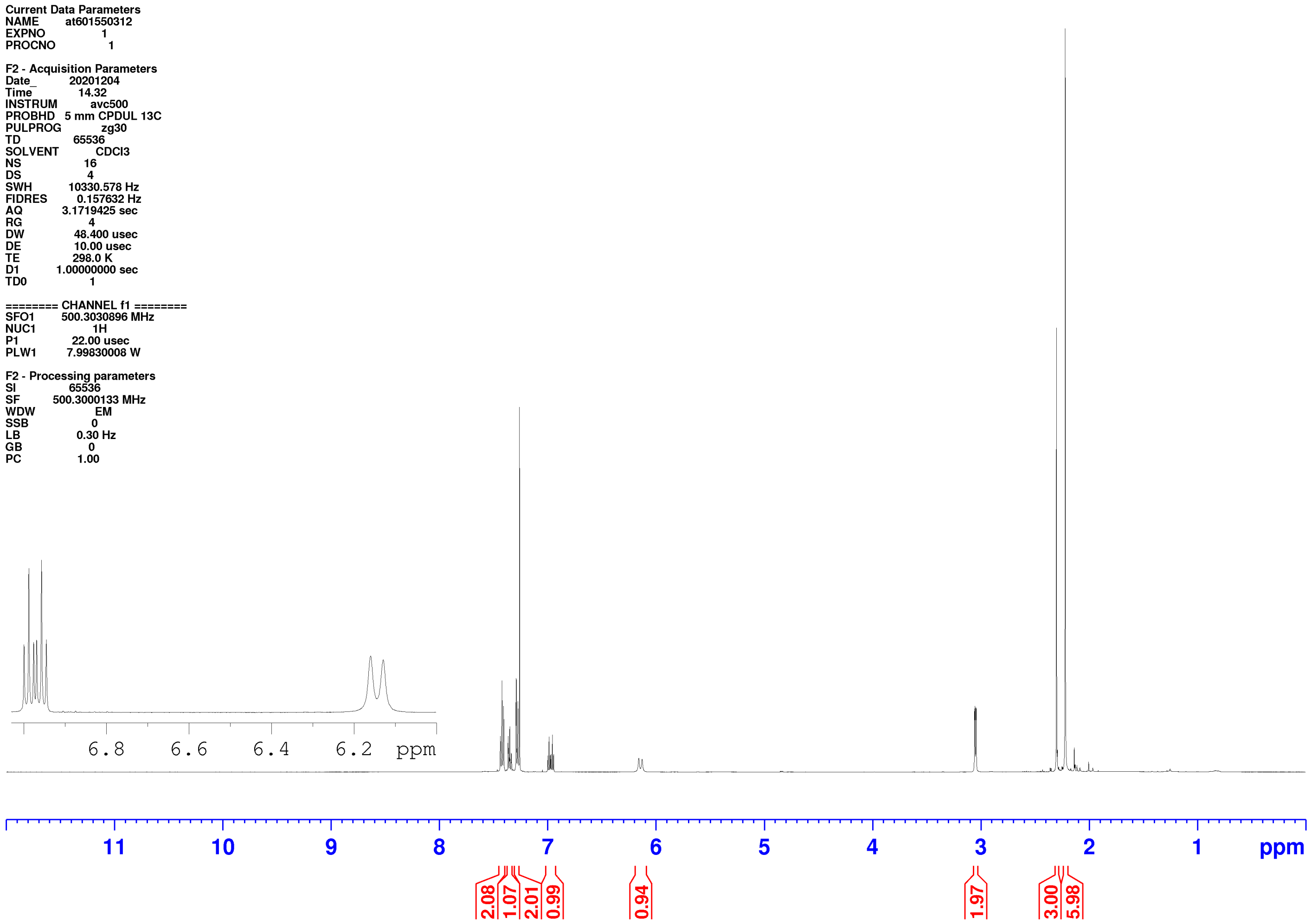

(*E*)-4-(Dimethylamino)-*N*-(3-methyl-4-phenylisoxazol-5-yl)but-2-enamide (14)

```
Current Data Parameters
NAME          at601550312
EXPNO                   4
PROCNO                  1

F2 - Acquisition Parameters
Date_            20201204
Time                15.47
INSTRUM            avc500
PROBHD   5 mm CPDUL 13C
PULPROG           zgpg30
TD                  65536
SOLVENT             CDCl3
NS                   1024
DS                      2
SWH           31250.000 Hz
FIDRES         0.476837 Hz
AQ            1.0485760 sec
RG                    912
DW               16.000 usec
DE                18.00 usec
TE                298.0 K
D1           2.00000000 sec
D11          0.03000000 sec
TD0                     1

======== CHANNEL f1 ========
SFO1        125.8131152 MHz
NUC1                  13C
P1                  10.00 usec
PLW1        20.18400002 W

======== CHANNEL f2 ========
SFO2        500.3020012 MHz
NUC2                   1H
CPDPRG[2          waltz16
PCPD2               80.00 usec
PLW2         7.99830008 W
PLW12        0.60487002 W
PLW13        0.38712001 W

F2 - Processing parameters
SI                  32768
SF          125.8005197 MHz
WDW                    EM
SSB                     0
LB                   1.00 Hz
GB                      0
PC                   1.40
```

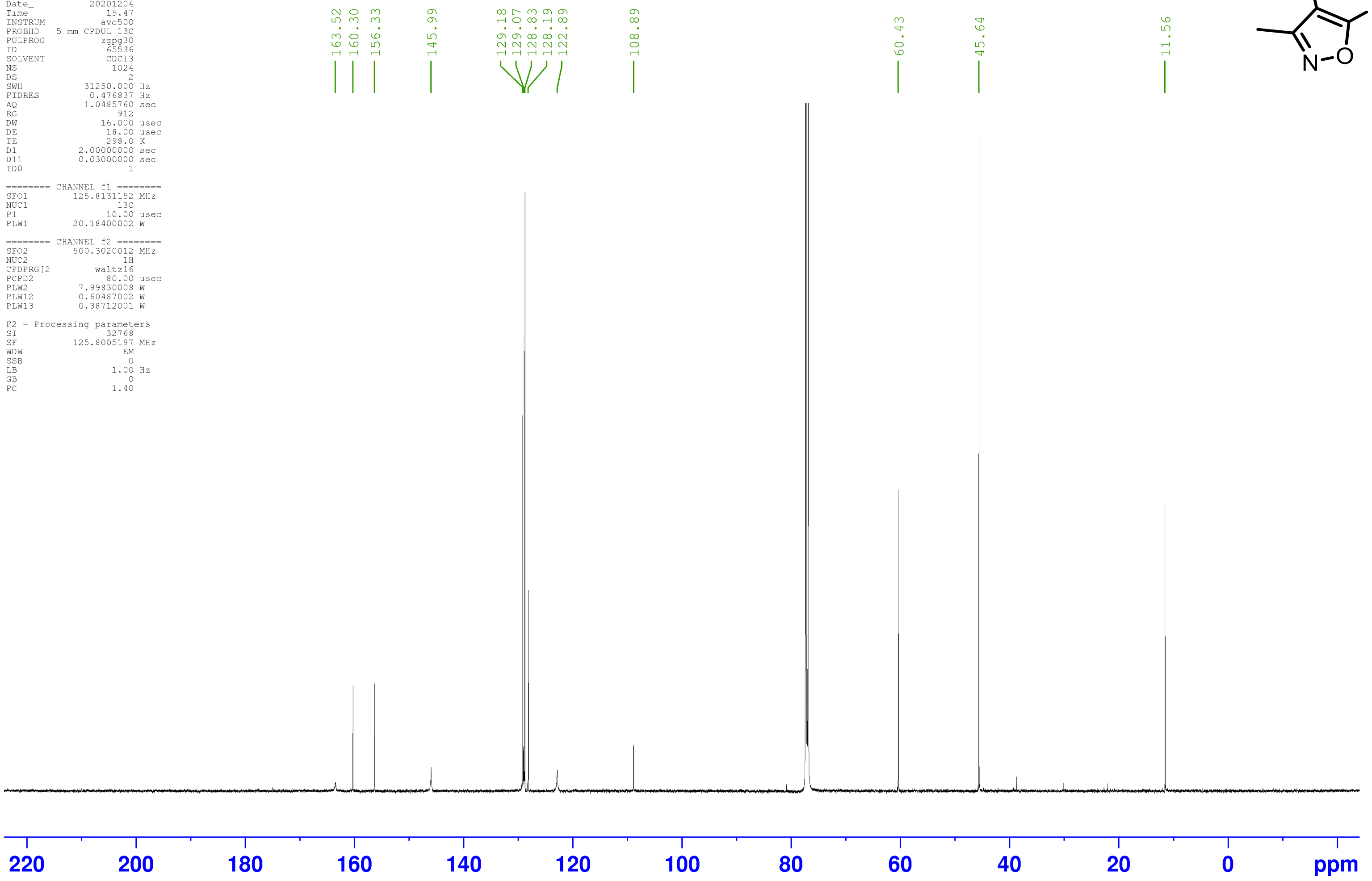

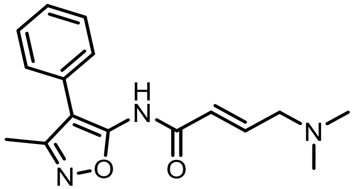

(*E*)-*N*-(3-Methyl-4-phenylisoxazol-5-yl)but-2-enamide (15)

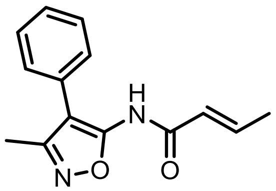

Current Data Parameters
NAME at615020104
EXPNO 1
PROCNO 1

F2 - Acquisition Parameters
Date_ 20210403
Time 8.22 h
INSTRUM avx500
PROBHD Z119877_0007 (
PULPROG zg60
TD 65536
SOLVENT CDCl3
NS 16
DS 2
SWH 10000.000 Hz
FIDRES 0.305176 Hz
AQ 3.2767999 sec
RG 191.37
DW 50.000 usec
DE 6.50 usec
TE 298.5 K
D1 1.00000000 sec
TD0 1
SFO1 500.3025015 MHz
NUC1 1H
P1 15.00 usec
PLW1 31.53700066 W

F2 - Processing parameters
SI 65536
SF 500.3000122 MHz
WDW EM
SSB 0
LB 0.30 Hz
GB 0
PC 1.00

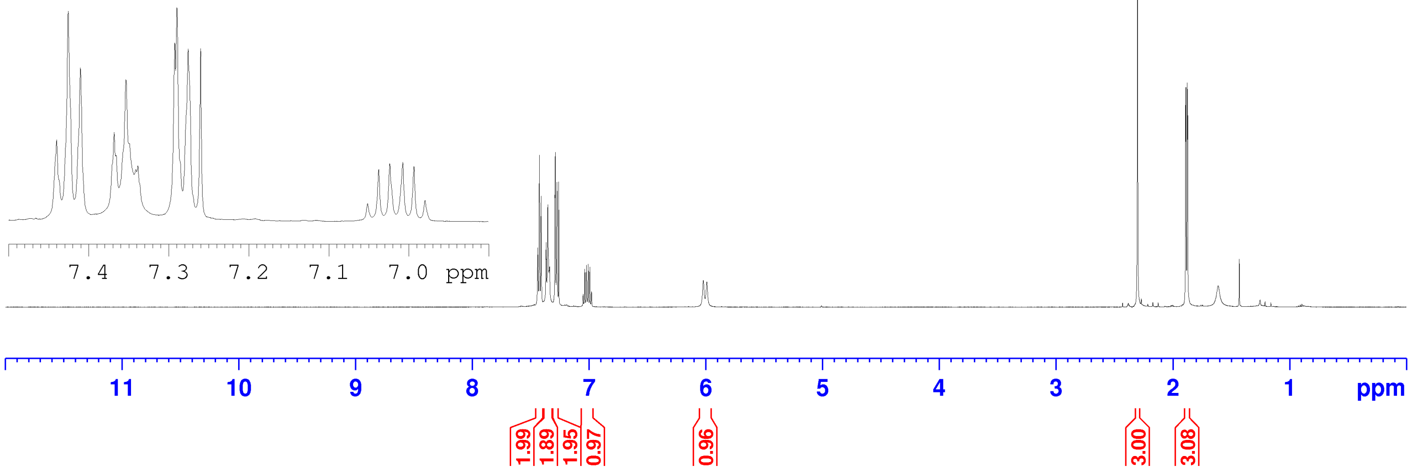

(*E*)-*N*-(3-Methyl-4-phenylisoxazol-5-yl)but-2-enamide (15)

```
Current Data Parameters
NAME          at615020104
EXPNO                   5
PROCNO                  1

F2 - Acquisition Parameters
Date_            20210403
Time                12.31 h
INSTRUM            avx500
PROBHD    Z119877_0007 (
PULPROG           zgpg30
TD                  65536
SOLVENT             CDCl3
NS                   2048
DS                      4
SWH             29761.904 Hz
FIDRES           0.908261 Hz
AQ              1.1010048 sec
RG                 191.37
DW                 16.800 usec
DE                   6.50 usec
TE                  299.8 K
D1             2.00000000 sec
D11            0.03000000 sec
TD0                     1
SFO1          125.8131151 MHz
NUC1                  13C
P0                   3.33 usec
P1                  10.00 usec
PLW1          46.89899826 W
SFO2          500.3020012 MHz
NUC2                   1H
CPDPRG[2          waltz16
PCPD2               80.00 usec
PLW2          31.53700066 W
PLW12          1.10870004 W
PLW13          0.55768001 W

F2 - Processing parameters
SI                  32768
SF            125.8005183 MHz
WDW                    EM
SSB                     0
LB                   1.00 Hz
GB                      0
PC                   1.40
```

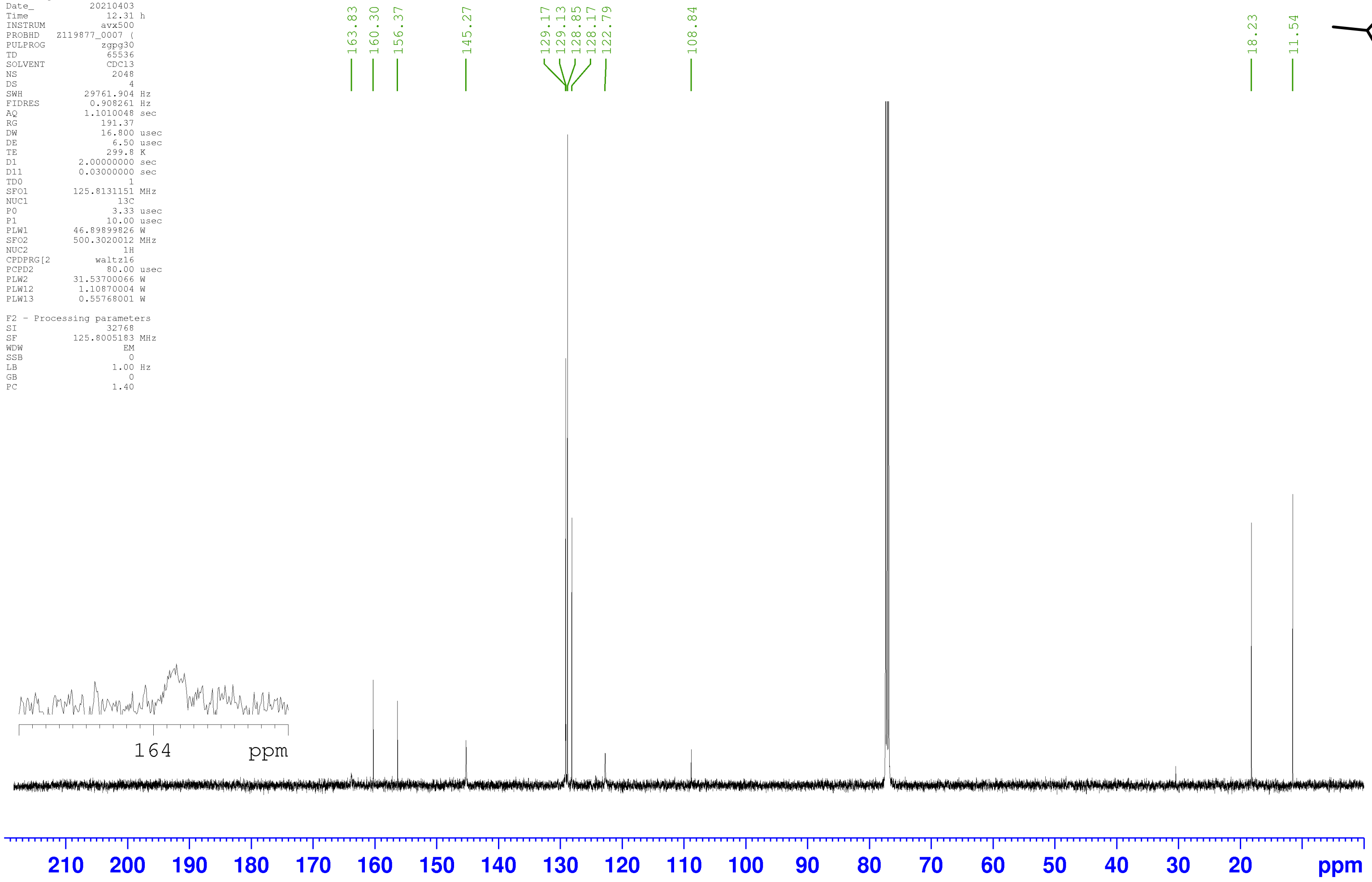

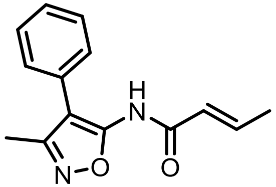

N-[(3-Methylisoxazol-5-yl)methyl]prop-2-enamide (16)

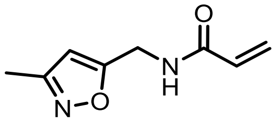

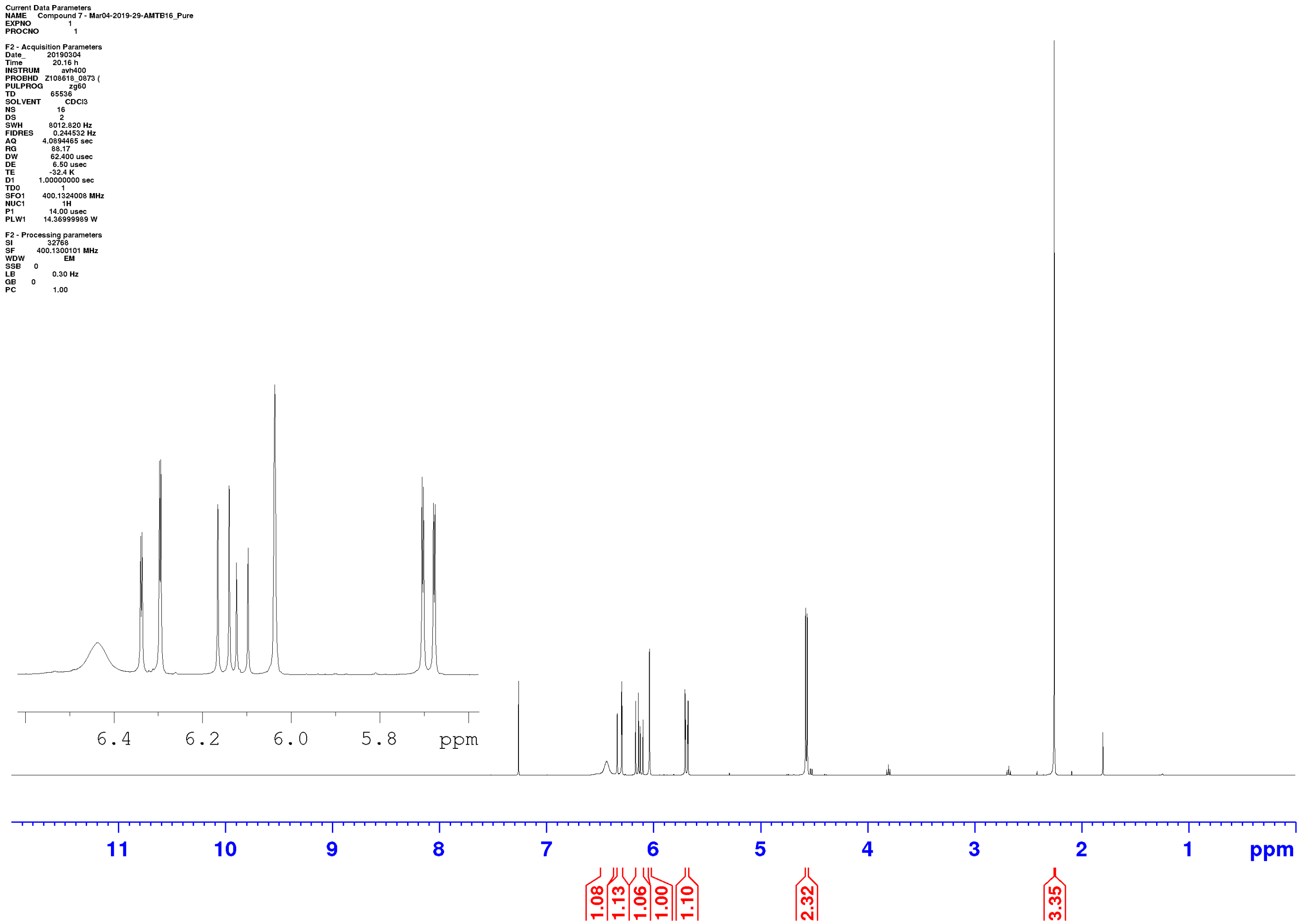

N-[(3-Methylisoxazol-5-yl)methyl]prop-2-enamide (16)

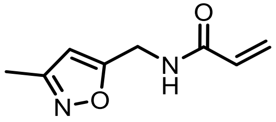


```
Current Data Parameters
NAME     Compound 7 - Mar04-2019-29-AMTB16_Pure
EXPNO                  3
PROCNO                 1

F2 - Acquisition Parameters
Date_          20190304
Time              20.45 h
INSTRUM          avh400
PROBHD   Z108618_0873 (
PULPROG          zgpg30
TD                32768
SOLVENT           CDCl3
NS                  512
DS                    4
SWH           26041.666 Hz
FIDRES         1.589457 Hz
AQ            0.6291456 sec
RG               197.18
DW               19.200 usec
DE                 6.50 usec
TE                -32.4 K
D1           1.00000000 sec
D11          0.03000000 sec
TD0                   1
SFO1        100.6228298 MHz
NUC1                13C
P1                10.00 usec
PLW1        47.86100006 W
SFO2        400.1316005 MHz
NUC2                 1H
CPDPRG[2        waltz16
PCPD2             90.00 usec
PLW2        14.36999989 W
PLW12        0.34660661 W
PLW13        0.17371930 W

F2 - Processing parameters
SI                32768
SF          100.6127586 MHz
WDW                  EM
SSB      0
LB                 1.00 Hz
GB       0
PC                 1.40
```

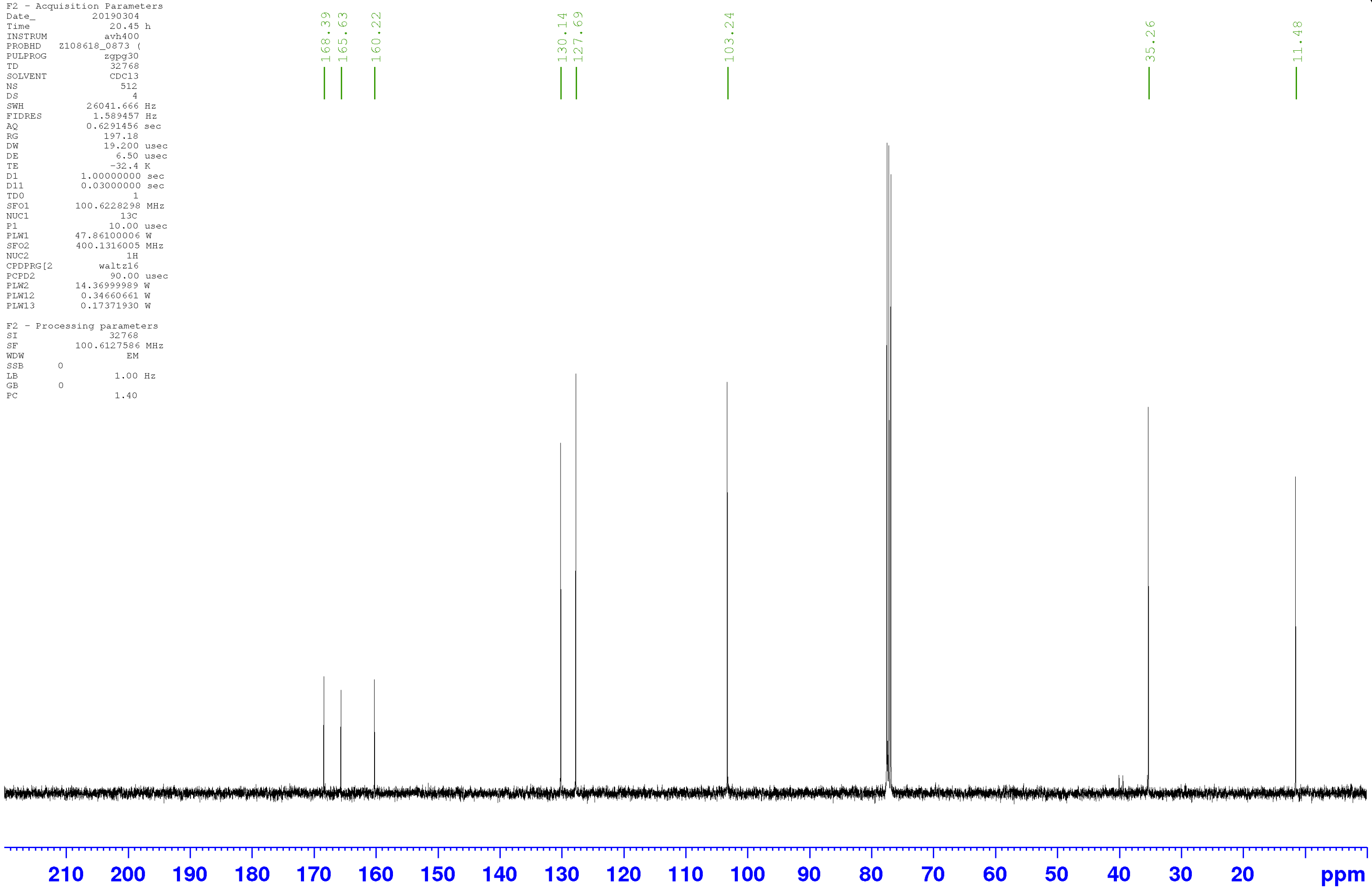

N-[(5-Methylisoxazol-3-yl)methyl]prop-2-enamide (17)

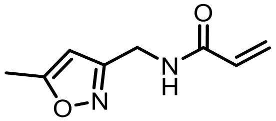

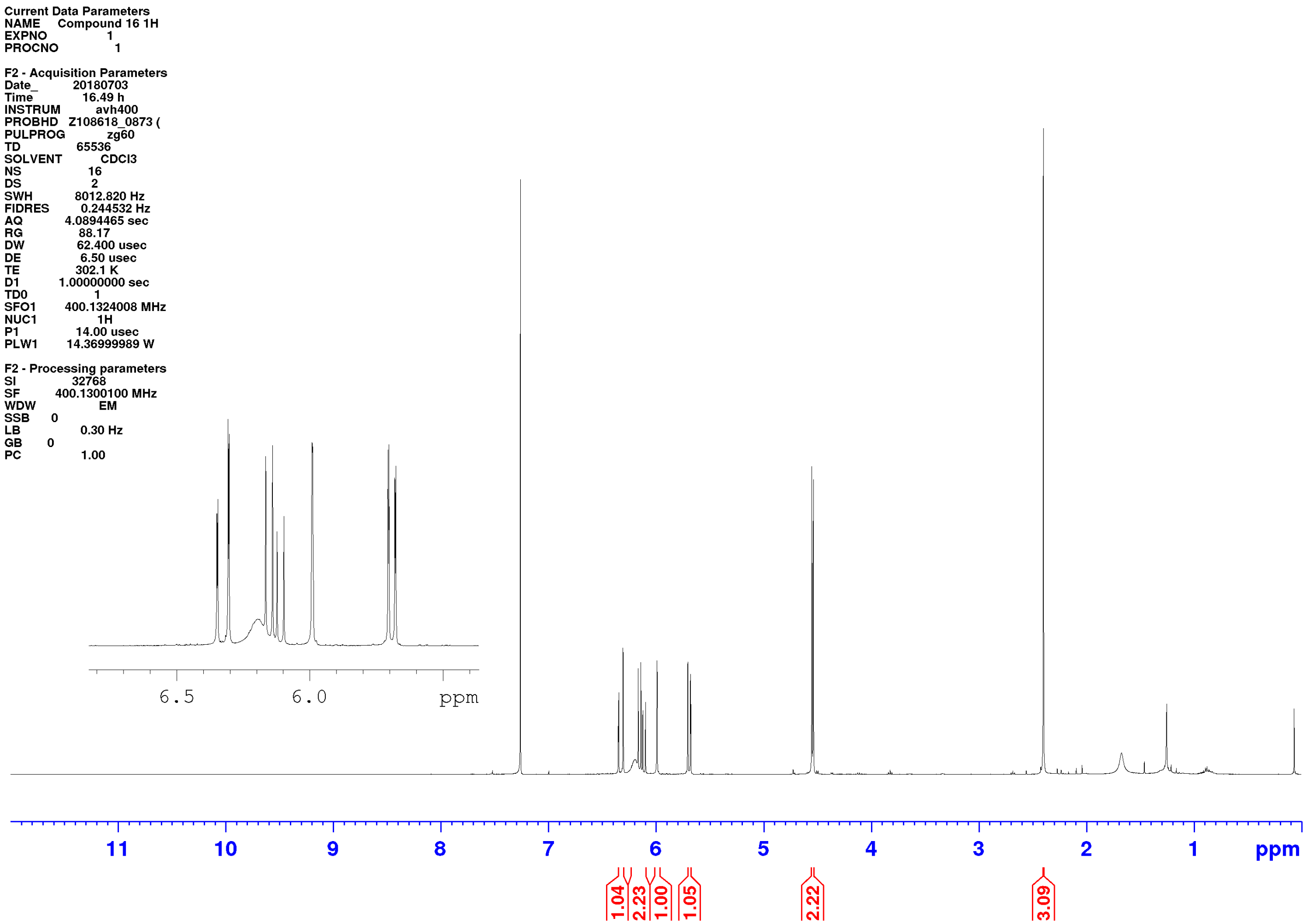

N-[(5-Methylisoxazol-3-yl)methyl]prop-2-enamide (17)

```
Current Data Parameters
NAME      Compound 16 13C
EXPNO                   2
PROCNO                  1

F2 - Acquisition Parameters
Date_            20180511
Time                20.29 h
INSTRUM            avh400
PROBHD    Z108618_0873 (
PULPROG           zgpg30
TD                 32768
SOLVENT            CDCl3
NS                   512
DS                     4
SWH            26041.666 Hz
FIDRES          1.589457 Hz
AQ             0.6291456 sec
RG                197.18
DW                19.200 usec
DE                  6.50 usec
TE                 296.9 K
D1            1.00000000 sec
D11           0.03000000 sec
TD0                    1
SFO1         100.6228298 MHz
NUC1                 13C
P1                 10.00 usec
PLW1         47.86100006 W
SFO2         400.1316005 MHz
NUC2                  1H
CPDPRG[2        waltz16
PCPD2              90.00 usec
PLW2         14.36999989 W
PLW12         0.34660661 W
PLW13         0.17371930 W

F2 - Processing parameters
SI                 32768
SF           100.6127578 MHz
WDW                   EM
SSB       0
LB                  1.00 Hz
GB        0
PC                  1.40
```

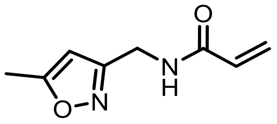

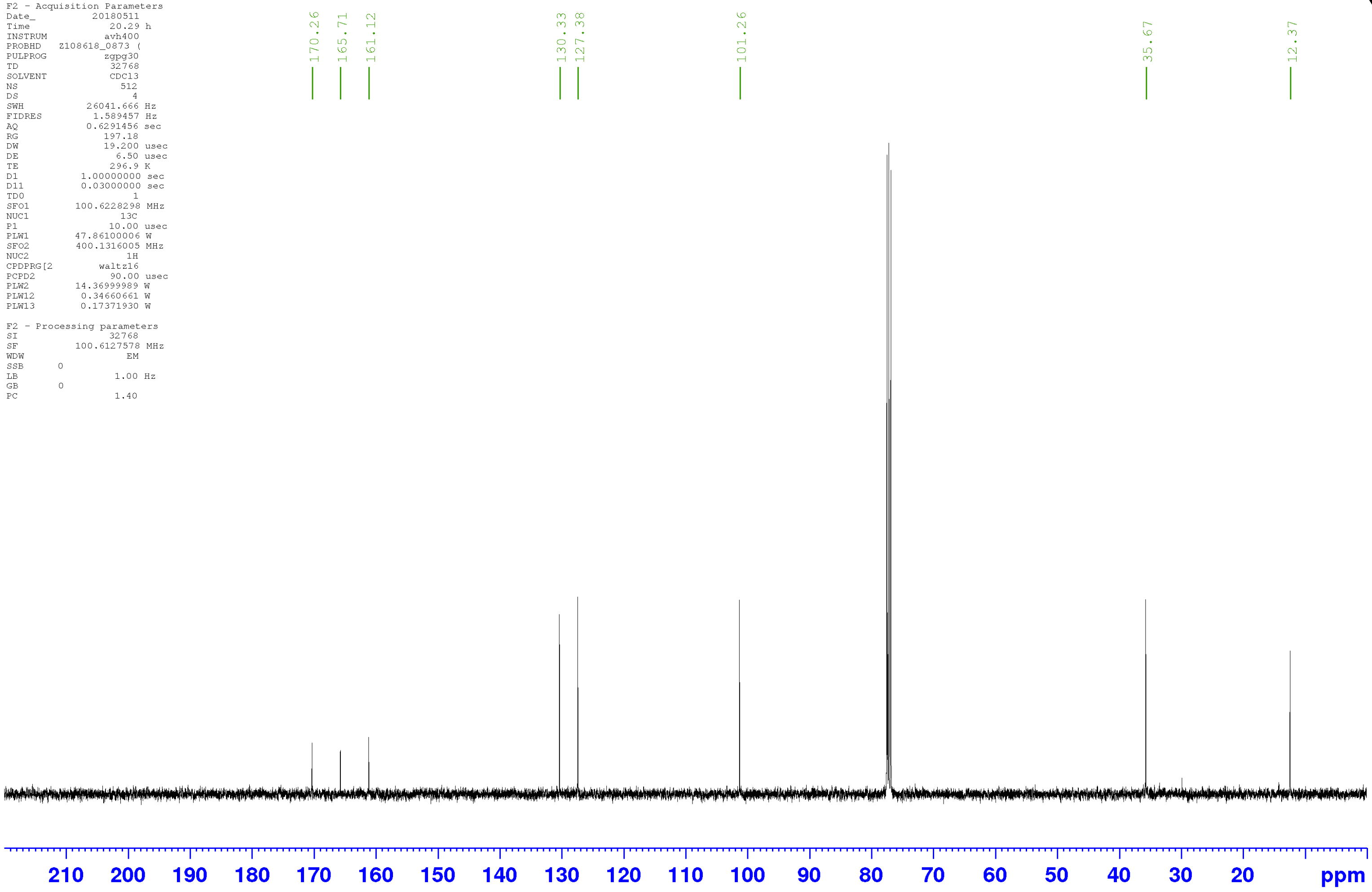

(*E*)-4-(Dimethylamino)-*N*-(3-methylisoxazol-5-yl)but-2-enamide (18)

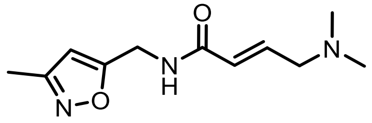

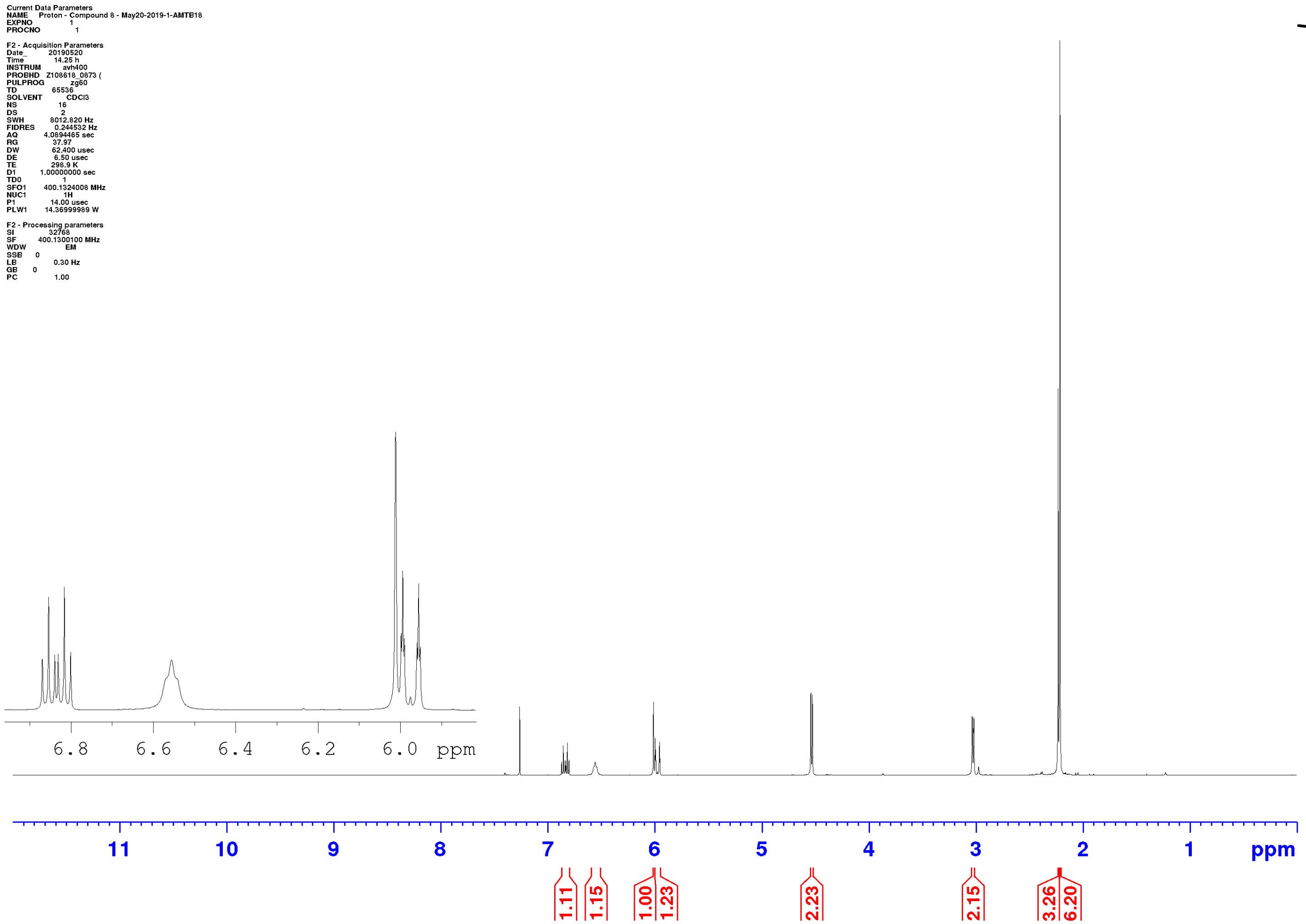

(*E*)-4-(Dimethylamino)-*N*-(3-methylisoxazol-5-yl)but-2-enamide (18)

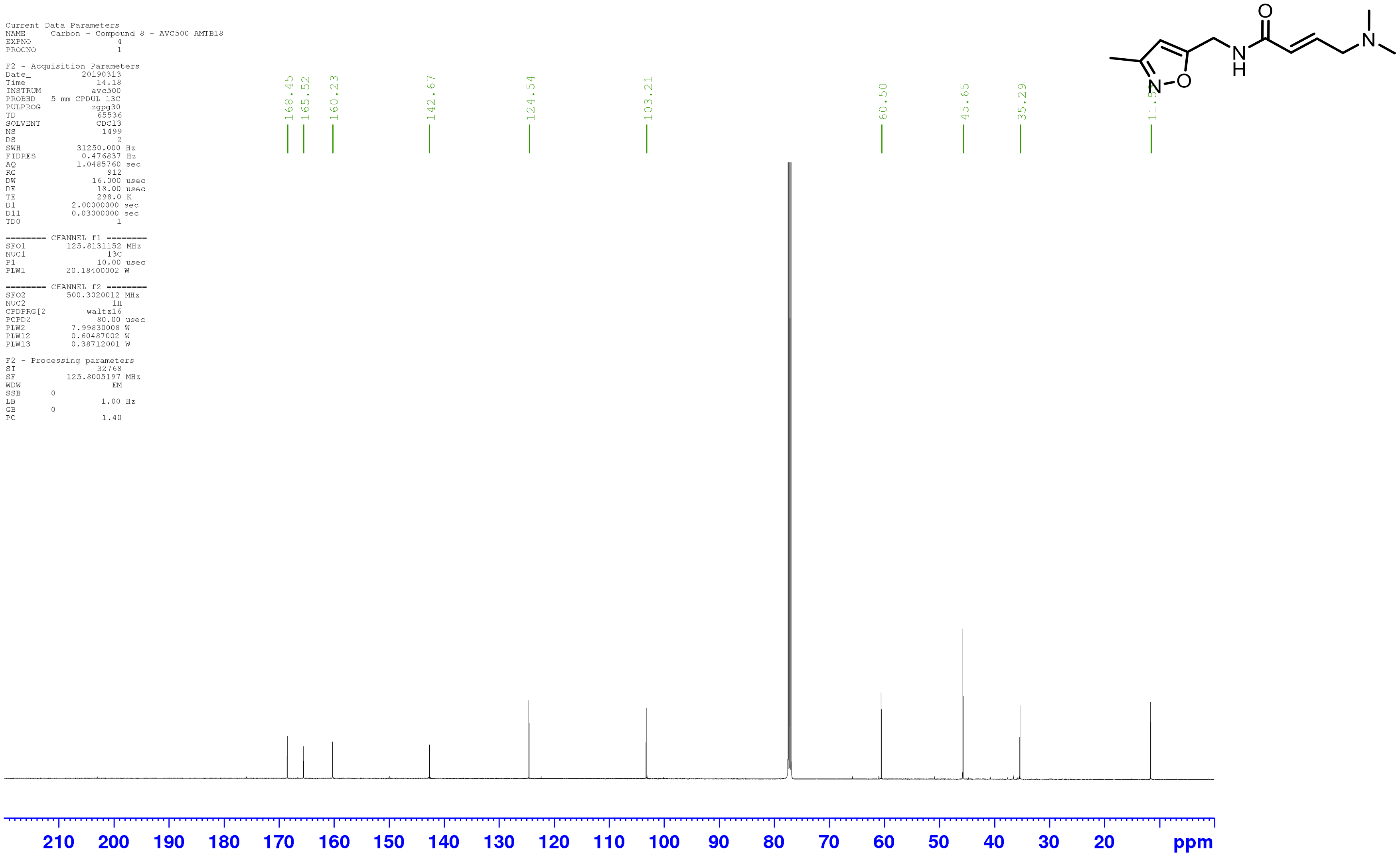

N-(3-Methylisoxazol-5-yl)prop-2-ynamide (19)

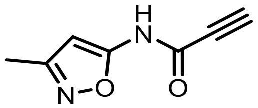

Current Data Parameters
NAME Compound 5 - Aug29-2019-58-AMTA88_Recryst
EXPNO 1
PROCNO 1

F2 - Acquisition Parameters
Date_ 20190829
Time 19.02 h
INSTRUM avh400
PROBHD Z108618_0873 (
PULPROG zg60
TD 65536
SOLVENT CDCl3
NS 16
DS 2
SWH 8012.820 Hz
FIDRES 0.244532 Hz
AQ 4.0894465 sec
RG 88.17
DW 62.400 usec
DE 6.50 usec
TE 309.8 K
D1 1.00000000 sec
TD0 1
SFO1 400.1324008 MHz
NUC1 1H
P1 14.00 usec
PLW1 14.36999989 W

F2 - Processing parameters
SI 32768
SF 400.1300100 MHz
WDW EM
SSB 0
LB 0.30 Hz
GB 0
PC 1.00

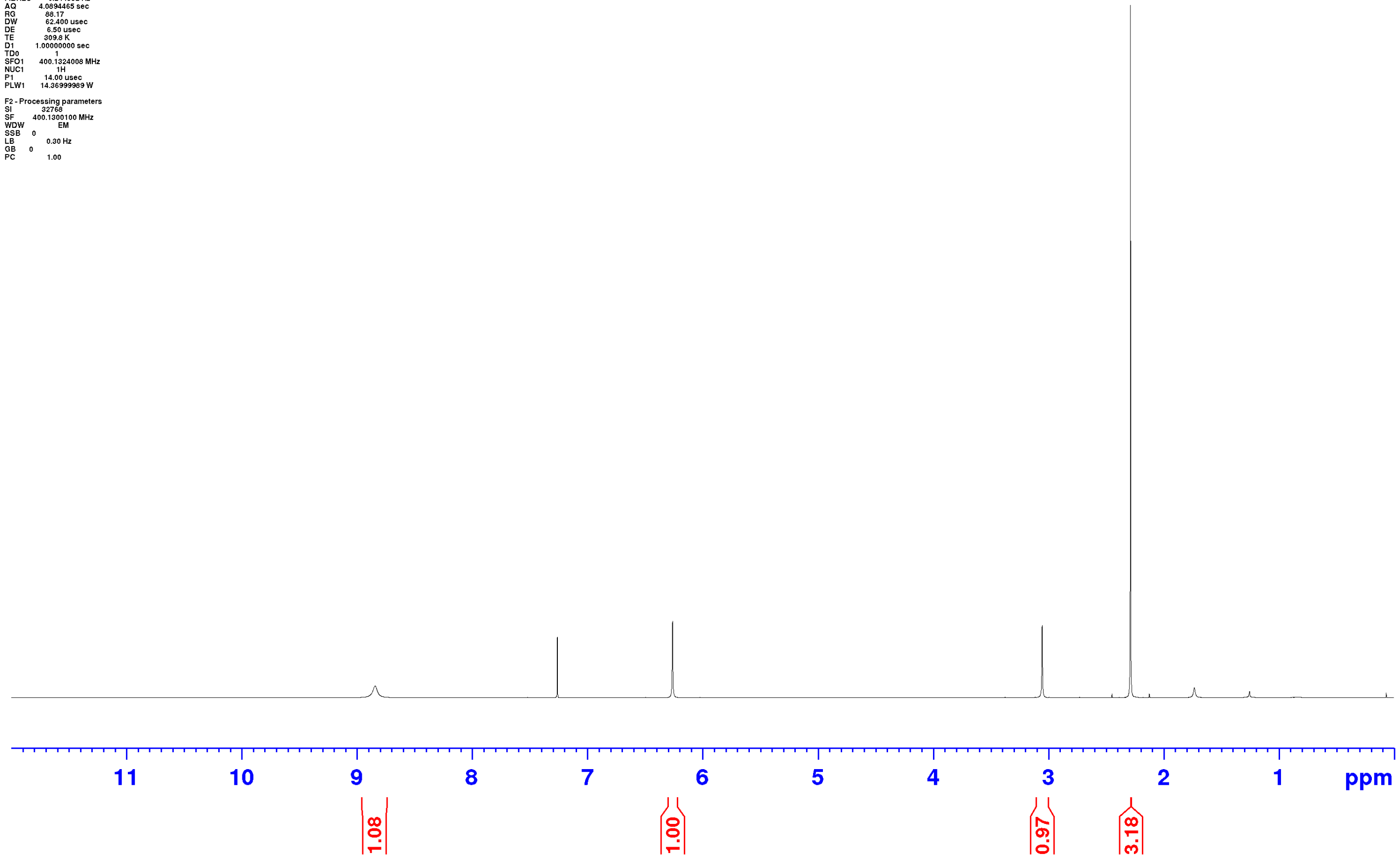

N-(3-Methylisoxazol-5-yl)prop-2-ynamide (19)

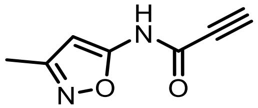


```
Current Data Parameters
NAME     Compound 5 - Aug29-2019-58-AMTA88_Recryst
EXPNO                   2
PROCNO                  1

F2 - Acquisition Parameters
Date_            20190829
Time                19.49 h
INSTRUM            avh400
PROBHD   Z108618_0873 (
PULPROG            zgpg30
TD                  32768
SOLVENT             CDCl3
NS                    512
DS                      4
SWH             26041.666 Hz
FIDRES           1.589457 Hz
AQ              0.6291456 sec
RG                 197.18
DW                 19.200 usec
DE                   6.50 usec
TE                  311.0 K
D1             1.00000000 sec
D11            0.03000000 sec
TD0                     1
SFO1          100.6228298 MHz
NUC1                  13C
P1                  10.00 usec
PLW1          47.86100006 W
SFO2          400.1316005 MHz
NUC2                   1H
CPDPRG[2          waltz16
PCPD2               90.00 usec
PLW2          14.36999989 W
PLW12          0.34660661 W
PLW13          0.17371930 W

F2 - Processing parameters
SI                  32768
SF            100.6127538 MHz
WDW                    EM
SSB      0
LB                   1.00 Hz
GB       0
PC                   1.40
```

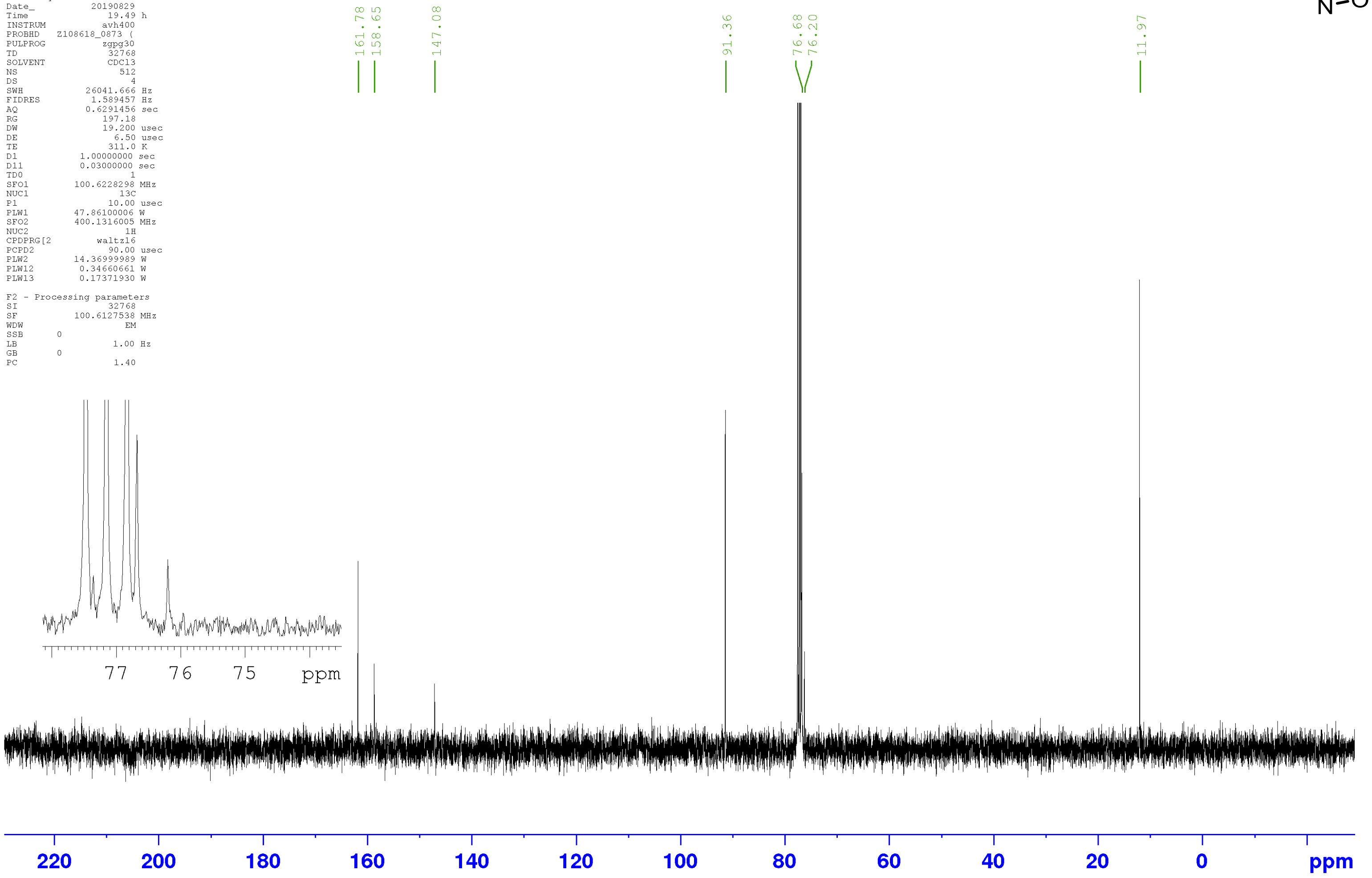

N-(3-Methylisoxazol-5-yl)but-2-ynamide (20)

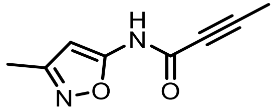

Current Data Parameters
NAME Compound 6 - Aug29-2019-57-AMTB56_Recryst
EXPNO 1
PROCNO 1

F2 - Acquisition Parameters
Date_ 20190829
Time 18.57 h
INSTRUM avh400
PROBHD Z108618_0873 (
PULPROG zg60
TD 65536
SOLVENT CDCl3
NS 16
DS 2
SWH 8012.820 Hz
FIDRES 0.244532 Hz
AQ 4.0894465 sec
RG 53.51
DW 62.400 usec
DE 6.50 usec
TE 309.9 K
D1 1.00000000 sec
TD0 1
SFO1 400.1324008 MHz
NUC1 1H
P1 14.00 usec
PLW1 14.36999989 W

F2 - Processing parameters
SI 32768
SF 400.1300099 MHz
WDW EM
SSB 0
LB 0.30 Hz
GB 0
PC 1.00

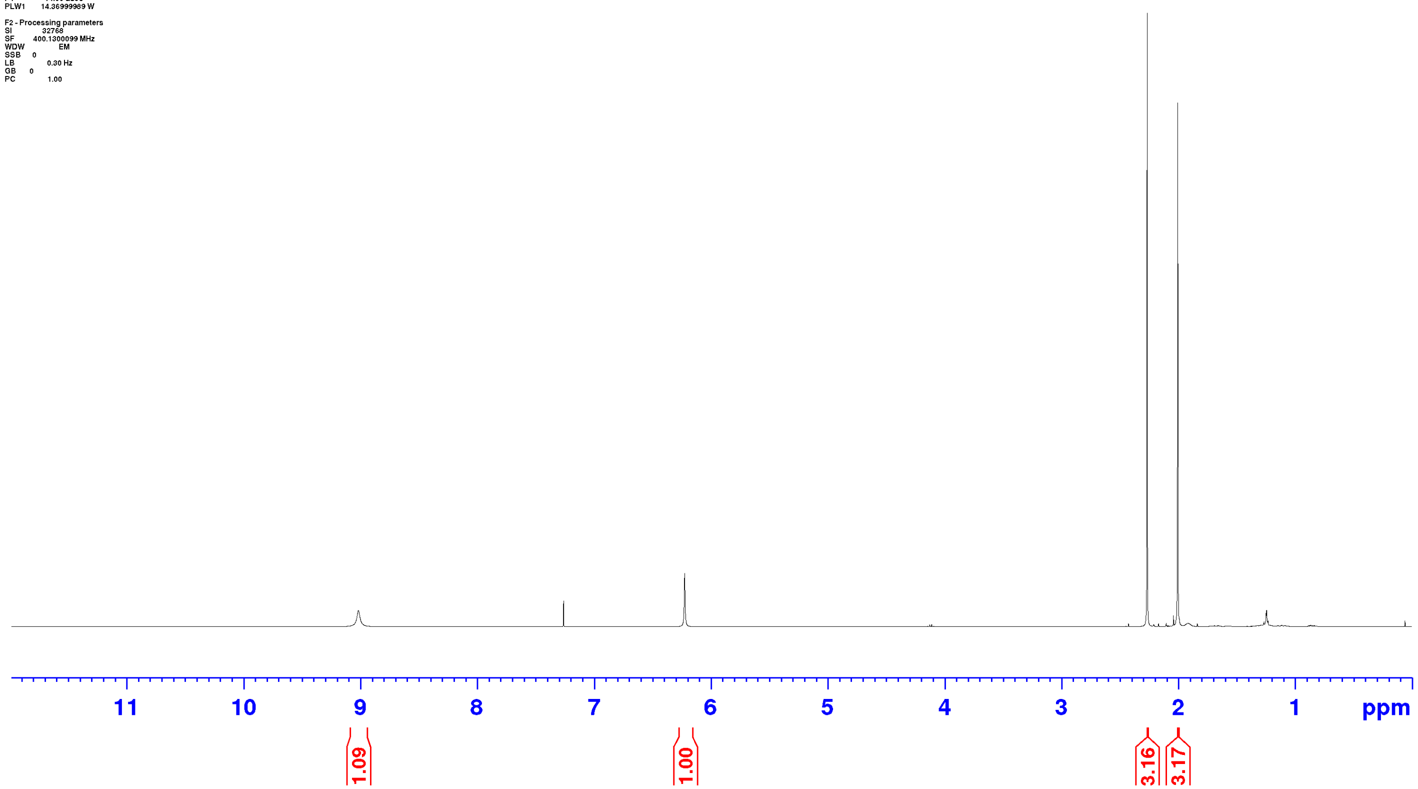

N-(3-Methylisoxazol-5-yl)but-2-ynamide (20)

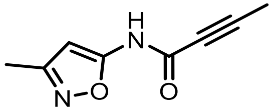


```
Current Data Parameters
NAME     Compound 6 - Aug29-2019-57-AMTB56_Recryst
EXPNO                 4
PROCNO                1

F2 - Acquisition Parameters
Date_           20190829
Time               19.31 h
INSTRUM           avh400
PROBHD   Z108618_0873 (
PULPROG          zgpg30
TD                32768
SOLVENT           CDCl3
NS                  512
DS                    4
SWH           26041.666 Hz
FIDRES         1.589457 Hz
AQ            0.6291456 sec
RG               197.18
DW               19.200 usec
DE                 6.50 usec
TE                310.9 K
D1           1.00000000 sec
D11          0.03000000 sec
TD0                   1
SFO1        100.6228298 MHz
NUC1                13C
P1                10.00 usec
PLW1        47.86100006 W
SFO2        400.1316005 MHz
NUC2                 1H
CPDPRG[2        waltz16
PCPD2             90.00 usec
PLW2        14.36999989 W
PLW12        0.34660661 W
PLW13        0.17371930 W

F2 - Processing parameters
SI                32768
SF          100.6127564 MHz
WDW                  EM
SSB      0
LB                 1.00 Hz
GB       0
PC                 1.40
```

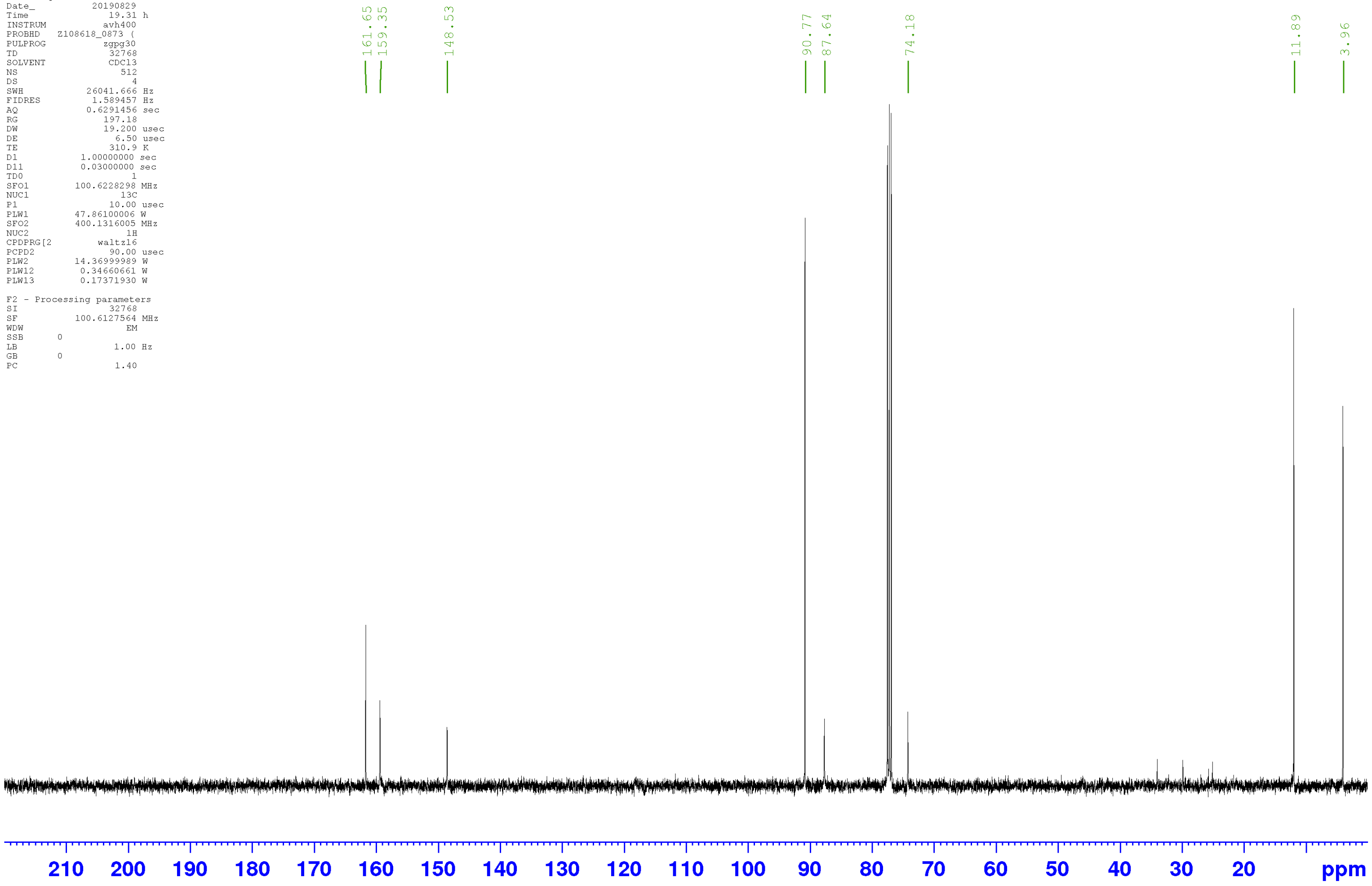

2-Chloro-*N*-(3-methylisoxazol-5-yl)acetamide (21)

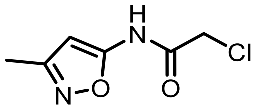

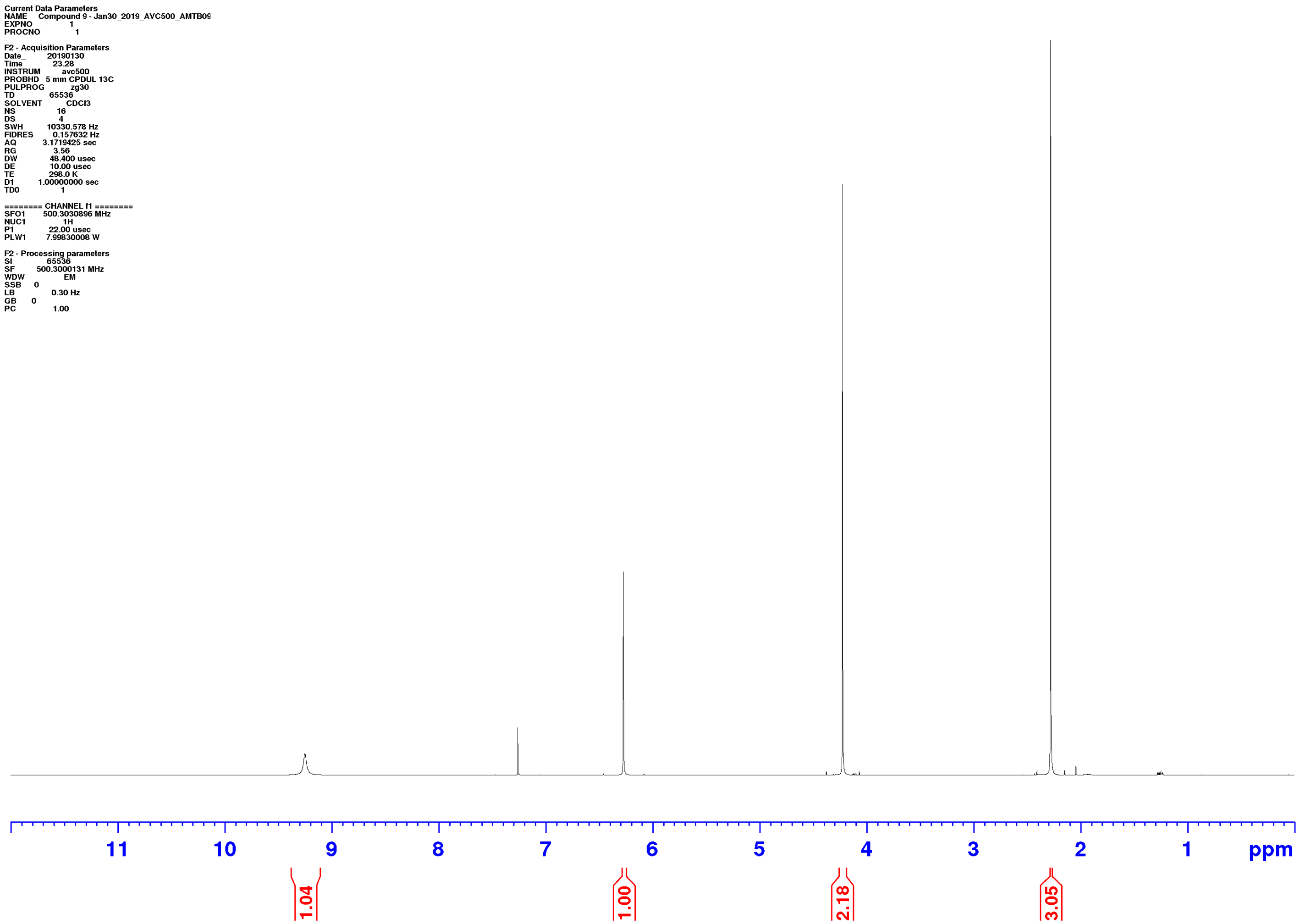

2-Chloro-*N*-(3-methylisoxazol-5-yl)acetamide (21)

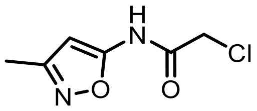

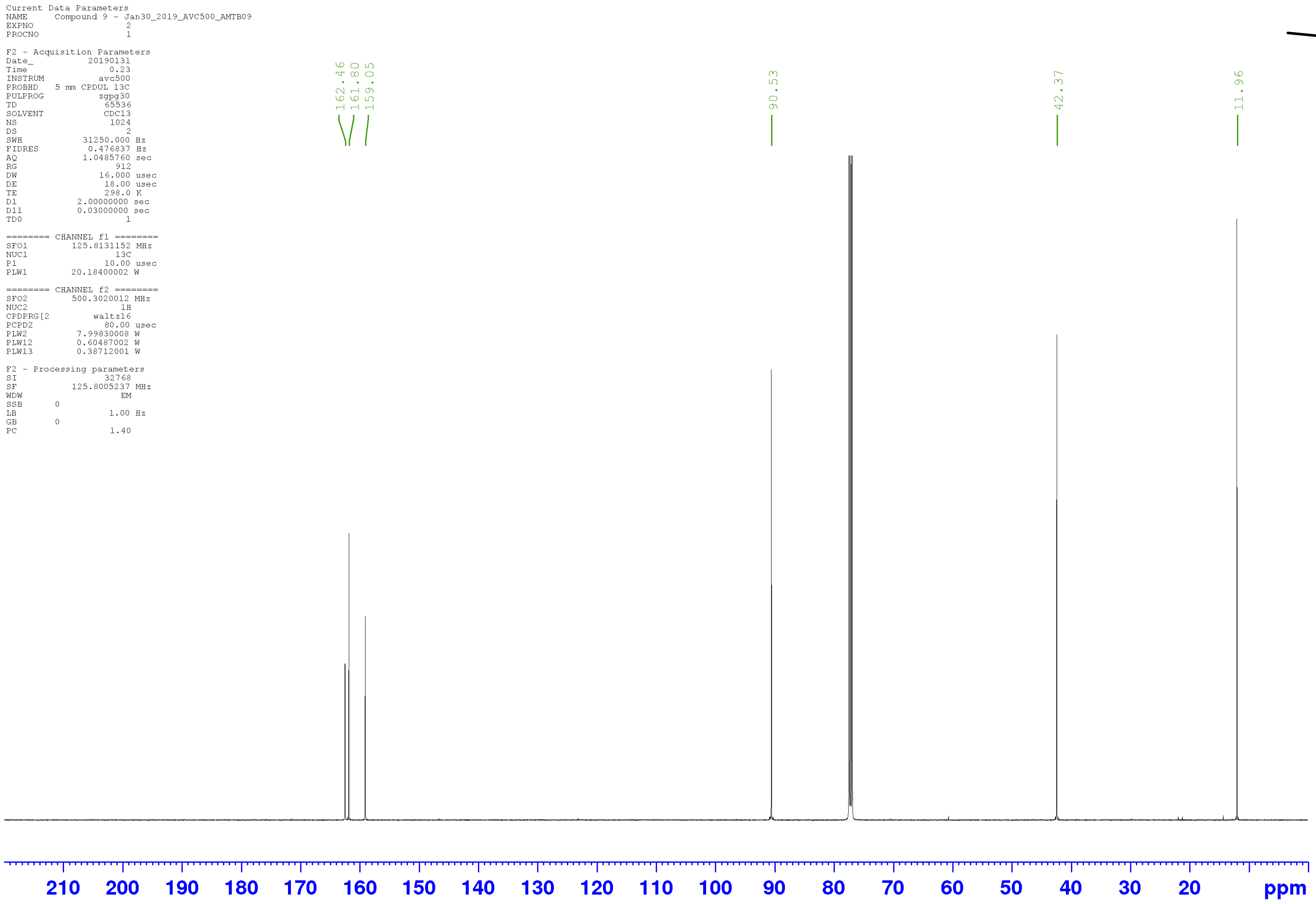

2-Chloro-*N*-(3-methyl-4-phenylisoxazol-5-yl)acetamide (22)

Current Data Parameters
NAME at76851002
EXPNO 1
PROCNO 1

F2 - Acquisition Parameters
Date_ 20200211
Time 5.44
INSTRUM avc500
PROBHD 5 mm CPDUL 13C
PULPROG zg30
TD 65536
SOLVENT CDCl3
NS 16
DS 4
SWH 10330.578 Hz
FIDRES 0.157632 Hz
AQ 3.1719425 sec
RG 4
DW 48.400 usec
DE 10.00 usec
TE 298.0 K
D1 1.00000000 sec
TD0 1

======== CHANNEL f1 ========
SFO1 500.3030896 MHz
NUC1 1H
P1 22.00 usec
PLW1 7.99830008 W

F2 - Processing parameters
SI 65536
SF 500.3000133 MHz
WDW EM
SSB 0
LB 0.30 Hz
GB 0
PC 1.00

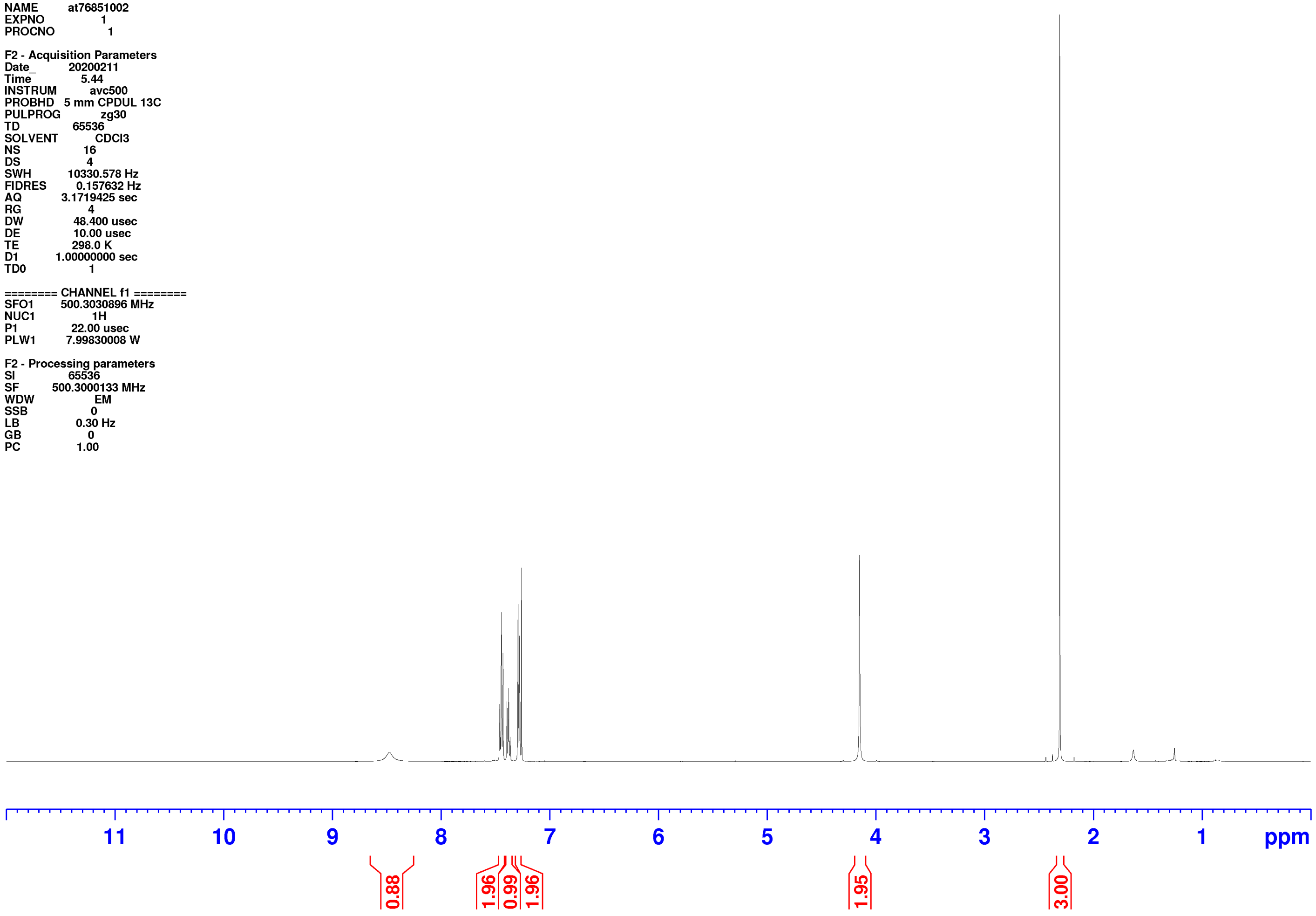

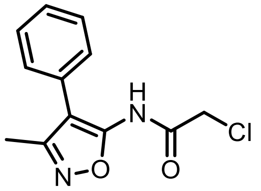

2-Chloro-*N*-(3-methyl-4-phenylisoxazol-5-yl)acetamide (22)

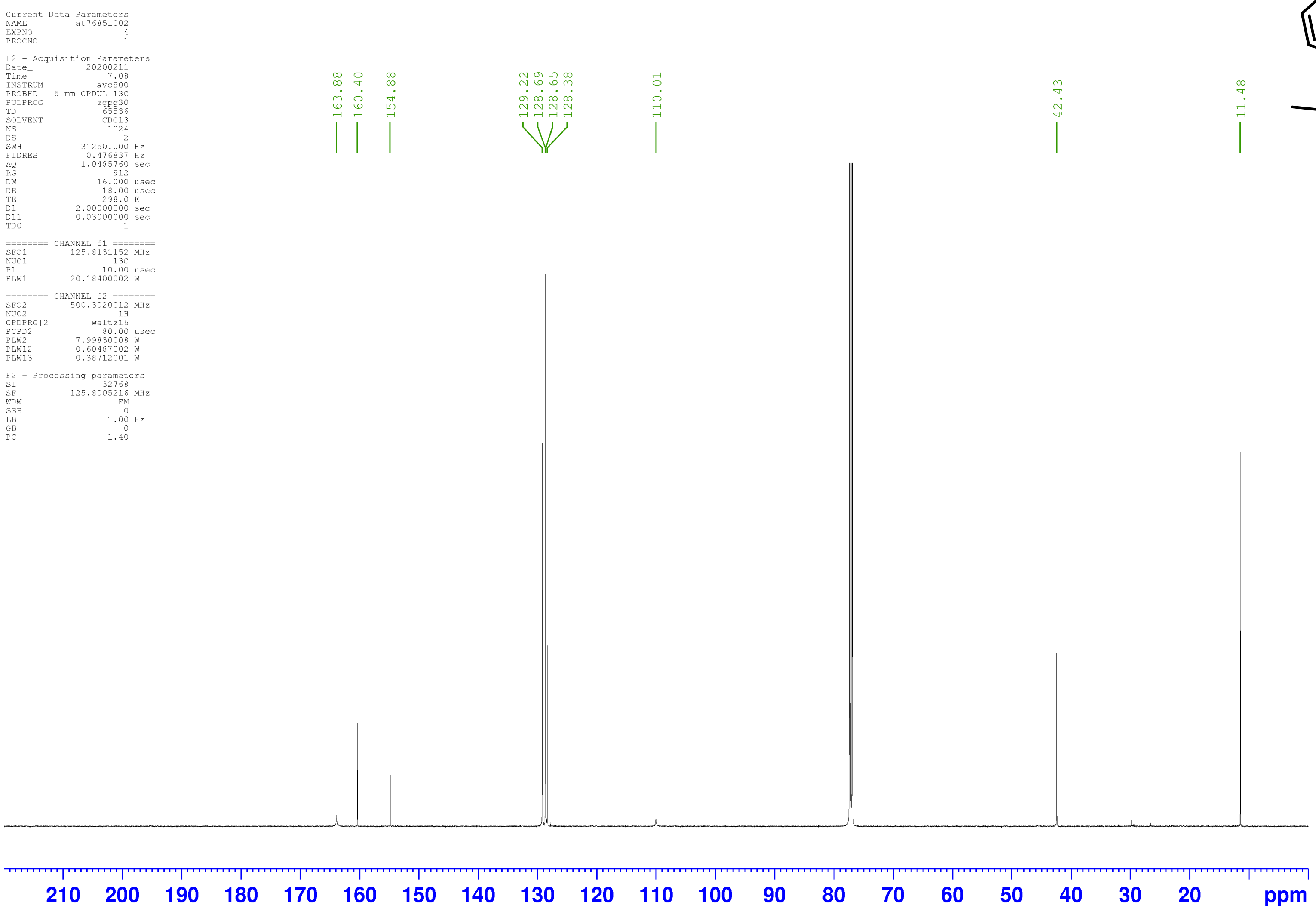

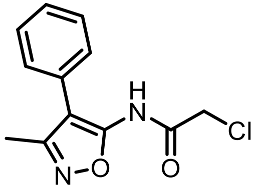

N-(3-Methylisoxazol-5-yl)ethenesulfonamide (23)

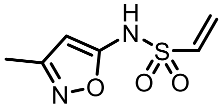

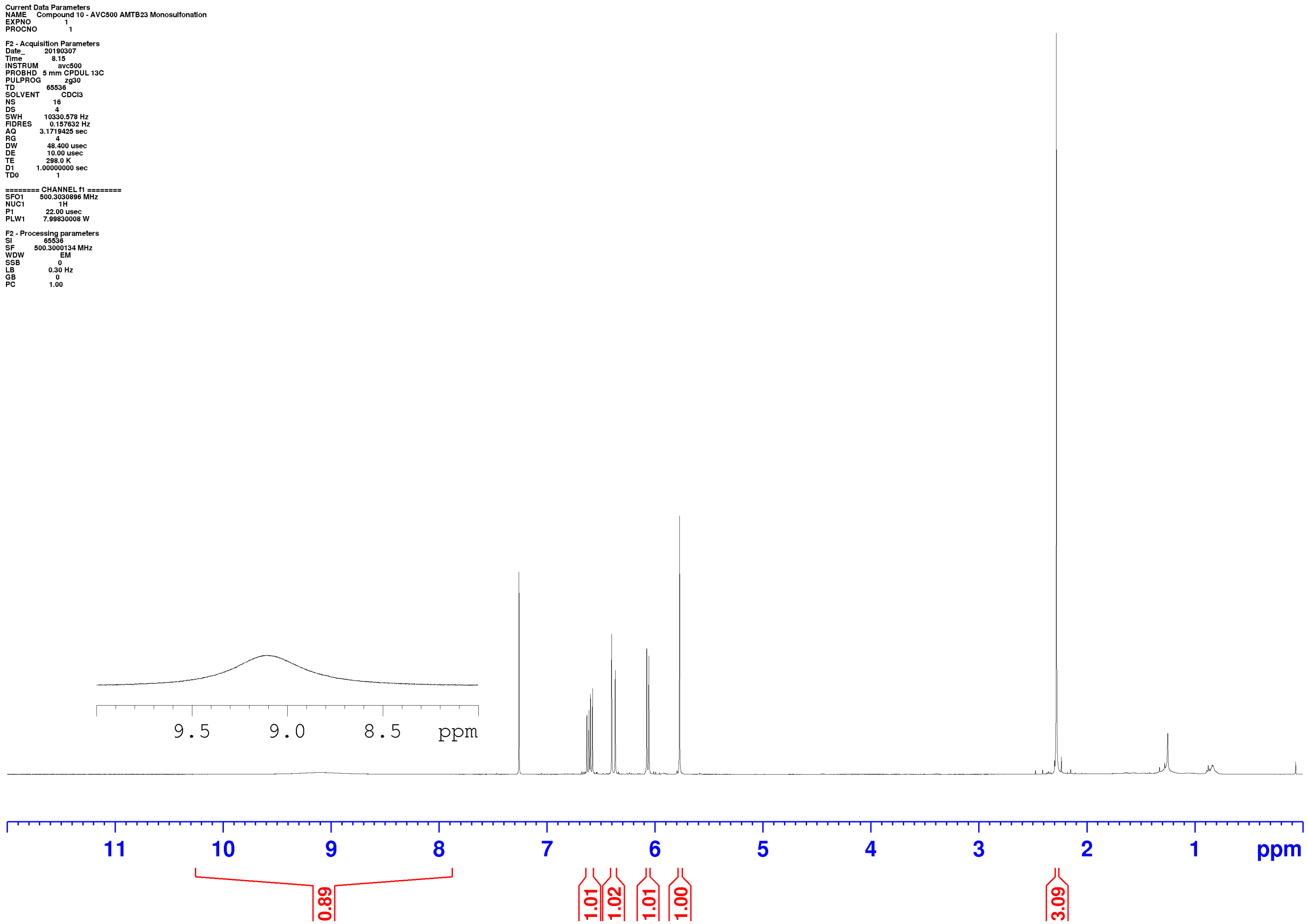

N-(3-Methylisoxazol-5-yl)ethenesulfonamide (23)

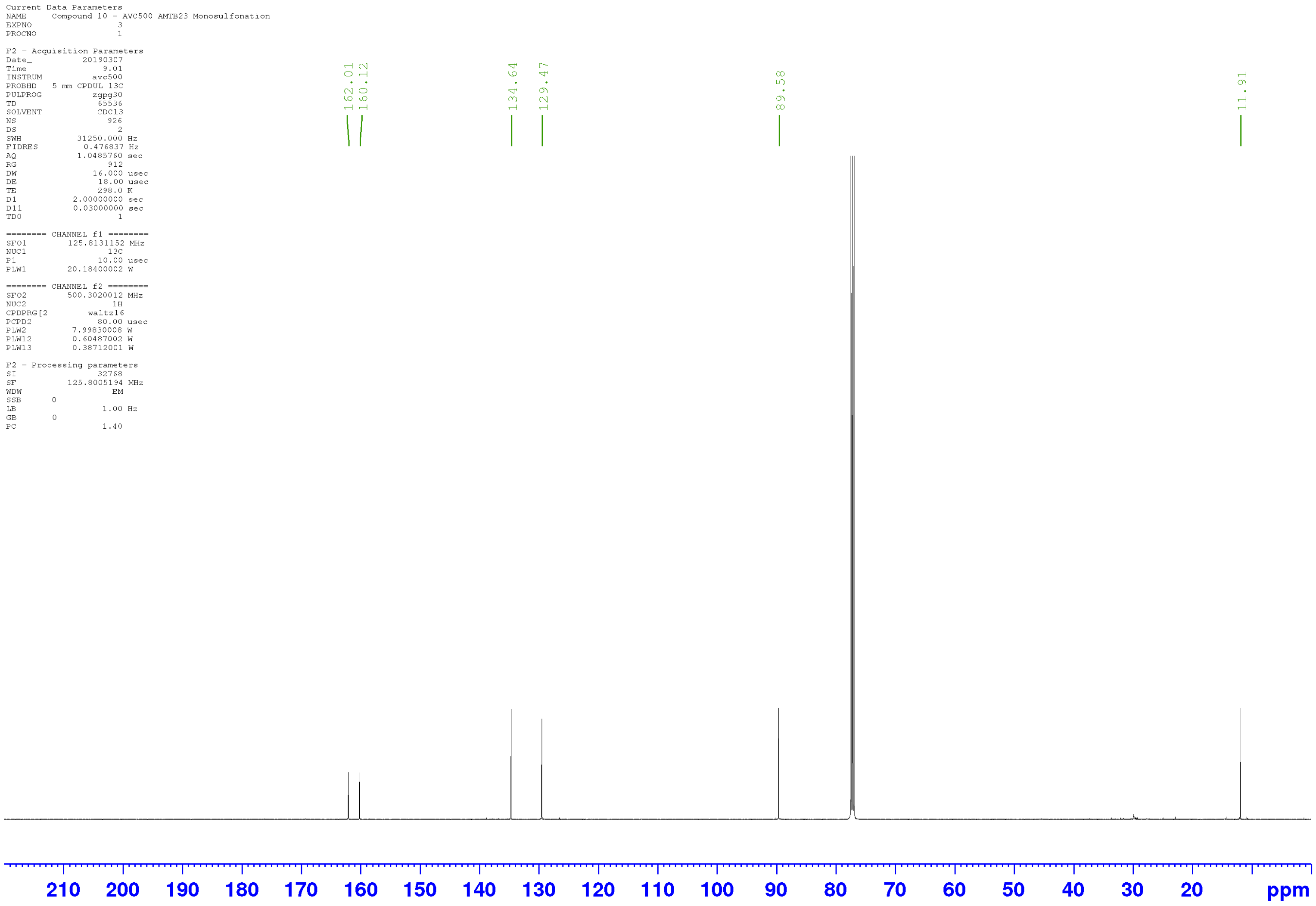

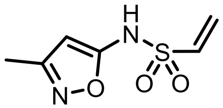

N-(3-Methyl-isoxazol-5-yl)propanamide (24)

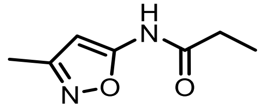

Current Data Parameters
NAME at73431612
EXPNO 1
PROCNO 1

F2 - Acquisition Parameters
Date_ 20191220
Time 0.21
INSTRUM avc500
PROBHD 5 mm CPDUL 13C
PULPROG zg30
TD 65536
SOLVENT CDCl3
NS 16
DS 4
SWH 10330.578 Hz
FIDRES 0.157632 Hz
AQ 3.1719425 sec
RG 4
DW 48.400 usec
DE 10.00 usec
TE 298.0 K
D1 1.00000000 sec
TD0 1

======== CHANNEL f1 ========
SFO1 500.3030896 MHz
NUC1 1H
P1 22.00 usec
PLW1 7.99830008 W

F2 - Processing parameters
SI 65536
SF 500.3000137 MHz
WDW EM
SSB 0
LB 0.30 Hz
GB 0
PC 1.00

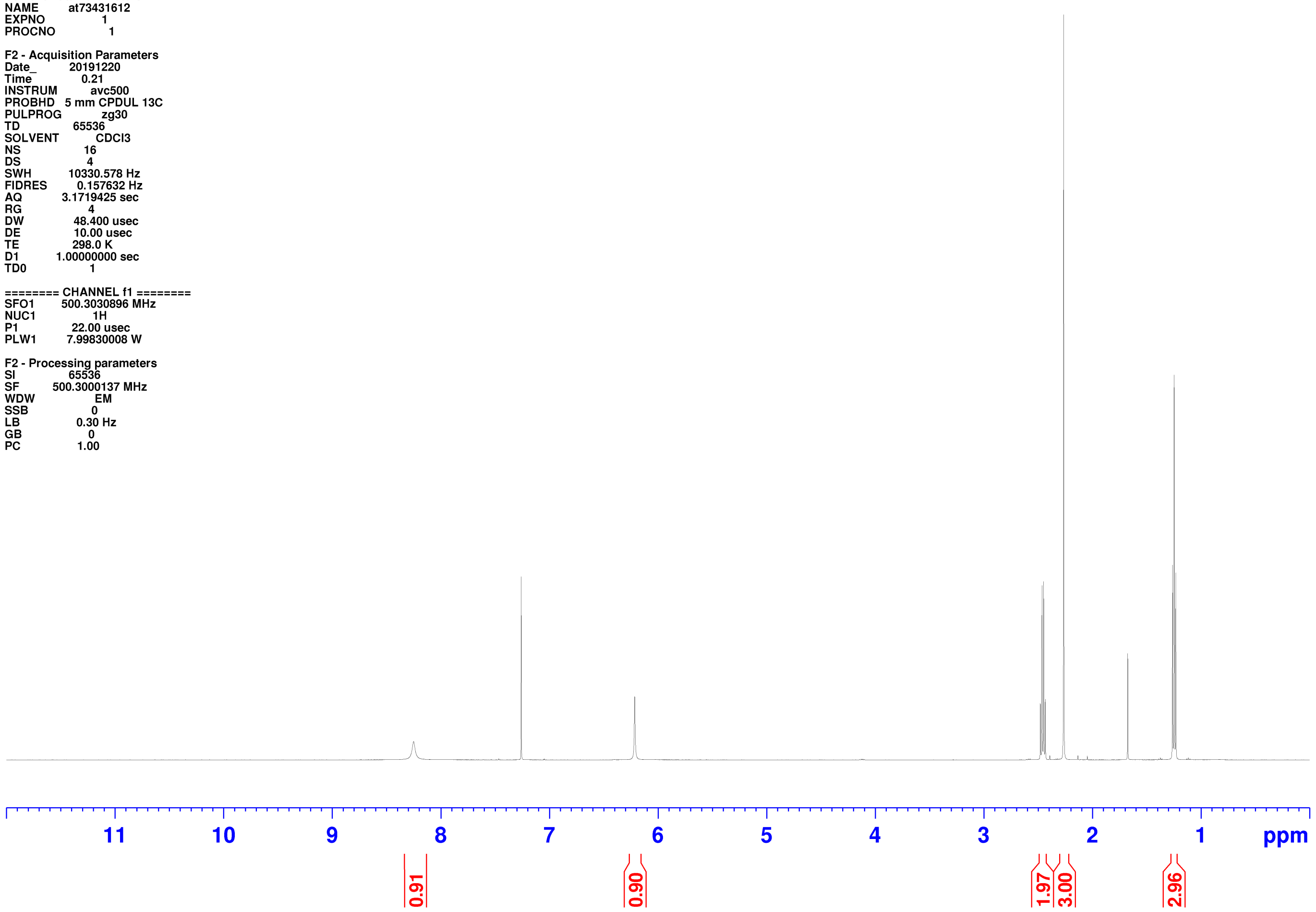

N-(3-Methyl-isoxazol-5-yl)propanamide (24)

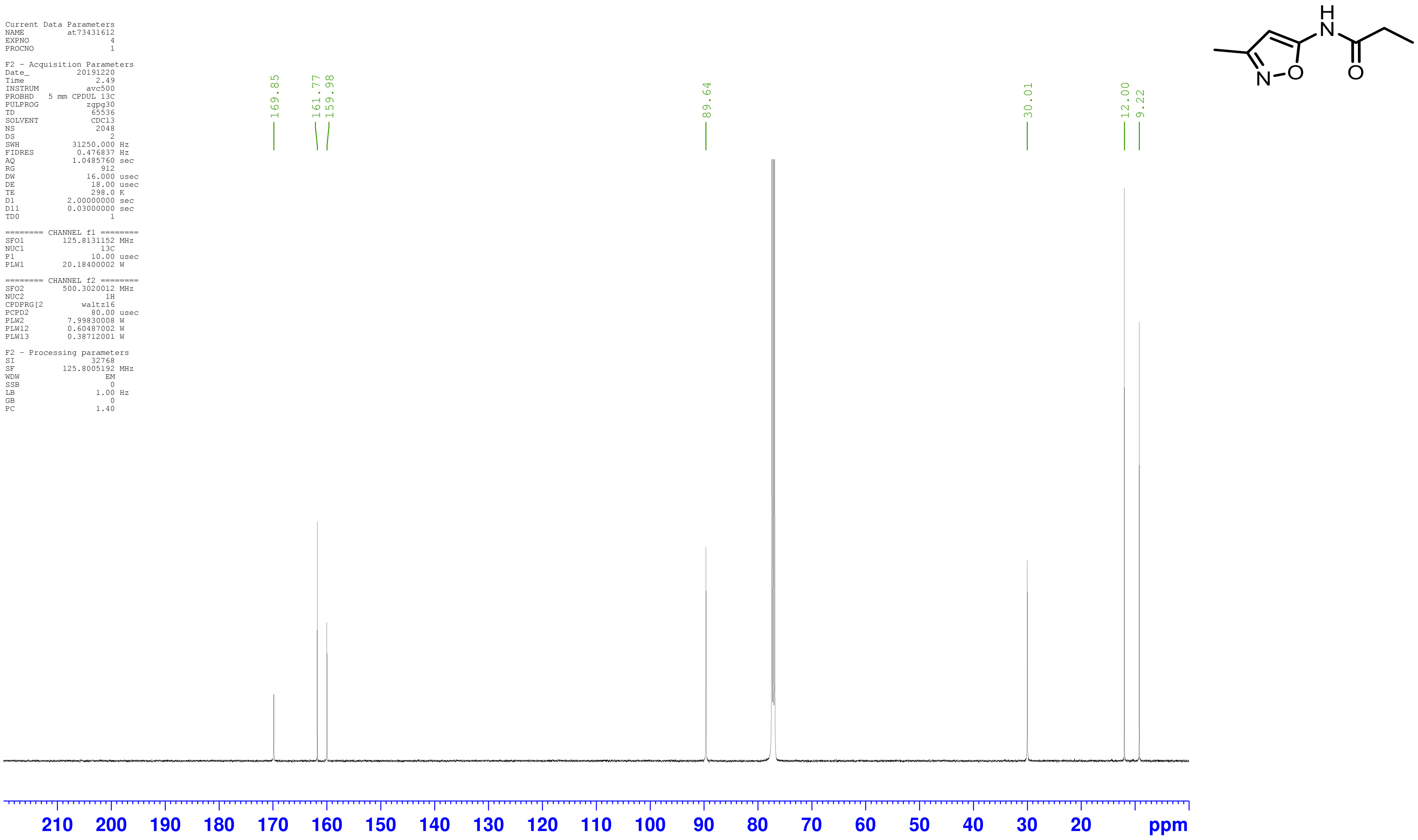

4-(Dimethylamino)-*N*-(3-methyl-4-phenylisoxazol-5-yl)butanamide (25)

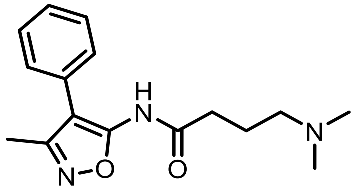

Current Data Parameters
NAME at625510306
EXPNO 1
PROCNO 1

F2 - Acquisition Parameters
Date_ 20210606
Time 9.20 h
INSTRUM Avance
PROBHD Z159656_0020 (
PULPROG zg30
TD 65536
SOLVENT CDCl3
NS 16
DS 2
SWH 11904.762 Hz
FIDRES 0.363304 Hz
AQ 2.7525120 sec
RG 58.3812
DW 42.000 usec
DE 22.00 usec
TE 298.0 K
D1 1.00000000 sec
TD0 1
SFO1 600.4230021 MHz
NUC1 1H
P0 4.00 usec
P1 12.00 usec
PLW1 13.51200008 W

F2 - Processing parameters
SI 65536
SF 600.4200149 MHz
WDW EM
SSB 0
LB 0.30 Hz
GB 0
PC 1.00

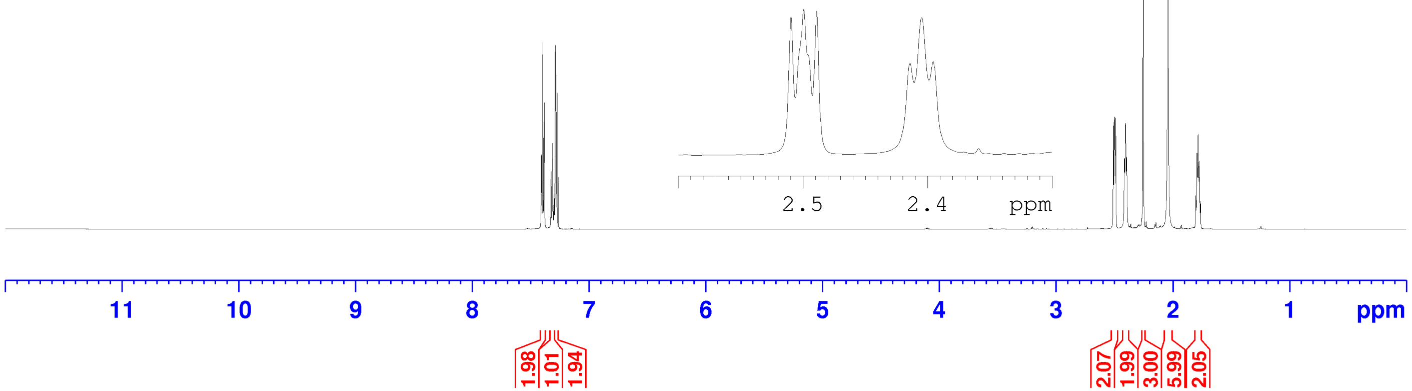

4-(Dimethylamino)-*N*-(3-methyl-4-phenylisoxazol-5-yl)butanamide (25)

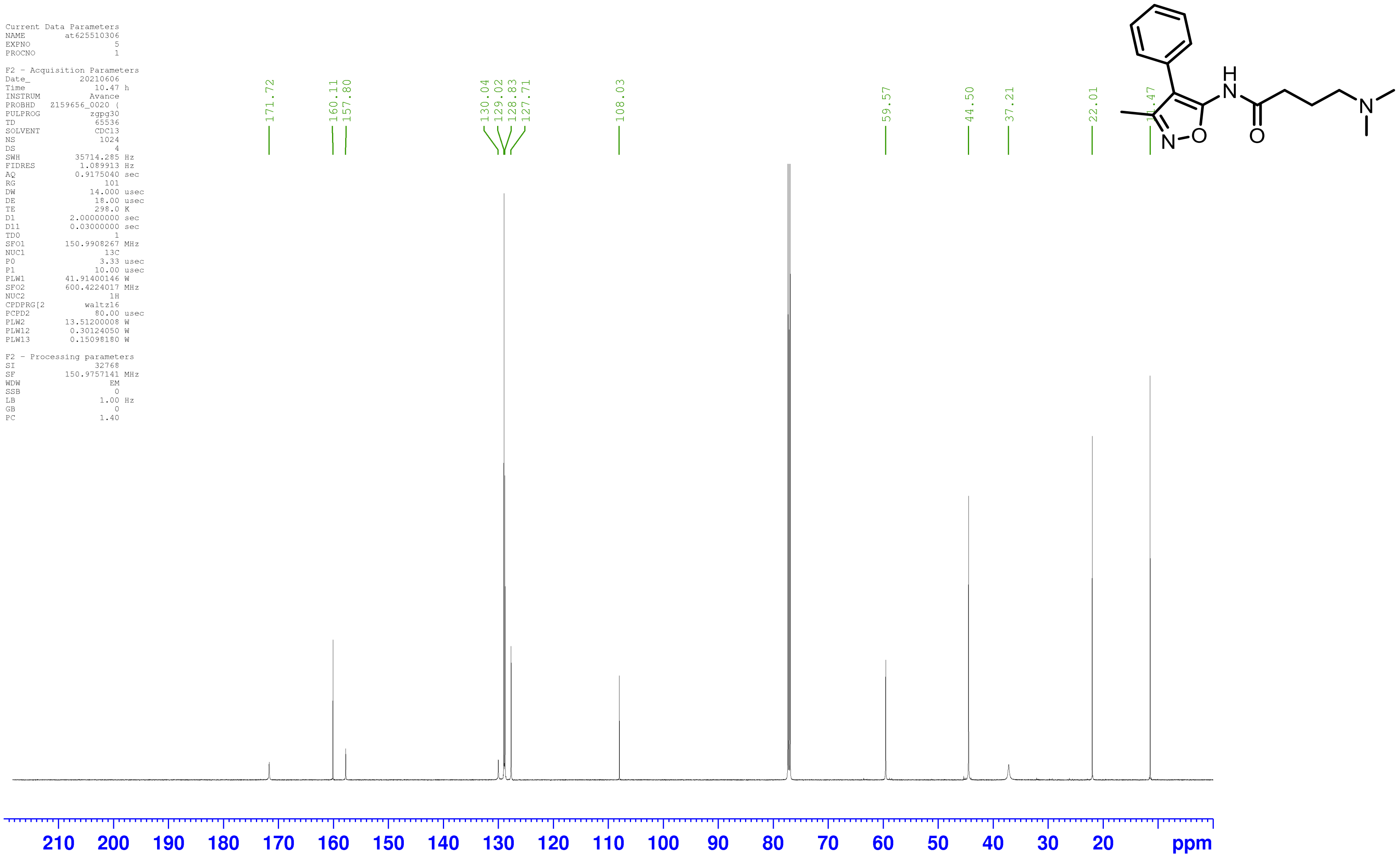

2-(4-Iodophenyl)-3-oxobutanenitrile (32)

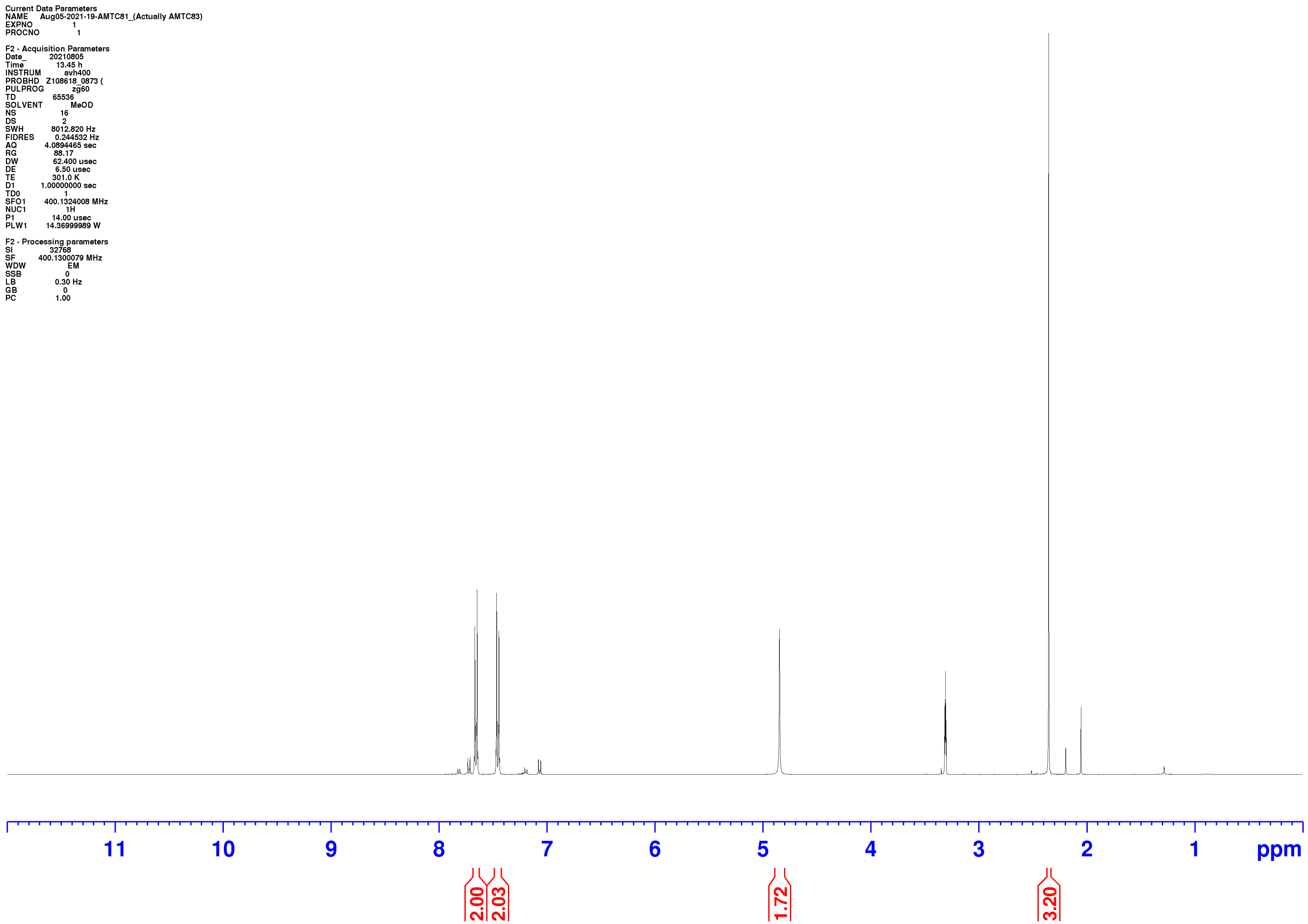

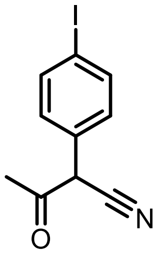

4-(4-Iodophenyl)-3-methylisoxazol-5-amine (33)

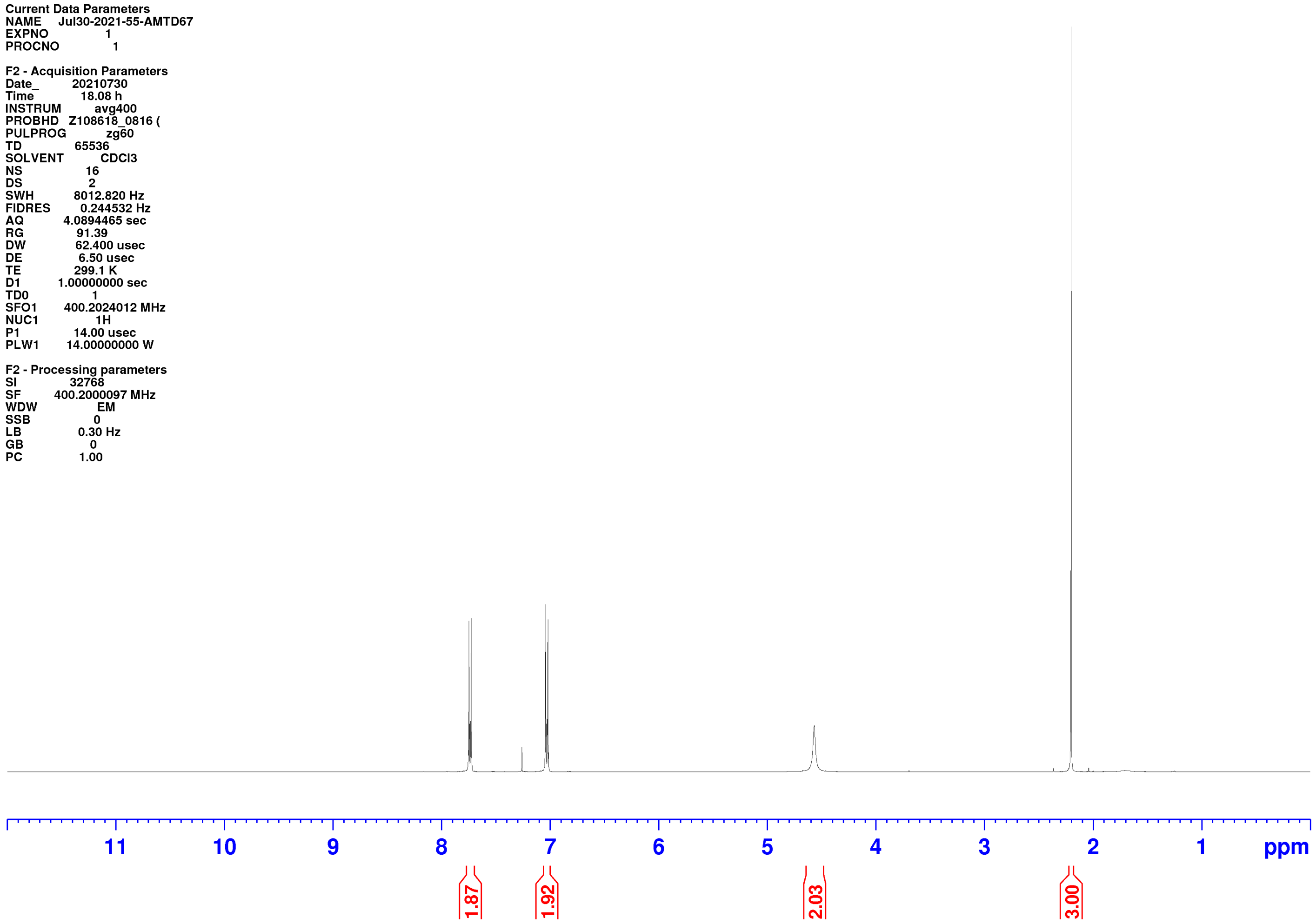

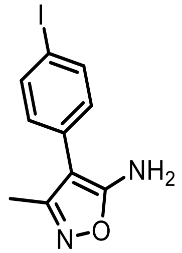

4-(4-Iodophenyl)-3-methylisoxazol-5-amine (33)

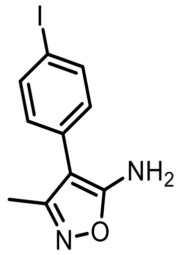


```
Current Data Parameters
NAME       Jul30-2021-55-AMTD67
EXPNO                     2
PROCNO                    1

F2 - Acquisition Parameters
Date_              20210803
Time                   3.51 h
INSTRUM              avg400
PROBHD   Z108618_0816 (
PULPROG              zgpg30
TD                    32768
SOLVENT               CDCl3
NS                      512
DS                        4
SWH               26041.666 Hz
FIDRES             1.589457 Hz
AQ                0.6291456 sec
RG                   206.87
DW                   19.200 usec
DE                     6.50 usec
TE                    300.5 K
D1               1.00000000 sec
D11              0.03000000 sec
TD0                       1
SFO1            100.6404331 MHz
NUC1                    13C
P0                     3.33 usec
P1                    10.00 usec
PLW1            56.00000000 W
SFO2            400.2016008 MHz
NUC2                     1H
CPDPRG[2            waltz16
PCPD2                 90.00 usec
PLW2            14.00000000 W
PLW12            0.33877000 W
PLW13            0.17039999 W

F2 - Processing parameters
SI                    32768
SF              100.6303590 MHz
WDW                      EM
SSB                       0
LB                     1.00 Hz
GB                        0
PC                     1.40
```

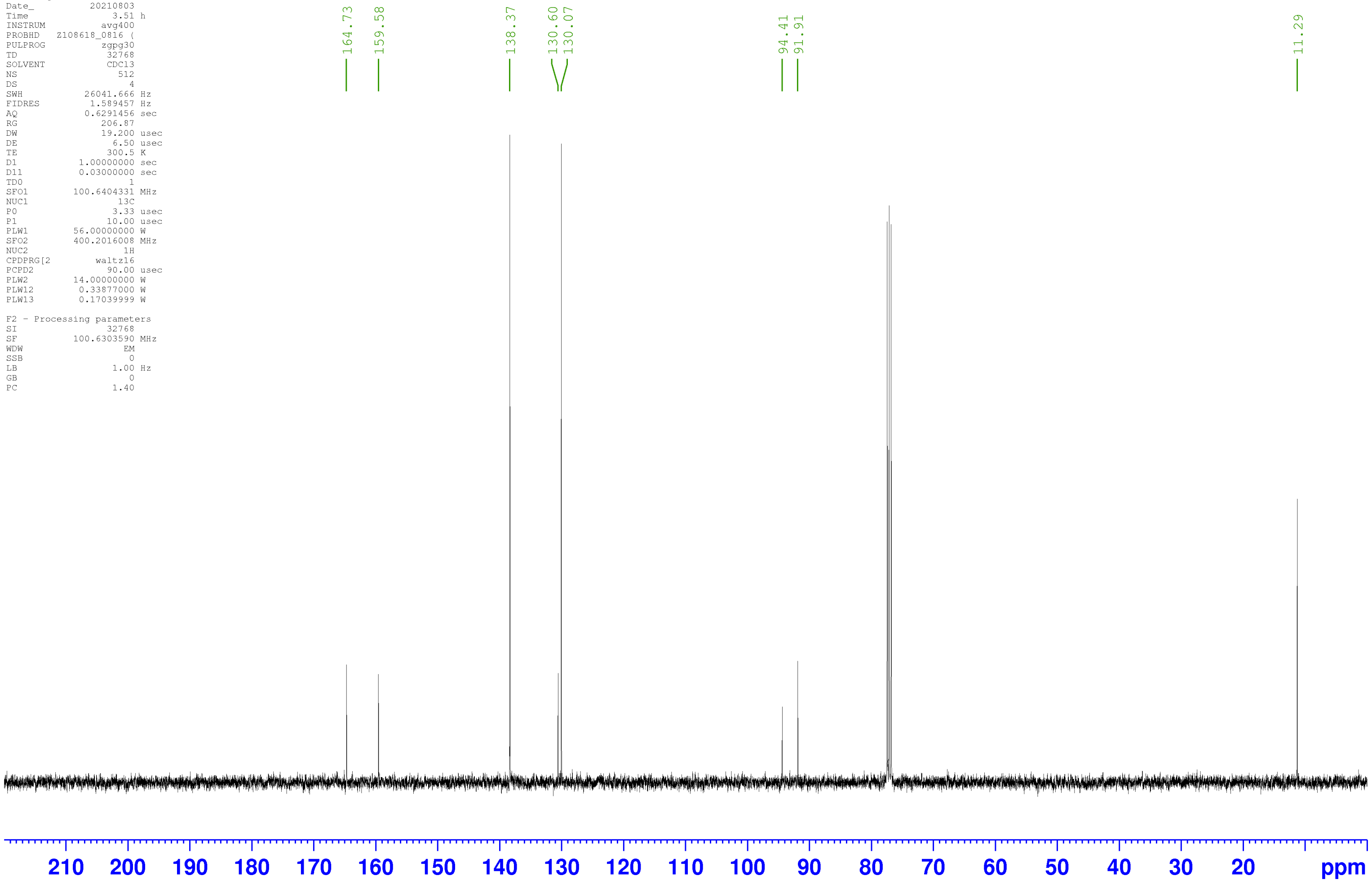

3-Methyl-4-{4-[(trimethylsilyl)ethynyl]phenyl}isoxazol-5-amine (34)

Current Data Parameters
NAME at630962207
EXPNO 1
PROCNO 1

F2 - Acquisition Parameters
Date_ 20210722
Time 10.32 h
INSTRUM Avance
PROBHD Z159656_0020 (
PULPROG zg30
TD 65536
SOLVENT CDCl3
NS 16
DS 2
SWH 11904.762 Hz
FIDRES 0.363304 Hz
AQ 2.7525120 sec
RG 55.4753
DW 42.000 usec
DE 22.00 usec
TE 298.0 K
D1 1.00000000 sec
TD0 1
SFO1 600.4230021 MHz
NUC1 1H
P0 4.00 usec
P1 12.00 usec
PLW1 13.51200008 W

F2 - Processing parameters
SI 65536
SF 600.4200143 MHz
WDW EM
SSB 0
LB 0.30 Hz
GB 0
PC 1.00

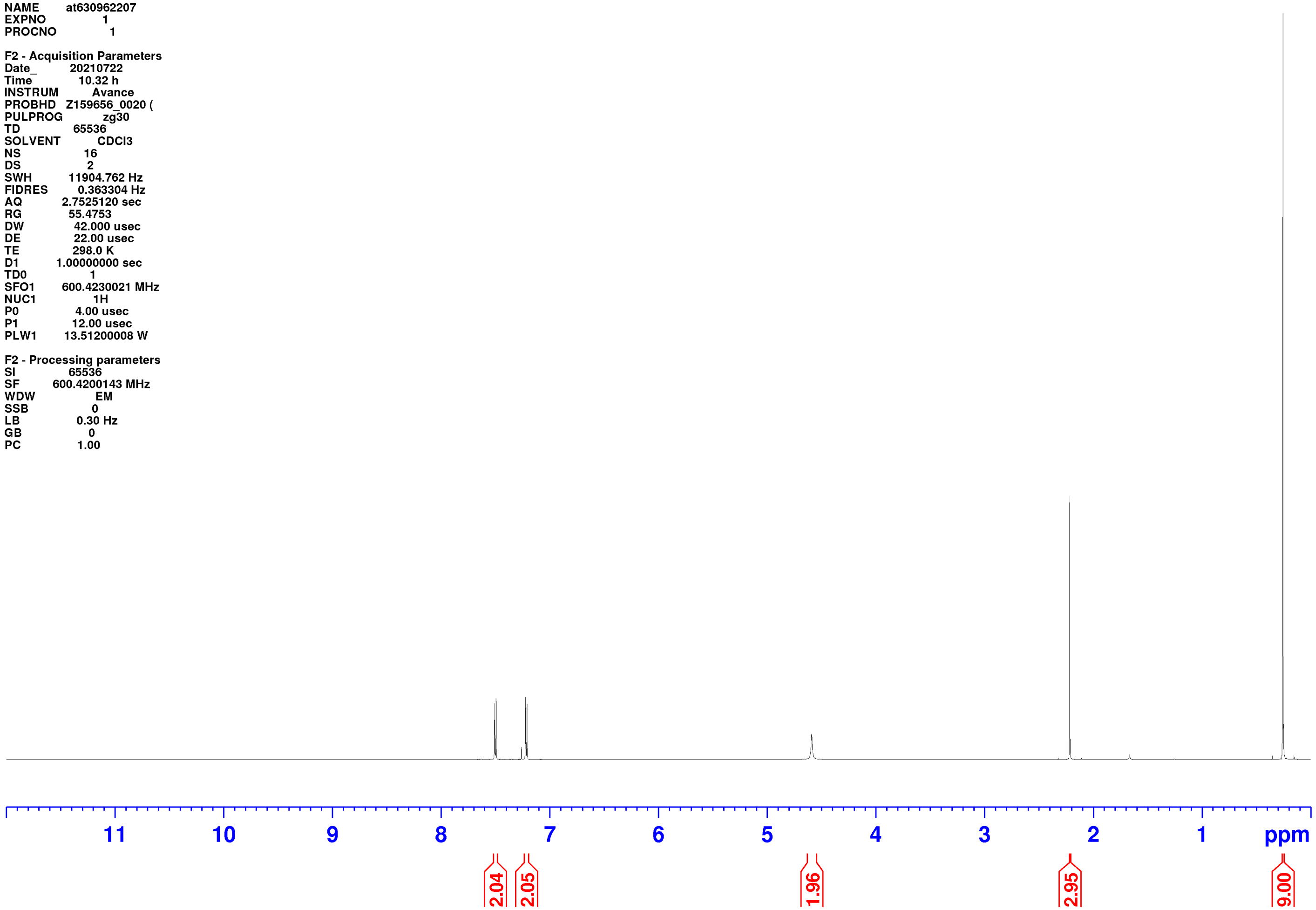

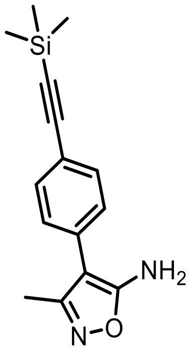

3-Methyl-4-{4-[(trimethylsilyl)ethynyl]phenyl}isoxazol-5-amine (34)

```
Current Data Parameters
NAME          at630962207
EXPNO                   5
PROCNO                  1

F2 - Acquisition Parameters
Date_            20210722
Time                11.12 h
INSTRUM            Avance
PROBHD   Z159656_0020 (
PULPROG           zgpg30
TD                 65536
SOLVENT            CDCl3
NS                   512
DS                     4
SWH            35714.285 Hz
FIDRES          1.089913 Hz
AQ             0.9175040 sec
RG                   101
DW                14.000 usec
DE                 18.00 usec
TE                 298.0 K
D1            2.00000000 sec
D11           0.03000000 sec
TD0                    1
SFO1         150.9908267 MHz
NUC1                 13C
P0                  3.33 usec
P1                 10.00 usec
PLW1         41.91400146 W
SFO2         600.4224017 MHz
NUC2                  1H
CPDPRG[2         waltz16
PCPD2              80.00 usec
PLW2         13.51200008 W
PLW12         0.30124050 W
PLW13         0.15098180 W

F2 - Processing parameters
SI                 65536
SF           150.9757109 MHz
WDW                   EM
SSB                    0
LB                  1.00 Hz
GB                     0
PC                  1.40
```

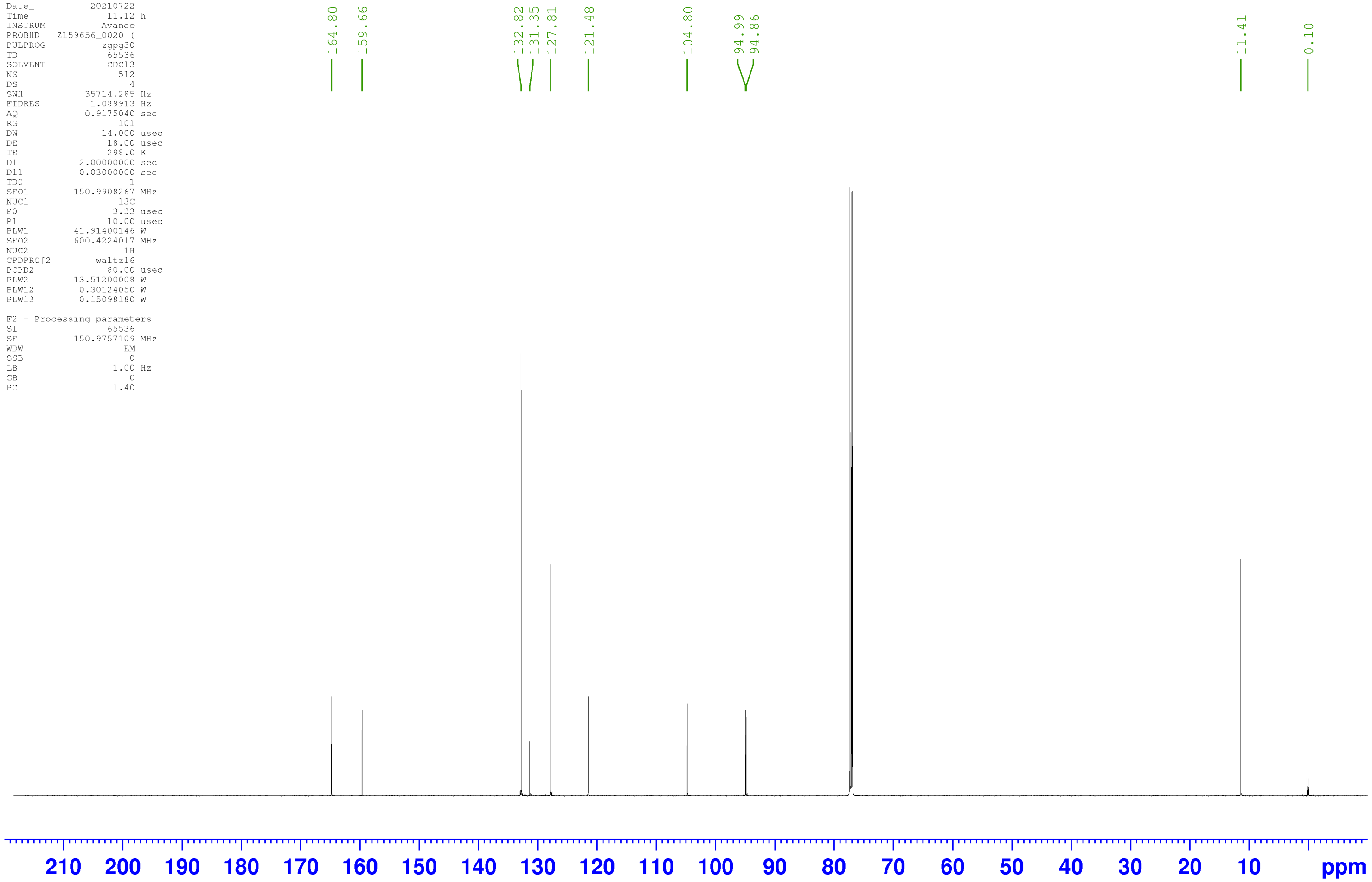

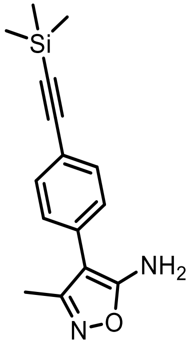

4-(4-Ethynylphenyl)-3-methylisoxazol-5-amine (35)

Current Data Parameters
NAME at630972207
EXPNO 1
PROCNO 1

F2 - Acquisition Parameters
Date_ 20210722
Time 11.20 h
INSTRUM Avance
PROBHD Z159656_0020 (
PULPROG zg30
TD 65536
SOLVENT CDCl3
NS 16
DS 2
SWH 11904.762 Hz
FIDRES 0.363304 Hz
AQ 2.7525120 sec
RG 58.3812
DW 42.000 usec
DE 22.00 usec
TE 298.0 K
D1 1.00000000 sec
TD0 1
SFO1 600.4230021 MHz
NUC1 1H
P0 4.00 usec
P1 12.00 usec
PLW1 13.51200008 W

F2 - Processing parameters
SI 65536
SF 600.4200143 MHz
WDW EM
SSB 0
LB 0.30 Hz
GB 0
PC 1.00

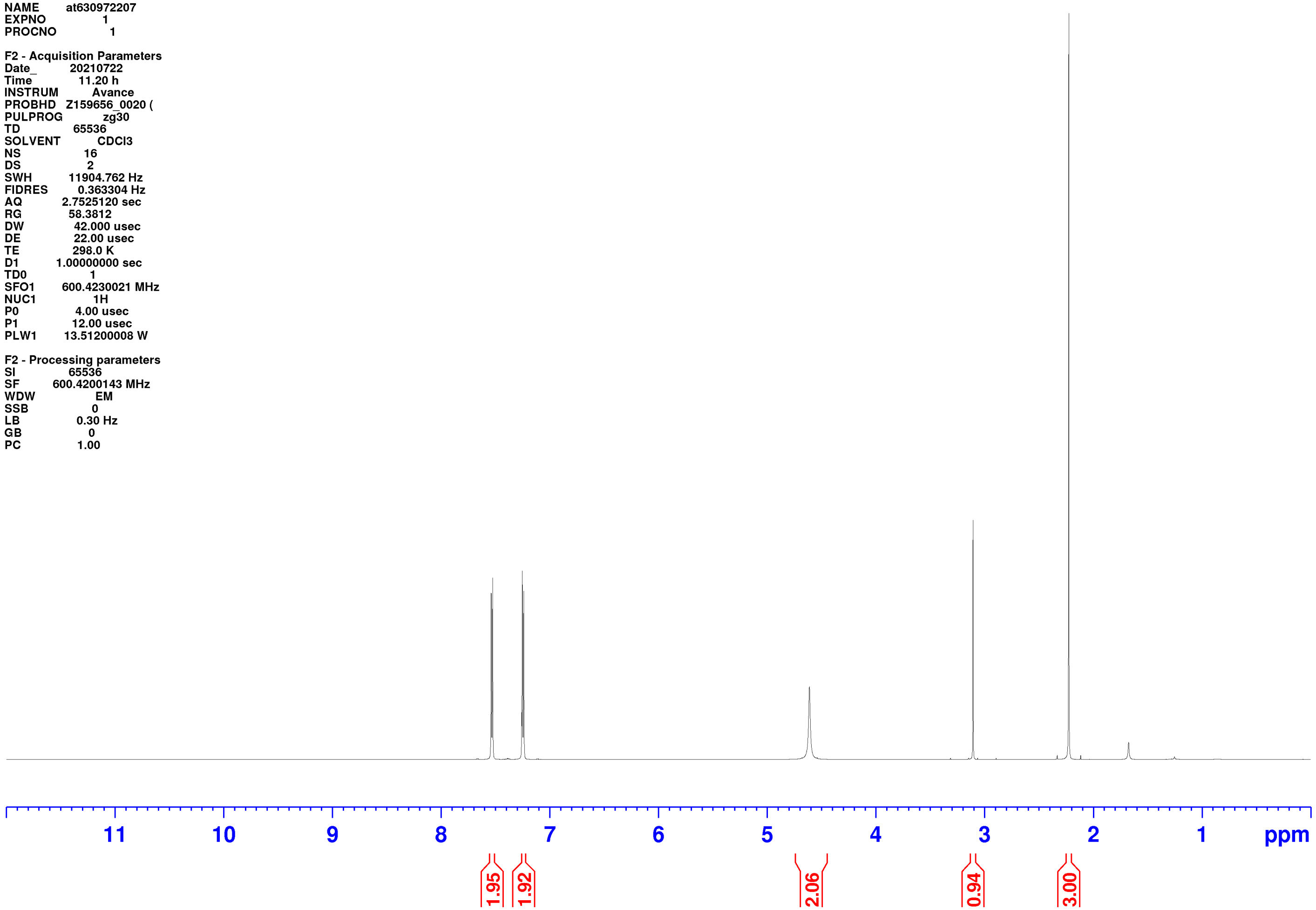

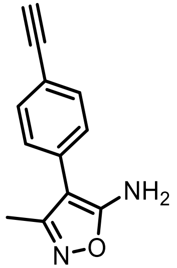

4-(4-Ethynylphenyl)-3-methylisoxazol-5-amine (35)

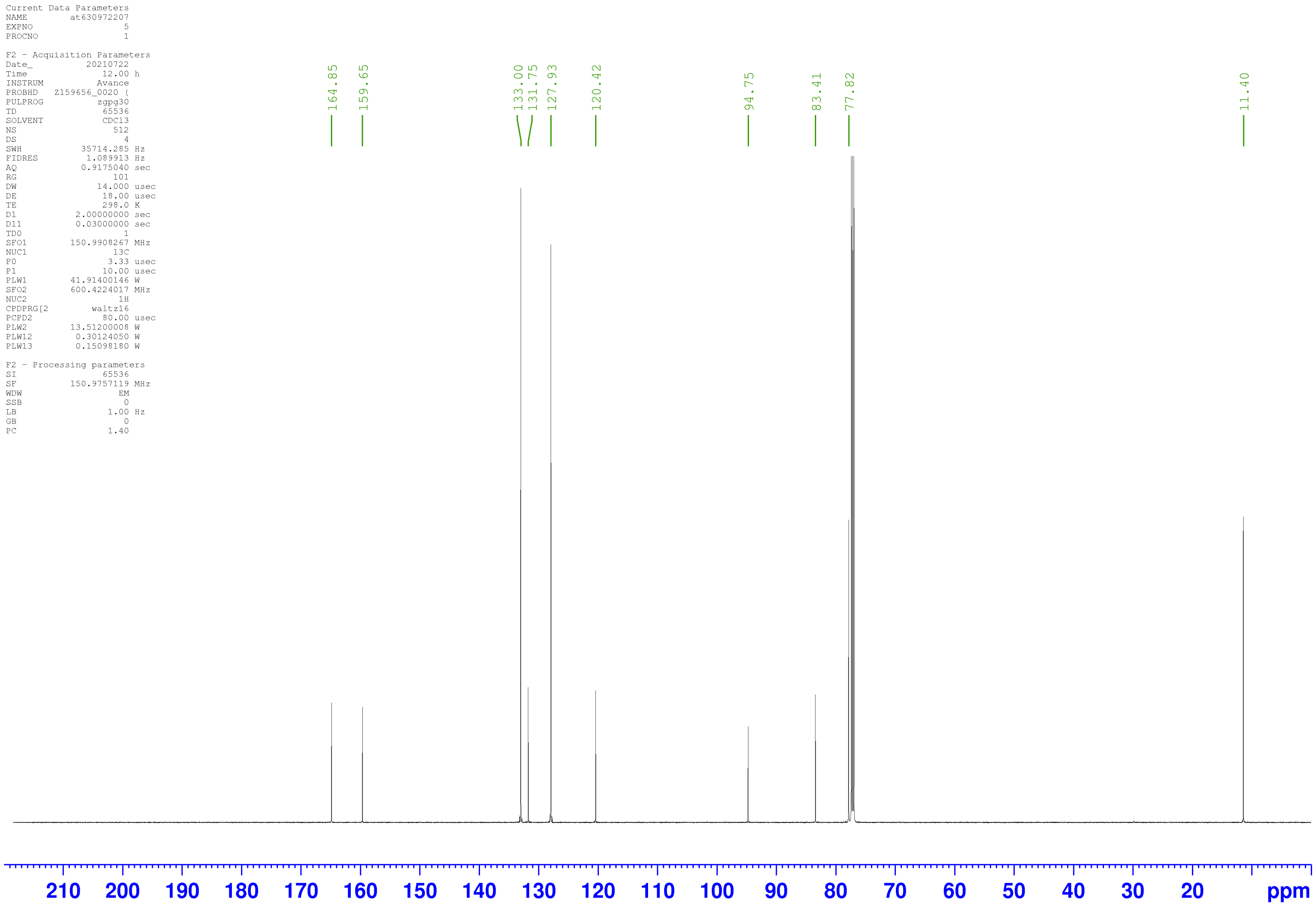

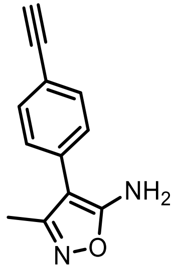

(*E*)-4-(Dimethylamino)-*N*-[4-(4-ethynylphenyl)-3-methylisoxazol-5-yl]but-2-enamide (28)

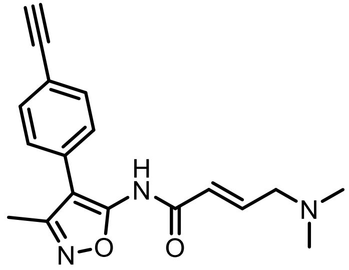

Current Data Parameters
NAME at601560412
EXPNO 1
PROCNO 1

F2 - Acquisition Parameters
Date_ 20201204
Time 16.07
INSTRUM avc500
PROBHD 5 mm CPDUL 13C
PULPROG zg30
TD 65536
SOLVENT CDCl3
NS 16
DS 4
SWH 10330.578 Hz
FIDRES 0.157632 Hz
AQ 3.1719425 sec
RG 4
DW 48.400 usec
DE 10.00 usec
TE 298.0 K
D1 1.00000000 sec
TD0 1

======== CHANNEL f1 ========
SFO1 500.3030896 MHz
NUC1 1H
P1 22.00 usec
PLW1 7.99830008 W

F2 - Processing parameters
SI 65536
SF 500.3000132 MHz
WDW EM
SSB 0
LB 0.30 Hz
GB 0
PC 1.00

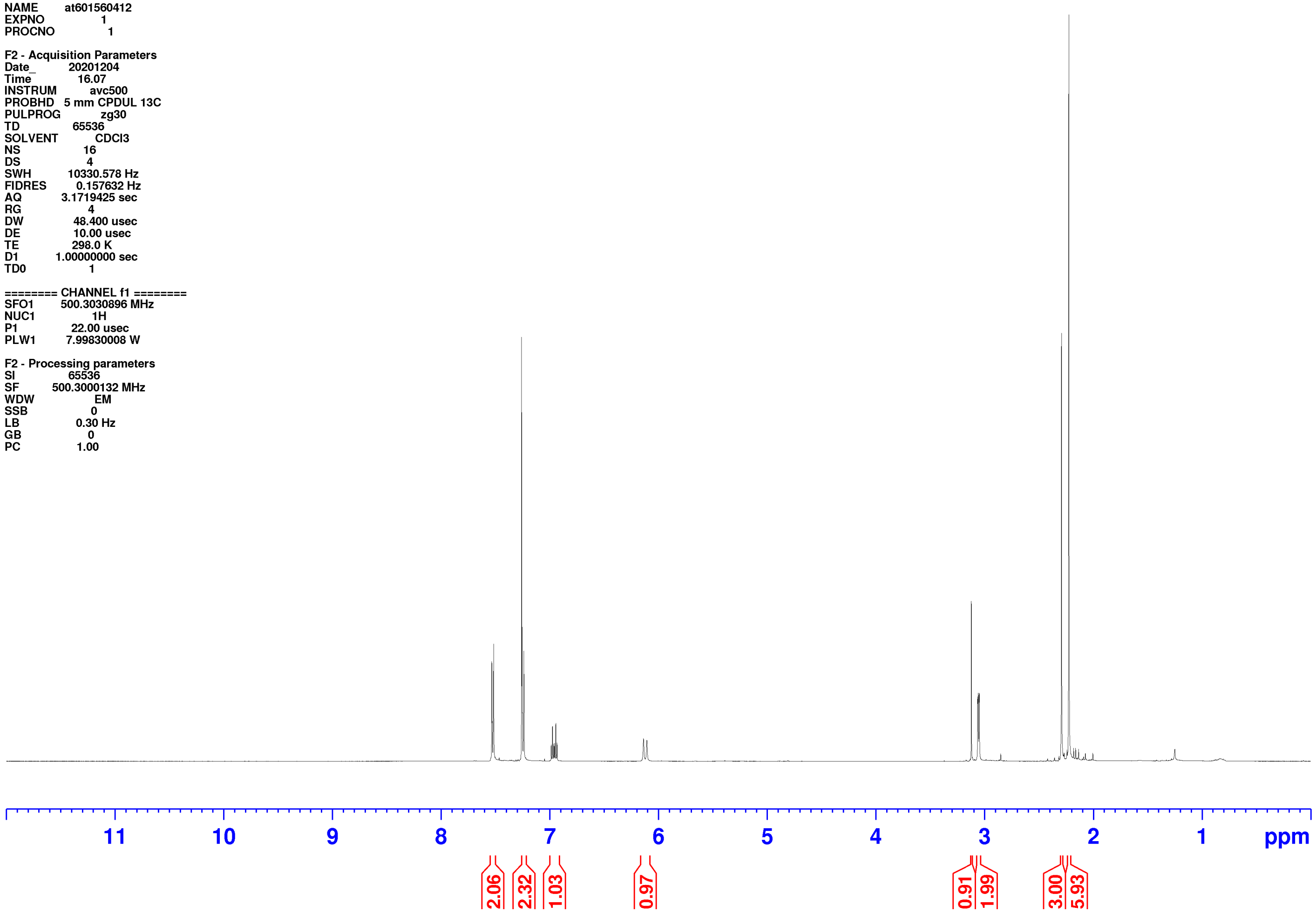

(*E*)-4-(Dimethylamino)-*N*-[4-(4-ethynylphenyl)-3-methylisoxazol-5-yl]but-2-enamide (28)

```
Current Data Parameters
NAME          at601560412
EXPNO                   4
PROCNO                  1

F2 - Acquisition Parameters
Date_            20201204
Time                18.14
INSTRUM            avc500
PROBHD   5 mm CPDUL 13C
PULPROG           zgpg30
TD                  65536
SOLVENT             CDCl3
NS                   2048
DS                      2
SWH           31250.000 Hz
FIDRES         0.476837 Hz
AQ            1.0485760 sec
RG                    912
DW               16.000 usec
DE                18.00 usec
TE                298.0 K
D1           2.00000000 sec
D11          0.03000000 sec
TD0                     1

======== CHANNEL f1 ========
SFO1        125.8131152 MHz
NUC1                  13C
P1                  10.00 usec
PLW1        20.18400002 W

======== CHANNEL f2 ========
SFO2        500.3020012 MHz
NUC2                   1H
CPDPRG[2          waltz16
PCPD2               80.00 usec
PLW2         7.99830008 W
PLW12        0.60487002 W
PLW13        0.38712001 W

F2 - Processing parameters
SI                  32768
SF          125.8005197 MHz
WDW                    EM
SSB                     0
LB                   1.00 Hz
GB                      0
PC                   1.40
```

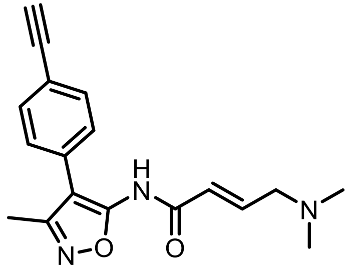

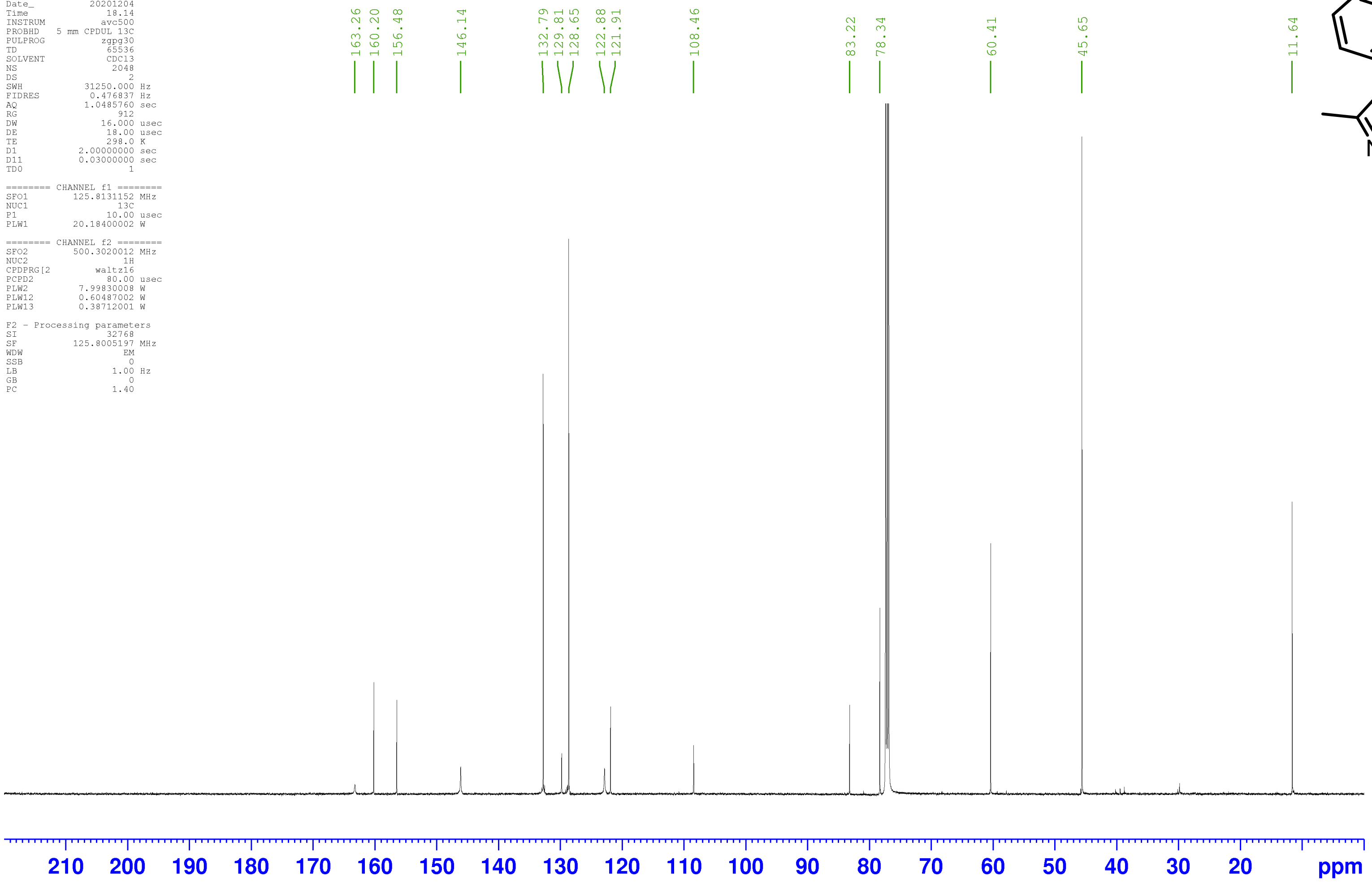

2-(4-Prop-2-ynoxyphenyl)acetonitrile (37)

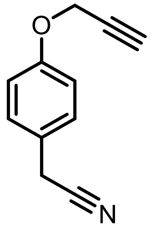

Current Data Parameters
NAME at635792708_2
EXPNO 1
PROCNO 1

F2 - Acquisition Parameters
Date_ 20210901
Time 20.35 h
INSTRUM Avance
PROBHD Z159656_0020 (
PULPROG zg30
TD 65536
SOLVENT CDCl3
NS 16
DS 2
SWH 11904.762 Hz
FIDRES 0.363304 Hz
AQ 2.7525120 sec
RG 36.4882
DW 42.000 usec
DE 22.00 usec
TE 298.0 K
D1 1.00000000 sec
TD0 1
SFO1 600.4230021 MHz
NUC1 1H
P0 4.00 usec
P1 12.00 usec
PLW1 13.51200008 W

F2 - Processing parameters
SI 65536
SF 600.4200719 MHz
WDW EM
SSB 0
LB 0.30 Hz
GB 0
PC 1.00

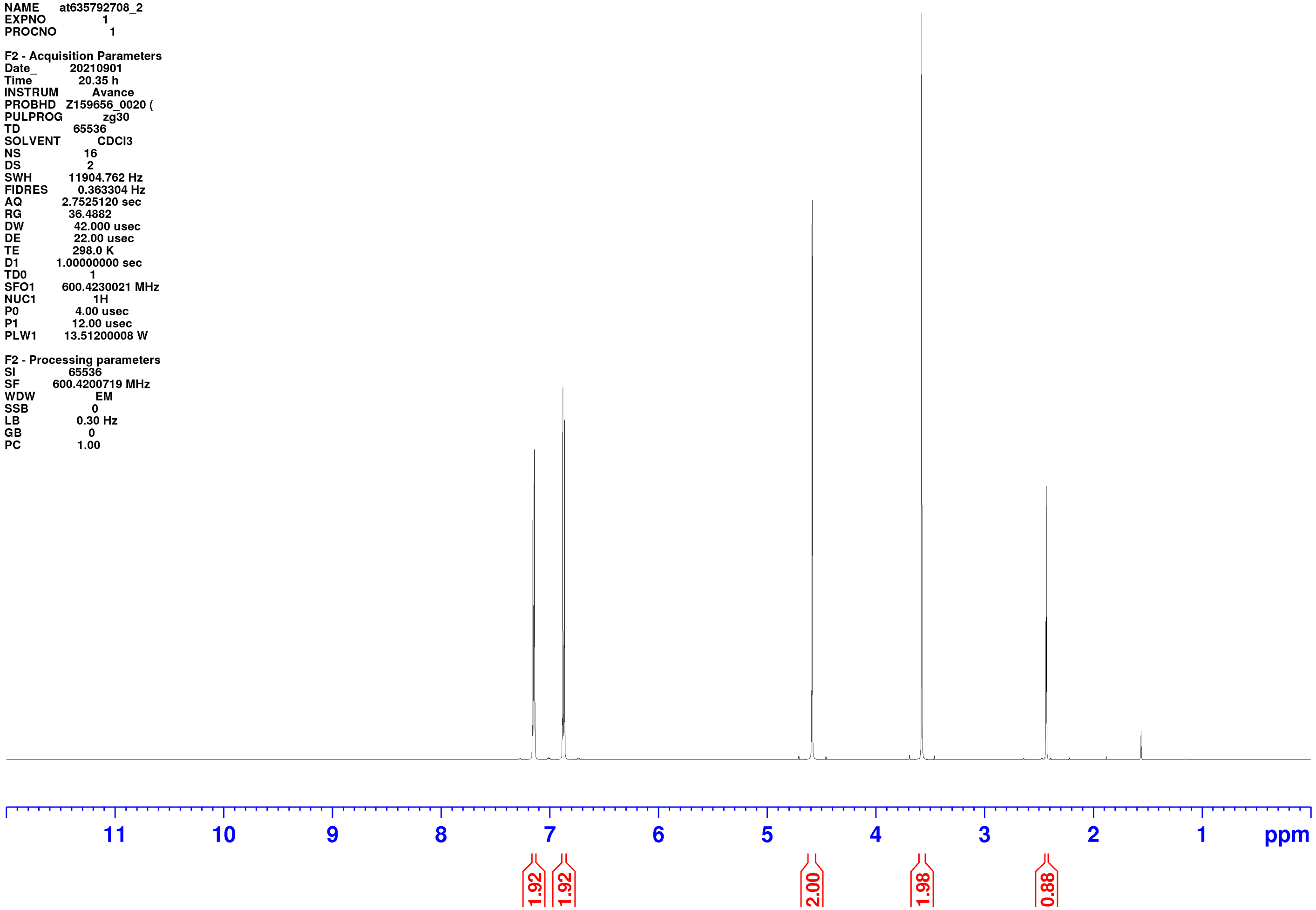

2-(4-Prop-2-ynoxyphenyl)acetonitrile (37)

```
Current Data Parameters
NAME       at635792708_2
EXPNO                  5
PROCNO                 1

F2 - Acquisition Parameters
Date_           20210901
Time               21.28 h
INSTRUM           Avance
PROBHD   Z159656_0020 (
PULPROG          zgpg30
TD                 65536
SOLVENT            CDCl3
NS                   512
DS                     4
SWH            35714.285 Hz
FIDRES          1.089913 Hz
AQ             0.9175040 sec
RG                   101
DW                14.000 usec
DE                 18.00 usec
TE                 298.0 K
D1            2.00000000 sec
D11           0.03000000 sec
TD0                    1
SFO1         150.9908267 MHz
NUC1                 13C
P0                  3.33 usec
P1                 10.00 usec
PLW1         41.91400146 W
SFO2         600.4224017 MHz
NUC2                  1H
CPDPRG[2         waltz16
PCPD2              80.00 usec
PLW2         13.51200008 W
PLW12         0.30124050 W
PLW13         0.15098180 W

F2 - Processing parameters
SI                 65536
SF           150.9757256 MHz
WDW                   EM
SSB                    0
LB                  1.00 Hz
GB                     0
PC                  1.40
```

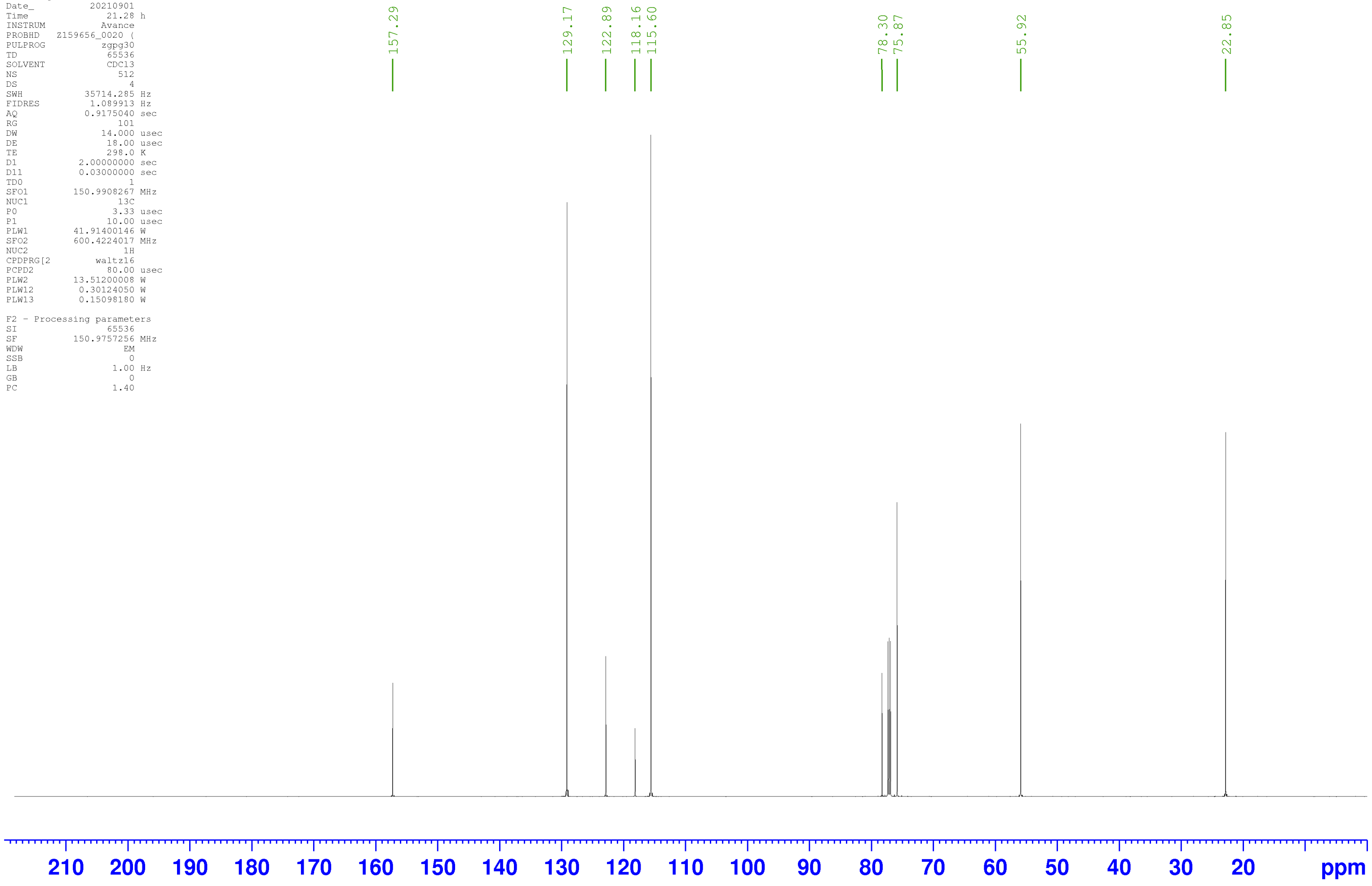

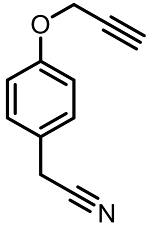

3-Oxo-2-(4-prop-2-ynoxyphenyl)butanenitrile (38)

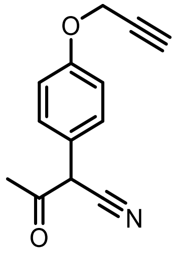

Current Data Parameters
NAME at635592408
EXPNO 1
PROCNO 1

F2 - Acquisition Parameters
Date_ 20210825
Time 5.45 h
INSTRUM Avance
PROBHD Z159656_0020 (
PULPROG zg30
TD 65536
SOLVENT CDCl3
NS 16
DS 2
SWH 11904.762 Hz
FIDRES 0.363304 Hz
AQ 2.7525120 sec
RG 45.9973
DW 42.000 usec
DE 22.00 usec
TE 298.0 K
D1 1.00000000 sec
TD0 1
SFO1 600.4230021 MHz
NUC1 1H
P0 4.00 usec
P1 12.00 usec
PLW1 13.51200008 W

F2 - Processing parameters
SI 65536
SF 600.4200142 MHz
WDW EM
SSB 0
LB 0.30 Hz
GB 0
PC 1.00

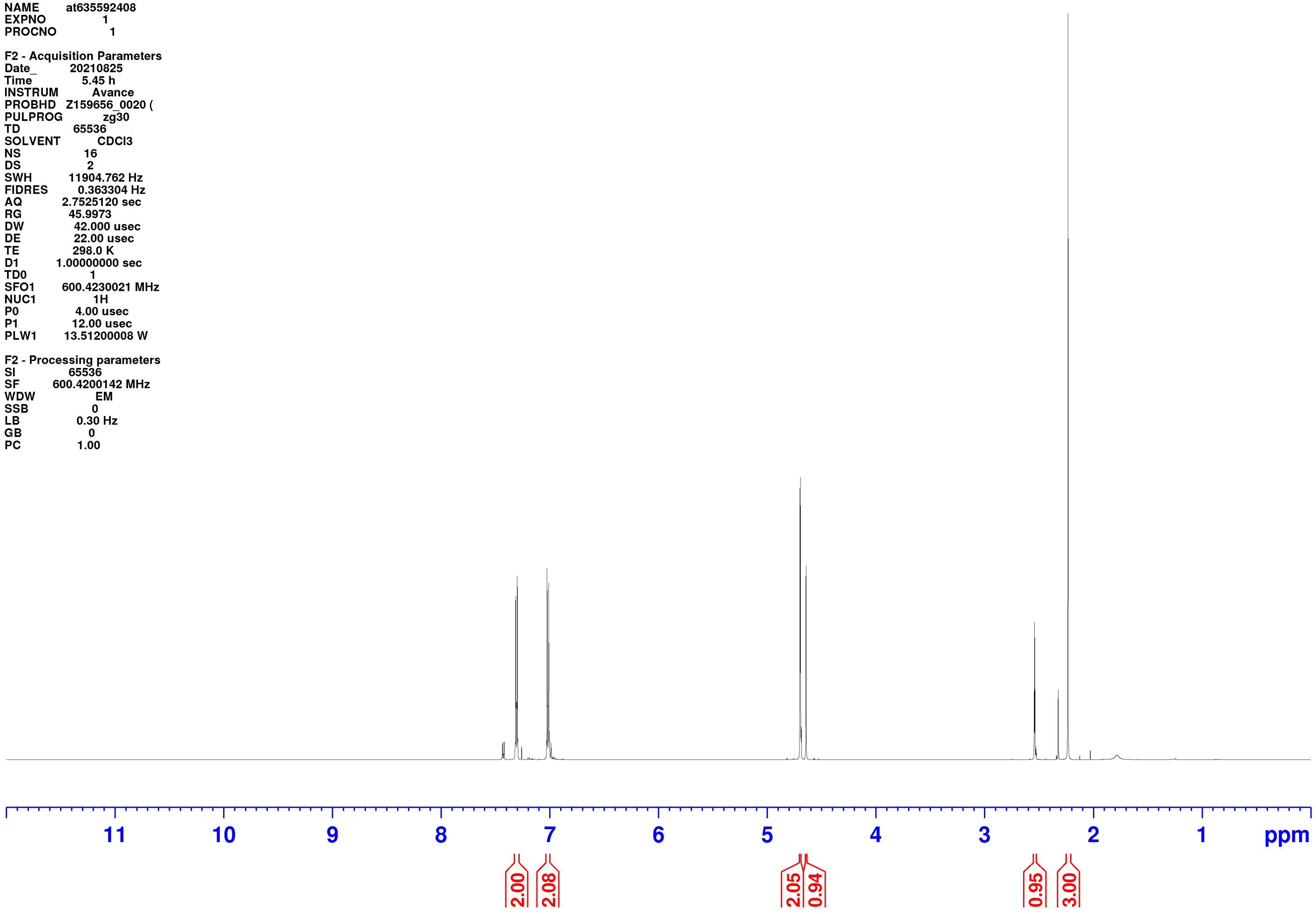

3-Oxo-2-(4-prop-2-ynoxyphenyl)butanenitrile (38)

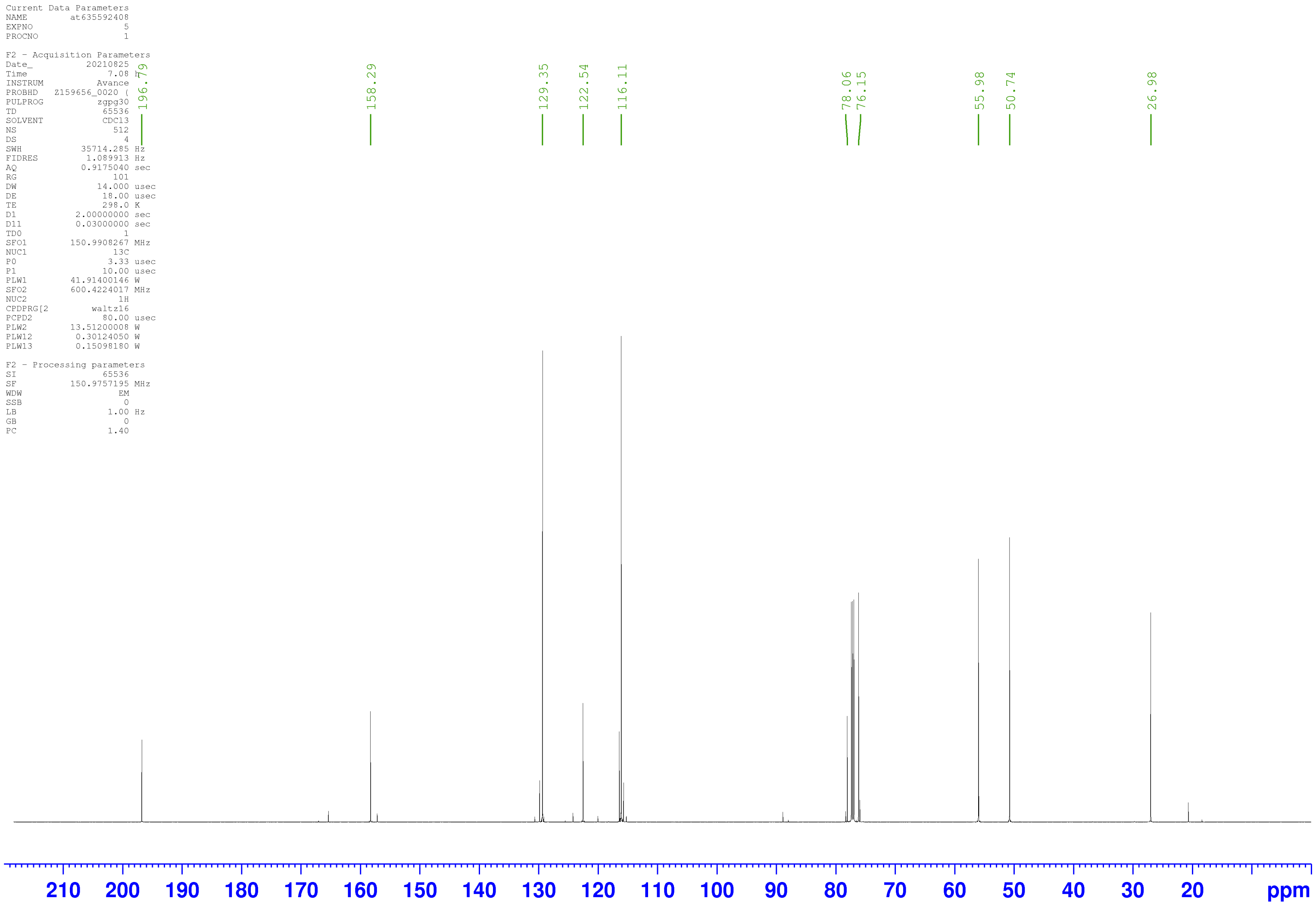

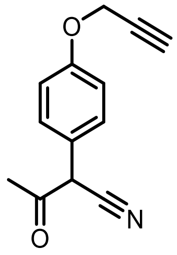

3-Methyl-4-(4-prop-2-ynoxyphenyl)-isoxazol-5-amine (39)

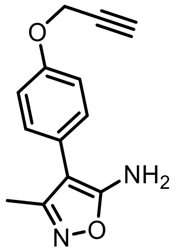

Current Data Parameters
NAME at604391301
EXPNO 1
PROCNO 1

F2 - Acquisition Parameters
Date_ 20210115
Time 1.33
INSTRUM avc500
PROBHD 5 mm CPDUL 13C
PULPROG zg30
TD 65536
SOLVENT CDCl3
NS 16
DS 4
SWH 10330.578 Hz
FIDRES 0.157632 Hz
AQ 3.1719425 sec
RG 4
DW 48.400 usec
DE 10.00 usec
TE 298.0 K
D1 1.00000000 sec
TD0 1

======== CHANNEL f1 ========
SFO1 500.3030896 MHz
NUC1 1H
P1 22.00 usec
PLW1 7.99830008 W

F2 - Processing parameters
SI 65536
SF 500.3000132 MHz
WDW EM
SSB 0
LB 0.30 Hz
GB 0
PC 1.00

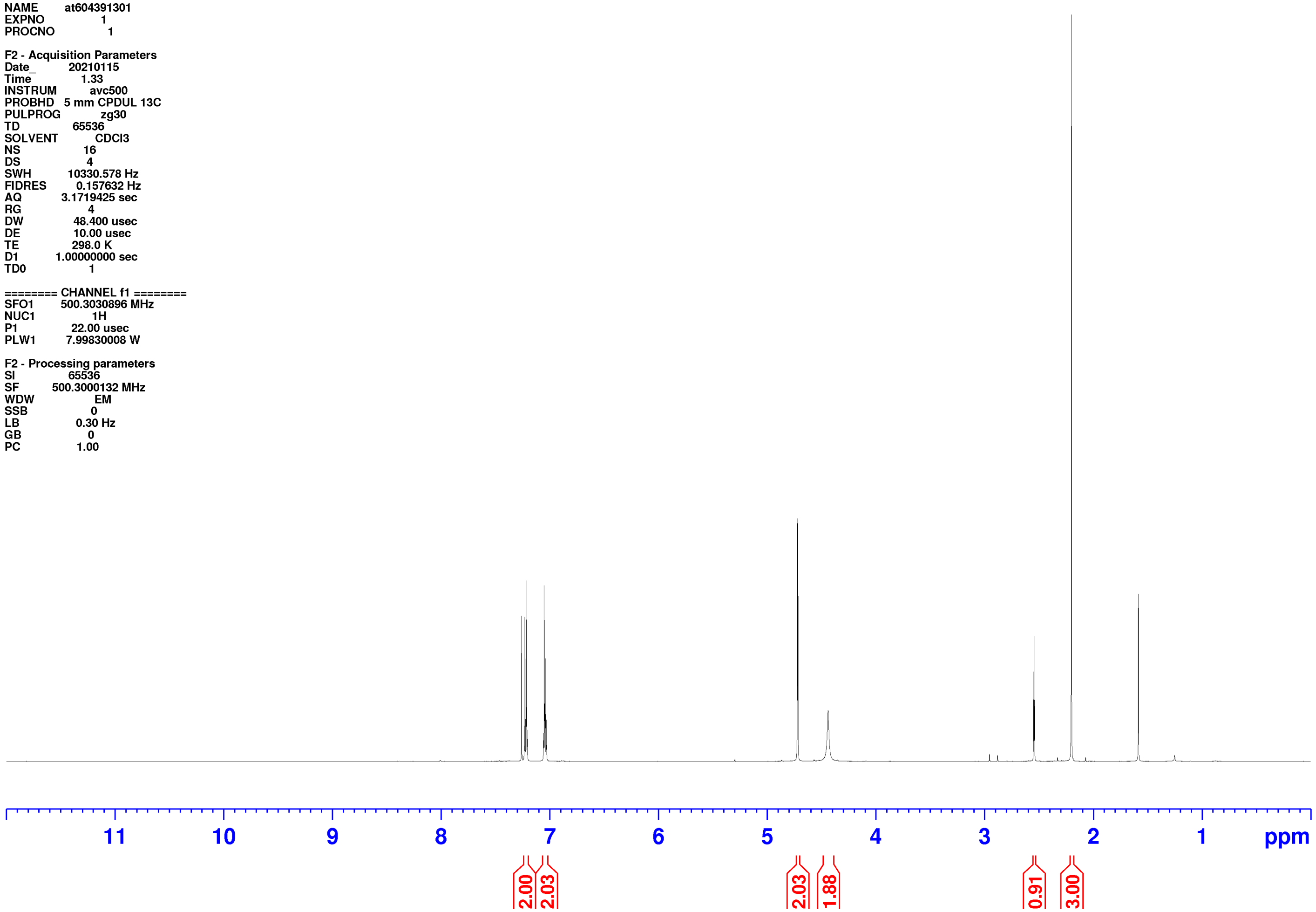

3-Methyl-4-(4-prop-2-ynoxyphenyl)-isoxazol-5-amine (39)

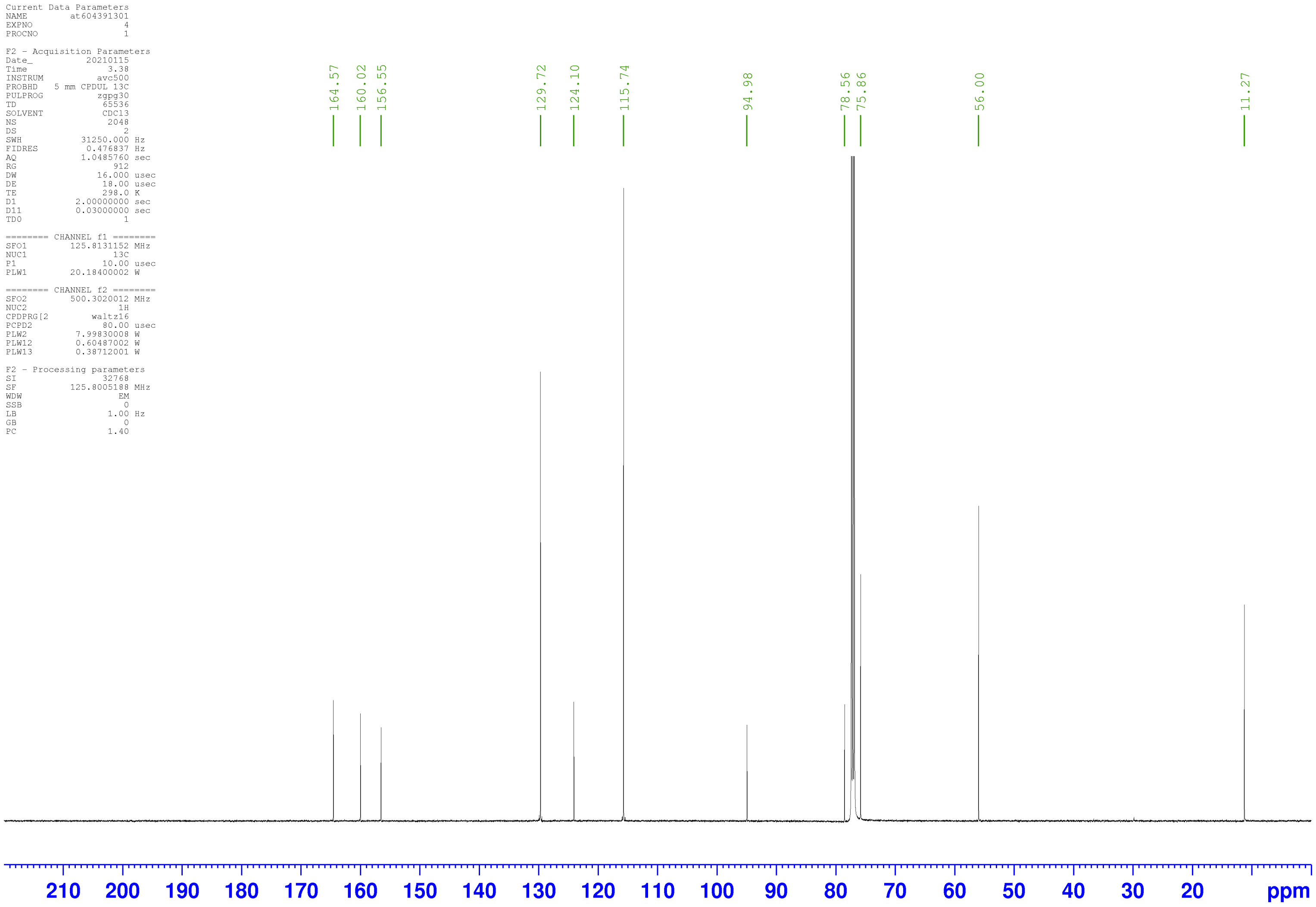

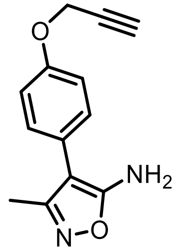

(*E*)-4-Bromo/Chloro-*N*-[3-methyl-4-(4-prop-2-ynoxyphenyl)-isoxazol-5-yl]but-2-enamide (71)

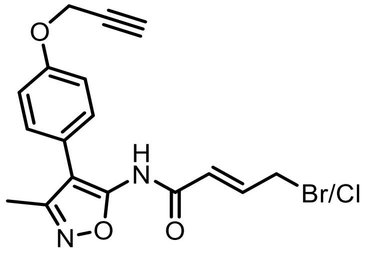

Current Data Parameters
NAME Sep24-2020-34-AMTD63
EXPNO 1
PROCNO 1

F2 - Acquisition Parameters
Date_ 20200924
Time 15.30 h
INSTRUM avg400
PROBHD Z108618_0816 (
PULPROG zg60
TD 65536
SOLVENT CDCl3
NS 16
DS 2
SWH 8012.820 Hz
FIDRES 0.244532 Hz
AQ 4.0894465 sec
RG 58.47
DW 62.400 usec
DE 6.50 usec
TE 297.7 K
D1 1.00000000 sec
TD0 1
SFO1 400.2024012 MHz
NUC1 1H
P1 14.00 usec
PLW1 14.00000000 W

F2 - Processing parameters
SI 32768
SF 400.2000096 MHz
WDW EM
SSB 0
LB 0.30 Hz
GB 0
PC 1.00

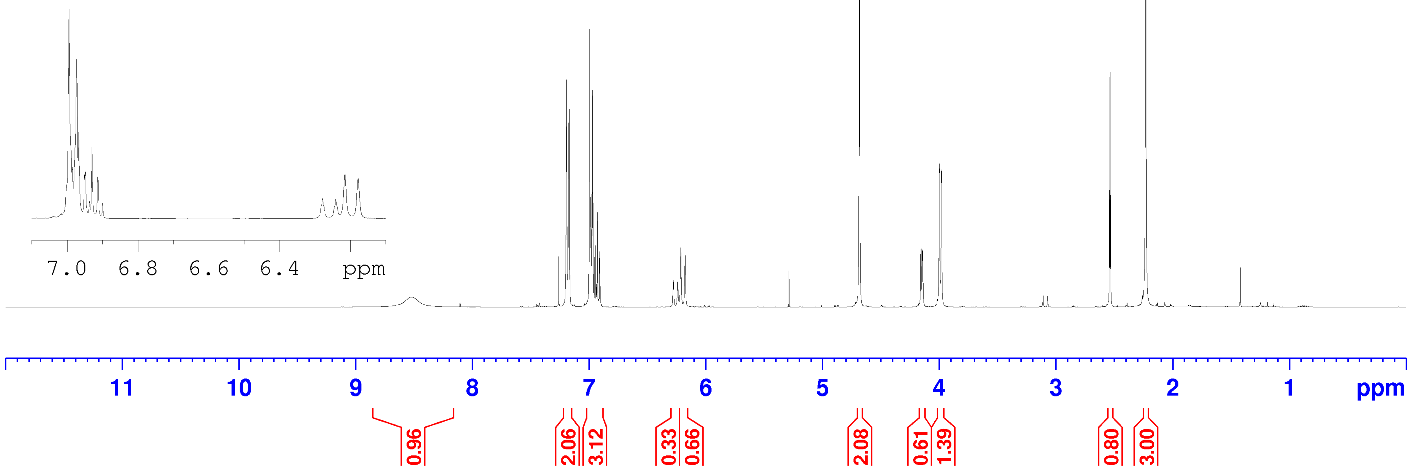

(*E*)-4-(Dimethylamino)-*N*-[3-methyl-4-(4-prop-2-ynoxyphenyl)-isoxazol-5-yl]but-2-enamide (29)

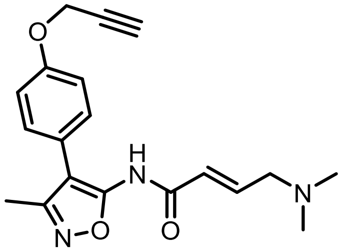

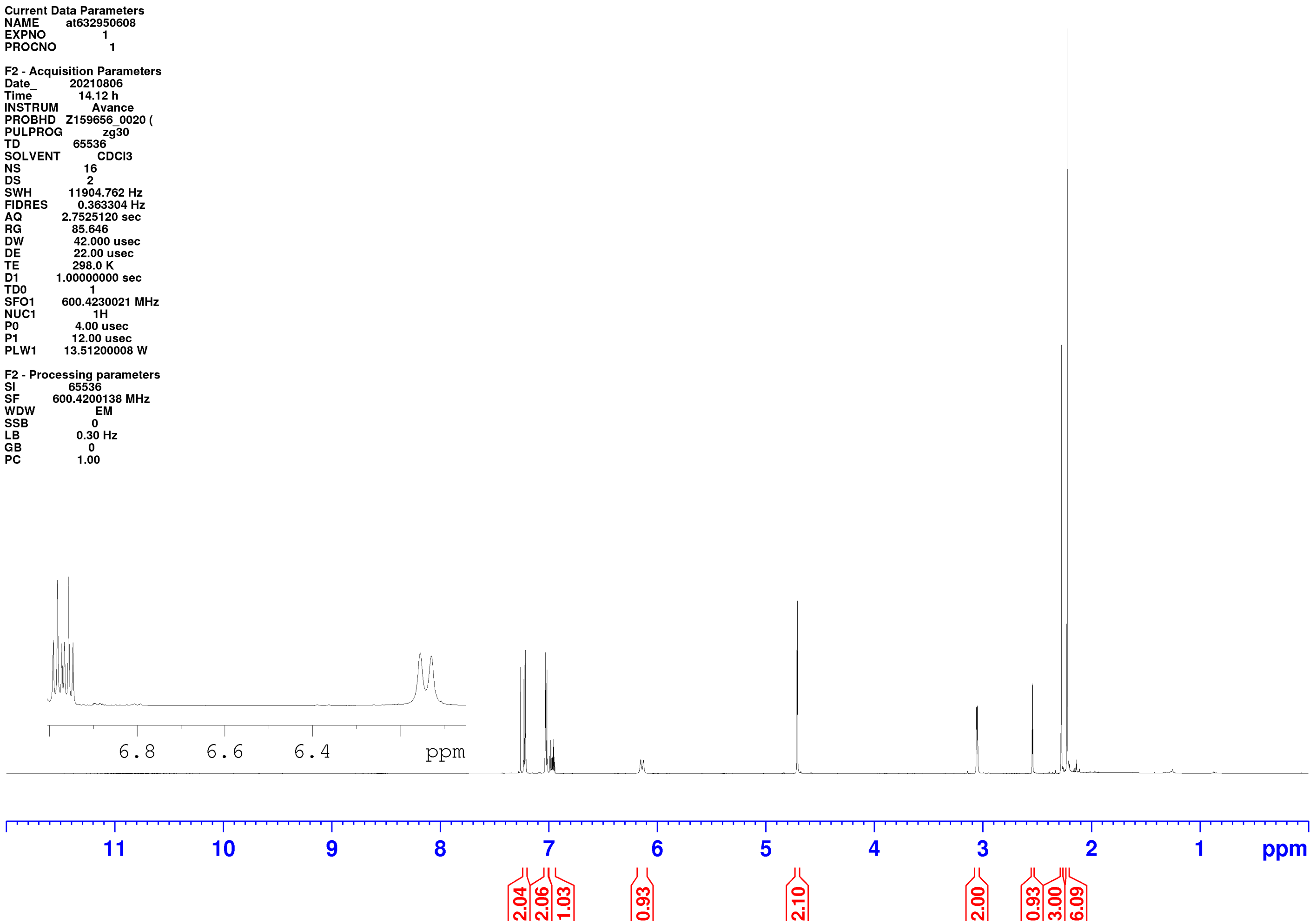

(*E*)-4-(Dimethylamino)-*N*-[3-methyl-4-(4-prop-2-ynoxyphenyl)-isoxazol-5-yl]but-2-enamide (29)

```
Current Data Parameters
NAME          at632950608
EXPNO                   5
PROCNO                  1

F2 - Acquisition Parameters
Date_            20210806
Time                15.46 h
INSTRUM            Avance
PROBHD   Z159656_0020 (
PULPROG           zgpg30
TD                 65536
SOLVENT            CDCl3
NS                  1024
DS                     4
SWH            35714.285 Hz
FIDRES          1.089913 Hz
AQ             0.9175040 sec
RG                   101
DW                14.000 usec
DE                 18.00 usec
TE                 298.0 K
D1            2.00000000 sec
D11           0.03000000 sec
TD0                    1
SFO1         150.9908267 MHz
NUC1                 13C
P0                  3.33 usec
P1                 10.00 usec
PLW1         41.91400146 W
SFO2         600.4224017 MHz
NUC2                  1H
CPDPRG[2         waltz16
PCPD2              80.00 usec
PLW2         13.51200008 W
PLW12         0.30124050 W
PLW13         0.15098180 W

F2 - Processing parameters
SI                 65536
SF           150.9757087 MHz
WDW                   EM
SSB                    0
LB                  1.00 Hz
GB                     0
PC                  1.40
```

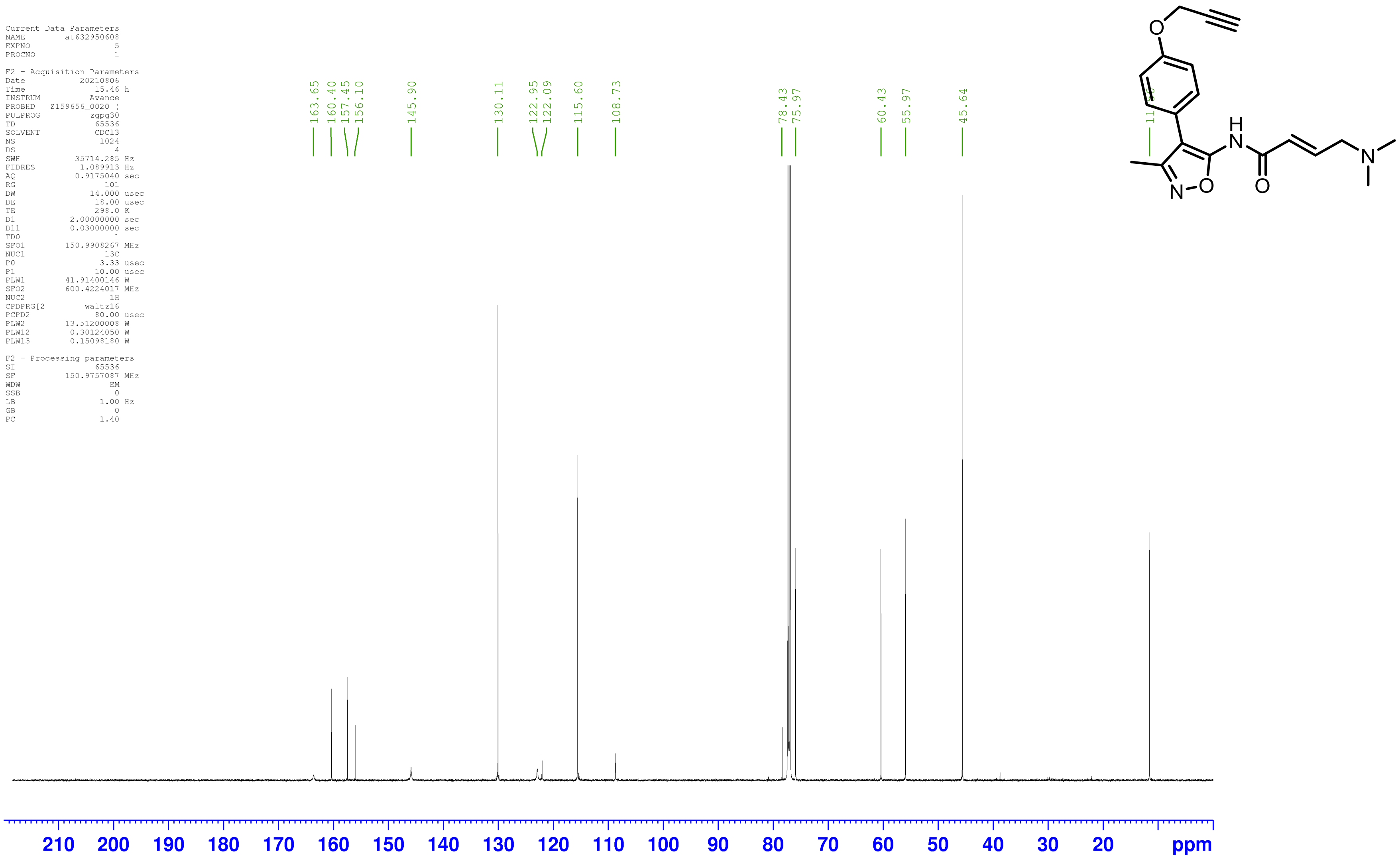

N-[3-Methyl-4-(4-prop-2-ynoxyphenyl)-isoxazol-5-yl]prop-2-enamide (30)

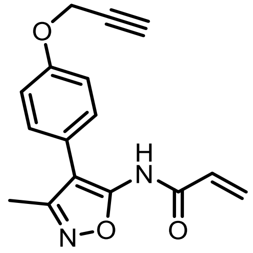

Current Data Parameters
NAME at633360908
EXPNO 1
PROCNO 1

F2 - Acquisition Parameters
Date_ 20210809
Time 22.48 h
INSTRUM Avance
PROBHD Z159656_0020 (
PULPROG zg30
TD 65536
SOLVENT CDCl3
NS 16
DS 2
SWH 11904.762 Hz
FIDRES 0.363304 Hz
AQ 2.7525120 sec
RG 53.5624
DW 42.000 usec
DE 22.00 usec
TE 298.0 K
D1 1.00000000 sec
TD0 1
SFO1 600.4230021 MHz
NUC1 1H
P0 4.00 usec
P1 12.00 usec
PLW1 13.51200008 W

F2 - Processing parameters
SI 65536
SF 600.4200146 MHz
WDW EM
SSB 0
LB 0.30 Hz
GB 0
PC 1.00

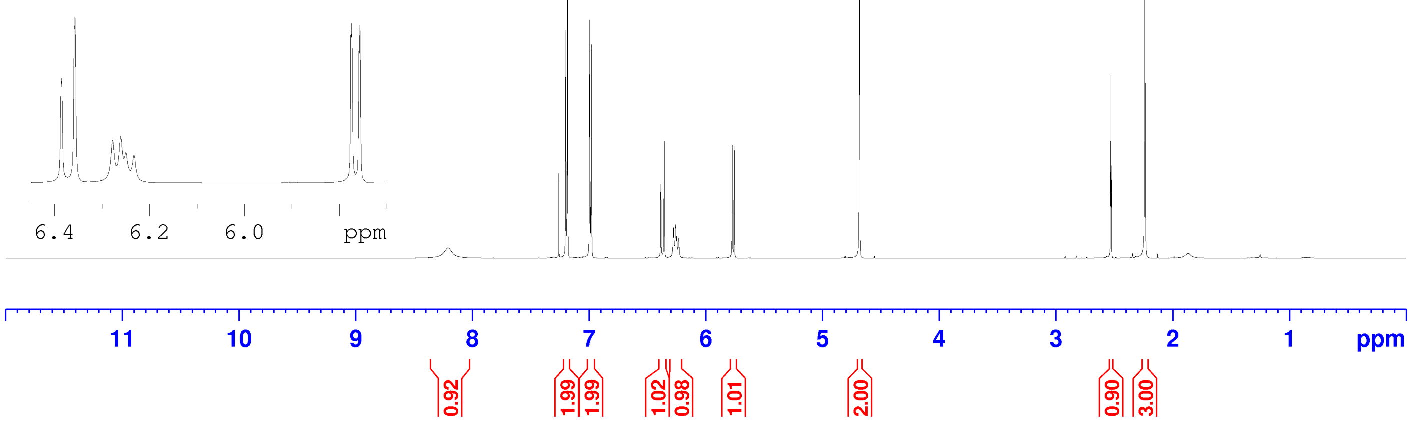

N-[3-Methyl-4-(4-prop-2-ynoxyphenyl)-isoxazol-5-yl]prop-2-enamide (30)

```
Current Data Parameters
NAME          at633360908
EXPNO                   5
PROCNO                  1

F2 - Acquisition Parameters
Date_            20210810
Time                 0.21 h
INSTRUM            Avance
PROBHD   Z159656_0020 (
PULPROG           zgpg30
TD                  65536
SOLVENT             CDCl3
NS                   1024
DS                      4
SWH             35714.285 Hz
FIDRES           1.089913 Hz
AQ              0.9175040 sec
RG                    101
DW                 14.000 usec
DE                  18.00 usec
TE                  298.0 K
D1             2.00000000 sec
D11            0.03000000 sec
TD0                     1
SFO1          150.9908267 MHz
NUC1                  13C
P0                   3.33 usec
P1                  10.00 usec
PLW1          41.91400146 W
SFO2          600.4224017 MHz
NUC2                   1H
CPDPRG[2          waltz16
PCPD2               80.00 usec
PLW2          13.51200008 W
PLW12          0.30124050 W
PLW13          0.15098180 W

F2 - Processing parameters
SI                  65536
SF            150.9757148 MHz
WDW                    EM
SSB                     0
LB                   1.00 Hz
GB                      0
PC                   1.40
```

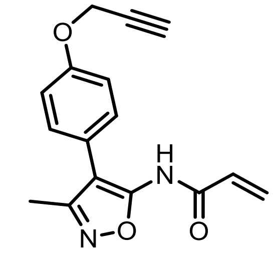

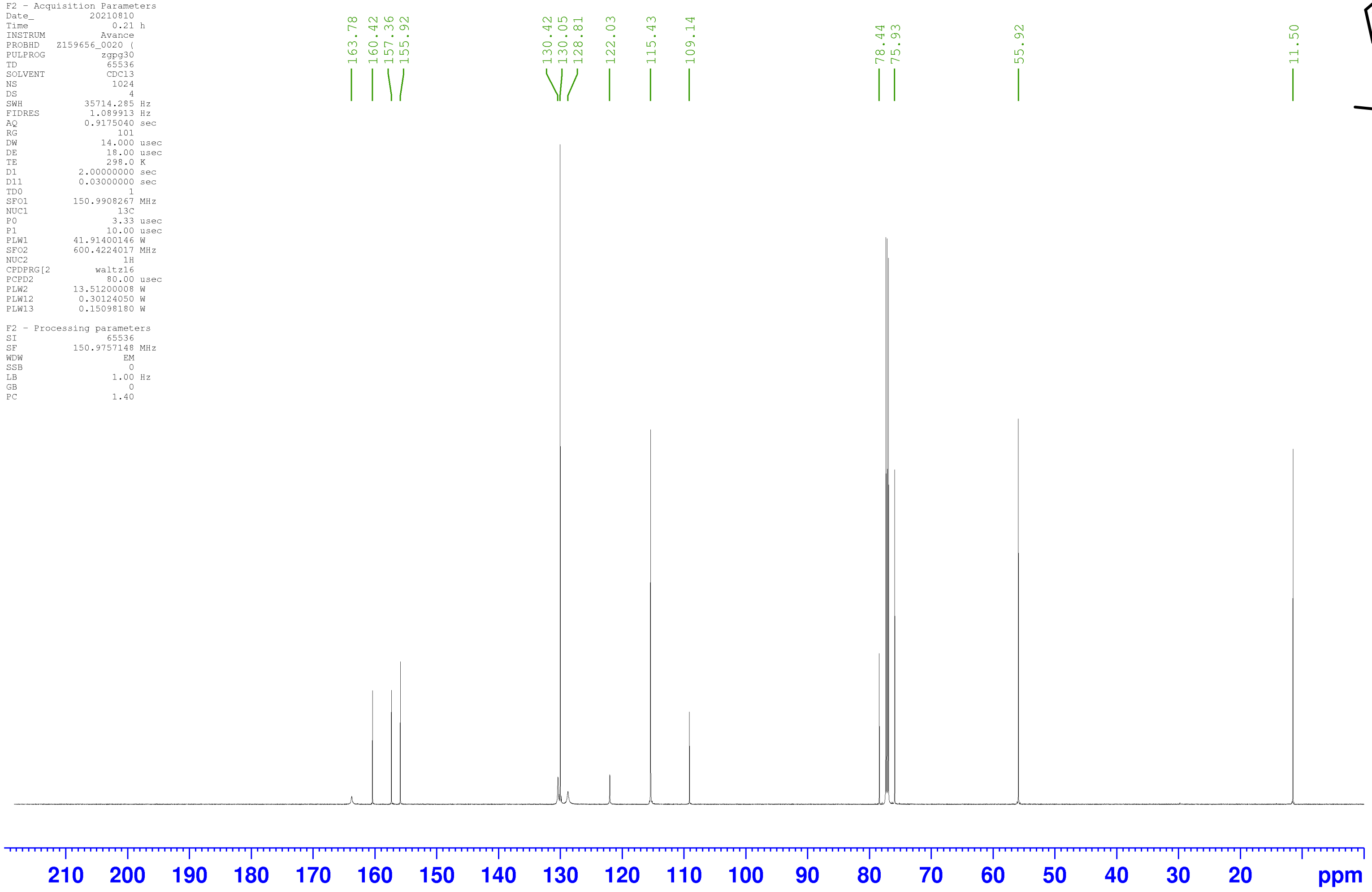

(*E*)-4-Bromo/Chloro-*N*-(3-methoxy-4-(2-oxopyrrolidin-1-yl)phenyl)but-2-enamide (44)

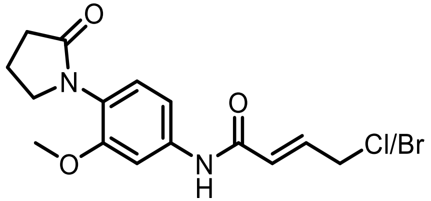

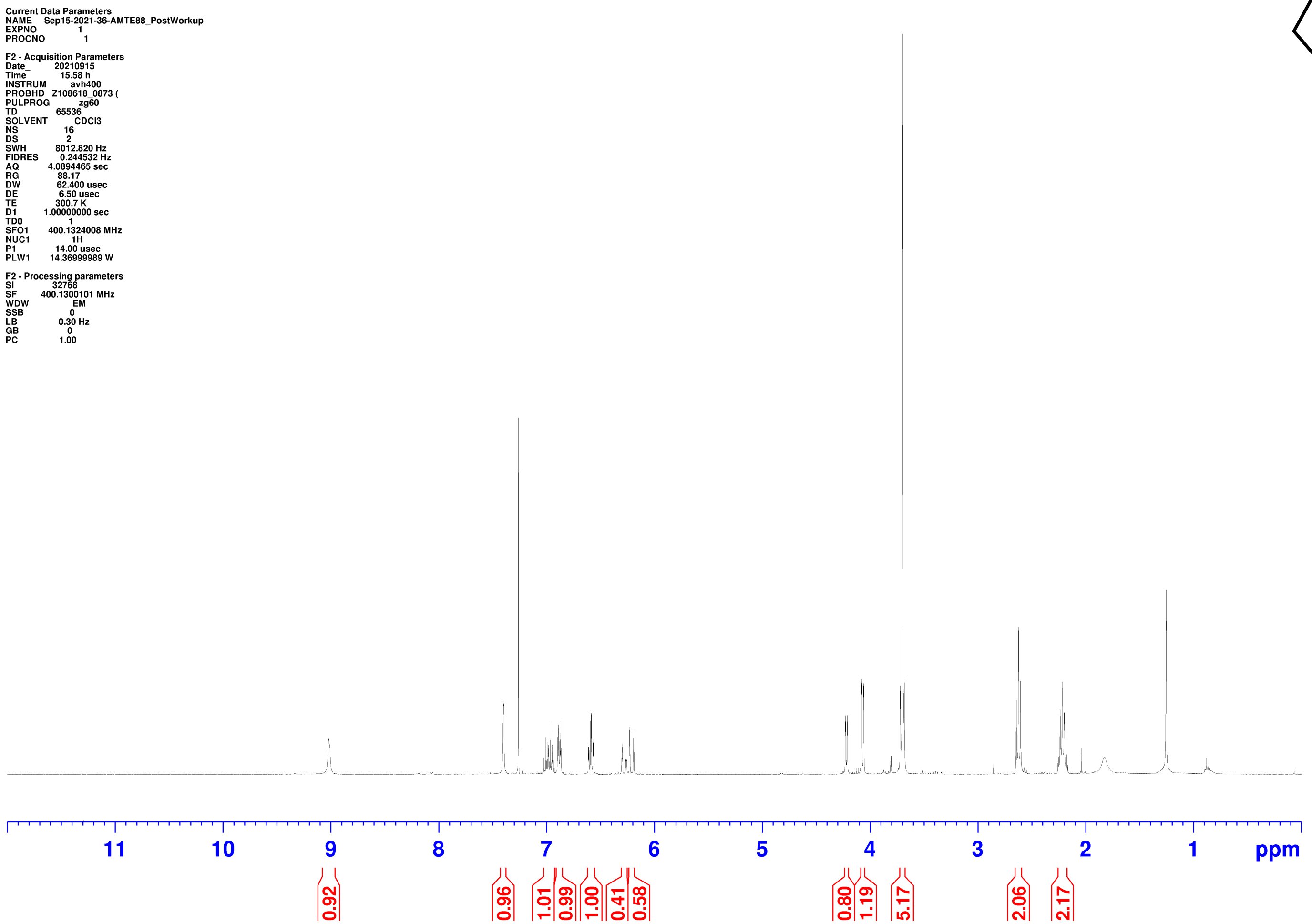

(*E*)-*N*-[3-Methoxy-4-(2-oxopyrrolidin-1-yl)phenyl]-4-[methyl(prop-2-ynyl)amino]but-2-enamide (41)

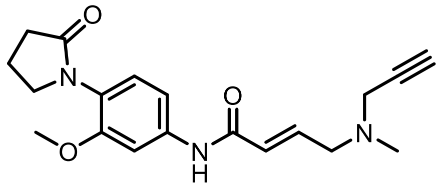

Current Data Parameters
NAME at638552009
EXPNO 1
PROCNO 1

F2 - Acquisition Parameters
Date_ 20210920
Time 14.23 h
INSTRUM Avance
PROBHD Z159656_0020 (
PULPROG zg30
TD 65536
SOLVENT CDCl3
NS 16
DS 2
SWH 11904.762 Hz
FIDRES 0.363304 Hz
AQ 2.7525120 sec
RG 87.0054
DW 42.000 usec
DE 22.00 usec
TE 298.0 K
D1 1.00000000 sec
TD0 1
SFO1 600.4230021 MHz
NUC1 1H
P0 4.00 usec
P1 12.00 usec
PLW1 13.51200008 W

F2 - Processing parameters
SI 65536
SF 600.4200151 MHz
WDW EM
SSB 0
LB 0.30 Hz
GB 0
PC 1.00

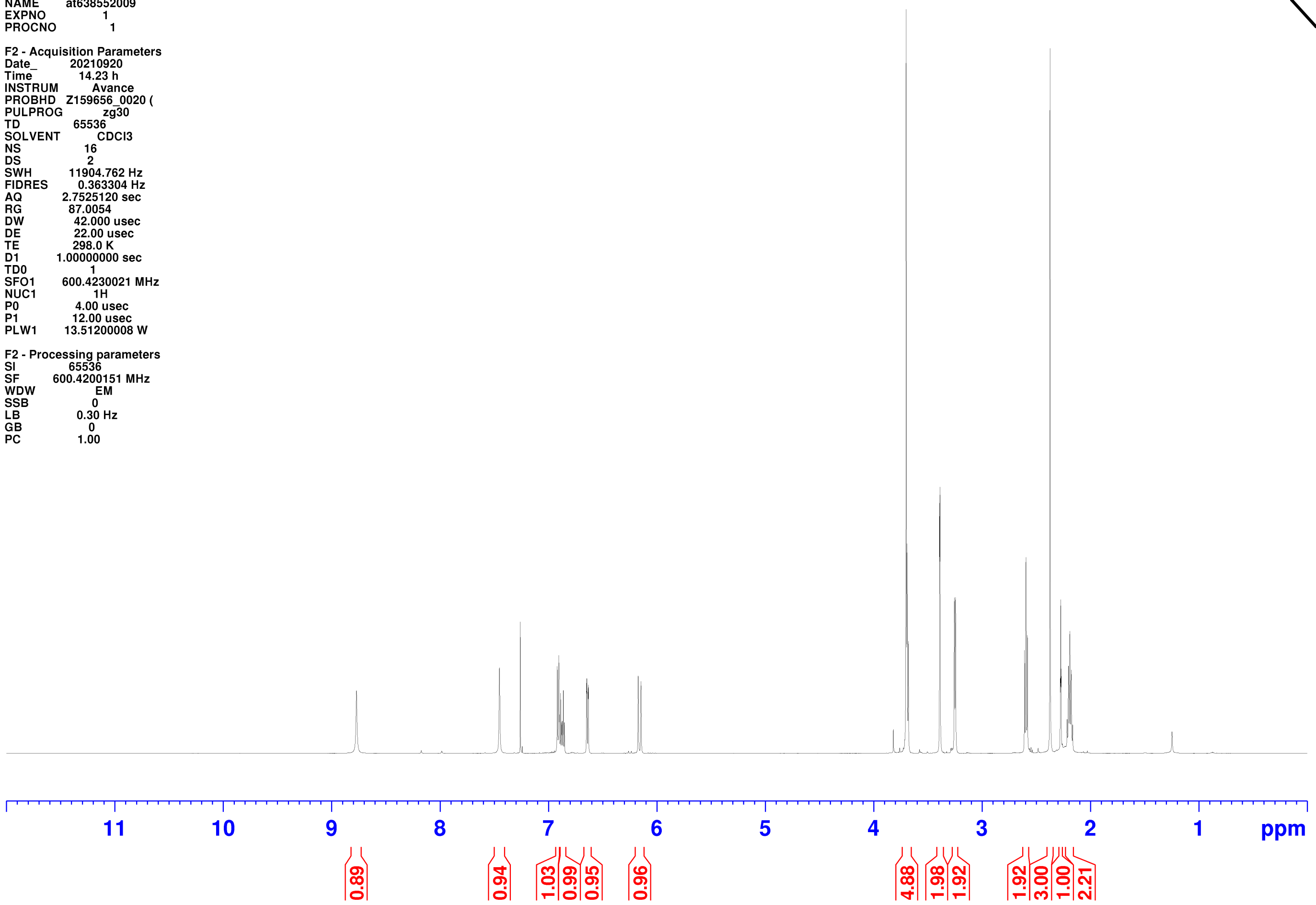

(*E*)-*N*-[3-Methoxy-4-(2-oxopyrrolidin-1-yl)phenyl]-4-[methyl(prop-2-ynyl)amino]but-2-enamide (41)

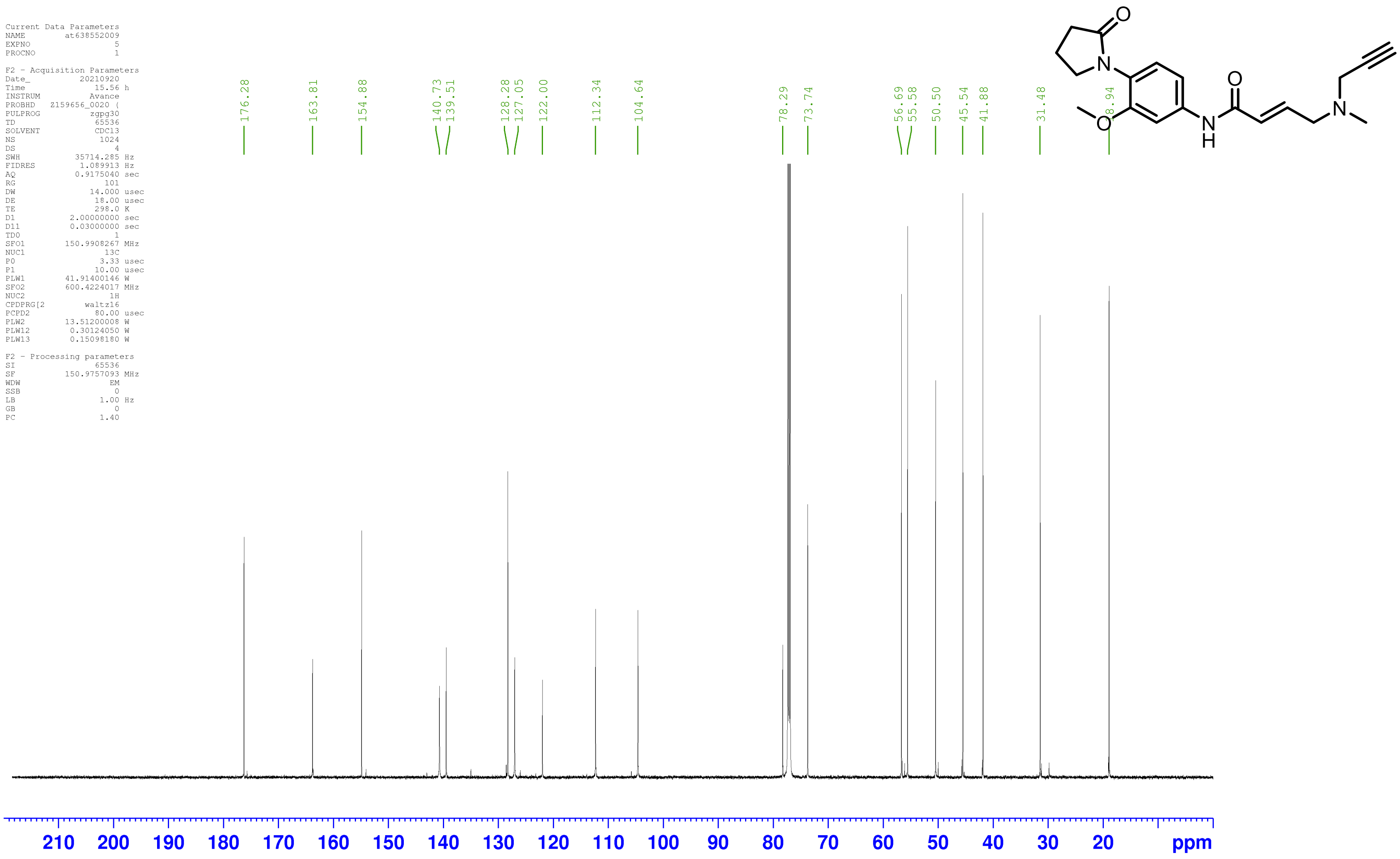

1-(4-Amino-2-hydroxyphenyl)pyrrolidin-2-one (45)

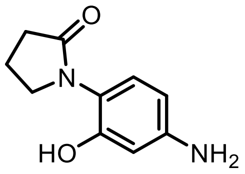

Current Data Parameters
NAME ms645782511_MSEB41_B70_B88
EXPNO 1
PROCNO 1

F2 - Acquisition Parameters
Date_ 20211126
Time 4.45 h
INSTRUM Avance
PROBHD Z159656_0020 (
PULPROG zg30
TD 65536
SOLVENT DMSO
NS 16
DS 2
SWH 11904.762 Hz
FIDRES 0.363304 Hz
AQ 2.7525120 sec
RG 77.86
DW 42.000 usec
DE 22.00 usec
TE 298.0 K
D1 1.00000000 sec
TD0 1
SFO1 600.4230021 MHz
NUC1 1H
P0 4.00 usec
P1 12.00 usec
PLW1 13.51200008 W

F2 - Processing parameters
SI 65536
SF 600.4200046 MHz
WDW EM
SSB 0
LB 0.30 Hz
GB 0
PC 1.00

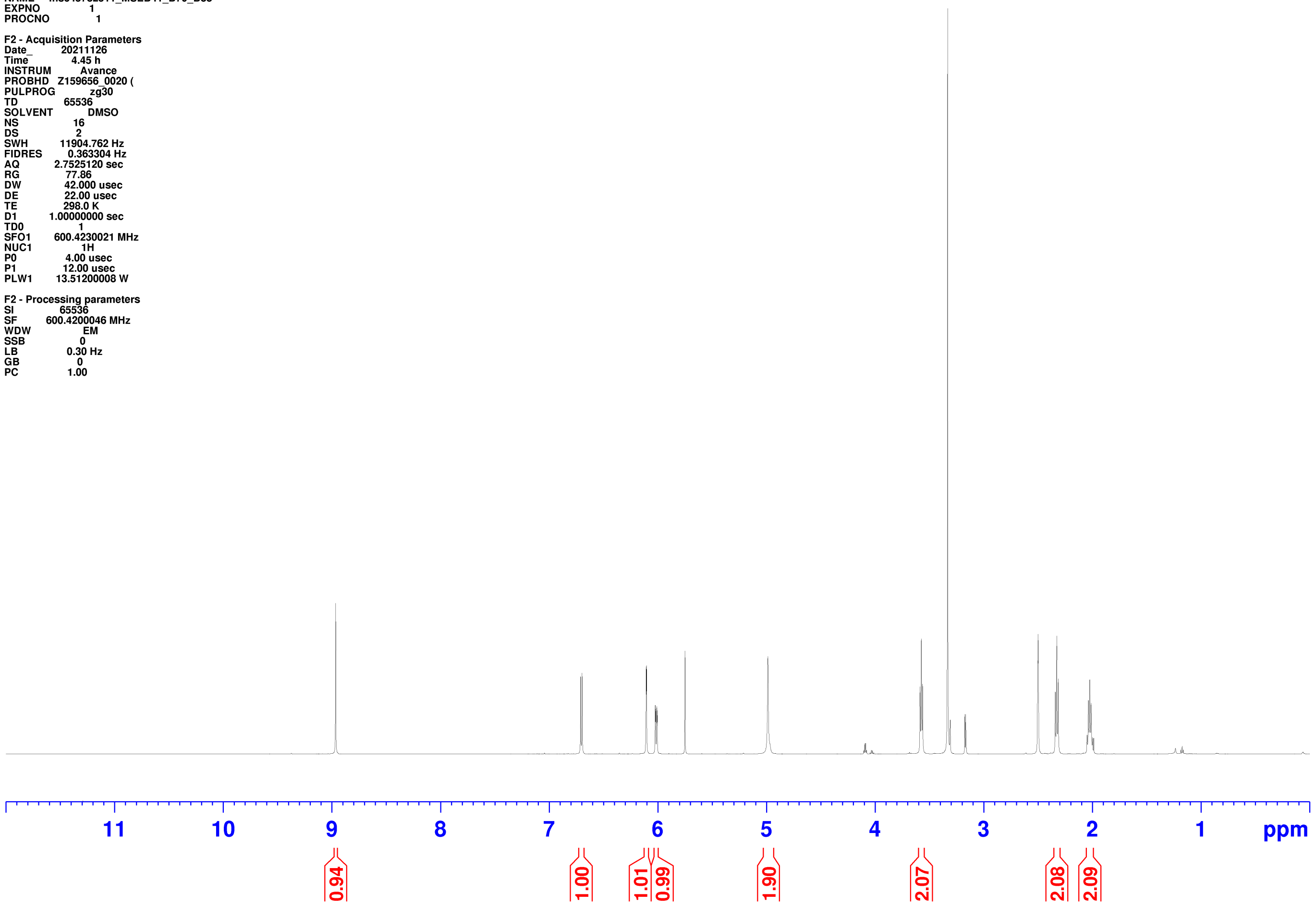

1-(4-Amino-2-hydroxyphenyl)pyrrolidin-2-one (45)

```
Current Data Parameters
NAME     ms645782511_MSEB41_B70_B88
EXPNO                  5
PROCNO                 1

F2 - Acquisition Parameters
Date_           20211126
Time                5.53 h
INSTRUM           Avance
PROBHD   Z159656_0020 (
PULPROG          zgpg30
TD                65536
SOLVENT            DMSO
NS                  512
DS                    4
SWH           35714.285 Hz
FIDRES         1.089913 Hz
AQ            0.9175040 sec
RG                  101
DW               14.000 usec
DE                18.00 usec
TE                298.0 K
D1           2.00000000 sec
D11          0.03000000 sec
TD0                   1
SFO1        150.9908267 MHz
NUC1                13C
P0                 3.33 usec
P1                10.00 usec
PLW1        41.91400146 W
SFO2        600.4224017 MHz
NUC2                 1H
CPDPRG[2        waltz16
PCPD2             80.00 usec
PLW2        13.51200008 W
PLW12        0.30124050 W
PLW13        0.15098180 W

F2 - Processing parameters
SI                65536
SF          150.9757291 MHz
WDW                  EM
SSB                   0
LB                 1.00 Hz
GB                    0
PC                 1.40
```

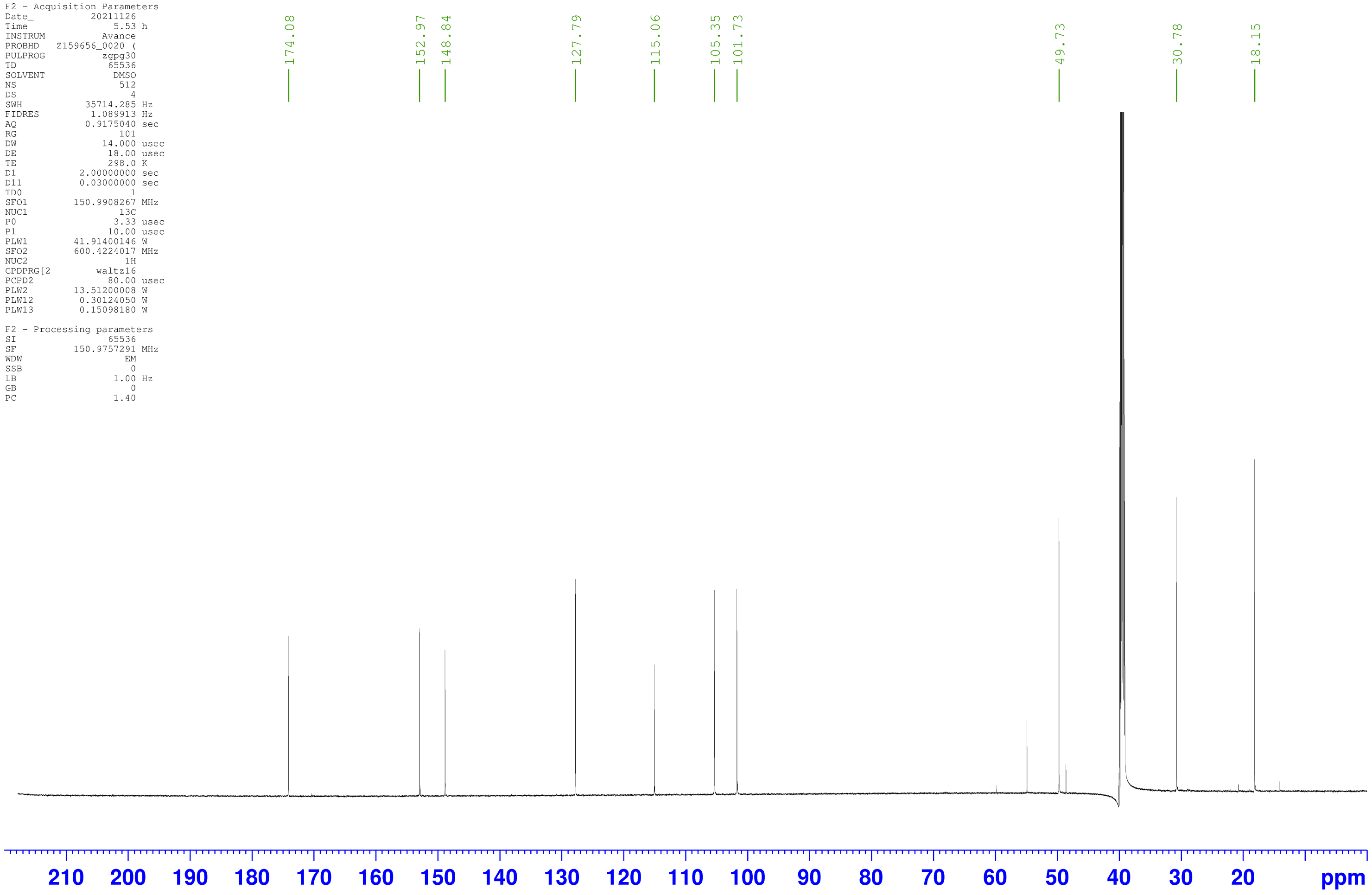

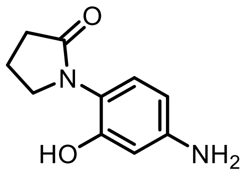

N-[4-(2-Oxopyrrolidin-1-yl)-3-prop-2-ynoxyphenyl]prop-2-enamide (42)

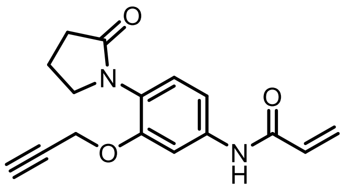

Current Data Parameters
NAME MSEC38_wash_all
EXPNO 1
PROCNO 1

F2 - Acquisition Parameters
Date_ 20220503
Time 13.36 h
INSTRUM avh400
PROBHD Z108618_0873 (
PULPROG zg60
TD 65536
SOLVENT CDCl3
NS 16
DS 2
SWH 8012.820 Hz
FIDRES 0.244532 Hz
AQ 4.0894465 sec
RG 88.17
DW 62.400 usec
DE 6.50 usec
TE 298.0 K
D1 1.00000000 sec
TD0 1
SFO1 400.1324008 MHz
NUC1 1H
P1 14.00 usec
PLW1 14.36999989 W

F2 - Processing parameters
SI 32768
SF 400.1300100 MHz
WDW EM
SSB 0
LB 0.30 Hz
GB 0
PC 1.00

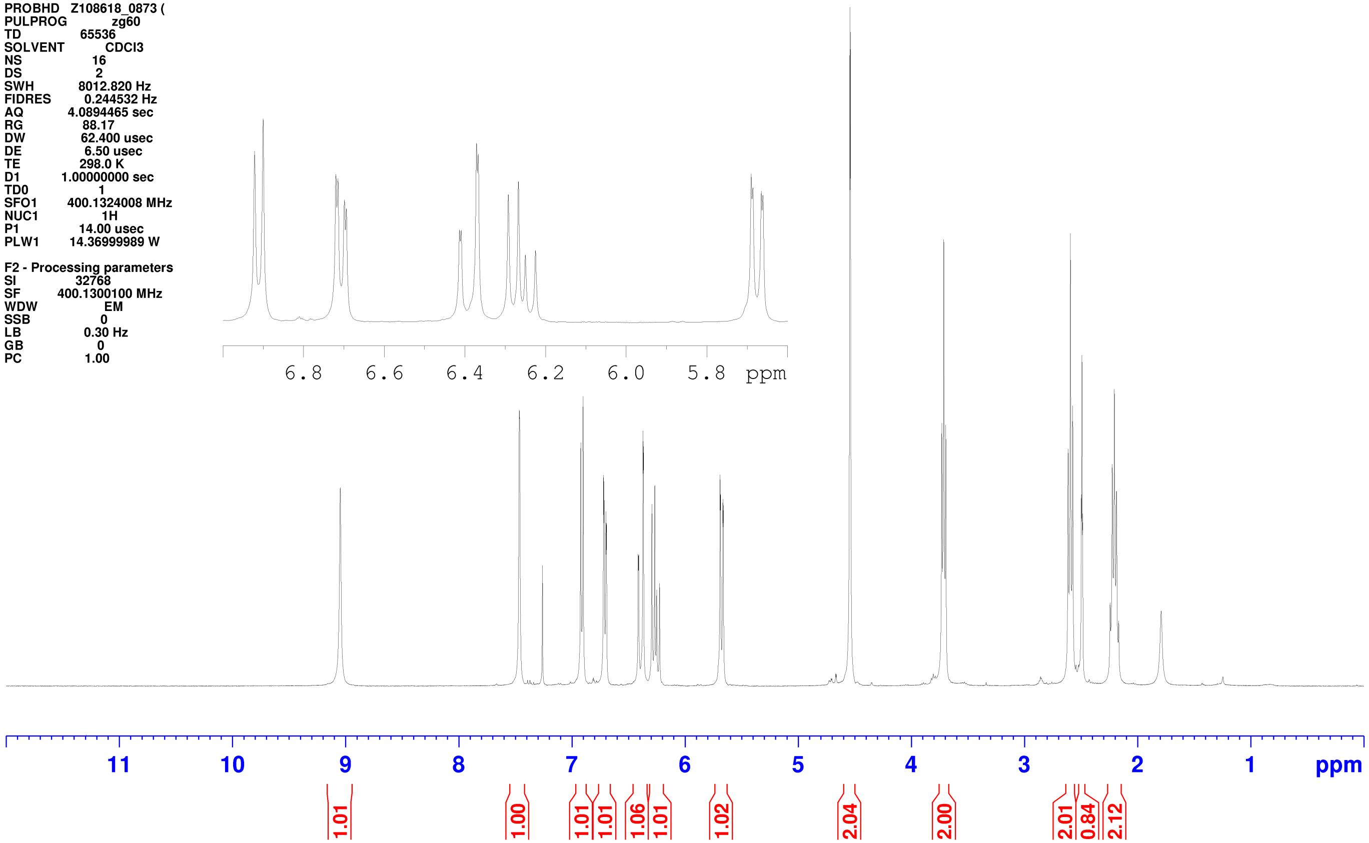

N-[4-(2-Oxopyrrolidin-1-yl)-3-prop-2-ynoxyphenyl]prop-2-enamide (42)

```
Current Data Parameters
NAME      MSEC38_wash_all
EXPNO                   2
PROCNO                  1

F2 - Acquisition Parameters
Date_            20220503
Time                13.59 h
INSTRUM            avh400
PROBHD   Z108618_0873 (
PULPROG            zgpg30
TD                  32768
SOLVENT             CDCl3
NS                    512
DS                      4
SWH             26041.666 Hz
FIDRES           1.589457 Hz
AQ              0.6291456 sec
RG                 197.18
DW                 19.200 usec
DE                   6.50 usec
TE                  298.5 K
D1             1.00000000 sec
D11            0.03000000 sec
TD0                     1
SFO1          100.6228298 MHz
NUC1                  13C
P0                   3.33 usec
P1                  10.00 usec
PLW1          47.86100006 W
SFO2          400.1316005 MHz
NUC2                   1H
CPDPRG[2          waltz16
PCPD2               90.00 usec
PLW2          14.36999989 W
PLW12          0.34772000 W
PLW13          0.17490000 W

F2 - Processing parameters
SI                  32768
SF            100.6127569 MHz
WDW                    EM
SSB                     0
LB                   1.00 Hz
GB                      0
PC                   1.40
```

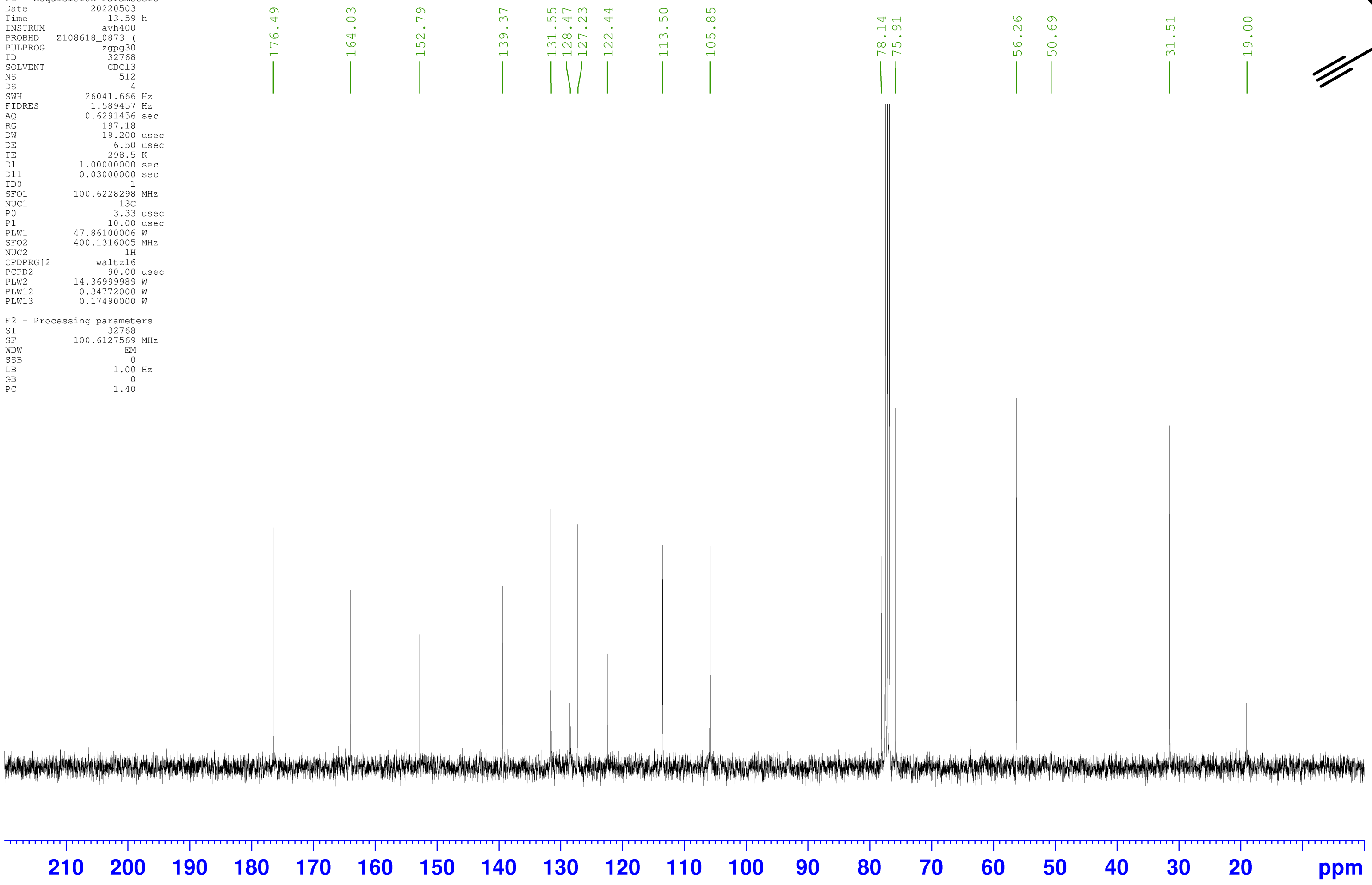

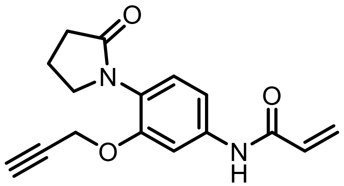

HPLC Traces of Biologically Tested Compounds

1-(3-Methylisoxazol-5-yl)prop-2-en-1-one (4)

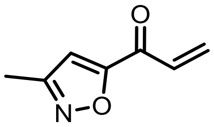

Acquisition Method	Purity short run @254 nm
Acquisition Date/Time	7/4/2018 2:20 pm
Injection Volume	10
Sample Name	AMTA38
Sample Description	
Batch Description	

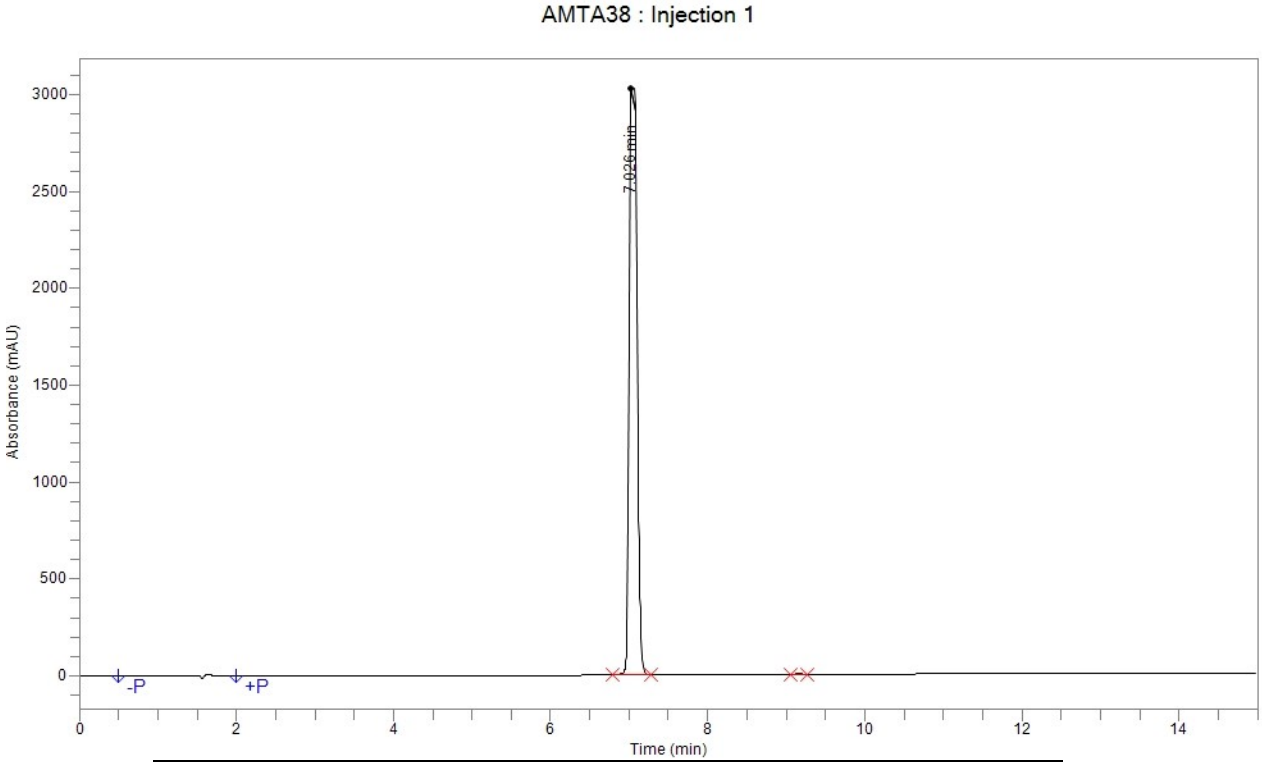

Time	Height	Area	Area %
7.026	3,068,342.4	20,338,130.4	99.85
9.147	6,090.7	30,575.7	0.15
**Total**		20,368,706.2	100.00

1-(5-Methylisoxazol-3-yl)prop-2-en-1-one (5)

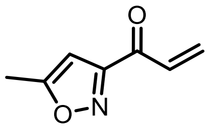

Acquisition Method	Purity short run @254 nm
Acquisition Date/Time	3/14/2019 6:31 pm
Injection Volume	10
Sample Name	AMTB27_254_NoTFA
Sample Description	
Batch Description	For start up

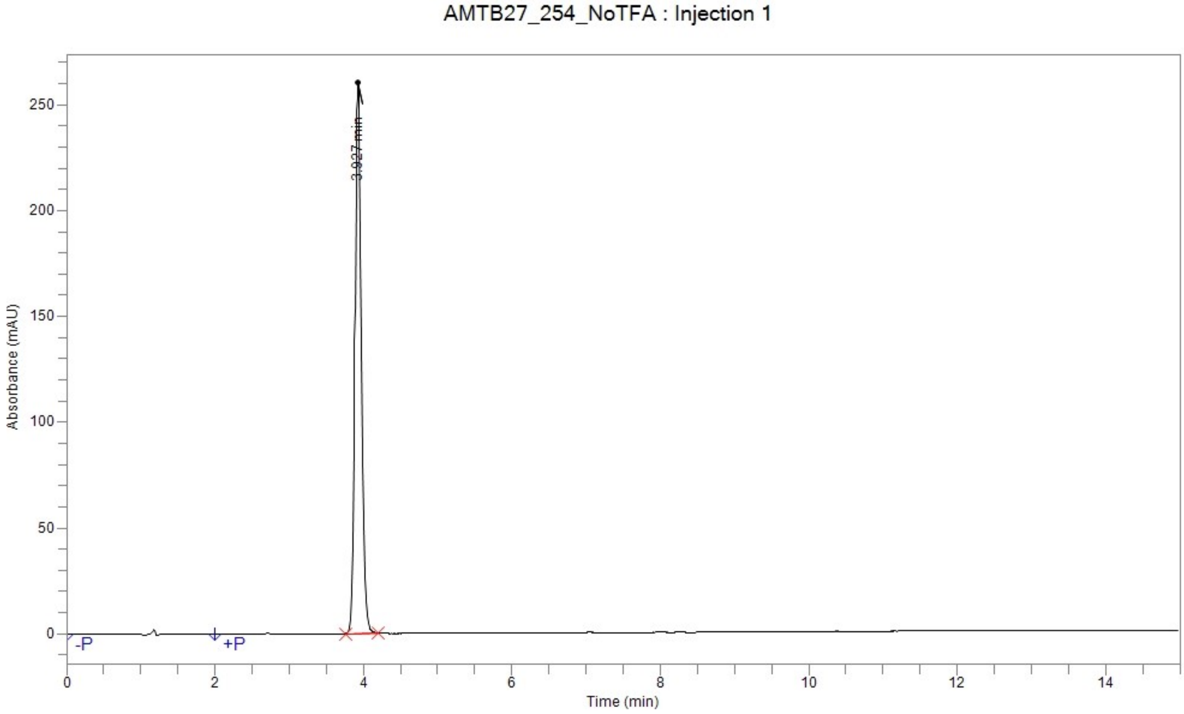

Time	Height	Area	Area %
3.927	260,636.3	1,613,469.5	100.00
**Total**		1,613,469.5	100.00

1-(3-Methyl-4-phenylisoxazol-5-yl)prop-2-en-1-one (6)

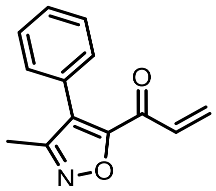

Chromatogram: DA-B@254nm

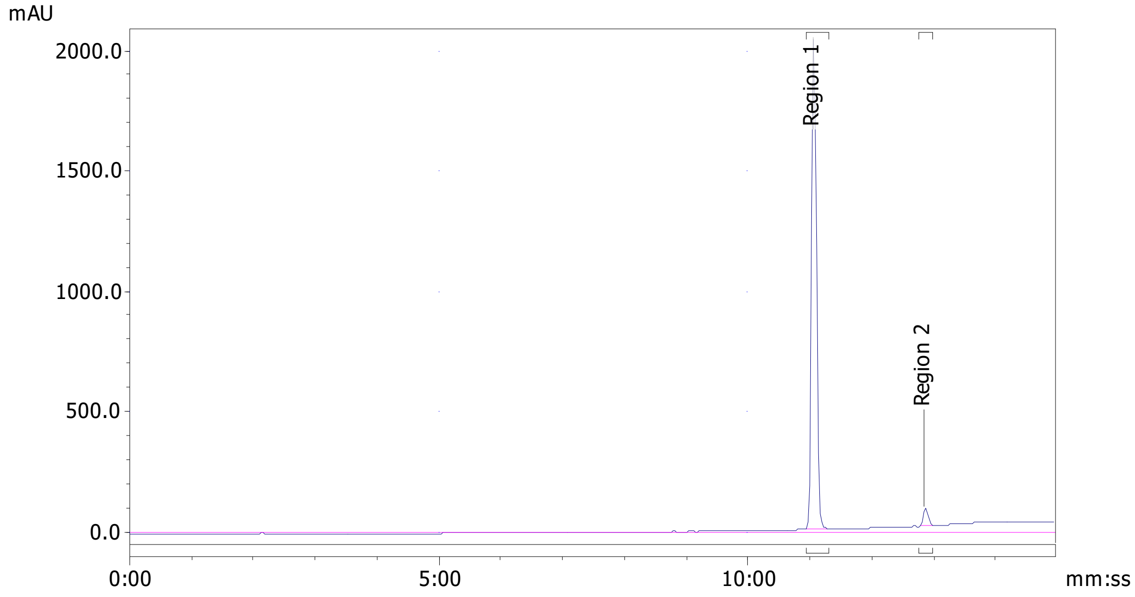

Regions: DA-B@254nm Detector:

Name	Start (mm:ss)	End (mm:ss)	Retention (mm:ss)	Area (mAU·s)	%ROI (%)
Region 1	10:56	11:18	11:03	10192.2	96.59
Region 2	12:45	12:59	12:51	360.2	3.41
2 Peaks				10552.3	100.00

Total Area: 13755.0 mAU
Average Background: N/A mAU

Method: Xbridge_nonRadio_default

Instrument: N/A Serial no FR1A/0217/389
Measured by: Super User on 27 February 2020 22:57:57
Method by: Super User on 19 December 2019 16:17:37
Evaluation by: Super User on 27 February 2020 23:14:03

Run Length: 15m
Dwell: 1s

_E_-3-[Methoxy(methyl)amino]-1-(3-methylisoxazol-5-yl)prop-2-en-1-one (7)

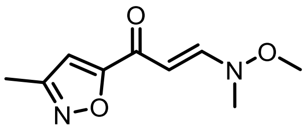

Acquisition Method	Purity short run @254 nm
Acquisition Date/Time	8/13/2019 8:13 pm
Injection Volume	10
Sample Name	AMTB24_TFA_Diluted
Sample Description	
Batch Description	

AMTB24_TFA_Diluted : Injection 1

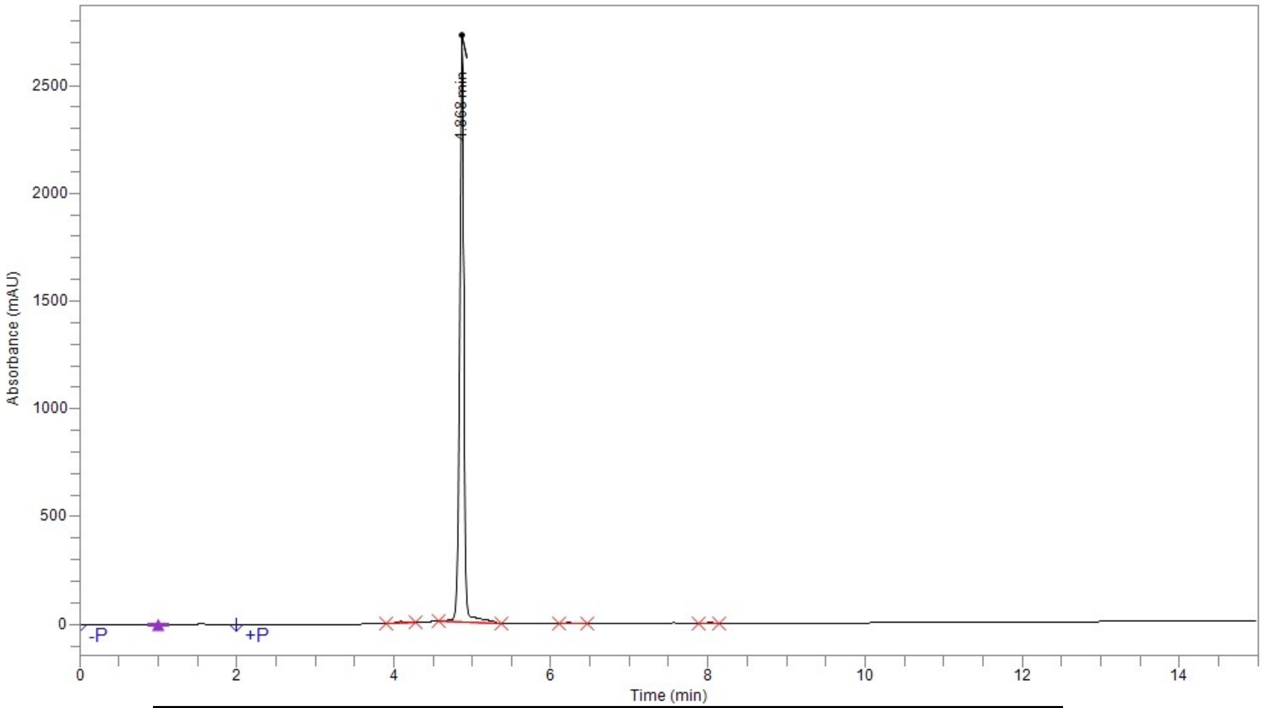

Time	Height	Area	Area %
4.085	7,292.8	49,242.2	0.44
4.868	2,728,756.6	11,055,442.8	98.84
6.225	8,571.1	50,672.5	0.45
8.027	6,759.0	29,280.1	0.26
**Total**		11,184,637.6	100.00

1-(3-Methylisoxazol-5-yl)prop-2-yn-1-one (8)

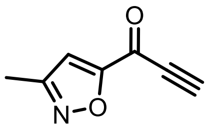

Acquisition Method	Purity short run @254 nm
Acquisition Date/Time	8/13/2019 3:41 pm
Injection Volume	10
Sample Name	AMTB58_TFA
Sample Description	
Batch Description	

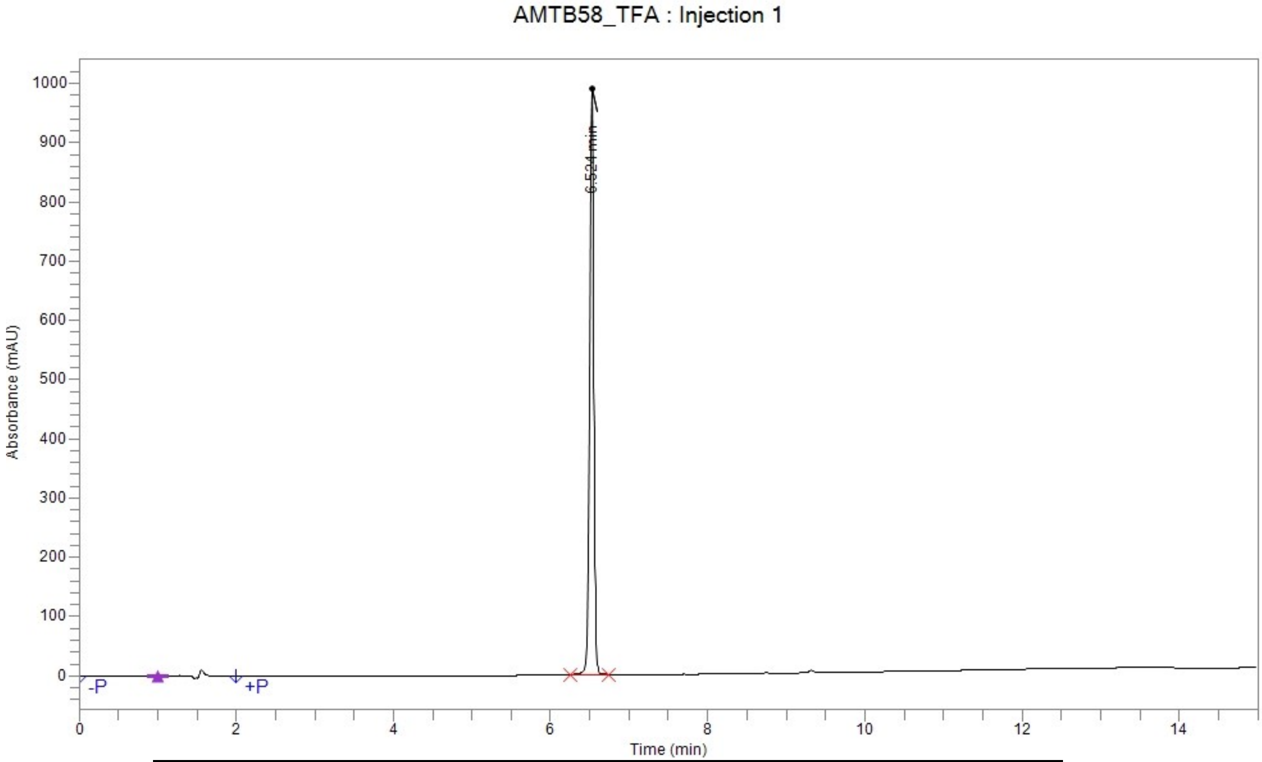

Time	Height	Area	Area %
6.524	989,931.4	3,700,163.9	100.00
**Total**		3,700,163.9	100.00

_N_-(3-Methylisoxazol-5-yl)prop-2-enamide (10)

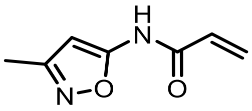

Acquisition Method	Purity short run @254 nm
Acquisition Date/Time	8/13/2019 4:37 pm
Injection Volume	10
Sample Name	AMTA93_TFA
Sample Description	
Batch Description	

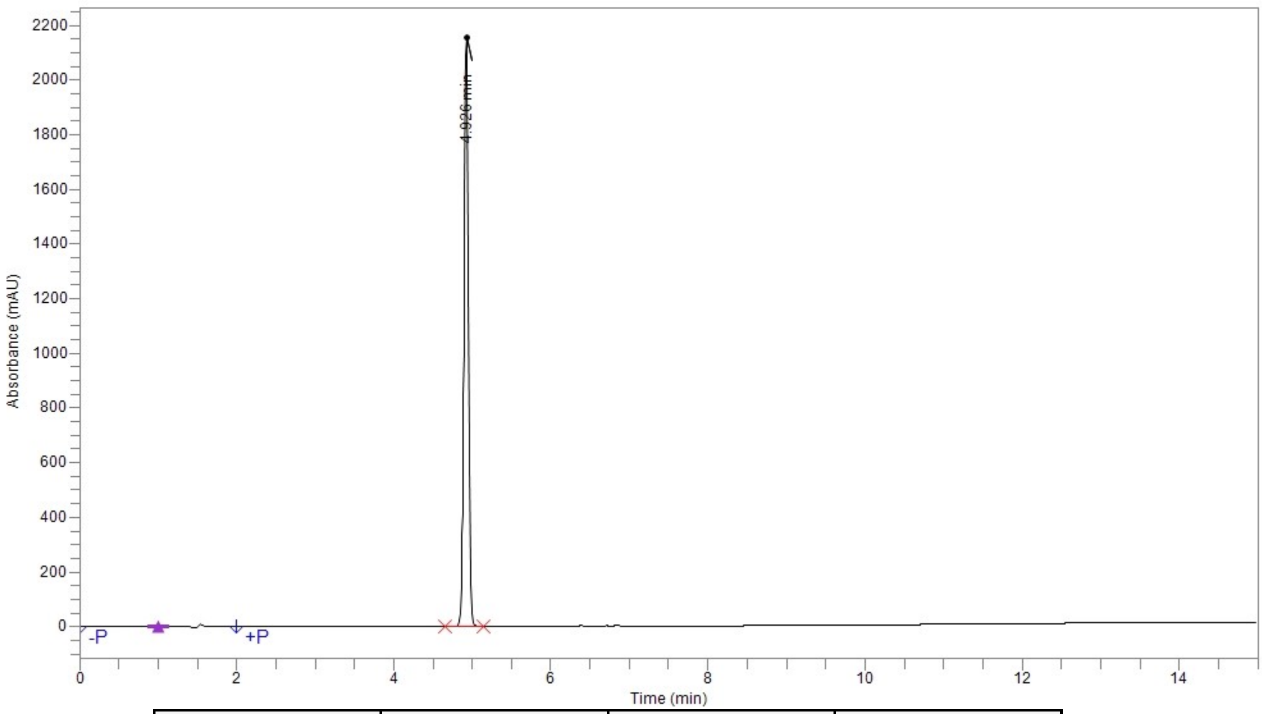

Time	Height	Area	Area %
4.926	2,158,198.5	8,457,705.7	100.00
**Total**		8,457,705.7	100.00

N-(5-Methylisoxazol-3-yl)prop-2-enamide (11)

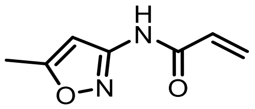

Acquisition Method	Purity short run @254 nm
Acquisition Date/Time	8/13/2019 7:52 pm
Injection Volume	10
Sample Name	AMTA94_TFA
Sample Description	
Batch Description	

AMTA94_TFA : Injection 1

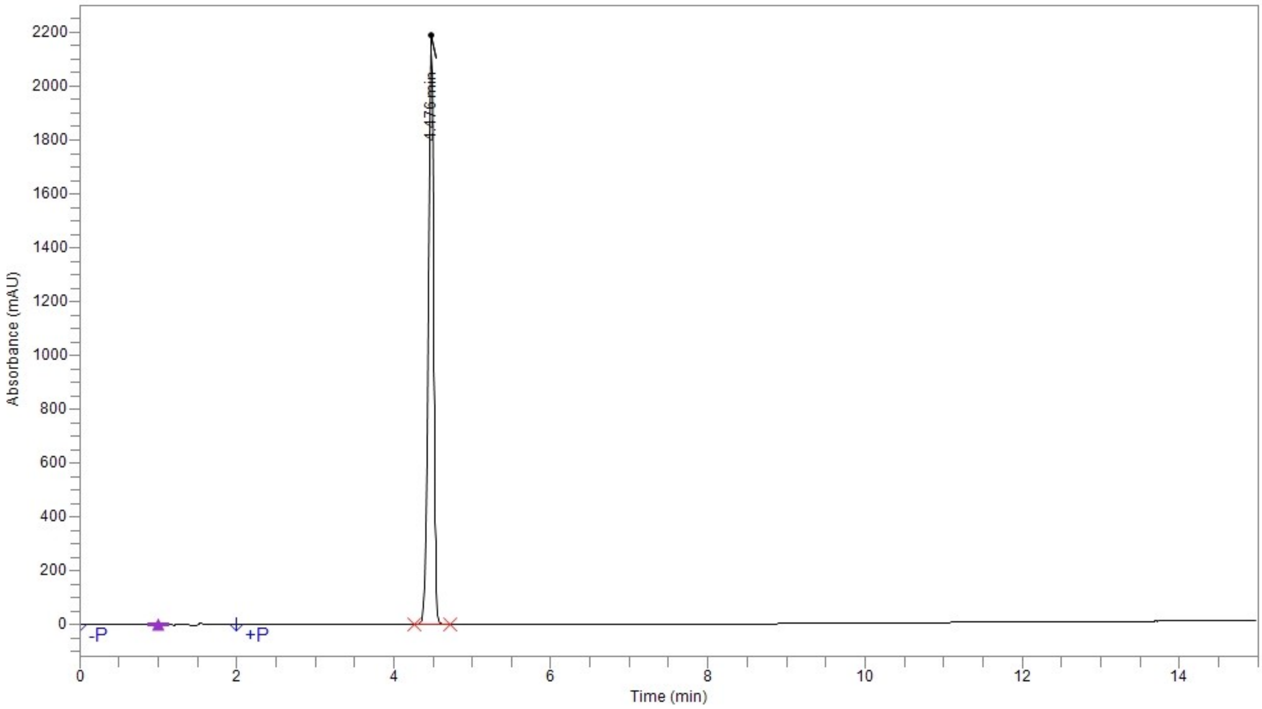

Time	Height	Area	Area %
4.476	2,193,225.0	10,304,018.3	100.00
**Total**		10,304,018.3	100.00

N-(3-Methyl-4-phenylisoxazol-5-yl)prop-2-enamide (12)

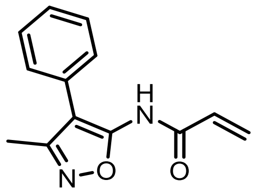

Acquisition Method	Purity short run @254 nm
Acquisition Date/Time	11/27/2020 11:44 am
Injection Volume	10
Sample Name	AMTE09_A5_A6
Sample Description	
Batch Description	

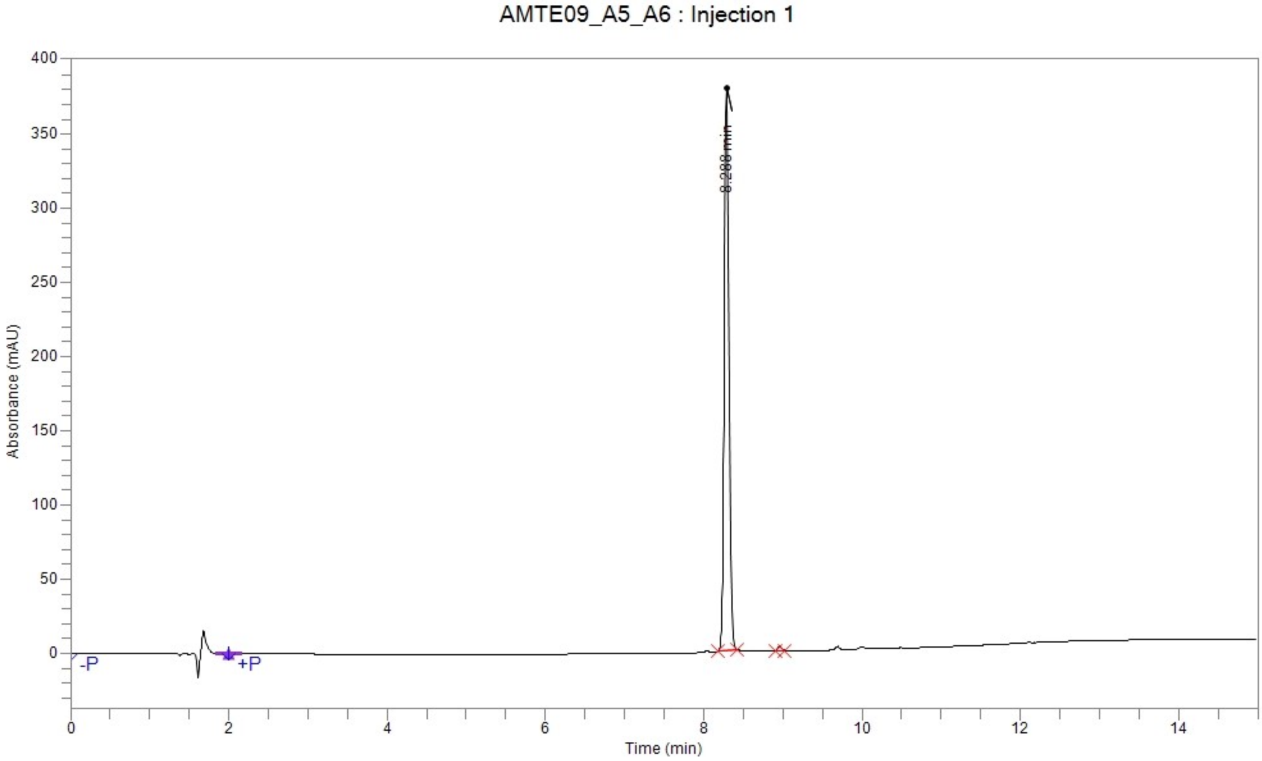

Time	Height	Area	Area %
8.288	380,245.4	1,571,895.9	99.35
8.960	2,907.5	10,333.7	0.65
**Total**		1,582,229.7	100.00

(*E*)-4-(Dimethylamino)-*N*-(3-methylisoxazol-5-yl)but-2-enamide (13)

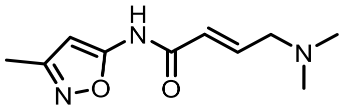

Acquisition Method	AtlantisT3_100A0Bgradient_1mlmin
Acquisition Date/Time	4/1/2019 2:56 pm
Injection Volume	10
Sample Name	AMTA68_TFA
Sample Description	
Batch Description	

AMTA68_TFA : Injection 1

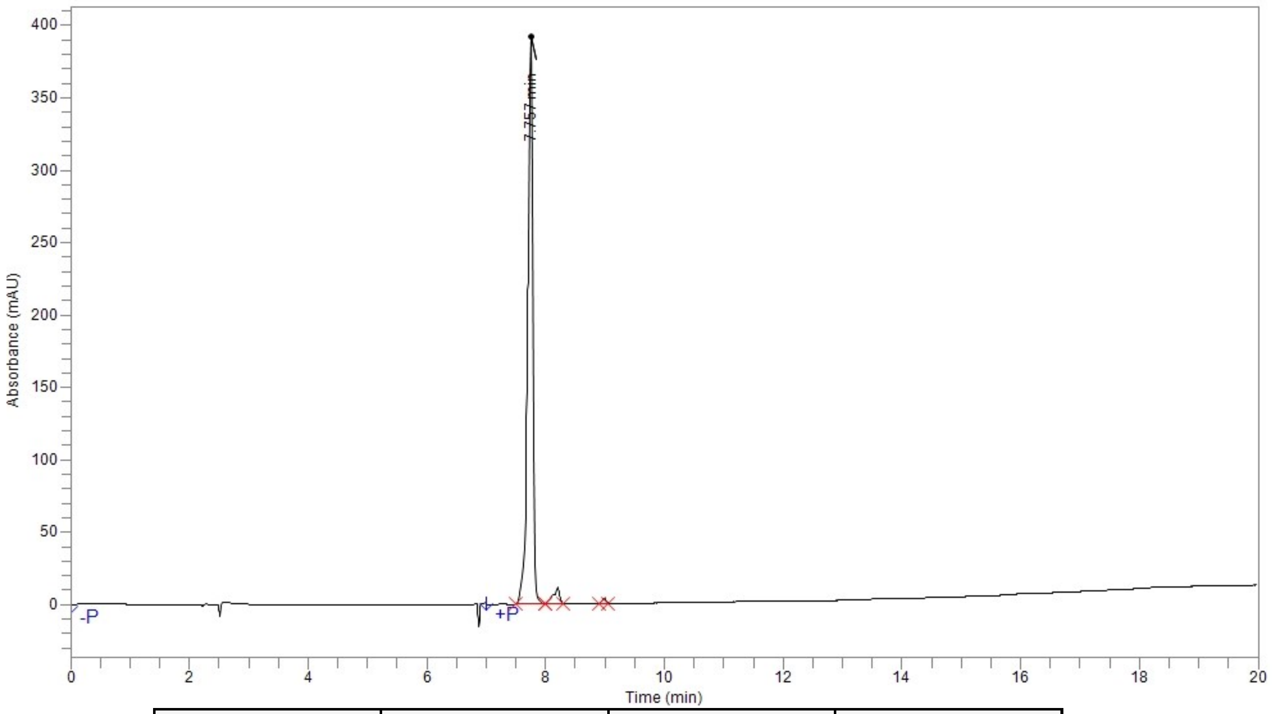

Time	Height	Area	Area %
7.757	392,267.2	2,359,530.2	96.01
8.201	11,287.5	82,209.9	3.35
8.981	3,704.3	15,875.3	0.65
**Total**		2,457,615.4	100.00

(*E*)-4-(Dimethylamino)-*N*-(3-methyl-4-phenylisoxazol-5-yl)but-2-enamide (14)

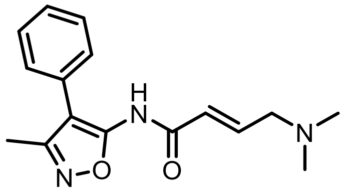

Acquisition Method	Purity short run @254 nm
Acquisition Date/Time	8/25/2021 4:06 pm
Injection Volume	20
Sample Name	AMTE35_Batch2_Prep
Sample Description	
Batch Description	

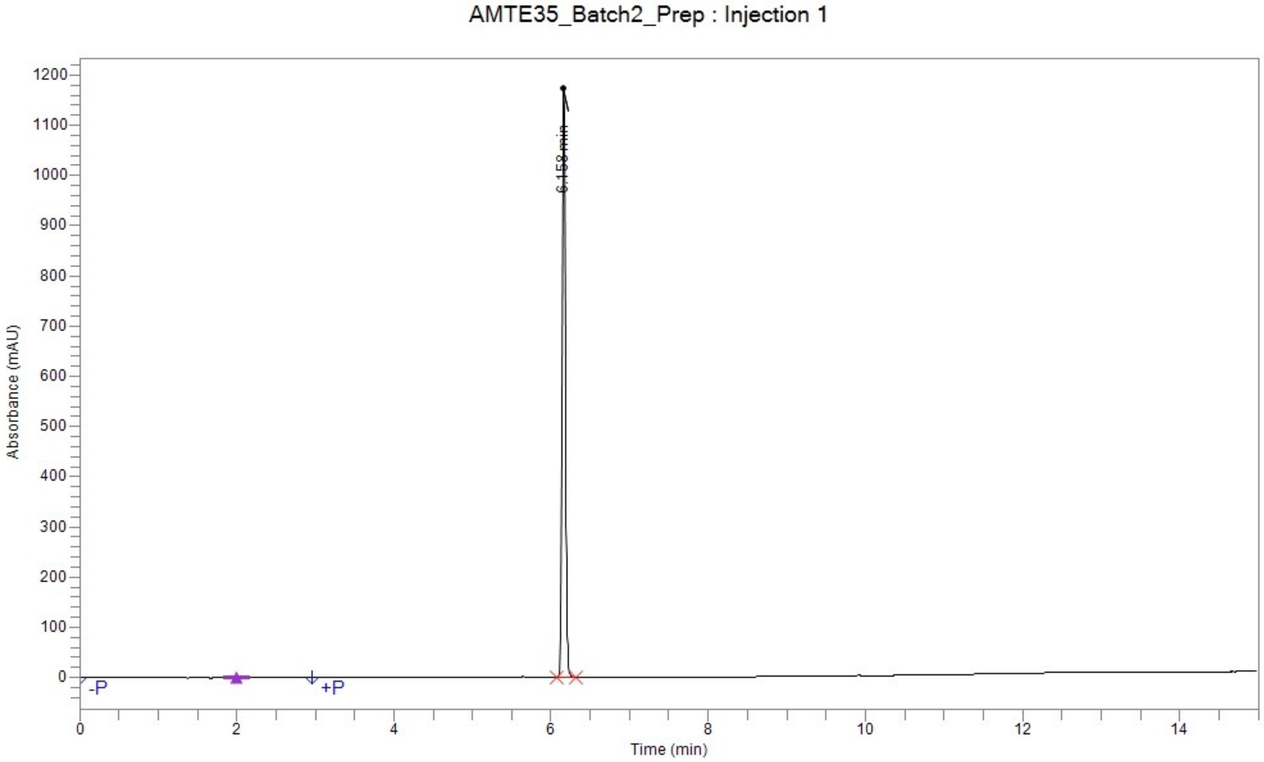

Time	Height	Area	Area %
6.158	1,177,978.5	3,422,609.7	100.00
**Total**		3,422,609.7	100.00

(*E*)-*N*-(3-methyl-4-phenylisoxazol-5-yl)but-2-enamide (15)

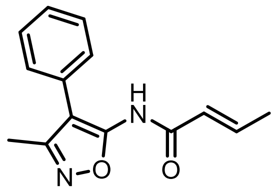

Acquisition Method	Purity short run @254 nm
Acquisition Date/Time	3/24/2021 5:04 pm
Injection Volume	25
Sample Name	AMTE52_Frac16_TFA_254
Sample Description	
Batch Description	

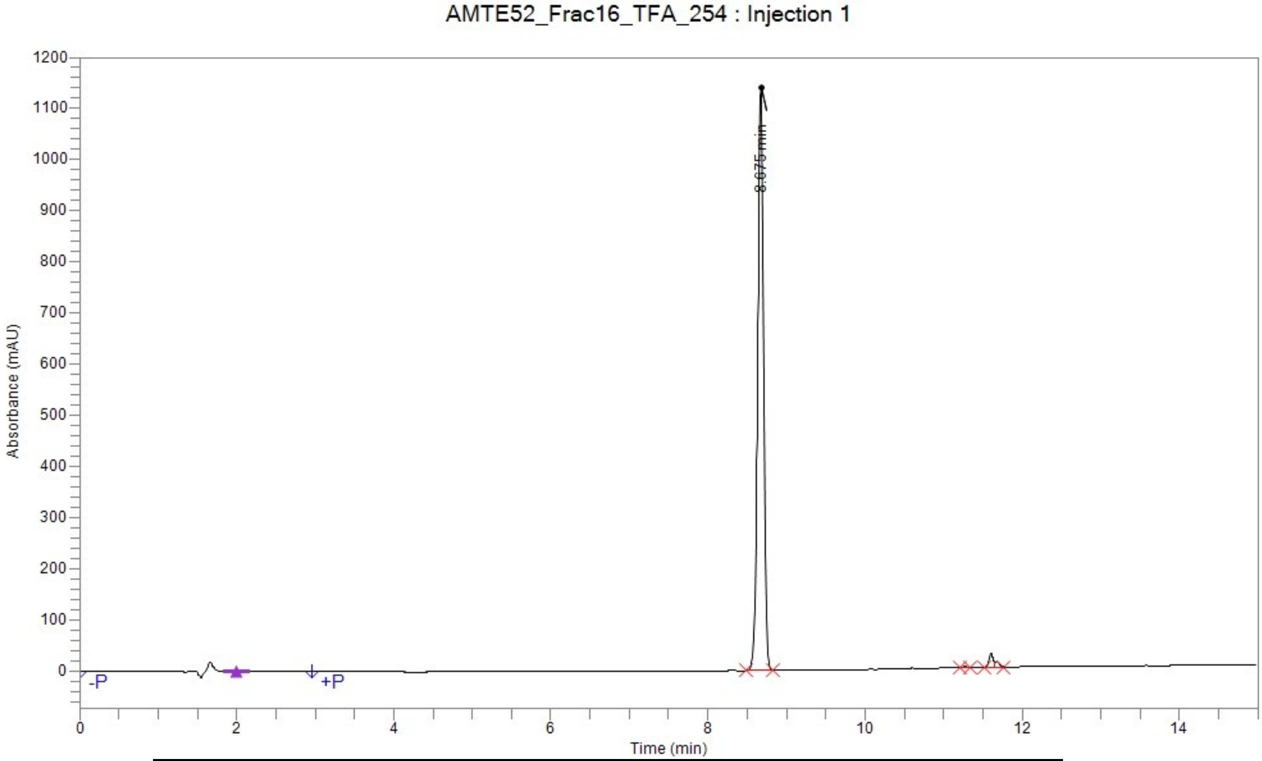

Time	Height	Area	Area %
8.675	1,139,091.5	5,926,915.6	97.46
11.274	3,071.1	10,658.5	0.18
11.603	27,786.6	105,318.8	1.73
11.682	10,567.9	38,351.1	0.63
**Total**		6,081,244.1	100.00

N-[(3-Methylisoxazol-5-yl)methyl]prop-2-enamide (16)

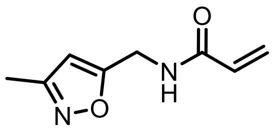

Acquisition Method	Purity short run @254 nm
Acquisition Date/Time	8/13/2019 12:54 pm
Injection Volume	10
Sample Name	AMTB16_TFA
Sample Description	
Batch Description	

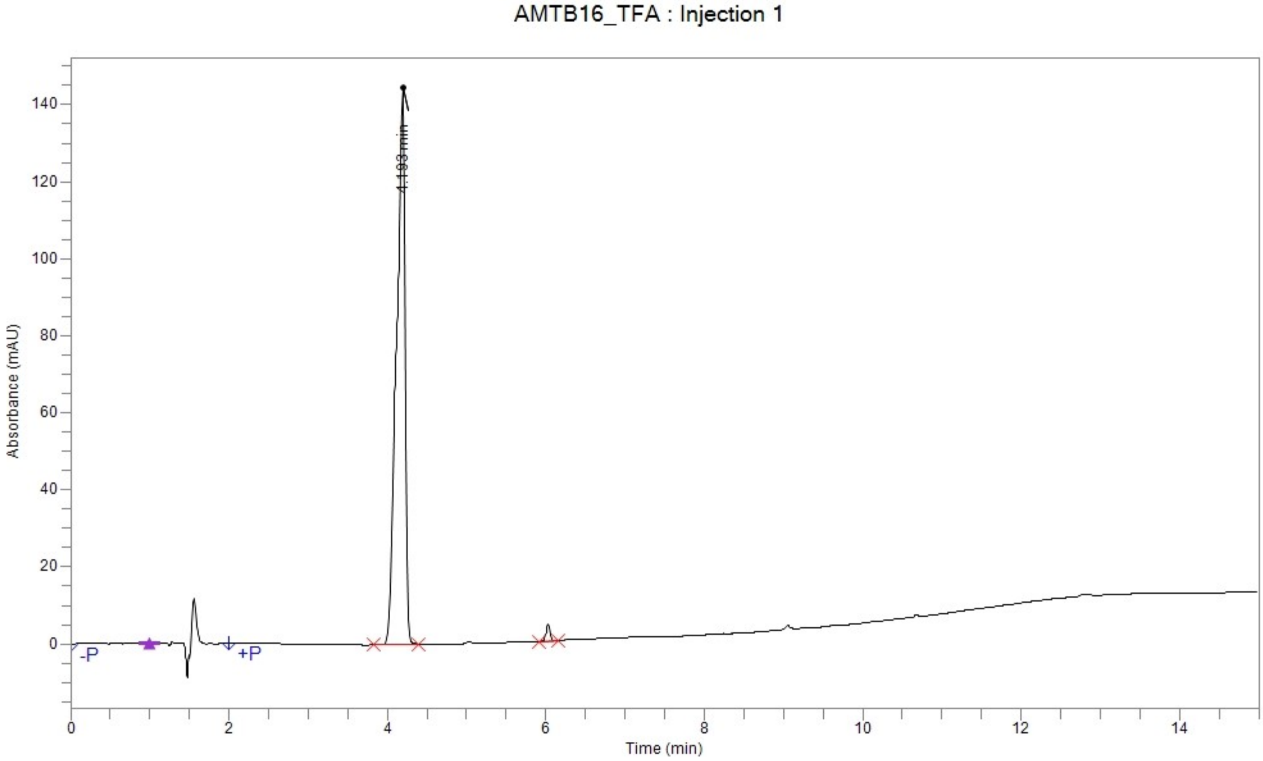

Time	Height	Area	Area %
4.193	144,711.9	1,071,288.0	98.60
6.029	4,536.2	15,199.7	1.40
**Total**		1,086,487.7	100.00

N-[(5-Methylisoxazol-3-yl)methyl]prop-2-enamide (17)

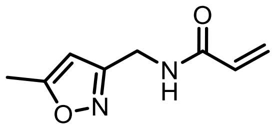

Acquisition Method	Purity short run @254 nm
Acquisition Date/Time	7/4/2018 3:31 pm
Injection Volume	10
Sample Name	AMTA34
Sample Description	
Batch Description	

AMTA34 : Injection 1

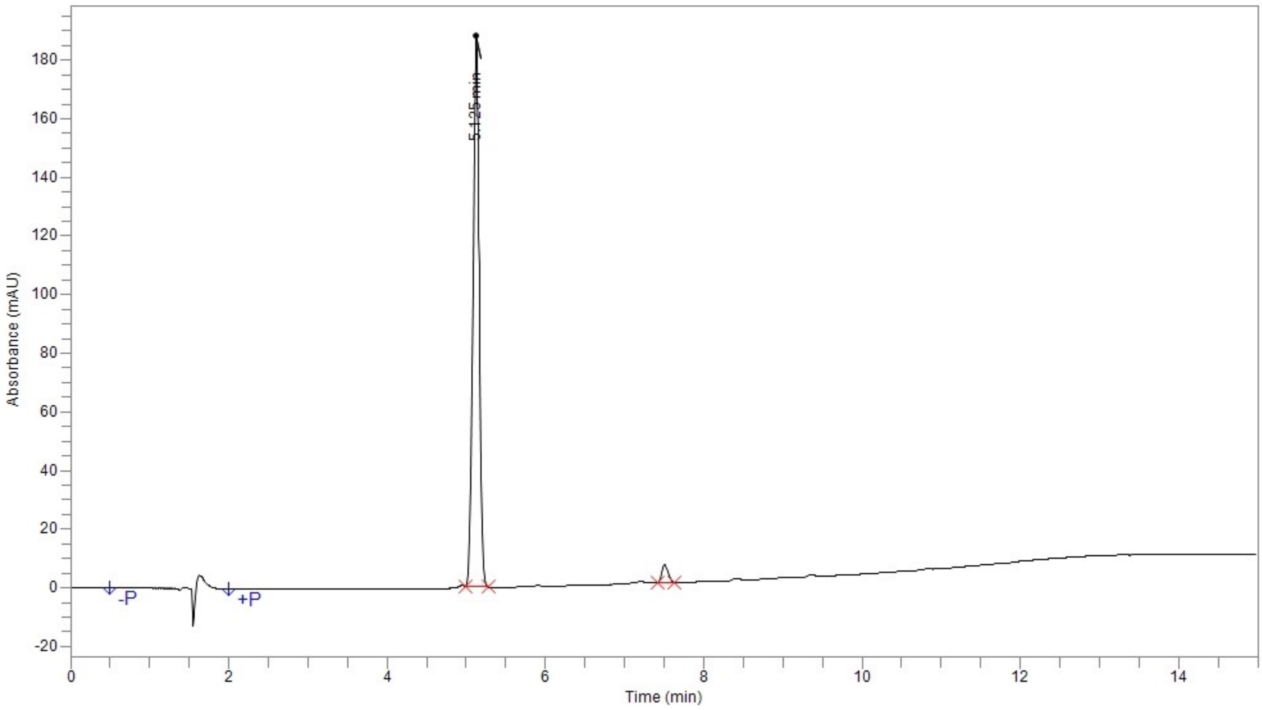

Time	Height	Area	Area %
5.125	188,169.2	1,061,188.7	97.06
7.504	6,127.6	32,106.7	2.94
**Total**		1,093,295.4	100.00

(*E*)-4-(Dimethylamino)-*N*-(3-methylisoxazol-5-yl)but-2-enamide (18)

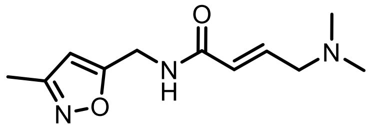

Acquisition Method	AtlantisT3_100A0Bgradient_1mlmin
Acquisition Date/Time	4/1/2019 4:22 pm
Injection Volume	10
Sample Name	AMTB18_TFA
Sample Description	
Batch Description	

AMTB18_TFA : Injection 1

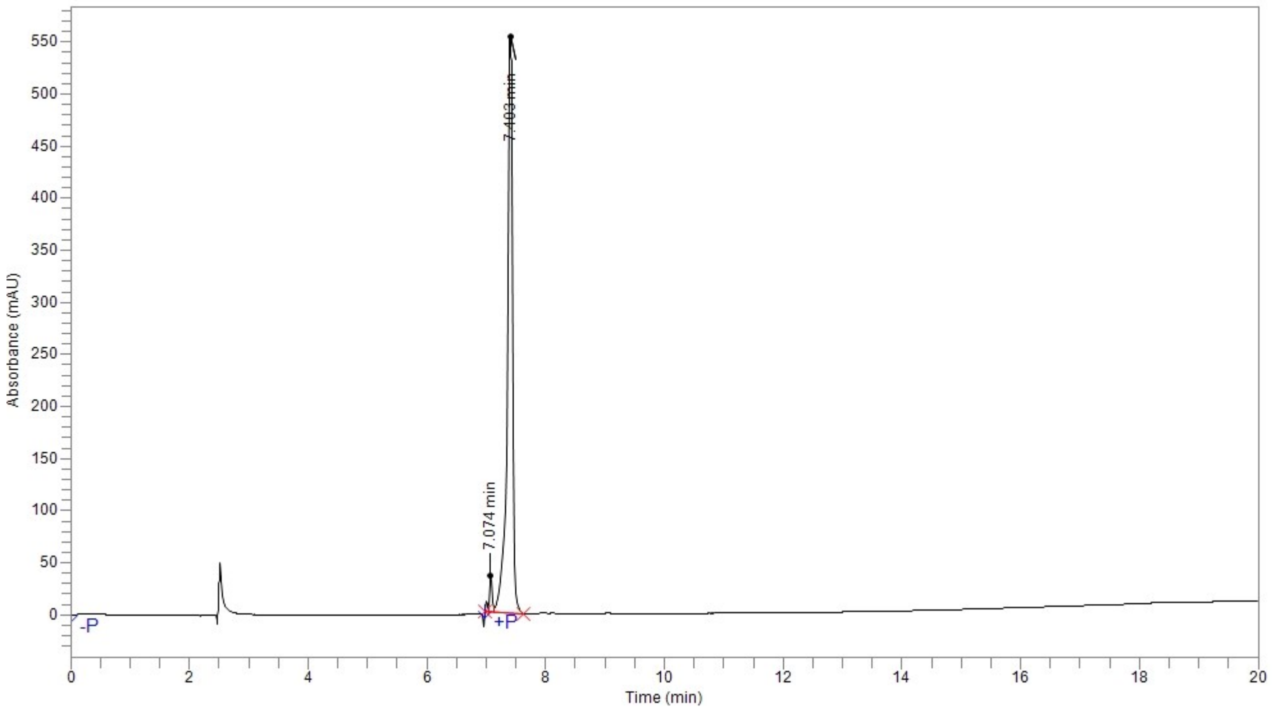

Time	Height	Area	Area %
7.001	10,662.5	25,140.0	0.64
7.074	35,493.4	98,613.2	2.52
7.403	554,033.7	3,788,883.0	96.84
**Total**		3,912,636.2	100.00

N-(3-Methylisoxazol-5-yl)prop-2-ynamide (19)

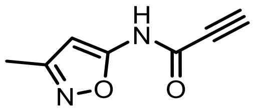

Acquisition Method	Purity short run @254 nm
Acquisition Date/Time	12/18/2018 4:51 pm
Injection Volume	10
Sample Name	AMTA88
Sample Description	
Batch Description	

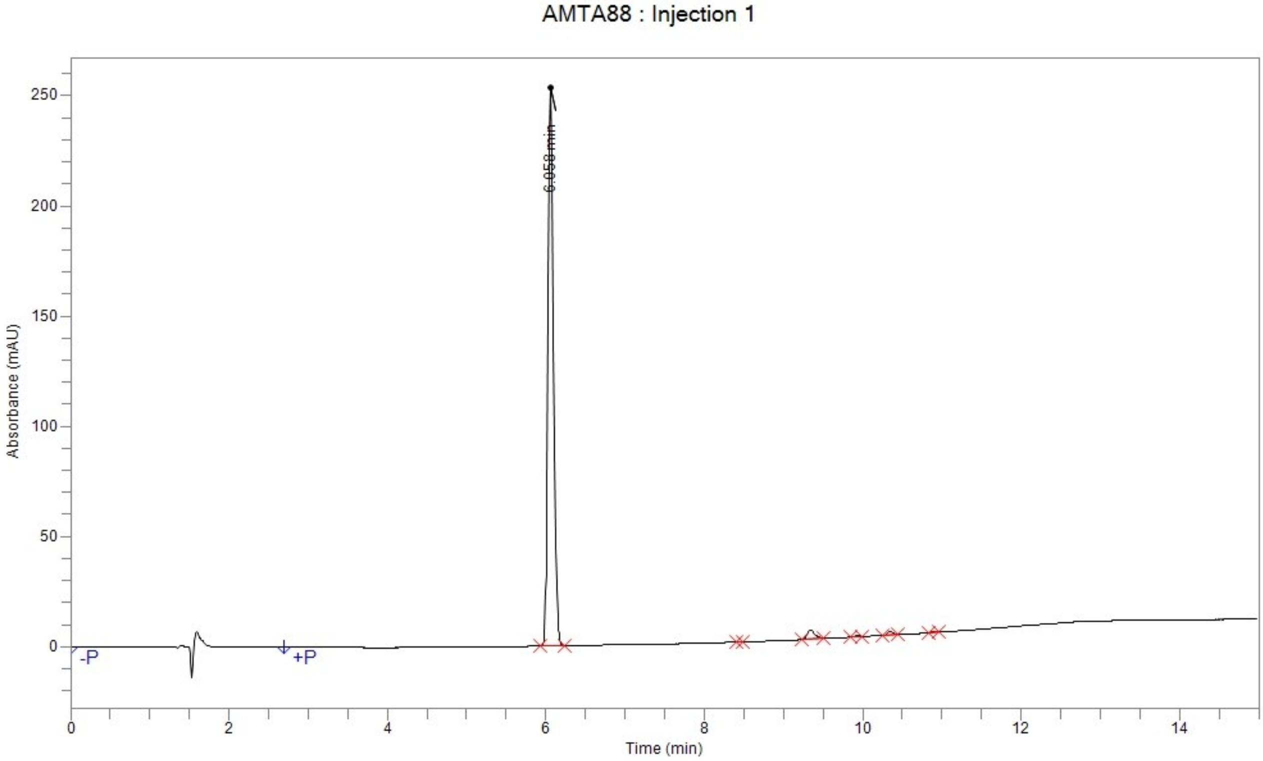

Time	Height	Area	Area %
6.058	253,950.3	1,158,796.7	96.92
8.461	103.2	384.4	0.03
9.344	4,090.2	25,054.0	2.10
9.936	456.0	2,125.3	0.18
10.351	1,536.8	7,216.3	0.60
10.914	482.6	2,088.7	0.17
**Total**		1,195,665.5	100.00

N-(3-Methylisoxazol-5-yl)but-2-ynamide (20)

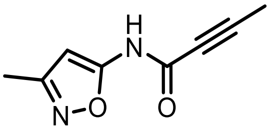

Acquisition Method	Purity short run @254 nm
Acquisition Date/Time	4/30/2019 1:34 pm
Injection Volume	10
Sample Name	AMTB56_TFA_254
Sample Description	
Batch Description	

AMTB56_TFA_254 : Injection 1

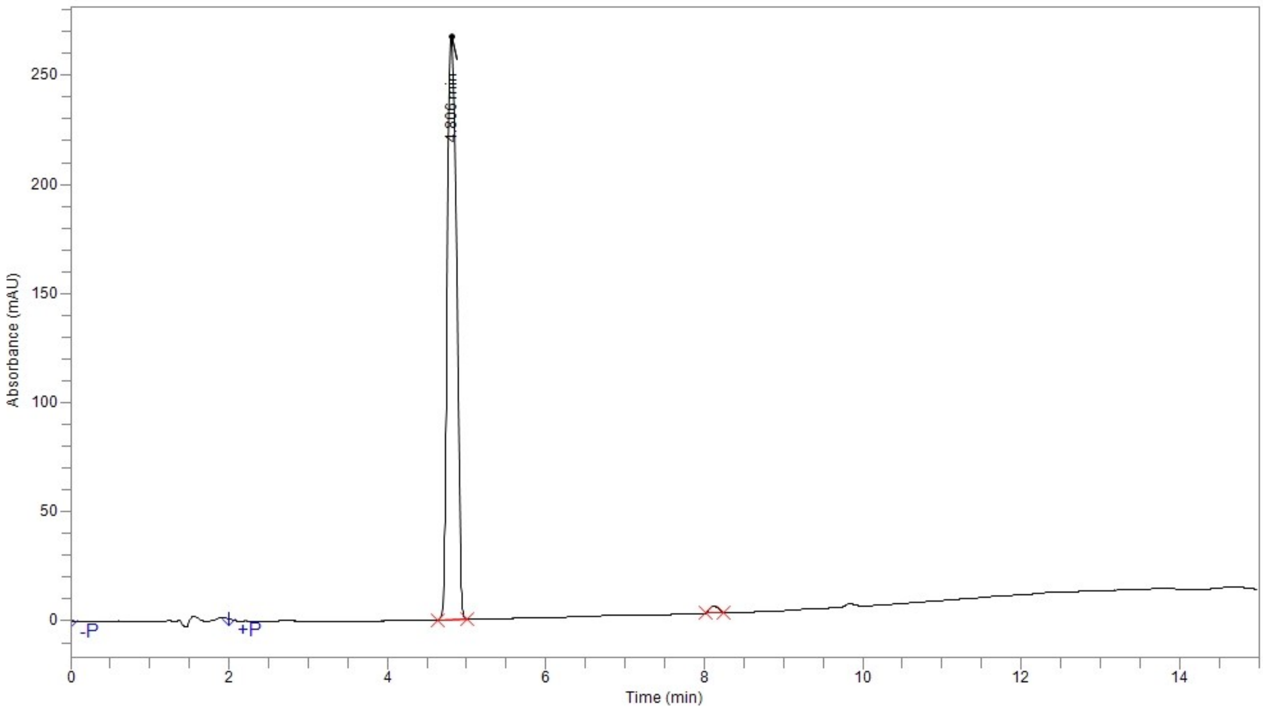

Time	Height	Area	Area %
4.806	267,371.3	2,105,133.2	98.88
8.112	3,181.7	23,817.1	1.12
**Total**		2,128,950.3	100.00

2-Chloro-*N*-(3-methylisoxazol-5-yl)acetamide (21)

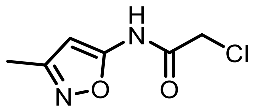

Acquisition Method	Purity short run @254 nm
Acquisition Date/Time	8/13/2019 12:18 pm
Injection Volume	10
Sample Name	AMTB09_TFA
Sample Description	
Batch Description	

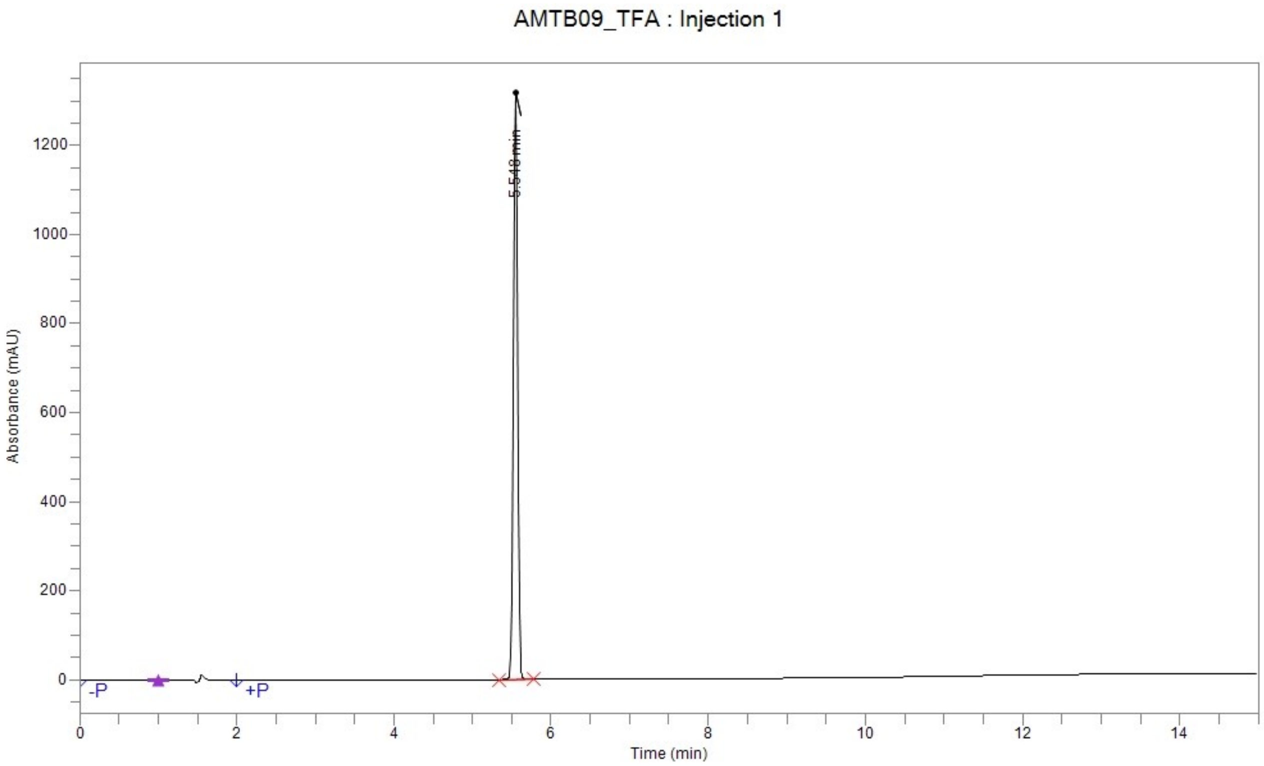

Time	Height	Area	Area %
5.548	1,320,787.4	4,528,342.3	100.00
**Total**		4,528,342.3	100.00

2-Chloro-*N*-(3-methyl-4-phenylisoxazol-5-yl)acetamide (22)

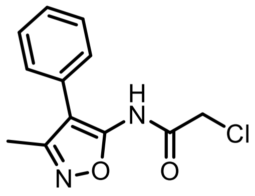

Acquisition Method	Purity short run @254 nm
Acquisition Date/Time	11/19/2019 1:43 pm
Injection Volume	10
Sample Name	AMTC21_TFA_254
Sample Description	
Batch Description	

AMTC21_TFA_254 : Injection 1

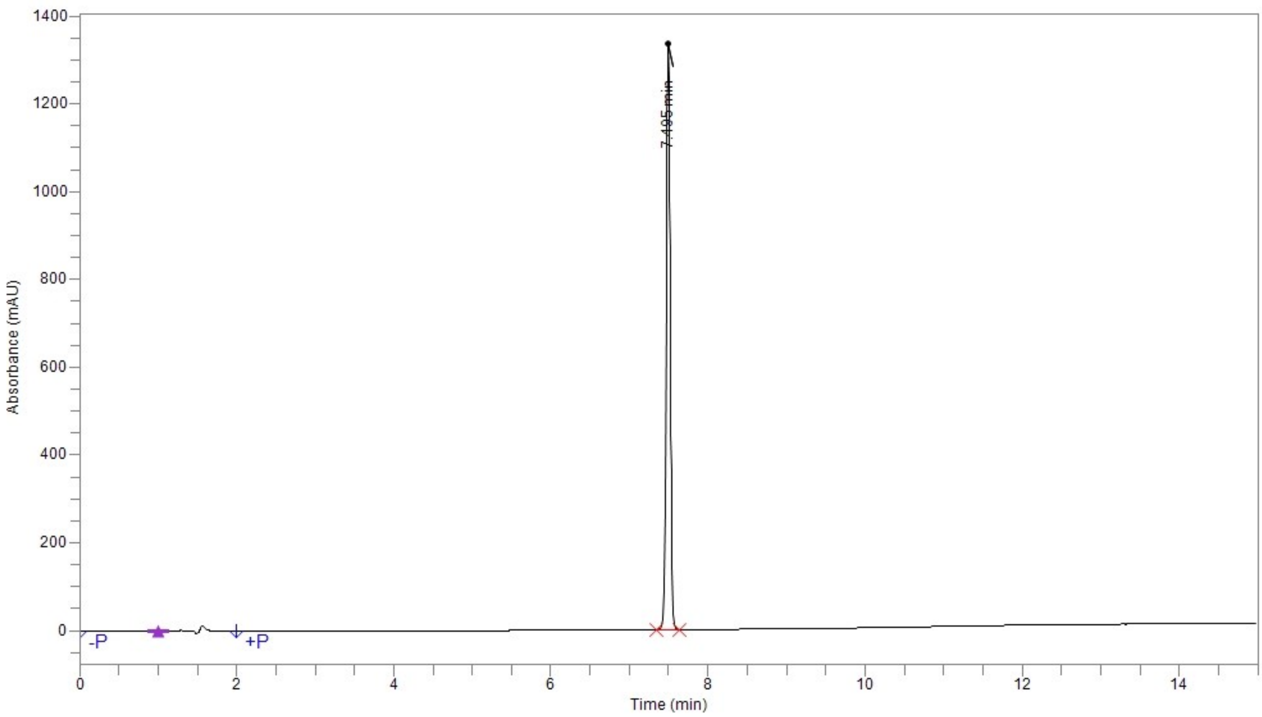

Time	Height	Area	Area %
7.495	1,338,791.3	4,757,803.5	100.00
**Total**		4,757,803.5	100.00

N-(3-Methylisoxazol-5-yl)ethenesulfonamide (23)

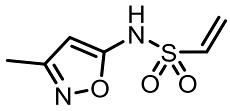

Acquisition Method	Purity short run @254 nm
Acquisition Date/Time	8/13/2019 2:46 pm
Injection Volume	10
Sample Name	AMTB23_TFA
Sample Description	
Batch Description	

AMTB23_TFA : Injection 1

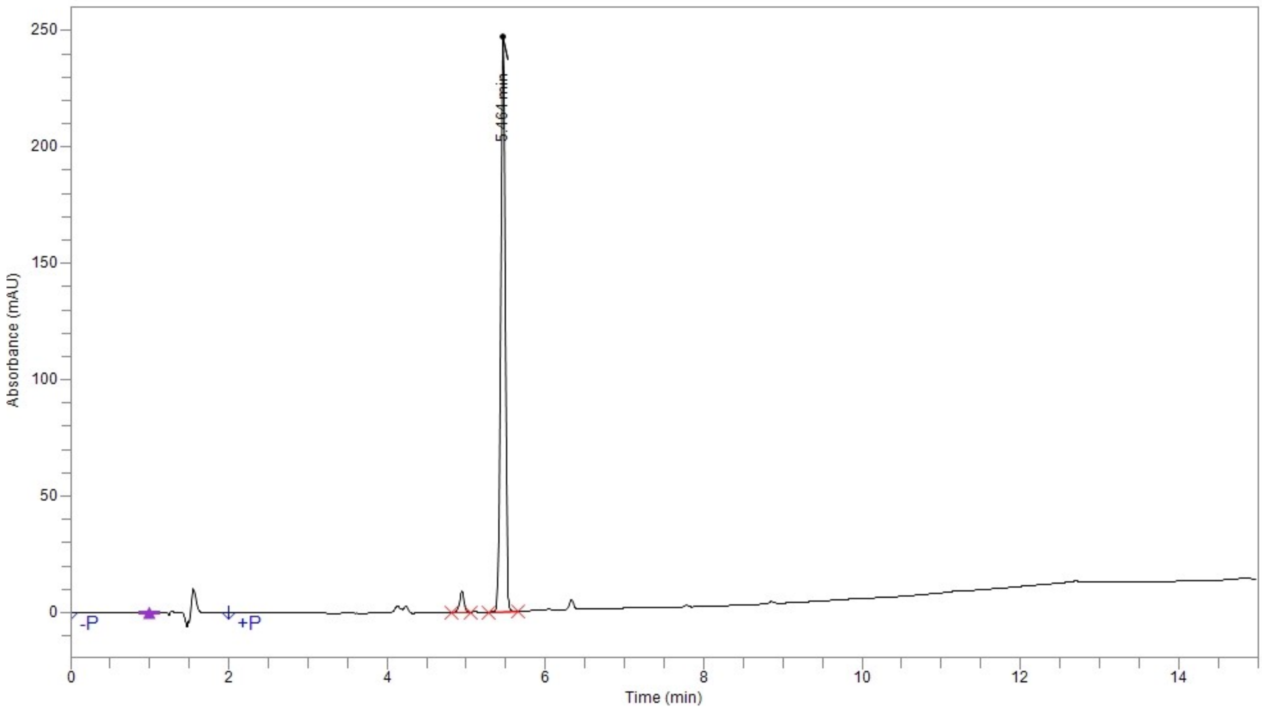

Time	Height	Area	Area %
4.946	9,176.7	38,664.6	4.04
5.464	247,494.2	918,993.6	95.96
**Total**		957,658.2	100.00

N-(3-Methyl-isoxazol-5-yl)propanamide (24)

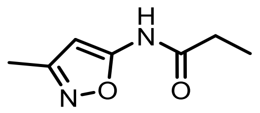

Acquisition Method	Purity short run @254 nm
Acquisition Date/Time	8/26/2021 4:22 pm
Injection Volume	10
Sample Name	AMTC32_254
Sample Description	
Batch Description	

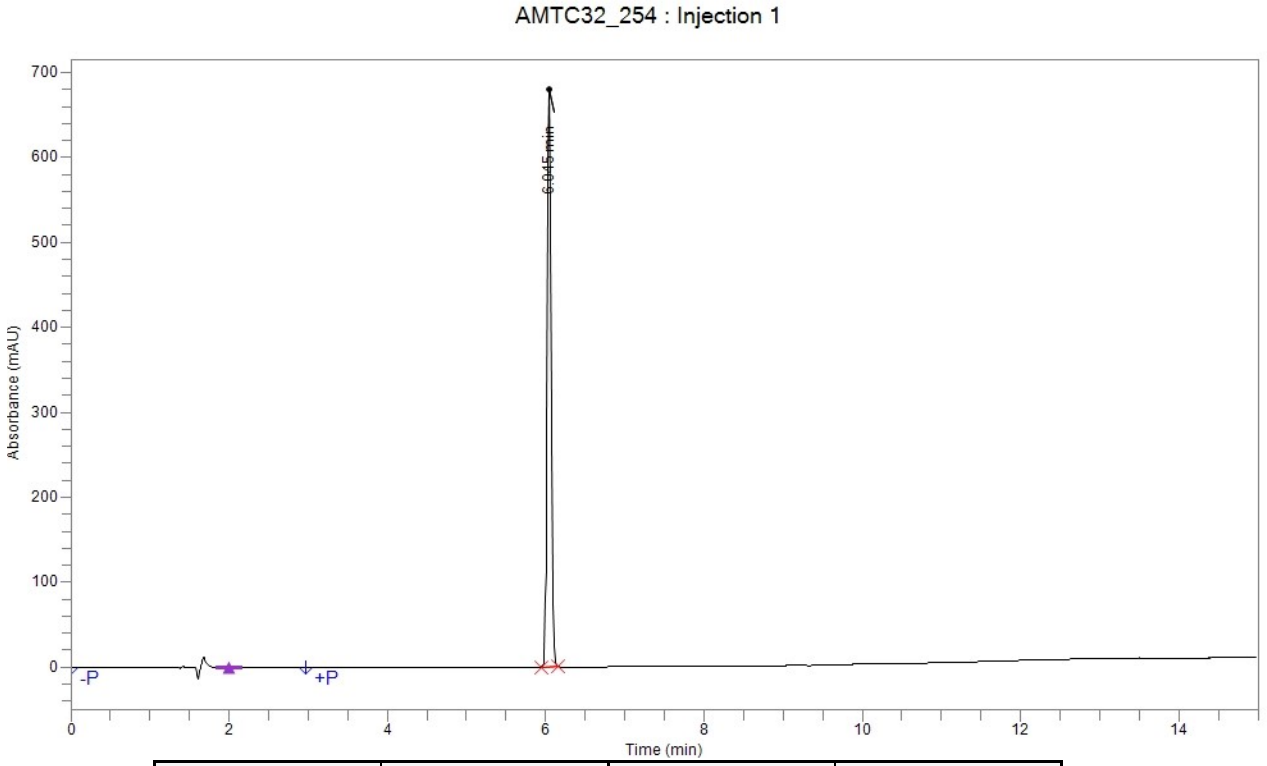

Time	Height	Area	Area %
6.045	681,891.1	2,303,092.0	100.00
**Total**		2,303,092.0	100.00

4-(Dimethylamino)-*N*-(3-methyl-4-phenylisoxazol-5-yl)butanamide (25)

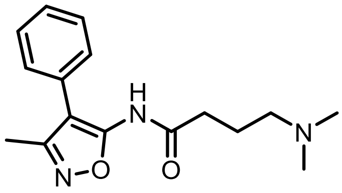

Acquisition Method	Purity short run @254 nm
Acquisition Date/Time	5/26/2021 1:24 pm
Injection Volume	20
Sample Name	AMTE65_B3_TFA
Sample Description	
Batch Description	

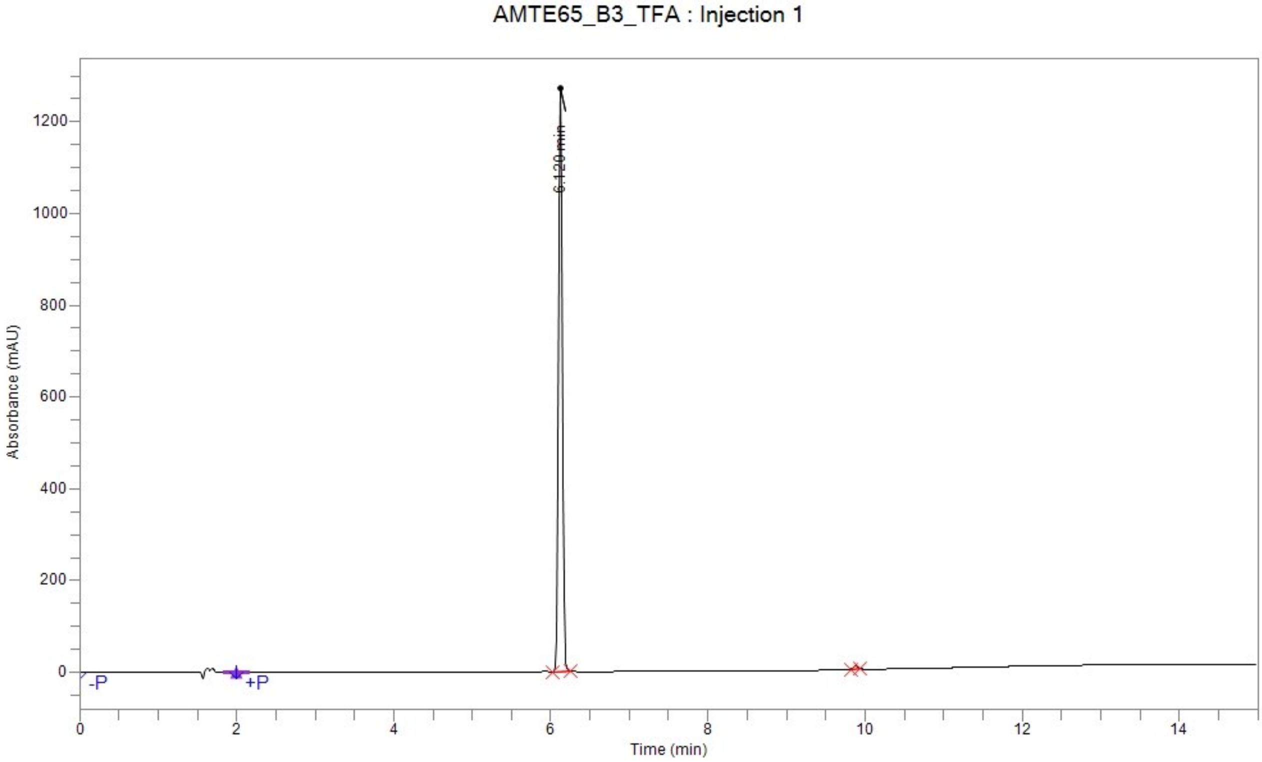

Time	Height	Area	Area %
6.120	1,272,655.8	4,025,006.1	99.43
9.877	6,023.4	23,013.7	0.57
**Total**		4,048,019.8	100.00

(*E*)-4-(Dimethylamino)-*N*-[4-(4-ethynylphenyl)-3-methylisoxazol-5-yl]but-2-enamide (28)

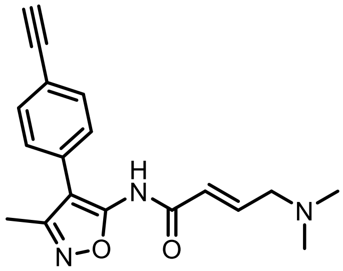

Acquisition Method	Purity short run @254 nm
Acquisition Date/Time	11/11/2020 4:58 pm
Injection Volume	30
Sample Name	AMTE08_MoreTFA_in_sample
Sample Description	
Batch Description	

AMTE08_MoreTFA_in_sample : Injection 1

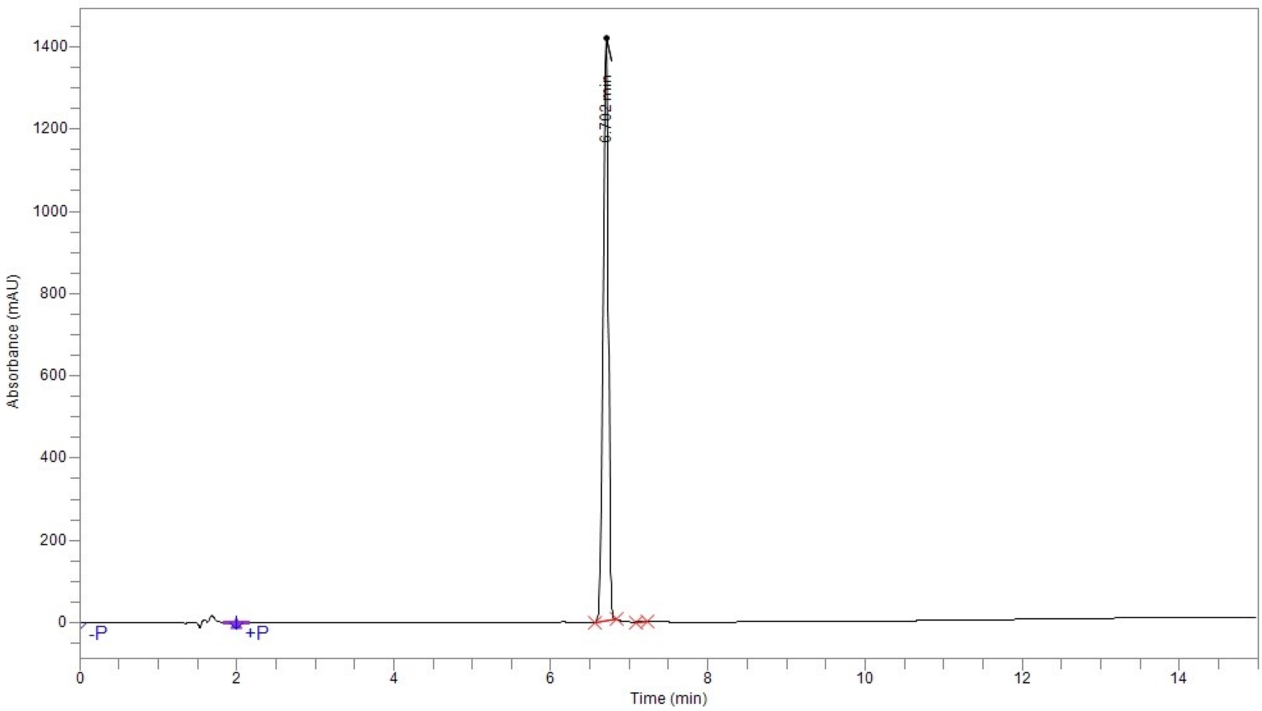

Time	Height	Area	Area %
6.702	1,419,945.7	6,382,551.5	99.81
7.161	2,722.6	12,386.4	0.19
**Total**		6,394,937.9	100.00

(*E*)-4-(Dimethylamino)-*N*-[3-methyl-4-(4-prop-2-ynoxyphenyl)-isoxazol-5-yl]but-2-enamide (29)

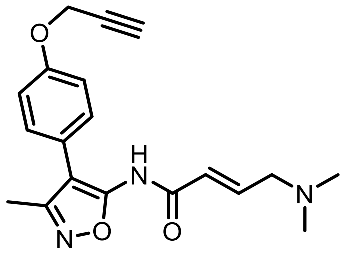

Acquisition Method	Purity short run @254 nm
Acquisition Date/Time	8/24/2021 3:46 pm
Injection Volume	30
Sample Name	AMTE84_Prep
Sample Description	
Batch Description	

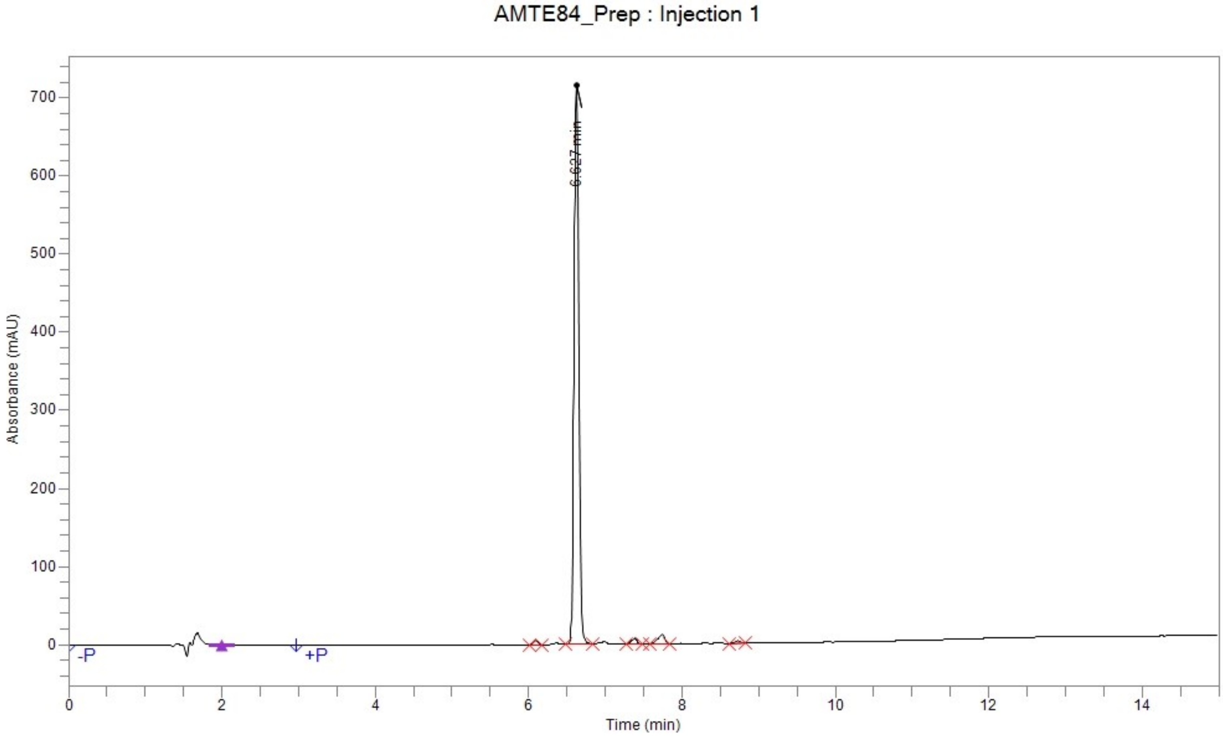

Time	Height	Area	Area %
6.091	5,127.0	21,780.9	0.65
6.627	716,332.9	3,229,772.0	95.69
7.385	7,400.9	37,582.1	1.11
7.739	11,941.5	70,618.9	2.09
8.736	3,324.8	15,610.2	0.46
**Total**		3,375,364.1	100.00

N-[3-Methyl-4-(4-prop-2-ynoxyphenyl)-isoxazol-5-yl]prop-2-enamide (30)

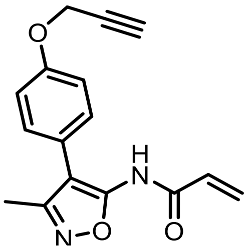

Acquisition Method	Purity short run @254 nm
Acquisition Date/Time	8/5/2021 3:56 pm
Injection Volume	30
Sample Name	AMTE63_254_30ul
Sample Description	
Batch Description	

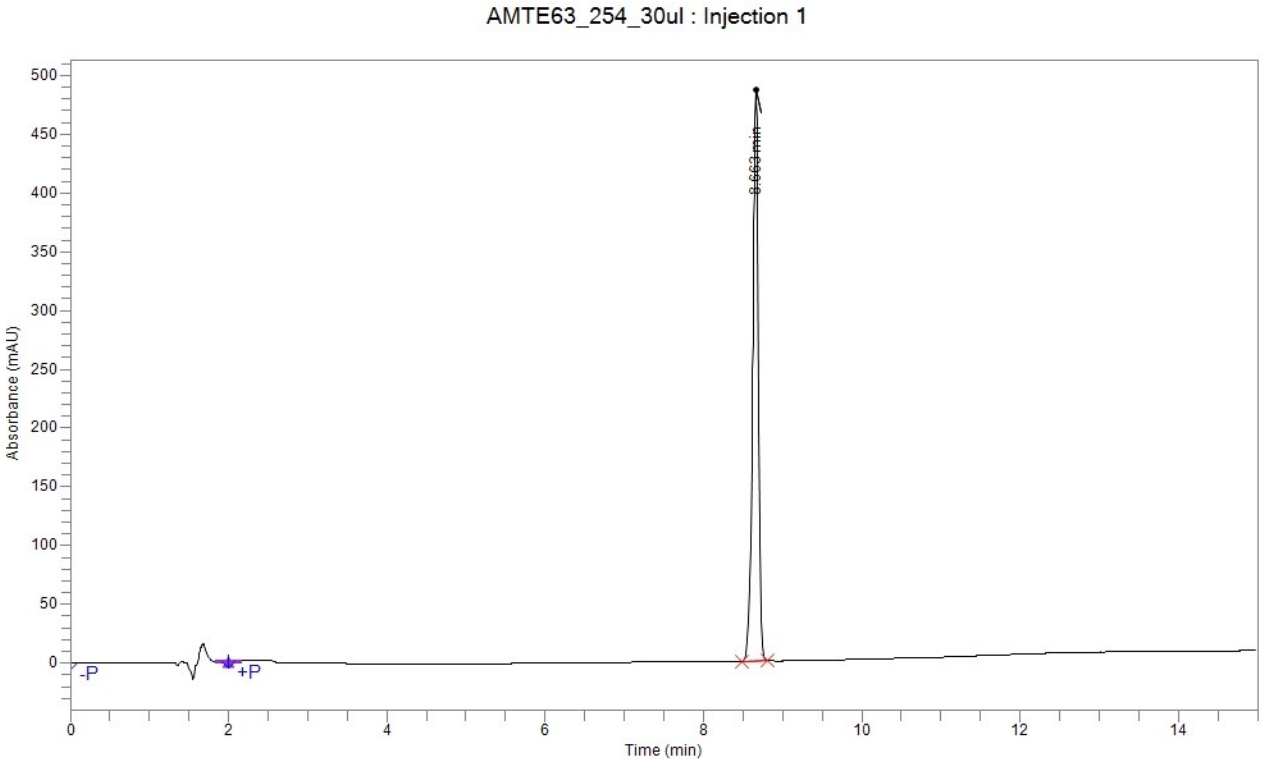

Time	Height	Area	Area %
8.663	486,594.8	2,460,900.5	100.00
**Total**		2,460,900.5	100.00

(*E*)-*N*-[3-Methoxy-4-(2-oxopyrrolidin-1-yl)phenyl]-4-[methyl(prop-2-ynyl)amino]but-2-enamide (41)

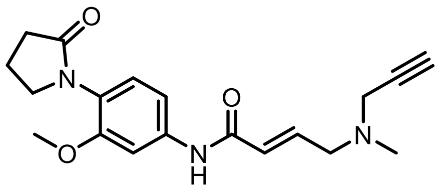

Acquisition Method	Purity short run @254 nm
Acquisition Date/Time	9/20/2021 3:39 pm
Injection Volume	20
Sample Name	AMTE89_254_20
Sample Description	
Batch Description	

AMTE89_254_20 : Injection 1

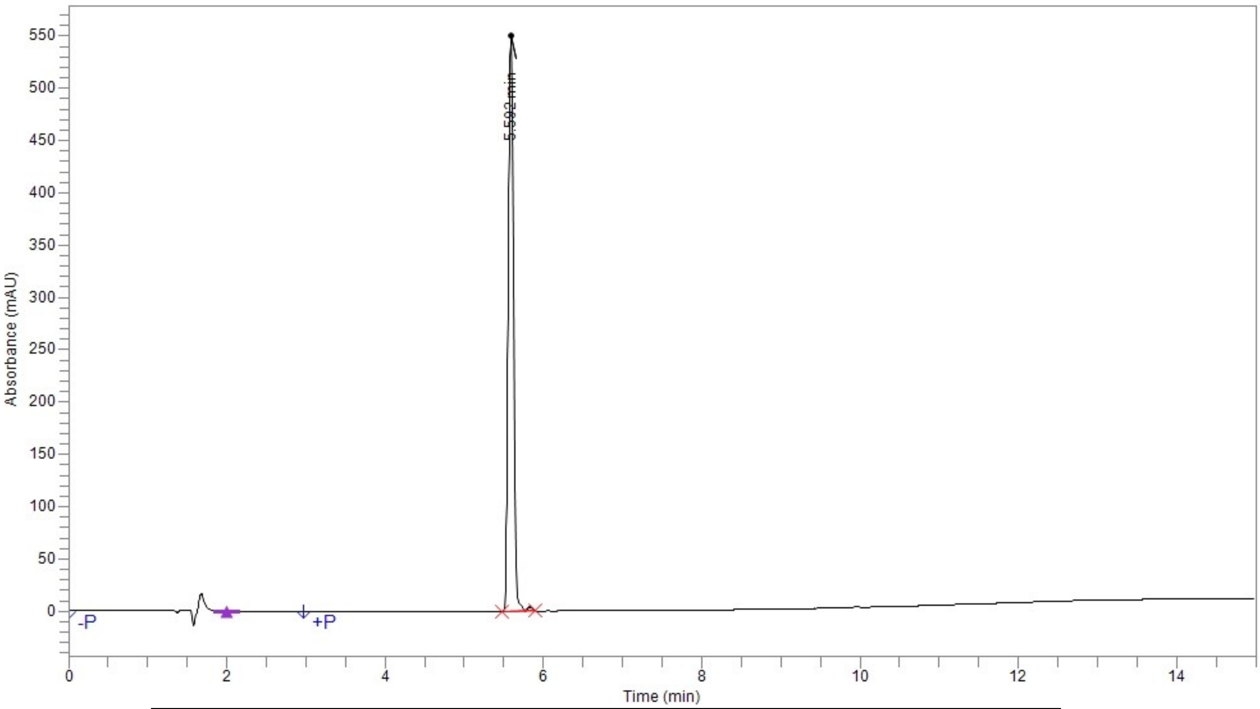

Time	Height	Area	Area %
5.592	551,535.7	2,619,010.1	99.37
5.829	3,803.6	16,590.8	0.63
**Total**		2,635,600.9	100.00

N-[4-(2-Oxopyrrolidin-1-yl)-3-prop-2-ynoxyphenyl]prop-2-enamide (42)

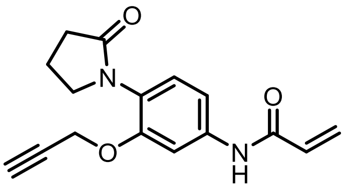

**Sample name:**	MSEC38_10microL		
**Description:**			
**Sample amount:**	0.000	**Sample type:**	Sample
**Instrument:**	Analytical HPLC	**Location:**	P2-F4
**Injection date:**	2022-04-29 15:24:40+01:00	**Injection:**	1 of 1
**Acq. method:**	MSE_PURITY_CHECK_10MICRO.M	**Injection volume:**	10.000
**Analysis method:**	MSE_PURITY_CHECK_10MICRO.M	**Acq. operator:**	SYSTEM
**Last changed:**	2021-10-21 14:08:56+01:00		

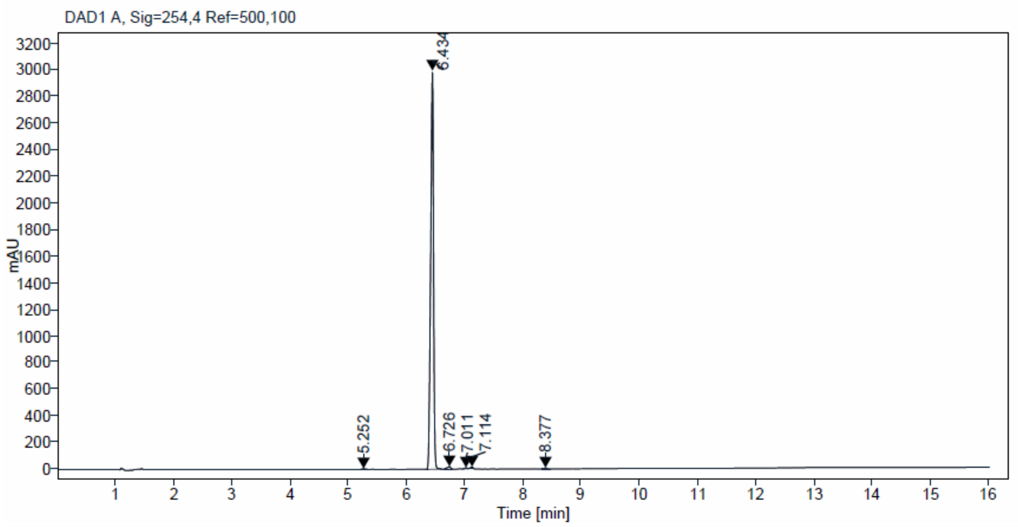

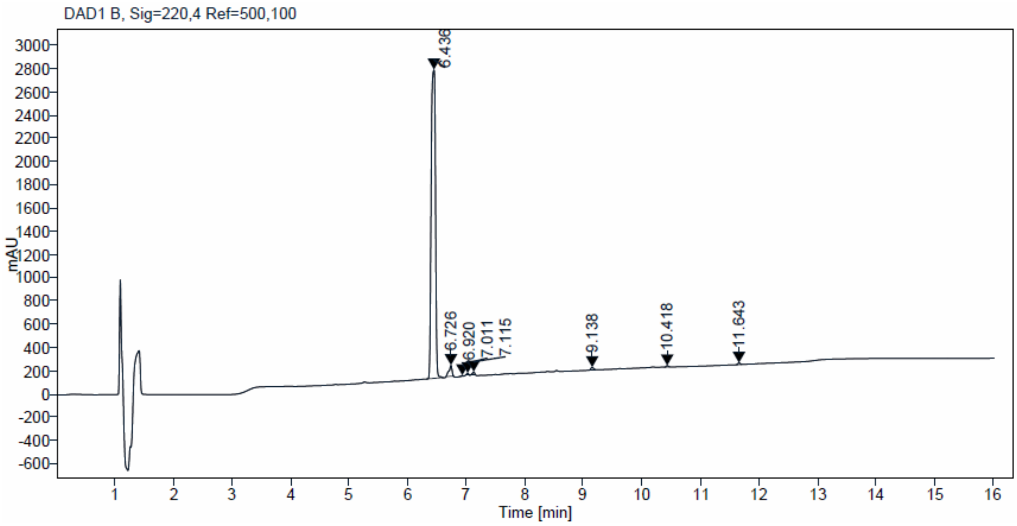

Signal: DAD1 A, Sig=254,4 Ref=500,100

RT [min]	Type	Width [min]	Area	Height	Area%	Name
5.252	MM	0.0619	20.6822	5.5691	0.1990	
6.434	MM	0.0567	10194.9326	2998.5164	98.1182	
6.726	MM	0.0658	79.4493	20.1194	0.7646	
7.011	MM	0.0452	22.4078	8.2657	0.2157	
7.114	MM	0.0454	36.3553	13.3344	0.3499	
8.377	MM	0.0603	36.6352	10.1230	0.3526	
		Sum	10390.4623			

Signal: DAD1 B, Sig=220,4 Ref=500,100

RT [min]	Type	Width [min]	Area	Height	Area%	Name
6.436	MM	0.0837	13333.2305	2654.7192	95.6094	
6.726	MM	0.0588	331.2090	93.9000	2.3750	
6.920	MM	0.0375	16.9600	7.5478	0.1216	
7.011	MM	0.0350	35.9166	17.1057	0.2575	
7.115	MM	0.0452	76.7607	28.3158	0.5504	
9.138	MM	0.0523	71.4154	22.7757	0.5121	
10.418	MM	0.0462	35.0189	12.6276	0.2511	
11.643	MM	0.0492	45.0063	15.2348	0.3227	
		Sum	13945.5173			

References

(1) Geoghegan, K. F.; Dixon, H. B. F.; Rosner, P. J.; Hoth, L. R.; Lanzetti, A. J.; Borzilleri, K. A.; Marr, E. S.; Pezzullo, L. H.; Martin, L. B.; Lemotte, P. K.; McColl, A. S.; Kamath, A. v.; Stroh, J. G. Spontaneous α-N-6-Phosphogluconoylation of a "His Tag" in Escherichia Coli: The Cause of Extra Mass of 258 or 178 Da in Fusion Proteins. *Anal. Biochem.* **1999**, *267*, 169–184. https://doi.org/10.1006/abio.1998.2990.

(2) Fulmer, G. R.; Miller, A. J. M.; Sherden, N. H.; Gottlieb, H. E.; Nudelman, A.; Stoltz, B. M.; Bercaw, J. E.; Goldberg, K. I. NMR Chemical Shifts of Trace Impurities: Common Laboratory Solvents, Organics, and Gases in Deuterated Solvents Relevant to the Organometallic Chemist. *Organometallics* **2010**, *29*, 2176–2179. https://doi.org/10.1021/om100106e.

(3) Giacomelli, G.; de Luca, L.; Porcheddu, A. A Method for Generating Nitrile Oxides from Nitroalkanes: A Microwave Assisted Route for Isoxazoles. *Tetrahedron* **2003**, *59*, 5437–5440. https://doi.org/10.1016/S0040-4020(03)00859-7.

(4) Hewings, D. S. Developing Inhibitors of Bromodomain-Histone Interactions, 2014.

(5) Sekirnik (née Measures), A. R.; Hewings, D. S.; Theodoulou, N. H.; Jursins, L.; Lewendon, K. R.; Jennings, L. E.; Rooney, T. P. C.; Heightman, T. D.; Conway, S. J. Isoxazole-Derived Amino Acids Are Bromodomain-Binding Acetyl-Lysine Mimics: Incorporation into Histone H4 Peptides and Histone H3. *Angew. Chem., Int. Ed.* **2016**, *55*, 8353–8357. https://doi.org/10.1002/anie.201602908.

(6) Jackson, P. L.; Hanson, C. D.; Farrell, A. K.; Butcher, R. J.; Stables, J. P.; Eddington, N. D.; Scott, K. R. Enaminones 12. An Explanation of Anticonvulsant Activity and Toxicity per Linus Pauling's Clathrate Hypothesis. *Eur. J. Med. Chem.* **2012**, *51*, 42–51. https://doi.org/10.1016/j.ejmech.2012.02.003.

(7) Li, G.; Kakarla, R.; Gerritz, S. W. A Fast and Efficient Bromination of Isoxazoles and Pyrazoles by Microwave Irradiation. *Tetrahedron Lett.* **2007**, *48*, 4595–4599. https://doi.org/10.1016/j.tetlet.2007.04.118.

(8) Coombs, E. Covalent Binding to a BRD4 Mutant, University of Oxford, 2018.

(9) Smith, L. H.; Pinkerton, A. B.; Hershberger, P. APELIN RECEPTOR AGONISTS AND METHODS OF USE THEREOF. WO2019032720 (A1), 2019.

(10) Itoh, T.; Fukuda, T.; Fujisawa, T. Preparation of Optically Pure α-Alkyl β-Hydroxy Nitriles by the Bakers' Yeast Reduction. *Bull. Chem. Soc. Jpn.* 1989, pp 3851–3855.

(11) Chan, C. K.; Chang, M. Y. BF3·OEt2-Mediated [1,2]-Aryl Shift: Synthesis of Functionalized α-Arylnitriles via the Bromination/Cyanation/Deformylation of Substituted Deoxybenzoin. *Tetrahedron* **2017**, *73*, 5207–5213. https://doi.org/10.1016/j.tet.2017.07.015.

(12) Kim, B. R.; Lee, H. G.; Kang, S. B.; Jung, K. J.; Sung, G. H.; Kim, J. J.; Lee, S. G.; Yoon, Y. J. Synthesis of β-Ketonitriles, α,β-Alkynones and Biscabinols from Esters Using Tert-Butoxide-Assisted C(=O)-C (i.e., Acyl-C) Coupling under Ambient Conditions. *Tetrahedron* **2013**, *69*, 10331–10336. https://doi.org/10.1016/j.tet.2013.10.007.

(13) Krasavin, M.; Korsakov, M.; Zvonaryova, Z.; Semyonychev, E.; Tuccinardi, T.; Kalinin, S.; Tanç, M.; Supuran, C. T. Human Carbonic Anhydrase Inhibitory Profile of Mono- and Bis-

Sulfonamides Synthesized via a Direct Sulfochlorination of 3- and 4-(Hetero)Arylisoxazol-5-Amine Scaffolds. *Bioorg. Med. Chem.* **2017**, *25*, 1914–1925. https://doi.org/10.1016/j.bmc.2017.02.018.

(14) Lasri, J.; Mukhopadhyay, S.; Charmier, M. A. J. Efficient Regioselective Synthesis of 4- and 5-Substituted Isoxazoles under Thermal and Microwave Conditions. *J. Heterocycl. Chem.* **2008**, *45*, 1385–1389. https://doi.org/10.1002/jhet.5570450521.

(15) Pei, Z.; Mendonca, R.; Gazzard, L.; Pastor, R.; Goon, L.; Gustafson, A.; Vanderporten, E.; Hatzivassiliou, G.; Dement, K.; Cass, R.; Yuen, P. W.; Zhang, Y.; Wu, G.; Lin, X.; Liu, Y.; Sellers, B. D. Aminoisoxazoles as Potent Inhibitors of Tryptophan 2,3-Dioxygenase 2 (TDO2). *ACS Med. Chem. Lett.* **2018**, *9*, 417–421. https://doi.org/10.1021/acsmedchemlett.7b00427.

(16) Chew, W. Methods of Synthesizing Substituted 3-Cyanoquinolines and Intermediates Thereof, 2006.

(17) Dannhardt, G.; Kiefer, W.; Lambrecht, G.; Laufer, S.; Mutschler, E.; Schweiger, J.; Striegep, H. G. Regioisomeric 3-, 4- and 5-Aminomethyl Isoxazoles: Synthesis and Muscarinic Activity. *Eur. J. Med. Chem.* **1995**, *30*, 839–850.

(18) Good, R. H.; Jones, G.; Phipps, J. R. Syntheses with Isoxazoles. Part II. Rearrangement of Isoxazolo[2,3-a]-Pyridinium Salts into 5,6-Dihydro-4*H*-Furo[3,2-b]Pyridin-2-Ones. *J. Chem. Soc., Perkin Trans. 1* **1972**, *5*, 2441–2445.

(19) Quilico, A.; Panizzi, L. *Gazz. Chim. Ital.* **1938**, *68*, 625–635.

(20) Filippakopoulos, P.; Qi, J.; Picaud, S.; Shen, Y.; Smith, W. B.; Fedorov, O.; Morse, E. M.; Keates, T.; Hickman, T. T.; Felletar, I.; Philpott, M.; Munro, S.; McKeown, M. R.; Wang, Y.; Christie, A. L.; West, N.; Cameron, M. J.; Schwartz, B.; Heightman, T. D.; La Thangue, N.; French, C. A.; Wiest, O.; Kung, A. L.; Knapp, S.; Bradner, J. E. Selective Inhibition of BET Bromodomains. *Nature* **2010**, *468*, 1067–1073. https://doi.org/10.1038/nature09504.

(21) Niesen, F. H.; Berglund, H.; Vedadi, M. The Use of Differential Scanning Fluorimetry to Detect Ligand Interactions That Promote Protein Stability. *Nat. Protoc.* **2007**, *2*, 2212–2221. https://doi.org/10.1038/nprot.2007.321.

(22) Grant, E. K.; Fallon, D. J.; Hann, M. M.; Fantom, K. G. M.; Quinn, C.; Zappacosta, F.; Annan, R. S.; Chung, C. wa; Bamborough, P.; Dixon, D. P.; Stacey, P.; House, D.; Patel, V. K.; Tomkinson, N. C. O.; Bush, J. T. A Photoaffinity-Based Fragment-Screening Platform for Efficient Identification of Protein Ligands. *Angew. Chem., Int. Ed.* **2020**, *59*, 21096–21105. https://doi.org/10.1002/ANIE.202008361.

(23) Winter, G. Xia2: An Expert System for Macromolecular Crystallography Data Reduction. *J. Appl. Crystallogr.* **2010**, *43*, 186–190. https://doi.org/https://doi.org/10.1107/S0021889809045701.

(24) Lindorff-Larsen, K.; Piana, S.; Palmo, K.; Maragakis, P.; Klepeis, J. L.; Dror, R. O.; Shaw, D. E. Improved Side-Chain Torsion Potentials for the Amber Ff99SB Protein Force Field. *Proteins* **2010**, *78*, 1950–1958. https://doi.org/10.1002/prot.22711.

(25) Jorgensen, W. L.; Chandrasekhar, J.; Madura, J. D.; Impey, R. W.; Klein, M. L. Comparison of Simple Potential Functions for Simulating Liquid Water. *J. Chem. Phys.* **1983**, *79*, 926– 935.

(26) Abraham, M. J.; Murtola, T.; Schulz, R.; Páall, S.; Smith, J. C.; Hess, B.; Lindah, E. Gromacs: High Performance Molecular Simulations through Multi-Level Parallelism from Laptops to Supercomputers. *SoftwareX* **2015**, *1–2*, 19–25. https://doi.org/10.1016/j.softx.2015.06.001.

(27) Vriend., G.; Krause, R.; Hekkelman, M. L.; Nielsen, J. E. *WHAT-IF web interface*. https://swift.cmbi.umcn.nl/servers/html/index.html.

(28) Sanner, M. F. Python: A Programming Language for Software Integration and Development. *J. Mol. Graph. Model.* **1999**, *17*, 57–61.

(29) Morris, G. M.; Huey, R.; Lindstrom, W.; Sanner, M. F.; Belew, R. K.; Goodsell, D. S.; Olson, A. J. Autodock4 and AutoDockTools4: Automated Docking with Selective Receptor Flexiblity. *J. Comput. Chem.* **2009**, *16*, 2785–2791.
